# Iron-Catalyzed Enantioselective Multicomponent Cross-Couplings of α-Boryl Radicals

**DOI:** 10.1021/acs.orglett.3c03387

**Published:** 2023-11-13

**Authors:** Cassandra R. Youshaw, Ming-Hsiu Yang, Achyut Ranjan Gogoi, Angel Rentería-Gómez, Lei Liu, Lukas M. Morehead, Osvaldo Gutierrez

**Affiliations:** Department of Chemistry, Texas A&M University, College Station, Texas 77843, United States

## Abstract

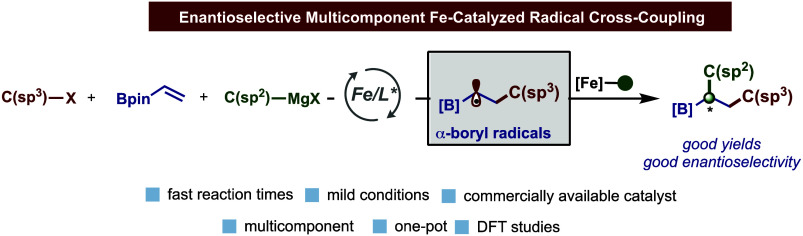

Despite recent interest in the development of iron-catalyzed transformations, methods that use iron-based catalysts capable of controlling the enantioselectivity in carbon–carbon cross-couplings are underdeveloped. Herein, we report a practical and simple protocol that uses commercially available and expensive iron salts in combination with chiral bisphosphine ligands to enable the regio- and enantioselective (up to 91:9) multicomponent cross-coupling of vinyl boronates, (fluoro)alkyl halides, and Grignard reagents. Preliminary mechanistic studies are consistent with rapid formation of an α-boryl radical followed by *reversible* radical addition to monoaryl bisphosphine-Fe(II) and subsequent enantioselective inner-sphere reductive elimination. From a broader perspective, this work provides a blueprint to develop asymmetric Fe-catalyzed multicomponent cross-couplings via the use of alkenes as linchpins to translocate alkyl radicals, modify their steric and electronic properties, and induce stereocontrol.

Transition-metal-catalyzed cross-coupling reactions (CCRs) are recognized as powerful methods for the synthesis of pharmaceuticals, polymers, and commercial products.^1^ Despite pioneering works by Kochi in the 1970s revealing the potential of simple iron salts as catalysts for carbon–carbon CCRs,^2^ this field has been dominated by palladium and nickel catalysis. Considering the inherent attractive features of iron (e.g., low toxicity, earth abundance, environmentally benign, and low cost), in the last two decades, there has been a surge in the development and, equally important, mechanistic understanding of iron-catalyzed cross-couplings.^3−7^ However, in contrast to other transition-metal-catalyzed CCRs, general and practical examples of asymmetric Fe-catalyzed cross-couplings involving sp^3^-hybridized coupling partners are extremely rare.^8−10,11d^ Specifically, seminal reports by the Nakamura and Byers groups disclosed *two-component* asymmetric Fe-catalyzed cross-couplings between alkyl halides and organometallic nucleophiles (Figure 1A). However, these methods are severely limited to the use of activated radical precursors (i.e., α-halo esters and benzyl alkyl chlorides) and the formation of one carbon–carbon bond (i.e., two-component cross-couplings). In part, the lack of general asymmetric Fe-catalyzed CCR methods can be attributed to the high reactivity and instability of alkyl radicals that are prone to undergo a plethora of side reactions (e.g., SET, β-hydride elimination, dimerization, H atom abstractions, etc.) prior to undergoing enantioselective C–C bond formation with a chiral organoiron species. Another major and long-standing challenge in this area is the lack of a detailed understanding of the factors (i.e., speciation, spin state, oxidation state, coordination, etc.) controlling the selectivity and reactivity of iron species under catalytic conditions.

**Figure 1:**
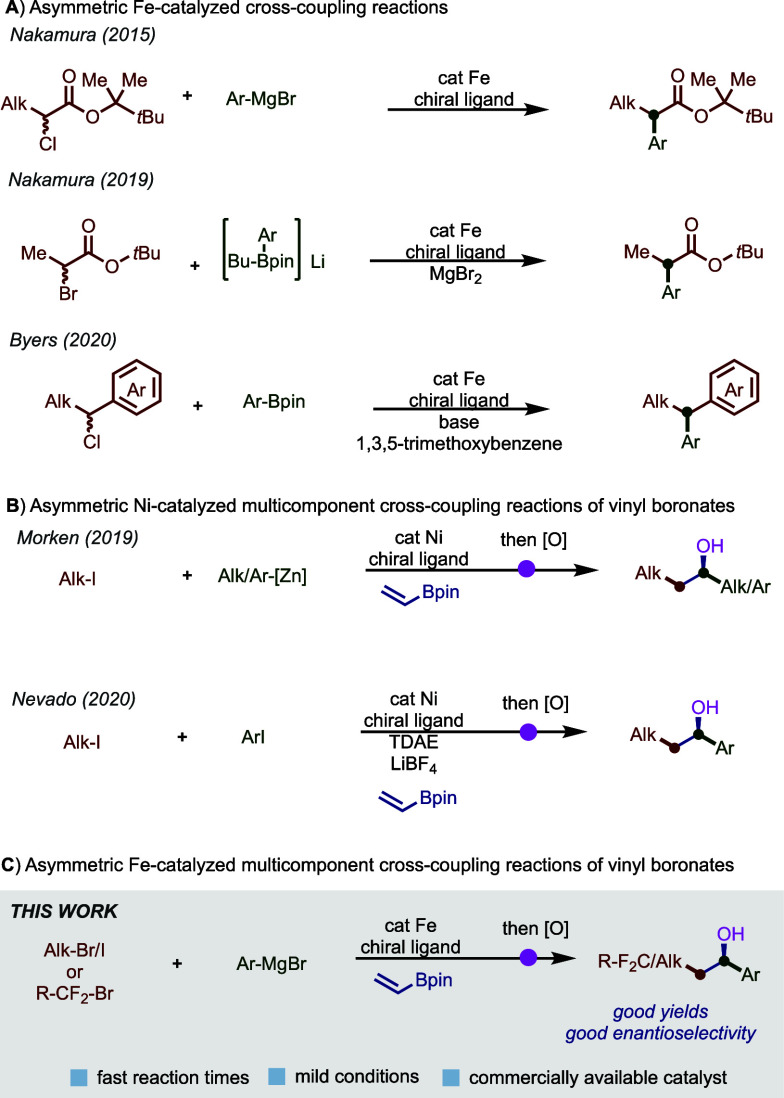
(A) Current state-of-the-art in asymmetric Fe-catalyzed cross-coupling reactions. (B) Asymmetric dicarbofunctionalization of vinyl boronates using nickel catalysis. (C) Asymmetric Fe-catalyzed three-component CCR of vinyl boronates, (fluoro)alkyl halides, and aryl Grignard reagents.

We have initiated a program aimed at using experimental and computational tools to elucidate the mechanisms of iron-catalyzed cross-coupling reactions and, in turn, use this information to develop Fe-catalyzed multicomponent CCRs.^11^ Inspired by these results and elegant work by the Morken^12^ and Nevado^13^ groups on nickel-catalyzed asymmetric CCRs (Figure 1B), we hypothesized that vinyl boronates could serve as effective linchpins to promote radical capture, relay, and *enantioselective* cross-coupling of α-boryl radicals with well-defined *chiral bisphosphine-iron* species (Figure 1C). If successful, this work can not only complement existing nickel-based catalytic systems for the synthesis of diverse and chiral alkyl boron reagents (Figure 1B) but also open the door for the development of multicomponent iron-catalyzed asymmetric cross-couplings. Herein we report the first general and broadly applicable use of commercially available iron salts and chiral bisphosphine ligands to promote asymmetric multicomponent radical cross-coupling between a range of (difluoro)alkyl halides, Grignard reagents, and vinyl boronates.

Based on prior work by us^11f^ and others,^14−17^ we initiated our studies using *tert*-butyl bromide, vinylB(pin), and 4-fluorophenylmagnesium bromide as model substrates to investigate the asymmetric three-component iron-catalyzed CCR. We hypothesized that, under slow addition of Grignard reagent, we could generate the active *chiral* monoaryl and bisaryl BenzP*Fe(II) species (i.e., without promoting chiral ligand dissociation) that are responsible for radical generation and C–C bond formation, respectively.^11f,18^ At the same time, this iron mechanistic manifold would permit electron-rich *tert*-butyl radical to add regioselectively to the electron-deficient vinyl boronate to form a stabilized α-boryl radical.^11f^ In turn, effective capture of α-boryl radical with chiral monoaryl BenzP*Fe(II)^18^ species could lead to enantioselective C–C bond formation. Gratifyingly, after extensive experimentation (see the Supporting Information), we identified the use of Fe(acac)_3_ and (*R*,*R*)-BenzP* **L1** as a suitable combination to form the desired multicomponent cross-coupling product **4** in good yield (56%) and enantioselectivity (80:20 er) (entry 1). The major byproducts are biaryl formation (∼25%) and the Grignard reagent coupled to B(pin) (∼8%). The two-component cross-coupling and beta-hydride elimination products were not observed. Notably, despite the complexity of three components and potential side products arising from the formation of alkyl radicals in the presence of organometallic reagents and potentially redox active iron species (i.e., H atom transfer, β-hydride elimination, polymerization, two-component cross-coupling, etc.) the observed enantioselectivities are up to par with current state-of-the-art *two-component* iron-catalyzed cross-coupling reactions.^8−10^ Moreover, as shown in entry 2, changing the alkyl radical precursor to *tert*-butyl iodide decreased both the yield and enantioselectivity, while using chiral bisphosphine ligand **L2** slightly improved enantioselectivity but decreased product yield (entry 3). Ligands that proved effective in related nickel-catalyzed transformations by Morken^12^ and Nevado^13^ were significantly less effective in this iron-catalyzed transformation (entries 4 and 5). In addition to the choice of ligand, we also found a drastic effect imposed by the precatalysts on both the yield and enantioselectivity. Specifically, while iron(III) halide salts (FeBr_3_ and FeCl_3_) provided no enantiocontrol, FeBr_2_ was significantly less effective than Fe(acac)_3_ (entries 6–8). The use of other ethereal solvents, including noncoordinating solvent 2-Me-THF, which is known to accelerate transmetalation of monoaryl BenzP*Fe(II) to the corresponding, and nonselective, bisaryl BenzP*Fe(II),^18^ led to significantly lower enantioselectivity (entries 9 and 10). These results are consistent with the role of solvent in controlling *iron speciation* and, as a consequence, enantioselectivity in BenzP*-iron-catalyzed cross-couplings.^18^ Presumably, noncoordinating solvents decrease the concentration of the monoaryl BenP*Fe(II) species that is required for trapping alkyl radicals and promote enantioselective C–C bond formation (*vide infra*). Consistent with this hypothesis, dimethylacetamide (DMA), a strongly coordinating solvent, significantly improved the enantioselectivity (up to 91:9) but decreased the product yields (only 19% of the desired cross-coupled product). A palladium precatalyst was also used to examine the potential catalytic activity of this reaction but did not yield the desired product (entry 14). Finally, control experiments confirmed the need for both the iron precatalyst and the ligand to drive the enantioselective multicomponent transformation (entries 15–17). Thus, after extensive screening, we moved forward with BenzP* in this transformation given the balanced initial three-component product yield and enantioselectivity for further substrate exploration.

With the optimized conditions in hand, we turned our attention to exploring the reaction scope by first varying the nature of the Grignard nucleophile (Figure 2). Overall the reaction tolerates electron-rich and electron-poor aryl Grignard reagents, leading to the desired three-component products in good yields (up to 69% *over two steps*) and enantioselectivities (up to 91:9 er). In addition, this method tolerates a range of electron-rich and -poor *meta*- and *para*-substituents including C–Cl bonds that can be further handles in cross-coupling reactions (**10** and **11**).^19,20^ Finally, extended π-systems work well in this transformation (**15**–**18**). Notably, X-ray analysis of **18** unambiguously determined the absolute stereochemistry of the major enantiomer as (*S*). Next, we turned our sights to the radical precursor scope (Figure 3). The sterically encumbered cyclic tertiary 1-iodoadamantyl radical precursor provided improved enantioselectivity (**19**; 85:15 er) over the acyclic tertiary system using the same Grignard reagent (68:32 er; Table 1, entry 2). Further, the tertiary radical precursor bearing an oxygen-containing cyclohexyl ring gave the desired product **20** in good yield and modest enantioselectivity, revealing the potential applicability toward the practical synthesis of heteroatom-containing compounds with relevance to pharmaceutical research.^21^ Notably, the reaction was selective toward tertiary alkyl bromides in the presence of other carbon–halogen bonds including C(sp^2^)–Br and primary C(sp^3^)–Cl bonds (**21** and **24**). In addition, other representative acyclic tertiary alkyl radical precursors yield the desired products in good yields and modest enantioselectivities (**22**, **23**, **25**). Given that ∼20% of drugs on the market contain at least one fluorine atom,^22−24^ combined with the lack of enantioselective three-component methods using vinyl borates and difluoroalkyl radical precursors, we were also interested in investigating the possibility of rapidly producing diverse fluorine-containing enantioeneriched alkylboron reagents (inset; Figure 3). Indeed, in contrast to prior methods, fluorinated radical precursors were tolerated in the enantioselective iron-catalyzed multicomponent radical cross-coupling reaction, producing the enantioenriched fluorinated organoboron products (**26**, **27**, and **28**), although slightly lower yields and modest enantioselectivity were observed. Presumably, the lower yields are attributed to polarity mismatch between electron-deficient vinylB(pin) and electron-poor (poly)fluoroalkyl radicals that open opportunities for side reactions prior to undergoing the desired Giese addition to vinylB(pin).^25,26^

**Table 1:**
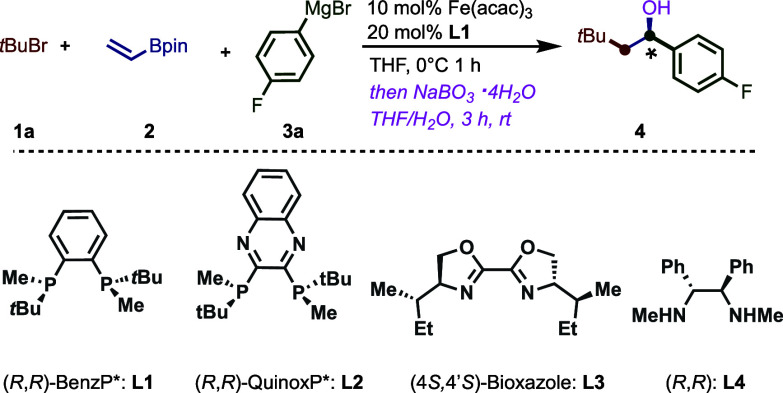
Optimization of Asymmetric Dicarbofunctionalization of Vinyl Boronates Using Iron Catalysis^a^

Entry	Deviations	Yield	er
1	none	56	80:20
2	*t*BuI instead of *t*BuBr	43	68:32
3	**L2**	41	87:13
4	**L3**	21	84:16
5	**L4**	7	74:26
6	using FeBr_3_ (10 mol %)	28	racemic
7	using FeBr_2_ (10 mol %)	50	75:25
8	using FeCl_3_ (10 mol %)	20	racemic
9	using 2-MeTHF	51	racemic
10	using (iPr)_2_O	43	54.5:45.5
11	using DMA	19	91:9
12	at –15 °C	39	87:13
13	addition of **3a** over 2 h	43	77:23
14	using Pd(PPh_3_)_2_Cl_2_ (10 mol %)	0	N/A
15	no Fe(acac)_3_	0	N/A
16	no **L1**	0	N/A
17	no Fe(acac)_3_ and no **L1**	0	N/A

a Reaction conditions: All reactions were performed using *t*-butyl bromide **1** (0.2 mmol, 2 equiv), vinylboronic acid pinacol ester **2** (0.1 mmol, 1 equiv), 4-fluorophenyl Grignard **3** (0.2 mmol, 2 equiv), and 1 mL of 1.0 M solution of THF. Aryl Grignard was added dropwise over an hour via syringe pump. Crude product was directly oxidized with 3.0 equiv of NaBO_3_·4H_2_O in 1:1 H_2_O:THF and purified. Enantiomeric ration (e.r.) was determined by HPLC using a chiral stationary phase on Daicel’s CHIRALPAK AD-H column.

**Figure 2:**
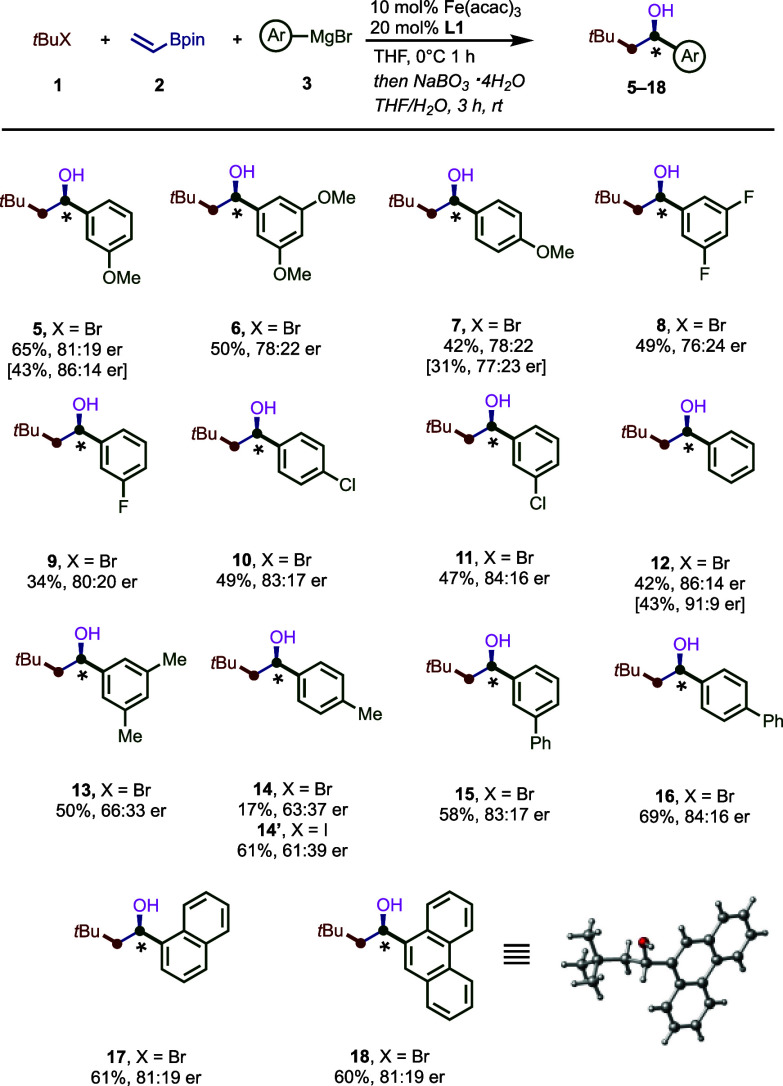
Scope of Grignard nucleophile in the three-component dicarbofunctionalization with vinylboronic acid pinacol ester. Reactions were carried out under the optimized conditions (Table 1, entry 1) with 0.2 mmol of **2**. Er determined by HPLC using a chiral stationary phase. Yields and enantiomeric ratios using ligand (*R*,*R*)-QuinoxP* are shown in brackets.

**Figure 3:**
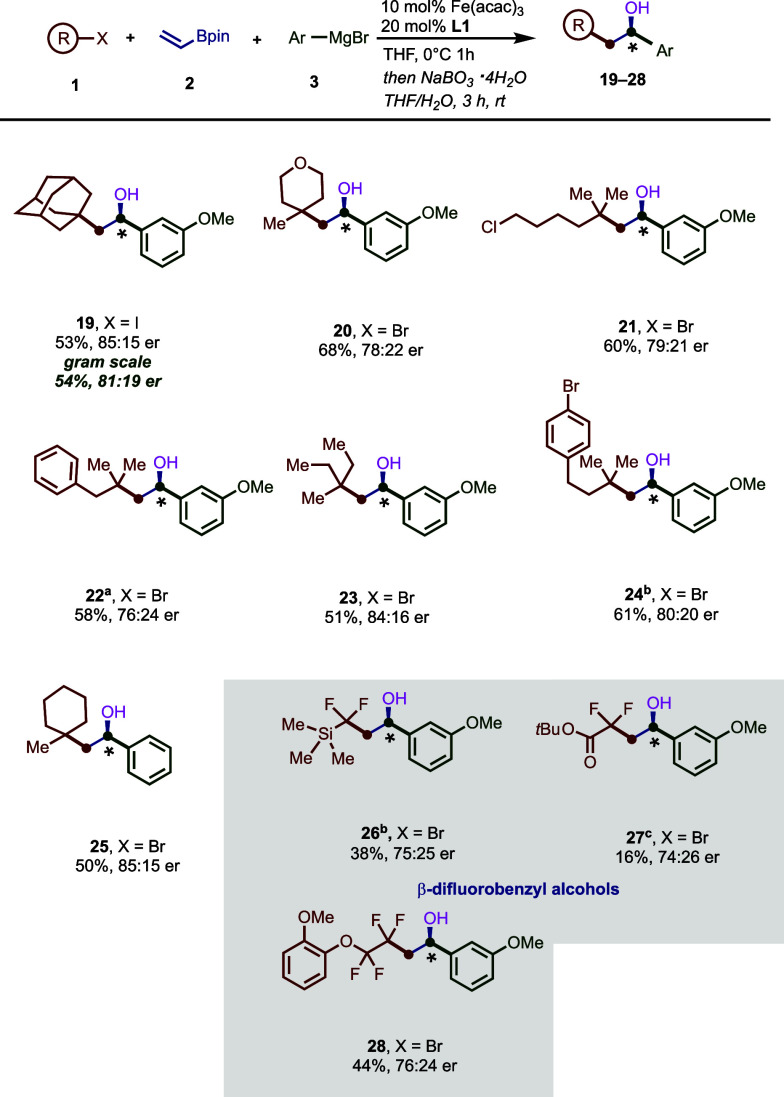
Scope of the alkyl halides in the three-component dicarbofunctionalization with vinylboronic acid pinacol ester and 3-methoxyphenyl magnesium bromide. Reactions were carried out under the optimized conditions (Table 1, entry 1). Enantiomeric ratio (e.r.) determined by HPLC using a chiral stationary phase. ^a^Using 2.5 equiv of **1**. ^b^Using 20 mol % Fe(acac)_3_ and 40 mol % (*R*,*R*)-BenzP*. ^c^Using 4 equiv of **1** and 4.5 equiv of **3**.

On the basis on prior experimental and mechanistic studies on Fe-catalyzed cross-coupling reactions^18,11f^ we turned to DFT calculations to gain insight into the nature of C–C bond formation and the origin of enantioselectivity (Figure 4). Neidig has shown that bisaryl BenzP*-Fe(II) **C** could generate alkyl radicals. In turn, this tBu•alkyl radical, **II**, can then undergo irreversible Giese addition to the vinyl boronate **2** via a low energy barrier (10.8 kcal/mol) to form the α-boryl radical **III**. Finally, akin to 1,2-bis(cyclohexylohosphino)ethane-Fe-catalyzed cross-couplings,^11f^ this α-boryl radical **III** can then undergo spin-selective radical addition/dissociation with **B** to establish an equilibrium with the corresponding Fe(III) intermediate **IV** prior to undergoing C–C bond formation. Thus, consistent with prior work, the equilibrium with the chiral BenzP* ligand also favors the pentacoordinate Fe(III) intermediate (**IV**) as opposed to the dissociated α-boryl radical and iron(II) **B**. We hypothesize that this shift in equilibrium can have pronounced effects in preventing deleterious and unwanted radical pathways stemming from noncoordinated α-boryl radicals (i.e., polymerization, H atom abstraction, SET oxidation, etc.). Finally, Fe(III) **IV** intermediate is poised to undergo a rapid and enantioselective C–C bond to form the desired enantioenriched multicomponent product **4** and Fe(I) **I** species that can then restart the catalytic cycle.^11a,27^ Consistent with the observed enantioselectivity, the energy difference between the lowest energy diastereomeric transition states for the reductive elimination step is ∼1.0 kcal/mol (Figure 4B) in favor of the (*S*)-product. As shown in Figure 4B, closer inspection of the lowest energy diastereomeric transition states revealed a favorable C–H···O interaction in **TS**^**S**^**-IV-I**, which is absent in the competing transition state leading to a minor (*R*) product. In addition, the repulsion between the *t*Bu group and the Bpin group is more prominent in the transition state leading to the (*R*) product as evident from the NCI (non-covalent interaction) plots.

**Figure 4:**
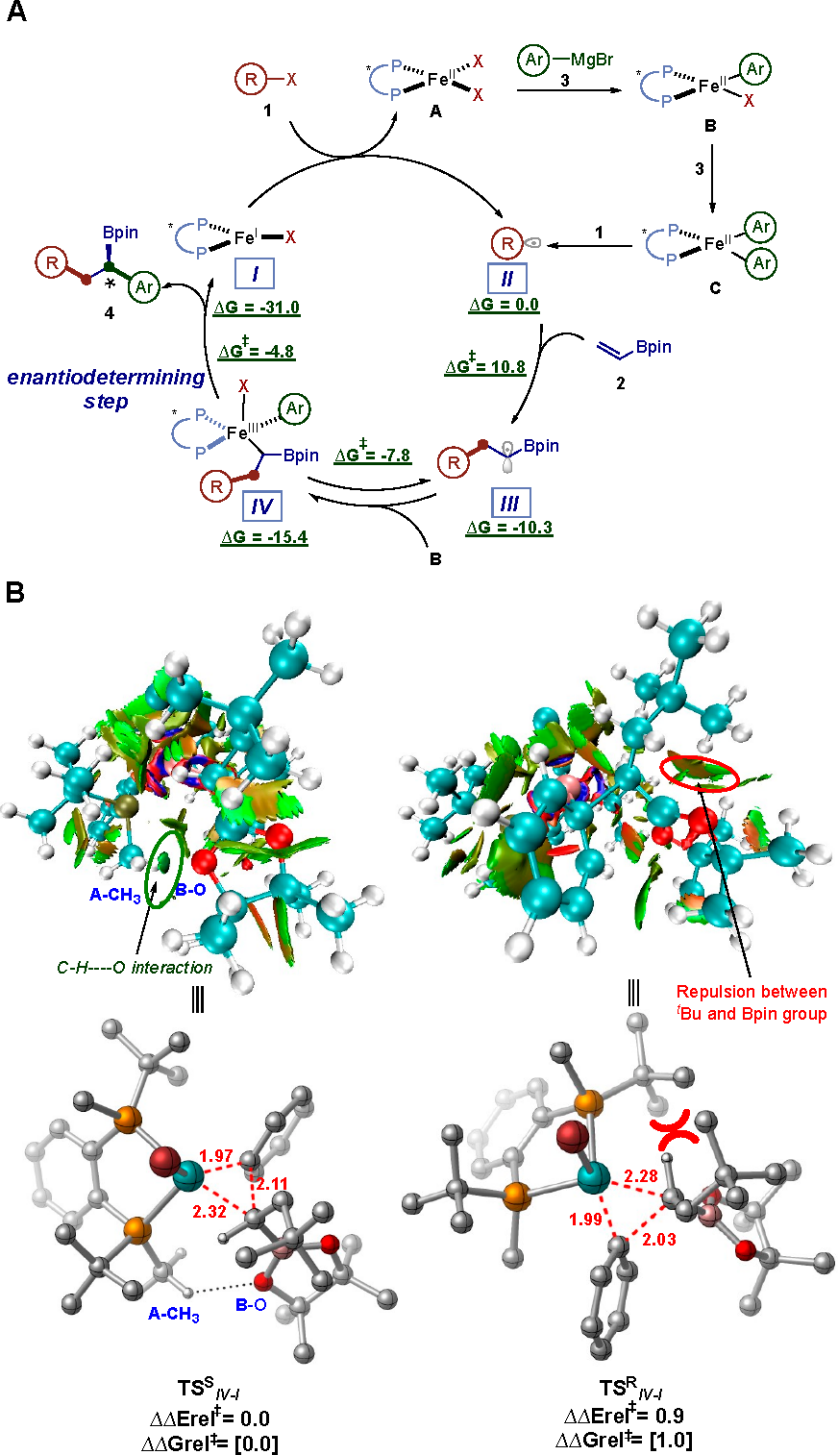
Working mechanistic manifold based on experiments and computational studies.

In conclusion, we have developed an enantioselective iron-catalyzed multicomponent radical cross-coupling reaction that enables 1,2-dicarbofunctionalization of vinyl boronates with diverse (fluoro)alkyl halides and aryl Grignard reagents. Mechanisitic studies are consistent with radical translocation to form a α-boryl radial that is rapidly intercepted by a chiral monoaryl bisphosphine-iron leading to enantioselective carbon–carbon bond formation. Further studies are underway to expand the scope of asymmetric multicomponent radical cross-coupling reactions with well-defined and readily available iron catalysts.

## Data Availability

The data underlying this study are available in the published article and its Supporting Information.

## Supporting Information Available

Experimental procedures, product characterizations, crystallographic data, and copies of the ^1^H and ^13^C NMR spectra (PDF)
