# Supplementary material for: Iron-Catalyzed Enantioselective Multicomponent Cross-Couplings of α-Boryl Radicals
Source: Org Lett. 2023 Nov 13;25(46):8320–5. doi: 10.1021/acs.orglett.3c03387 (PMC10863393; doi:10.1021/acs.orglett.3c03387)
Supplement: Supplementary file 1 — ol3c03387_si_001.pdf [file ol3c03387_si_001.pdf]

Supplementary Materials for

Iron-Catalyzed Enantioselective Multicomponent Cross-Couplings of α-Boryl Radicals

Cassandra R. Youshaw,‡ Ming-Hsiu Yang,‡ Achyut Ranjan Gogoi,# Angel Rentería-Gómez,# Lei Liu, Lukas M. Morehead, Osvaldo Gutierrez*

Department of Chemistry, Texas A&M University, College Station, TX, 77843

Table of Contents

1. General Considerations **S3**

2. General Procedure for Iron-Catalyzed 1,2-Dicarbofunctionalization of VinylB(pin) **S3**

3. Screenings of Reaction Conditions **S5**

4. Current Limitation **S11**

5. Preparation and Characterization of Materials **S1–S8** **S12**

6. List of Compounds **4–28** **S14**

7. Experimental Spectra **S44**

8. Crystallographic Data **S77**

9. Computational Methods, Energies, and Coordinates **S79**

10. References **S202**

1. General Considerations

Unless otherwise stated, all non-aqueous reactions were carried out under an atmosphere of dry nitrogen in oven (105 °C) or flame-dried glassware. When necessary, solvents and reagents were dried prior to use. Tetrahydrofuran (THF) was dried by passage through activated alumina in Mbraun's 5 solvent purification system. All work-up and purification procedures used reagent grade solvents purchased from VWR, Sigma-Aldrich, or Fisher. Organometallic reagents were purchased from Sigma-Aldrich and STREM Chemicals. Analytical thin layer chromatography (TLC) was performed on Silicycle 250 µm silica-gel F-254 plates. Silica gel chromatography was performed on Biotage Selekt systems with prepacked silica-gel cartridges (Biotage). Purification via flash column chromatography was performed on silica gel 60 (230-400 mesh ASTM). ^{1}H NMR and ^{13}C NMR spectra were recorded on Avance neo (400 MHz) NMR spectrometer. Chemical shifts (δ) are reported in parts per million (ppm) relative to the internal residual solvent resonance peak δ 7.26 ($CDCl_3$) and δ 0.00 (TMS) for ^{1}H and δ 77.16 ($CDCl_3$) and δ 0.00 (TMS) for ^{13}C. Data are reported as follows: chemical shift, multiplicity (s = singlet, d = doublet, t = triplet, q = quartet, p = quintet, b.s. = broad singlet, m = multiplet, dd = doublet of doublets, dt = doublet of triplets, ddd = doublet of doublet of doublets, dtd = doublet of triplet of doublets, coupling constants (*J*) are reported in Hertz (Hz), and number of protons. High Resolution Mass (HRMS) spectra using ESI and APCI modes were obtained using a QExactive Focus from Thermo Scientific. IR spectra were recorded on a Thermo Nicolet NEXUS 670 FTIR and are reported in wavenumbers (cm^{-1}). High Performance Liquid Chromatography (HPLC) was performed on an Agilent 1260 Infinity II system. Preparative High Performance Liquid Chromatography was measured on an Agilent 1260 Infinity II Preparative LC System with a CHIRALCEL® OD Preparative column, 50 mm ID, 500 mm length, 20 µm particle size.

2. General Procedure for Iron-Catalyzed 1,2-Dicarbofunctionalization of VinylB(pin)

Procedure A: Reaction Optimization (Including Table 1)

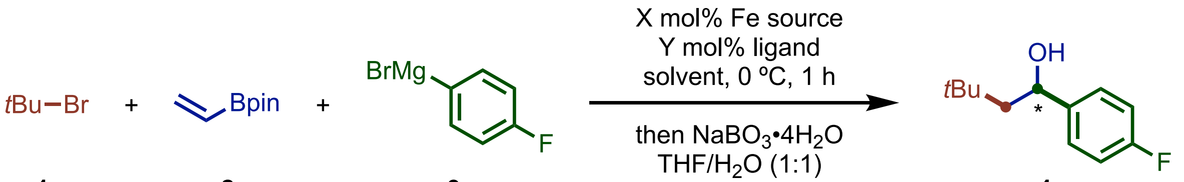

1a 2 3a 4

In an argon-filled glovebox, an oven-dried 5 mL microwave vial with a stir bar was charged with Fe source, ligand, *tert*-butyl bromide **1a** (or *tert*-butyl iodide), and vinylboronic acid pinacol ester **2** (0.1 mmol, 1.0 equiv.). The vial was sealed with a rubber septa and then brought out of the glovebox. Anhydrous THF (0.1 mL) was added by a syringe outside the glovebox. The solution mixture was stirred at room temperature (rt) for 5 min under nitrogen gas and then cooled to 0 °C. 4-fluorophenylmagnesium bromide solution **3a** (1.0 M solution in THF, 2.0 equiv.) was added dropwise into the reaction mixture over 1 hour using a syringe pump at 0 °C. After complete addition of **3**, the reaction mixture was stirred for an additional 30 min at 0 °C. The reaction was quenched with 1.0 M HCl (1.0 mL) at 0 °C and then warmed to rt. The aqueous layer was extracted with EtOAc (3 x 2.0 mL). The combined organic layer was filtered through a plug of silica gel (pre-rinsed with EtOAc) and the filtrate was concentrated under reduced pressure to give the residue (Bpin-containing product). This crude product was dissolved in a cosolvent of THF (1.0 mL) and H_2O (1.0 mL) at rt and then $NaBO_3•4H_2O$ (3.0 equiv.) was added as a solid portion at rt. The resulting reaction mixture was stirred at rt for 3 hours and then was added with EtOAc (2.0 mL). After being stirred for 3 min, the

two phases were separated once the stirring was stopped. The organic phase was collected and the aqueous layer was extracted with EtOAc (3 x 2.0 mL). The combined organic phase was filtered through a plug of silica gel (pre-rinsed with EtOAc), and the filtrate was concentrated under reduced pressure to give the residue (alcohol product). The residue was dissolved in $CDCl_3$ and was added with CH_2Br_2 (as an internal standard, 7 µL) for ^1H NMR analysis. Removal of the solvents in *vacuo* and purification by Isolera™ Flash Chromatography Systems or preparative thin layer chromatography (PTLC) with an elution system of hexanes and EtOAc provided the desired product **4**. The enantiomeric ratio was determined by HPLC with the Daicel CHIRALPAK® AD-H column (hexane/2-propanol 98:2, 1.0 mL/min). The positions of two enantiomers on the HPLC chromatogram were referred to those of the racemic compound **4**.

Procedure B: Catalytic Reaction of Substrates in Figures 2 and 3

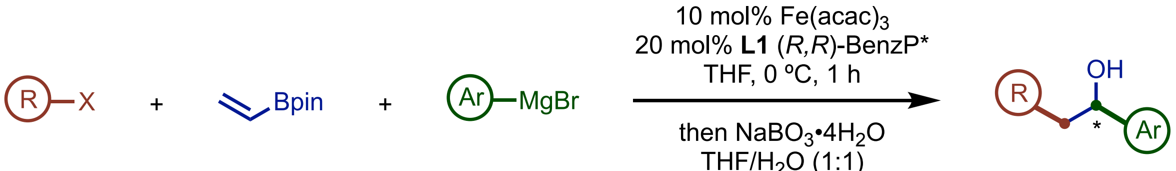

1 **2** **3** **5–28**

In an argon-filled glovebox, an oven-dried 5 mL microwave vial with a stir bar was charged with Fe source (0.02 mmol, 10 mol%), **L1** (*R*,*R*)-(+)-1,2-Bis(t-butylmethylphosphino)benzene ((*R*,*R*)-BenzP*) (0.04 mmol, 20 mol%), alkyl halide (0.4 mmol, 2.0 equiv.), and vinylboronic acid pinacol ester **2** (0.2 mmol, 1.0 equiv). The vial was sealed with a rubber septa and then brought out of the glovebox. Anhydrous THF (0.2 mL) was added by a syringe outside the glovebox. The solution mixture was stirred at room temperature (rt) for 5 min under nitrogen gas and then cooled to 0 °C. Grignard reagent (ArMgBr, 0.3 – 1.0 M solution in THF, 2.0 equiv.) was added dropwise into the reaction mixture over 1 hour using a syringe pump at 0 °C. After complete addition of Grignard reagent, the reaction mixture was stirred for an additional 30 min at 0 °C. The reaction was quenched with 1.0 M HCl (1.0 mL) at 0 °C and then warmed to rt. The aqueous layer was extracted with EtOAc (3 x 2.0 mL). The combined organic layer was filtered through a plug of silica gel (pre-rinsed with EtOAc) and the filtrate was concentrated under reduced pressure to give the residue (Bpin-containing product). This crude product was dissolved in a cosolvent of THF (2.0 mL) and H_2O (2.0 mL) at rt and then $NaBO_3$•$4H_2O$ (3.0 equiv.) was added as a solid portion at rt. The resulting reaction mixture was stirred at rt for 3 hours and then was added with EtOAc (2.0 mL). After being stirred for 3 min, the two phases were separated once the stirring was stopped. The organic phase was collected and the aqueous layer was extracted with EtOAc (3 x 2.0 mL). The combined organic phase was filtered through a plug of silica gel (pre-rinsed with EtOAc), and the filtrate was concentrated under reduced pressure to give the residue (alcohol product). The residue was dissolved in $CDCl_3$ and was added with CH_2Br_2 (as an internal standard, 7 µL) for ^1H NMR analysis. Removal of the solvents in *vacuo* and purification by Isolera™ Flash Chromatography Systems or column chromatography on silica gel with a gradient elution of hexanes/EtOAc/DCM provided the desired product **5**–**28**. The enantiomeric ratio values were determined by HPLC equipped with a chiral column. The positions of two enantiomers on the HPLC chromatogram were referred to those of the racemate of compound **5**–**28**. Racemic compound was prepared according to the general procedure C. Absolute stereochemistry was assigned by analogy (see product **18**).

Procedure C: Preparation of Racemic Compounds

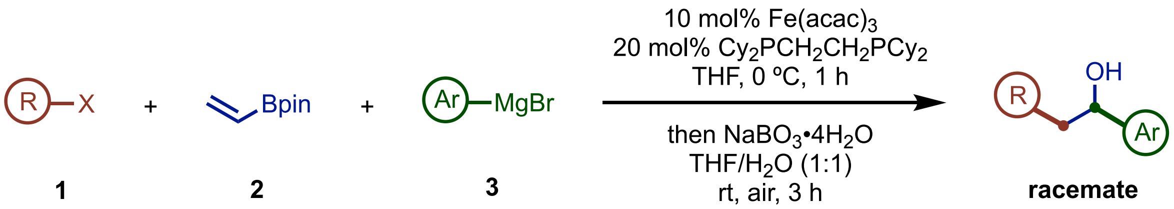

Preparation of racemic products were carried using a modified previously reported procedure.[1] In an argon-filled glovebox, an oven-dried 5 mL microwave vial with a stir bar was charged with $Fe(acac)_3$ (0.02 mmol, 10 mol%), 1,2-Bis(dicyclohexylphosphino)ethane (0.04 mmol, 20 mol%), alkyl halide (0.4 mmol, 2.0 equiv.), and vinylboronic acid pinacol ester **2** (0.20 mmol, 1.0 equiv). The vial was sealed with a rubber septa and then brought out of the glovebox. Anhydrous THF (0.2 mL) was added by a syringe outside the glovebox. The solution mixture was stirred at room temperature (rt) for 5 min under nitrogen gas and then cooled to 0 °C. Grignard reagent (ArMgBr, 0.3 – 1.0 M solution in THF, 2.0 equiv.) was added dropwise into the reaction mixture over 1 hour using a syringe pump at 0 °C. After complete addition of Grignard reagent, the reaction mixture was stirred for an additional 30 min at 0 °C. The reaction was quenched with 1.0 M HCl (1.0 mL) at 0 °C and then warmed to rt. The aqueous layer was extracted with EtOAc (3 x 2.0 mL). The combined organic layer was filtered through a plug of silica gel (pre-rinsed with EtOAc) and the filtrate was concentrated under reduced pressure to give the residue (Bpin-containing product). This crude product was dissolved in a cosolvent of THF (2.0 mL) and H_2O (2.0 mL) at rt and then $NaBO_3•4H_2O$ (3.0 equiv.) was added as a solid portion at rt. The resulting reaction mixture was stirred at rt for 3 hours and then was added with EtOAc (2.0 mL). After being stirred for 3 min, the two phases were separated once the stirring was stopped. The organic phase was collected and the aqueous layer was extracted with EtOAc (3 x 2.0 mL). The combined organic phase was filtered through a plug of silica gel (pre-rinsed with EtOAc), and the filtrate was concentrated under reduced pressure to give the residue (alcohol product). The residue was purified by Isolera™ Flash Chromatography Systems or column chromatography on silica gel with a gradient elution of hexanes/EtOAc/DCM to afford the desired product.

3. Screenings of Reaction Conditions

Table S1. Screening of iron sources for iron-catalyzed 1,2-dicarbofunctionalization.

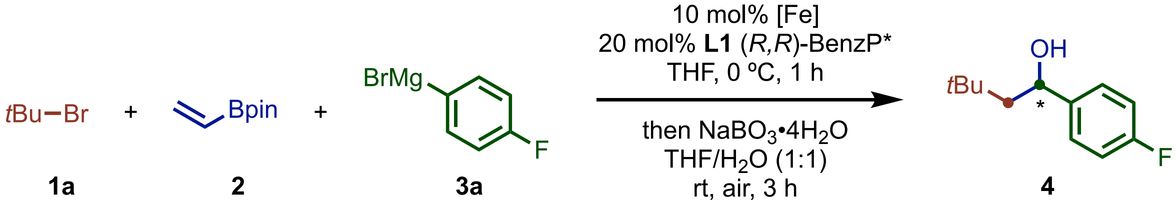

entry	[Fe]	^{1}H NMR (%)[a]	isolated (%)[b]	enantiomeric ratio (er)[c]
1	$Fe(acac)_3$	61	56	20:80
2	$FeCl_3$	21	20	49:51
3	$FeBr_3$	33	28	49:51

4	$FeCl_2$	33	32	22:78
5	$FeBr_2$	55	50	25:75
6	FeI_2	30	29	20:80
7	FeF_2	0	0	N/A[d]
8	$Fe(dibm)_3$	50	43	19:81
9	$Fe(OTf)_3$	0	0	N/A
10	$Fe(OTf)_2$	0	0	N/A
11	$Fe(OAc)_2$	31	29	22:78
12	$FeCp(CO)_2I$	0	0	N/A
13[e]	FeI_2	35	33	25:75

Reaction conditions: vinylboronic acid pinacol ester **2** (0.1 mmol), *tert*-butyl bromide **1a** (0.2 mmol), 4-fluorophenylmagnesium bromide solution **3a** (1.0 M in THF, 0.2 mL), and THF (0.1 mL) followed by oxidation with $NaBO_3•4H_2O$ (0.3 mmol), THF (1.0 mL), and H_2O (1.0 mL). [a]Crude yields were determined by ^1H NMR analysis with CH_2Br_2 as an internal standard. [b]Isolated yields were obtained after the purification of compound **4** by Biotage Selekt flash system and PTLC. [c]Enantiomeric ratio (er) values were determined by HPLC with the Daicel CHIRALPAK® AD-H column (hexane/2-propanol 98:2, 1.0 mL/min). [d]"N/A" means that the ratio was not determined. [e]Reaction was carried out on a 0.2 mmol scale of **2** with a pre-activation procedure: FeI_2 and **L1** were stirred in THF (0.2 mL) at rt for 30 min, and then stock solutions of **1** and **2** in THF (50 µL for each one) were added sequentially into the reaction vessel at rt. After that, the general procedure A was applied.

Table S2. Screening of commercially available ligands for iron-catalyzed 1,2-dicarbofunctionalization.

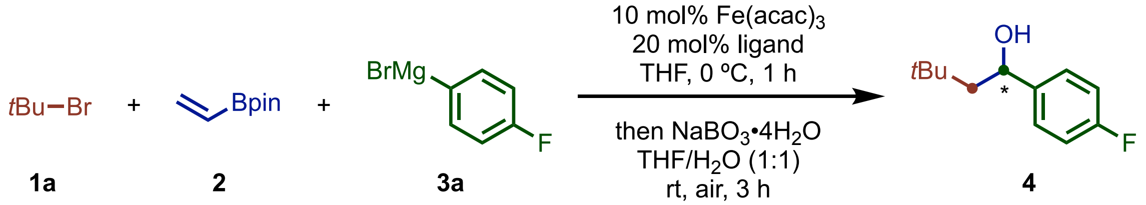

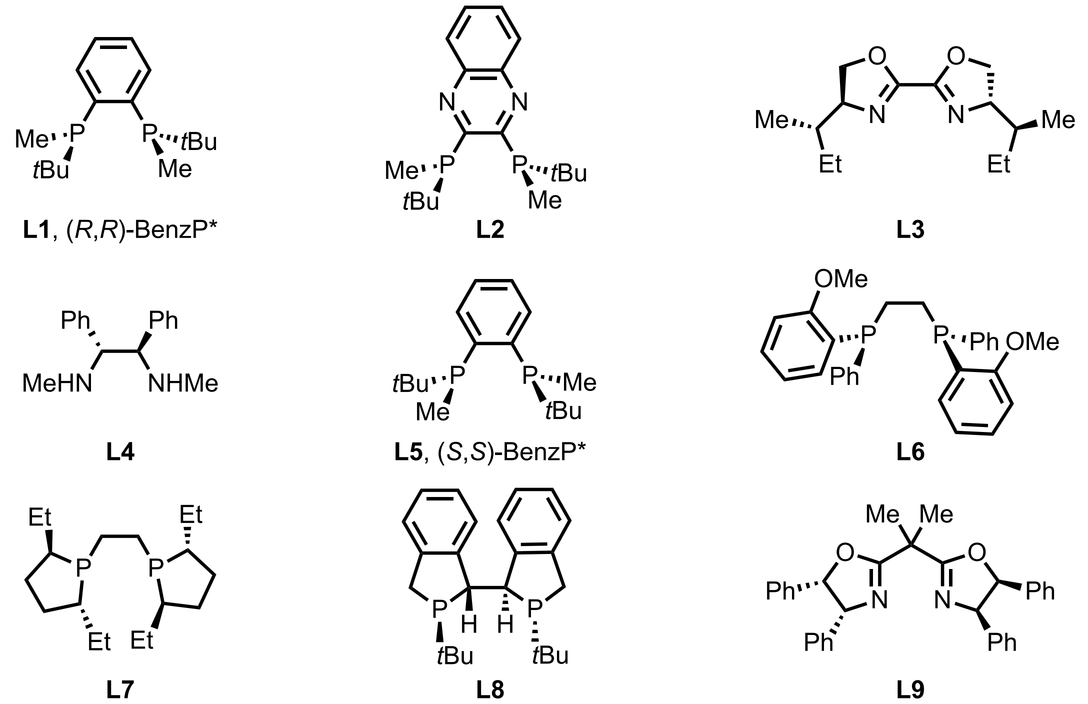

entry	ligand	^{1}H NMR (%)[a]	isolated (%)[b]	enantiomeric ratio (er)[c]
1	**L1**	61	56	20:80
2	**L2**	45	41	13:87
3	**L3**	25	21	16:84
4	**L4**	20	7	26:74
5	**L5**	45	44	79:21
6	**L6**	28	26	36:64
7	**L7**	36	22	30:70
8	**L8**	17	11	26:74

9	**L9**	20	17	87:13

Ligands that provided the product **4** in a yield (<10%) by ^{1}H NMR analysis

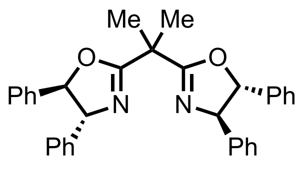
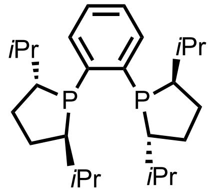
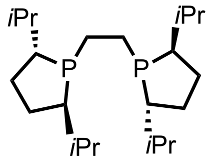
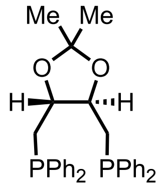

**L10**	**L11**	**L12**	**L13**
8.6%	7.4%	7%	7%

Ligands that provided the product **4** in 0% yield

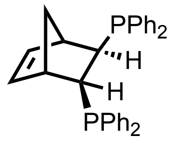

L14

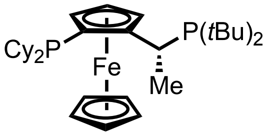

L15

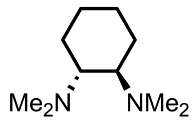

L16

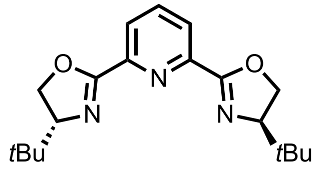

L17

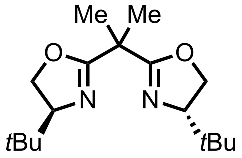

L18

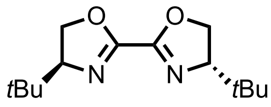

L19

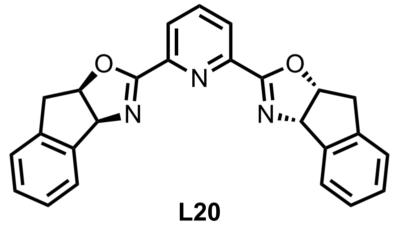

L20

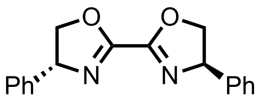

L21[d]

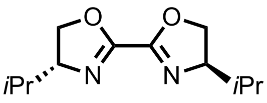

L22[d]

Figure S1. Reaction conditions: vinylboronic acid pinacol ester **2** (0.1 mmol), *tert*-butyl bromide **1a** (0.2 mmol), 4-fluorophenylmagnesium bromide solution **3a** (1.0 M in THF, 0.2 mL), and THF (0.1 mL) followed by oxidation with $NaBO_3 \cdot 4H_2O$ (0.3 mmol), THF (1.0 mL), and H_2O (1.0 mL). [a]Crude yields were determined by ^{1}H NMR analysis with CH_2Br_2 as an internal standard. [b]Isolated yields were obtained after the purification of compound **4** by Biotage Selekt flash system and PTLC. [c]Enantiomeric ratio (er) values were determined by HPLC with the Daicel CHIRALPAK® AD-H column (hexane/2-propanol 98:2, 1.0 mL/min). [d]*t*BuI (0.4 mmol), **2** (0.2 mmol), $FeBr_2$ (10 mol%), ligand (12 mol%), and $LiBF_4$ (1.0 equiv.) were used with a pre-activation procedure: $FeBr_2$ and ligand were stirred in THF (0.2 mL) at rt for 30 min, and then stock solutions of *t*BuI and **2** in THF (0.15 mL and 0.14 mL, respectively) were added sequentially into the reaction vessel at rt. After that, the general procedure A was applied.

Table S3. Screening of the ratios of $Fe(acac)_3$ to **L1** for iron-catalyzed 1,2-dicarbofunctionalization.

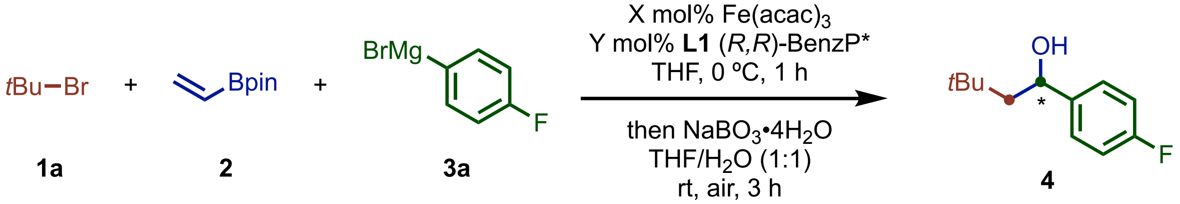

entry	X/Y (mol%)	^{1}H NMR (%)[a]	isolated (%)[b]	enantiomeric ratio (er)[c]
1	5/20	20	19	18:82
2	10/20	61	56	20:80
3	20/20	52	51	23:77
4	30/20	21	20	25:75
5	40/20	19	14	22:78
6	10/10	22	24	23:77
7	30/30	57	56	23:77
8	40/40	44	41	21:79
9	15/30	61	61	24:76
10	20/40	67	66	24:76

Reaction conditions: vinylboronic acid pinacol ester **2** (0.1 mmol), *tert*-butyl bromide **1a** (0.2 mmol), 4-fluorophenylmagnesium bromide solution **3a** (1.0 M in THF, 0.2 mL), and THF (0.1 mL) followed by oxidation with $NaBO_3 \cdot 4H_2O$ (0.3 mmol), THF (1.0 mL), and H_2O (1.0 mL). [a]Crude yields were determined by ^{1}H NMR analysis with CH_2Br_2 as an internal standard. [b]Isolated yields were obtained after the purification of compound **4** by Biotage Selekt flash systems and PTLC. [c]Enantiomeric ratio (er) values were determined by HPLC with the Daicel CHIRALPAK® AD-H column (hexane/2-propanol 98:2, 1.0 mL/min).

Table S4. Screening of solvents for iron-catalyzed 1,2-dicarbofunctionalization.

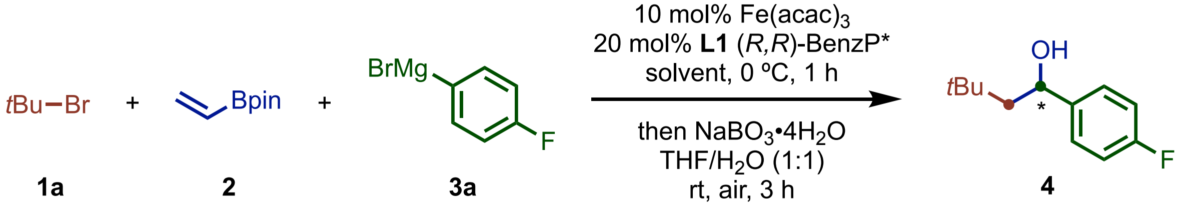

entry	solvent	^{1}H NMR (%)[a]	isolated (%)[b]	enantiomeric ratio (er)[c]
1	THF	61	56	20:80
2	1,4-dioxane	57	56	25:75
3	*n*Bu-O-*n*Bu	58	56	39:61
4	*i*Pr-O-*i*Pr	49	43	46:54
5	diethyl ether	25	19	46:54
6	DME	25	25	23:77
7	2-Me-THF	52	51	49:51
8	*t*BuOMe	57	56	49:51
9	DMA	20	19	9:91
10	NMP	<10	1	N/A
11	THF:DMA (4:1)	20	10	18:82
12	anisole (C_6H_5OMe)	48	47	22:78
13	toluene (C_6H_5Me)	46	45	50:50
14	DCE	31	28	50:50

Reaction conditions: vinylboronic acid pinacol ester **2** (0.1 mmol), *tert*-butyl bromide **1a** (0.2 mmol), 4-fluorophenylmagnesium bromide solution **3a** (1.0 M in THF, 0.2 mL), and THF (0.1 mL) followed by oxidation with $NaBO_3$•$4H_2O$ (0.3 mmol), THF (1.0 mL), and H_2O (1.0 mL). [a]Crude yields were determined by ^{1}H NMR analysis with CH_2Br_2 as an internal standard. [b]Isolated yields were obtained after the purification of compound **4** by Biotage Selekt flash systems and PTLC. [c]Enantiomeric ratio (er) values were determined by HPLC with the Daicel CHIRALPAK® AD-H column (hexane/2-propanol 98:2, 1.0 mL/min).

4. Current Limitations

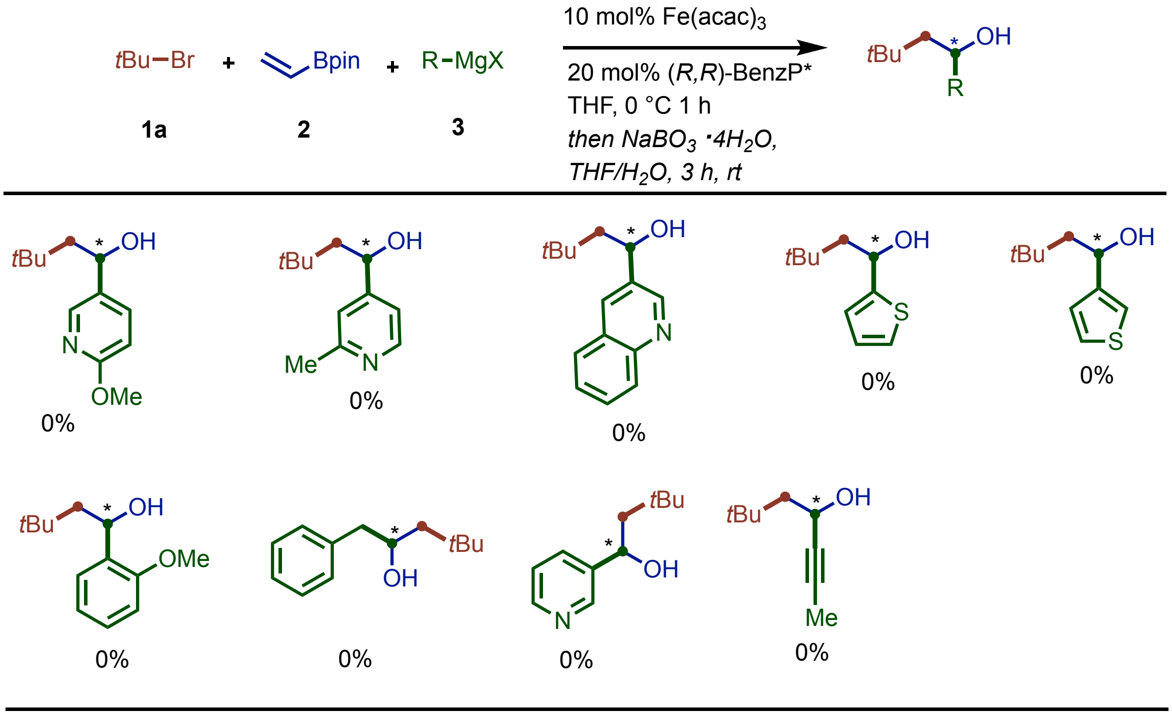

Figure S2. Limitation of the Grignard reagent scope. Reaction conditions: *Tert*-butyl bromide **1a** (0.4 mmol, 2.0 equiv.), vinylboronic acid pinacol ester **2** (0.2 mmol, 1.0 equiv.), Grignard reagent (RMgBr, 0.25 – 2.0 M solution in THF, 2.0 equiv.), and THF (0.2 mL) followed by oxidation with $NaBO_3{\bullet}4H_2O$ (0.6 mmol), THF (2.0 mL), and H_2O (2.0 mL). Yields were determined by ^1H NMR analysis with CH_2Br_2 as an internal standard.

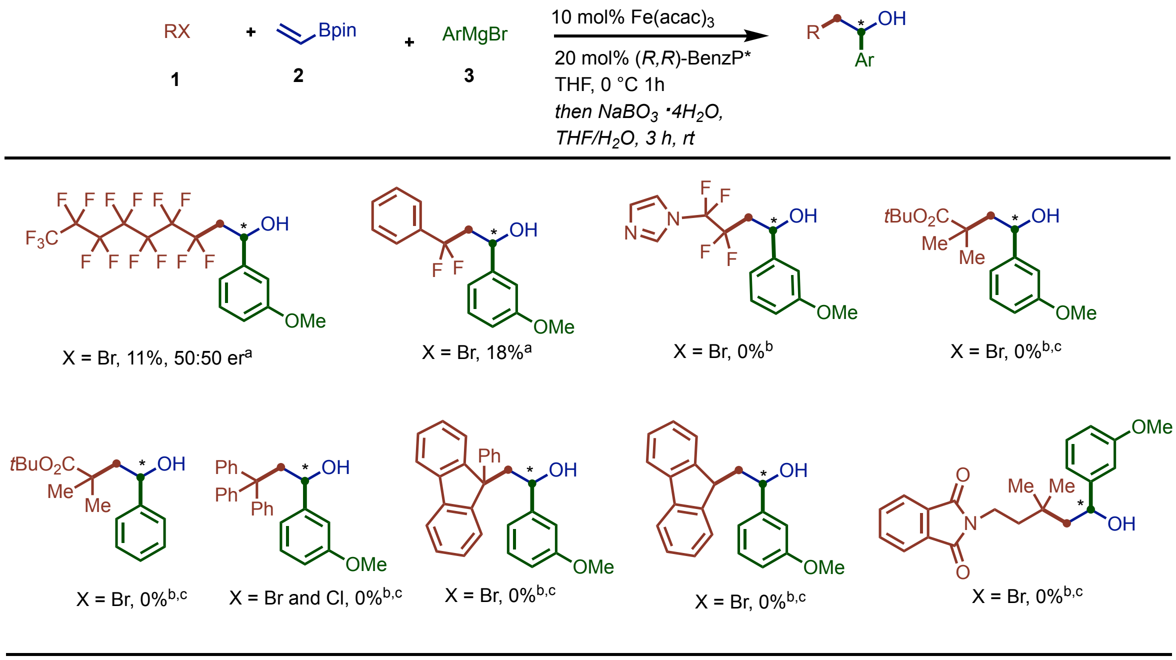

Figure S3. Limitation of the alkyl halide scope. Reaction conditions: Alkyl halide **1** (0.4 mmol, 2.0 equiv.), vinylboronic acid pinacol ester **2** (0.2 mmol, 1.0 equiv.), Grignard reagent (ArMgBr, 1.0 M in THF, 0.4 mL, 2.0 equiv.), and THF (0.2 mL) followed by oxidation with $NaBO_3$•$4H_2O$ (0.6 mmol), THF (2.0 mL), and H_2O (2.0 mL). [a]Isolated yields were obtained after the purification of compounds by Biotage Selekt flash system and PTLC. Enantiomeric ratio (er) values were determined by HPLC with the Daicel CHIRALPAK® IC column (hexane/2-propanol 95:5, 0.5 mL/min). [b]Crude yields were determined by ^{1}H NMR analysis with CH_2Br_2 as an internal standard. [c]Ligand 1,2-Bis(dicyclohexylphosphino)ethane was used instead of (*R*,*R*)-BenzP*.

5. Preparation and characterization of materials

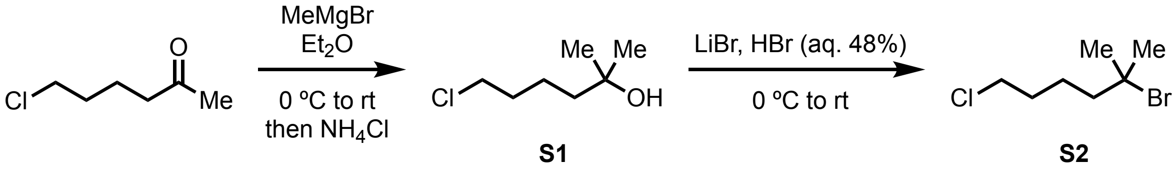

6-chloro-2-methylhexan-2-ol (S1)

The compound **S1** was prepared using a modified known procedure[2] using 6-chlorohexan-2-one (2.4 mL, 18 mmol), methylmagnesium bromide (7.3 mL, 3.0 M solution in Et_2O, 22 mmol), and anhydrous Et_2O (20.0 mL). After purification by column chromatography on silica gel with gradient 0% to 5% EtOAc in DCM, the product **S1** was obtained as a colorless oil (2.2 g, 81% yield). Spectra matched those reported previously.[2]

^{1}H NMR (400 MHz, $CDCl_3$): δ = 3.55 (t, *J* = 6.7 Hz, 2H), 1.83 – 1.76 (m, 2H), 1.56 – 1.45 (m, 4H), 1.23 (s, 6H).

5-bromo-1-chloro-5-methylhexane (S2)

The compound **S2** was prepared following the known procedure[3] using the compound **S1** (1.5 g, 10.0 mmol), LiBr (1.7 g, 20.0 mmol), and HBr (4.0 mL, 48 wt.% in water). After rapid purification by

short-path flash column chromatography on silica gel with pentane, the product **S2** was obtained as a colorless oil (1.8 g, 84% yield). Spectra matched those reported previously.[4]
^{1}H NMR (400 MHz, $CDCl_3$): δ = 3.57 (t, *J* = 6.6 Hz, 2H), 1.85 – 1.78 (m, 4H), 1.76 (s, 6H), 1.72 – 1.63 (m, 2H).

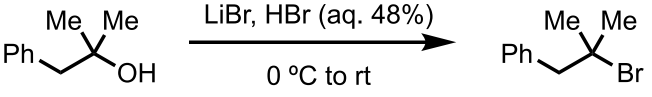

S3

(2-bromo-2-methylpropyl)benzene (S3)
The compound **S3** was prepared following the known procedure[5] using 2-methyl-1-phenyl-2-propanol (3.1 g, 20.0 mmol), LiBr (3.5 g, 40.0 mmol), and HBr (8.0 mL, 48 wt.% in water). After rapid purification by short-path flash column chromatography on silica gel with hexanes, the product **S3** was obtained as a colorless oil (3.8 g, 89% yield). Spectra matched those reported previously.[5]
^{1}H NMR (400 MHz, $CDCl_3$): δ = 7.34 – 7.25 (m, 5H), 3.20 (s, 2H), 1.77 (s, 6H).

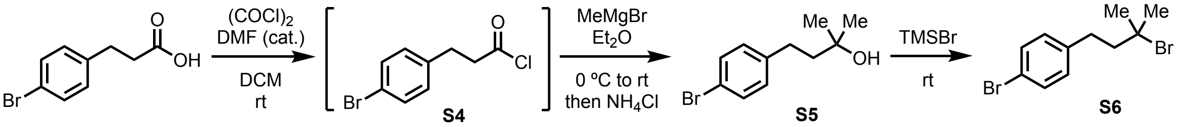

4-(4-bromophenyl)-2-methylbutan-2-ol (S5)
The compound **S5** was prepared following the known procedure[6] using 3-(4-bromophenyl)propionic acid (4.1 g, 18.0 mmol), oxalyl chloride (1.7 mL, 20.0 mmol), DMF (50.0 μL), and DCM (20.0 mL) for formation of the acyl chloride **S4**, followed by the treatment with methylmagnesium bromide (20.0 mL, 3 M solution in Et_2O, 60.0 mmol) and anhydrous Et_2O (20.0 mL). After purification by column chromatography on silica gel with gradient 10% to 30% EtOAc in hexanes, the product **S5** was obtained as a colorless solid (3.5 g, 80% yield). Spectra matched those reported previously.[6]
^{1}H NMR (400 MHz, $CDCl_3$): δ = 7.39 (d, *J* = 8.3 Hz, 2H), 7.07 (d, *J* = 8.3 Hz, 2H), 2.68 – 2.64 (m, 2H), 1.77 – 1.73 (m, 2H), 1.34 (b.s., 1H), 1.29 (s, 6H);
^{13}CNMR (100 MHz, $CDCl_3$): δ = 141.7, 131.5, 130.2, 119.5, 70.9, 45.6, 30.3, 29.5;
HRMS (APCI$^+$): calcd for $C_{11}H_{14}Br$ $[M+H-H_2O]^+$ *m/z* = 225.0273; found 225.0267.

1-bromo-4-(3-bromo-3-methylbutyl)benzene (S6)
The compound **S6** was prepared following a similar procedure[7] using the compound **S5** (0.58 g, 2.4 mmol) and bromotrimethylsilane (0.34 mL, 2.6 mmol). After concentration, the product **S6** was obtained as a pale yellow solid (0.69 g, 94% yield) and used directly without further purification (pure by NMR analysis).
^{1}H NMR (400 MHz, $CDCl_3$): δ = 7.41 (d, *J* = 8.2 Hz, 2H), 7.09 (d, *J* = 8.2 Hz, 2H), 2.83 – 2.79 (m, 2H), 2.06 – 2.02 (m, 2H), 1.81 (s, 6H);
^{13}CNMR (100 MHz, $CDCl_3$): δ = 140.7, 131.6, 130.3, 119.8, 67.3, 49.3, 34.4, 32.5;
FTIR (cm^{-1}): 2965, 2928, 1488, 1369, 1072, 1011, 857, 808, 635, 531, 495;
HRMS (+APCI, -APCI, +ESI, -ESI): Not found; GC-MS: 304 (52%), 306 (100%), and 308 (48%), this pattern matched the molecular formula containing two Br atoms with isotopes ^{79}Br and ^{81}Br in the abundance of 1:1 ratio in nature;

mp. 54–56 °C.

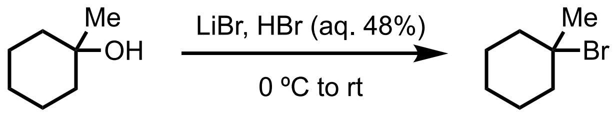

S7

bromo-1-methylcyclohexane (S7)

The compound **S7** was prepared following the known procedure[8] using 1-methylcyclohexanol (2.3 g, 20.0 mmol), LiBr (3.5 g, 40.0 mmol), and HBr (8.0 mL, 48 wt.% in water). After rapid purification by short-path flash column chromatography on silica gel with pentane, the product **S7** was obtained as a colorless oil (2.3 g, 65% yield). Spectra matched those reported previously.[8]

^{1}H NMR (400 MHz, $CDCl_3$): δ = 2.10 – 2.07 (m, 2H), 1.83 (s, 3H), 1.79 – 1.57 (m, 5H), 1.50 – 1.43 (m, 2H), 1.31 – 1.18 (m, 1H).

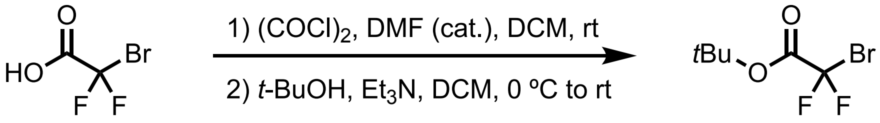

S8

***tert*-butyl 2-bromo-2,2-difluoroacetate (S8)**

The compound **S8** was prepared following the known procedure[9] using bromodifluoroaceticacid (5.2 g, 30.0 mmol), oxalyl chloride (2.9 mL, 33.0 mmol), DMF (50.0 µL), *t*BuOH (5.7 mL, 60.0 mmol), Et_3N (4.6 mL, 33.0 mmol), and DCM (65.0 mL). After purification by column chromatography on silica gel with pentane, the product **S8** was obtained as a colorless oil (2.5 g, 36% yield). Spectra matched those reported previously.[9]

^{1}H NMR (400 MHz, $CDCl_3$): δ = 1.57 (s, 9H);

^{19}F NMR (376 MHz, $CDCl_3$): δ = -60.9 (s, 2F).

6. List of compounds 4–28

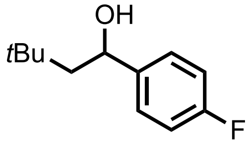

(*S*)-1-(4-fluorophenyl)-3,3-dimethylbutan-1-ol (4):

Compound **4** was synthesized following the general procedure B, using *tert*-butyl bromide (54.8 mg, 0.4 mmol), vinylboronic acid pinacol ester (30.8 mg, 0.2 mmol), and 4-fluorophenylmagnesium bromide (0.4 mL, 1.0 M solution in THF, 0.4 mmol), followed by the oxidation step using $NaBO_3•4H_2O$ (92.3 mg, 0.6 mmol), THF (2 mL), and H_2O (2 mL). The product **4** was obtained as a white solid (22.1 mg, 56% yield) after purification by flash column chromatography on silica gel with gradient 0% to 20% EtOAc in hexane.

^{1}H NMR (400 MHz, $CDCl_3$): δ = 7.33 – 7.29 (m, 2H), 7.02 (t, *J* = 8.7 Hz, 2H), 4.82 (dd, *J* = 8.3, 3.7 Hz, 1H), 1.77 – 1.71 (m, 2H), 1.57 (dd, *J* = 14.5, 3.7 Hz, 1H), 0.99 (s, 9H);

^{13}C NMR (100 MHz, $CDCl_3$): δ = 162.2 (d, *J* = 243.7 Hz), 142.3 (d, *J* = 3.2 Hz), 127.5 (d, *J* = 8.0 Hz), 115.4 (d, *J* = 21.1 Hz), 72.0, 53.1, 30.6, 30.3;

^{19}F NMR (376 MHz, $CDCl_3$): δ = -115.43;
FTIR (cm^{-1}): 3374, 2952, 1508, 1222, 834, 544;
HRMS (APCI$^-$): calcd for $C_{12}H_{17}FO$ $[M]^-$ *m/z* = 195.1183; found 195.1180;
mp: 51–53 °C;
$[\alpha]_D^{18.9}$ = -41.45 (*c* = 0.690, EtOAc, *l* = 100 mm);
HPLC (CHIRALPAK® AD-H (4.6 x 250 mm), 5 mic, hexane/2-propanol 98:2, 1 mL/min) t_R 7.519 (minor), 8.076 (major), 20:80 er, 60% ee.

Racemic Material

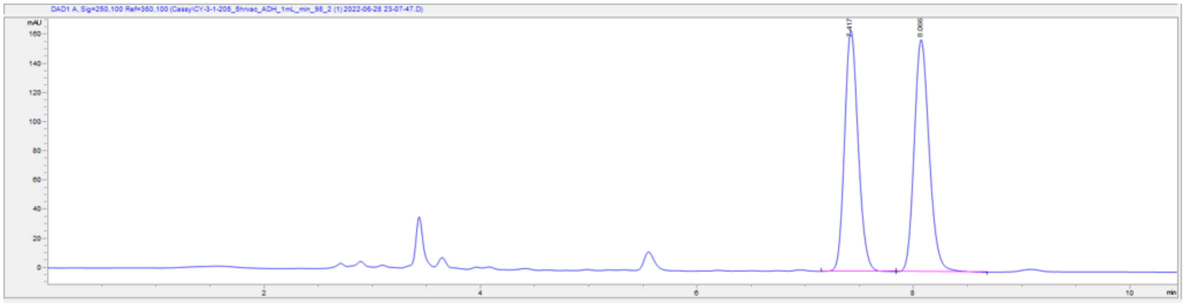

#	Time	Type	Area	Height	Width	Area%	Symmetry
1	7.417	BB	1435	165	0.1355	48.491	0.828
2	8.066	BB	1524.3	158.7	0.148	51.509	0.802

Enantioenriched Material

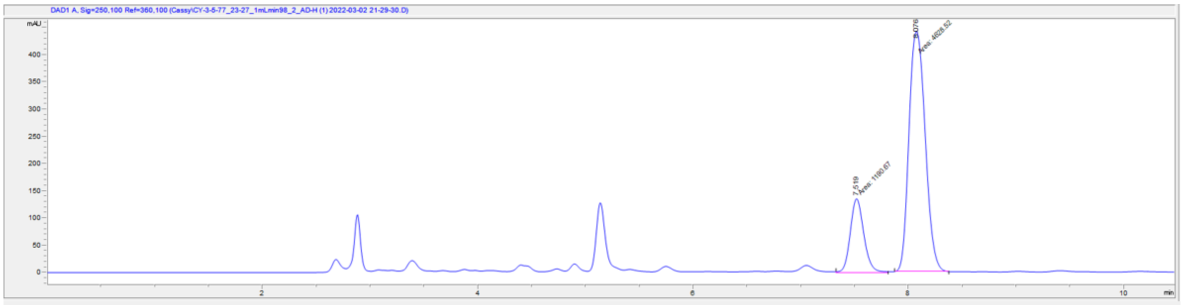

#	Time	Type	Area	Height	Width	Area%	Symmetry
1	7.519	MM	1190.7	135.8	0.1462	20.461	0.838
2	8.076	MM	4628.5	441.1	0.1749	79.539	0.822

Using (*R*,*R*)-(-)-2,3-Bis(t-butylmethylphosphino)quinoxaline ((*R*,*R*)-QuinoxP*) instead of (*R*,*R*)-BenzP*.
HPLC (CHIRALPAK® AD-H (4.6 x 250 mm), 5 mic, hexane/2-propanol 98:2, 1 mL/min) t_R 8.355 (minor), 9.207 (major), 13:87 er, 74% ee.

Racemic Material

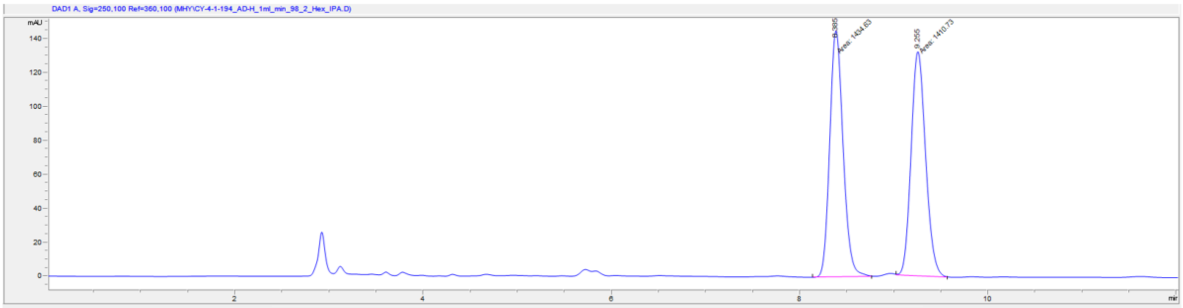

#	Time	Type	Area	Height	Width	Area%	Symmetry
1	8.385	MM	1434.6	145	0.1649	50.420	0.832
2	9.255	MM	1410.7	131.8	0.1783	49.580	0.852

Enantioenriched Material

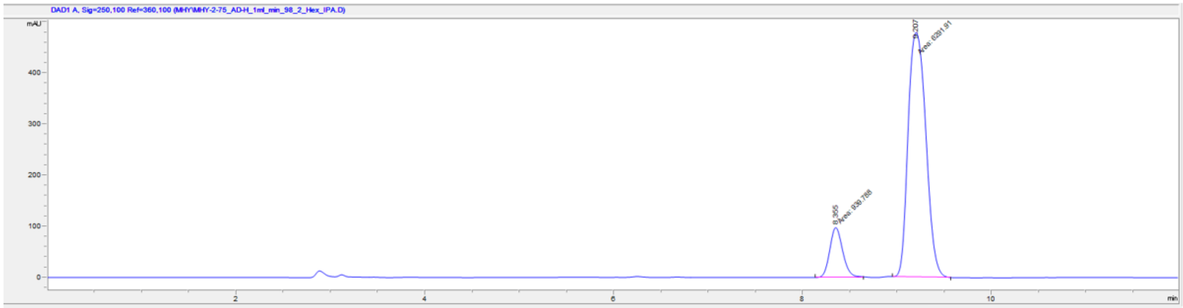

#	Time	Type	Area	Height	Width	Area%	Symmetry
1	8.355	MM	939.8	97.9	0.1601	12.995	0.84
2	9.207	MM	6291.9	479.9	0.2185	87.005	0.796

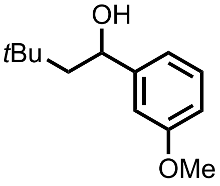

(*S*)-1-(3-methoxyphenyl)-3,3-dimethylbutan-1-ol (5):

Compound **5** was synthesized following the general procedure B, using *tert*-butyl bromide (54.8 mg, 0.4 mmol), vinylboronic acid pinacol ester (30.8 mg, 0.2 mmol), and 3-methoxyphenylmagnesium bromide (0.4 mL, 1.0 M solution in THF, 0.4 mmol), followed by the oxidation step using $NaBO_3$•$4H_2O$ (92.3 mg, 0.6 mmol), THF (2 mL), and H_2O (2 mL). The product **5** was obtained as a colorless liquid (27.2 mg, 65% yield) after purification by flash column chromatography on silica gel with gradient 0% to 20% EtOAc in hexane.

^{1}H NMR (400 MHz, $CDCl_3$): δ = 7.27 – 7.23 (m, 1H), 6.92 – 6.91 (m, 2H), 6.81 – 6.78 (m, 1H), 4.80 (dd, *J* = 8.4, 3.3 Hz, 1H), 3.82 (s, 3H), 1.78 – 1.72 (m, 2H), 1.60 (dd, *J* = 14.6, 3.4 Hz, 1H), 1.00 (s, 9H);

^{13}C NMR (100 MHz, $CDCl_3$): δ = 159.9, 148.4, 129.7, 118.2, 112.9, 111.4, 72.6, 55.4, 53.0, 30.7, 30.3;

FTIR (cm^{-1}): 3420, 2950, 1599, 1465, 1260, 1045, 766;

HRMS (APCI$^+$): calcd for $C_{13}H_{19}O$ $[M-H_2O+H]^+$ *m/z* = 191.1420; found 191.1430;

$[\alpha]_D^{23.2}$ = -39.42 (*c* = 0.550, EtOAc, *l* = 50 mm);

HPLC (CHIRALPAK® AS-H (4.6 x 250 mm), 5 mic, hexane/2-propanol 90:10, 1 mL/min) t_R 4.902 (minor), 6.052 (major), 19:81 er, 62% ee.

Racemic Material

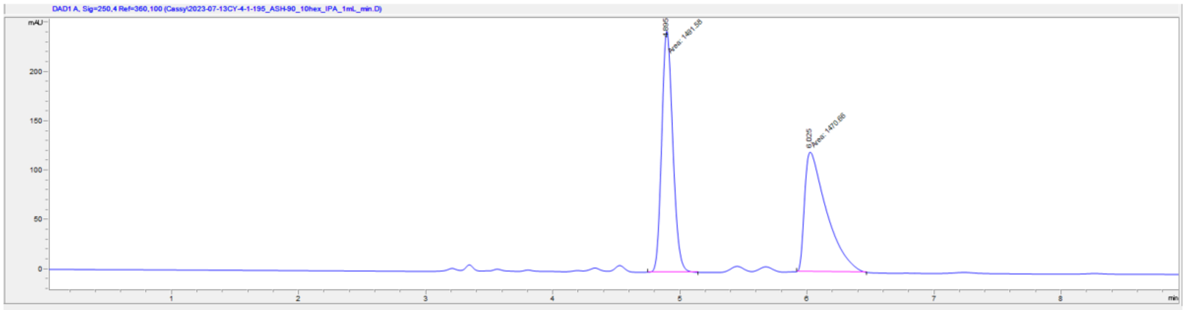

#	Time	Type	Area	Height	Width	Area%	Symmetry
1	4.895	MM	1491.6	246	0.101	50.353	0.816
2	6.025	MM	1470.7	121.8	0.2012	49.647	0.334

Enantioenriched Material

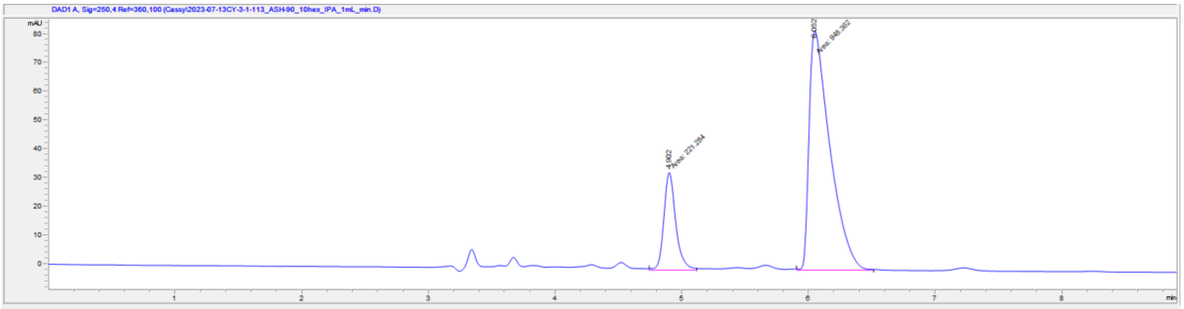

#	Time	Type	Area	Height	Width	Area%	Symmetry
1	4.902	MM	221.3	33.9	0.1088	18.919	0.8
2	6.052	MM	948.4	83	0.1904	81.081	0.371

Using (*R*,*R*)-QuinoxP* instead of (*R*,*R*)-BenzP*.
HPLC (CHIRALPAK® AS-H (4.6 x 250 mm), 5 mic, hexane/2-propanol 90:10, 1 mL/min) t_R 4.896 (minor), 5.883 (major), 14:86 er, 72% ee.

Racemic Material

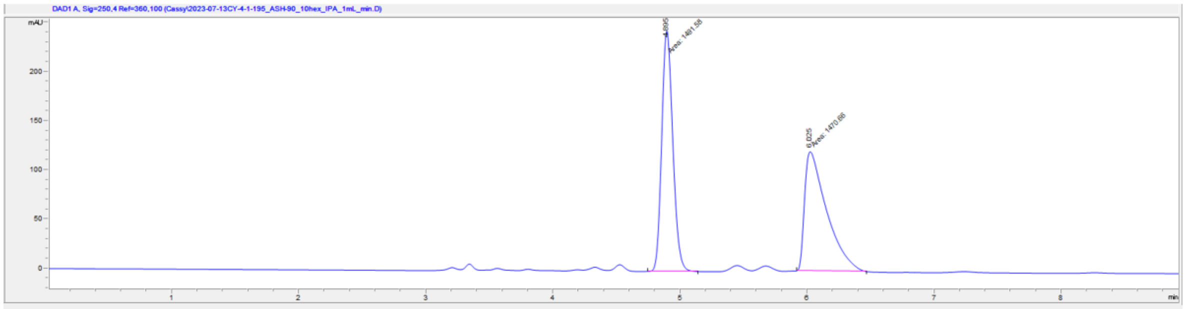

#	Time	Type	Area	Height	Width	Area%	Symmetry
1	4.895	MM	1491.6	246	0.101	50.353	0.816
2	6.025	MM	1470.7	121.8	0.2012	49.647	0.334

Enantioenriched Material

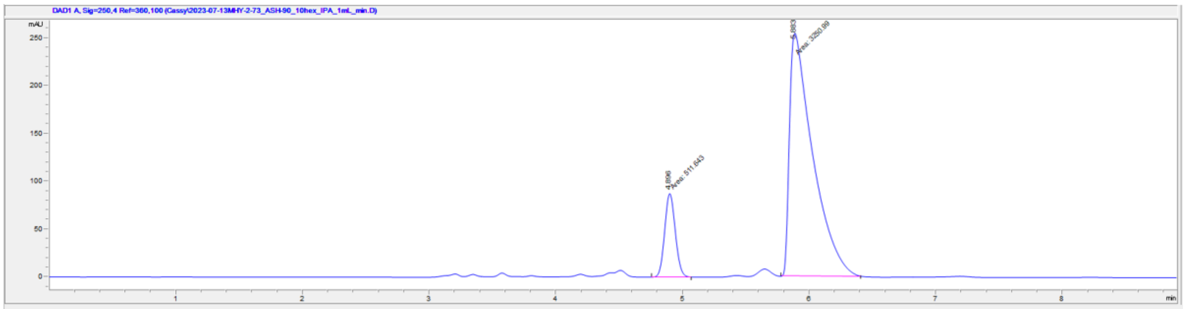

#	Time	Type	Area	Height	Width	Area%	Symmetry
1	4.896	MM	511.6	87.9	0.0971	13.598	0.896
2	5.883	MM	3251	254.8	0.2126	86.402	0.29

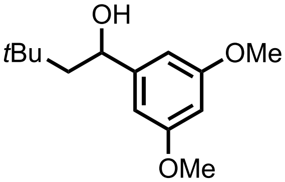

(*S*)-1-(3,5-dimethoxyphenyl)-3,3-dimethylbutan-1-ol (6):

Compound **6** was synthesized following the general procedure B, using *tert*-butyl bromide (54.8 mg, 0.4 mmol), vinylboronic acid pinacol ester (30.8 mg, 0.2 mmol), and 3,5-dimethoxyphenylmagnesium chloride (0.4 mL, 1.0 M solution in THF, 0.4 mmol), followed by the oxidation step using $NaBO_3$•$4H_2O$ (92.3 mg, 0.6 mmol), THF (2 mL), and H_2O (2 mL). The product **6** was obtained as a colorless liquid (24.2 mg, 50% yield) after purification by flash column chromatography on silica gel with gradient 0% to 20% EtOAc in hexane.

^{1}H NMR (400 MHz, $CDCl_3$): δ = 6.51 (d, *J* = 2.3 Hz, 2H), 6.36 (t, *J* = 2.3 Hz, 1H), 4.76 (dd, *J* = 8.6, 3.2 Hz, 1H), 3.80 (s, 6H), 1.73 (dd, *J* = 14.5, 8.6 Hz, 1H), 1.65 (b.s., 1H), 1.58 (dd, *J* = 14.5, 3.3 Hz, 1H), 1.00 (s, 9H);

^{13}C NMR (100 MHz, $CDCl_3$): δ = 161.1, 149.3, 103.8, 99.3, 72.8, 55.5, 53.0, 30.7, 30.3;

FTIR (cm^{-1}): 3440, 2950, 1594, 1203, 1151, 837;

HRMS (ESI^+): calcd for $C_{14}H_{23}O_3$ $[M+H]^+$ *m/z* = 239.1642; found 239.1637;

$[\alpha]_D^{23.7}$ = -25.15 (*c* = 0.450, EtOAc, *l* = 50 mm);

HPLC (CHIRALPAK® AD-H (4.6 x 250 mm), 5 mic, hexane/2-propanol 95:5, 1 mL/min) t_R 14.305 (major), 15.340 (minor), 78:22 er, 56% ee.

Racemic Material

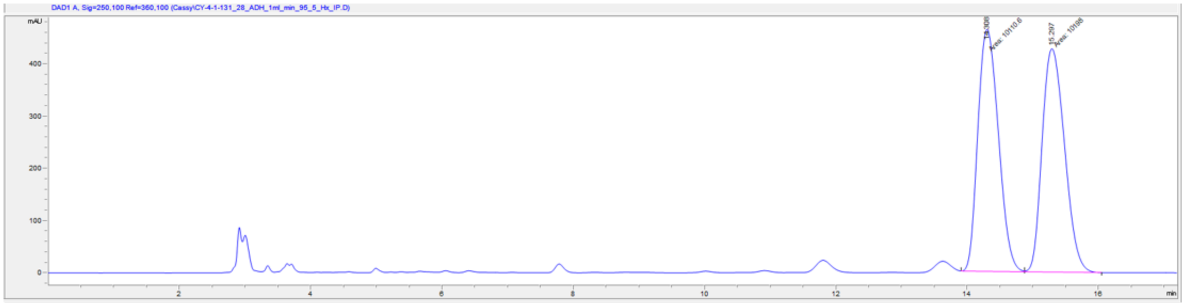

#	Time	Type	Area	Height	Width	Area%	Symmetry
1	14.308	MF	10110.6	463.2	0.3638	49.785	0.776
2	15.297	FM	10198	427.8	0.3973	50.215	0.75

Enantioenriched Material

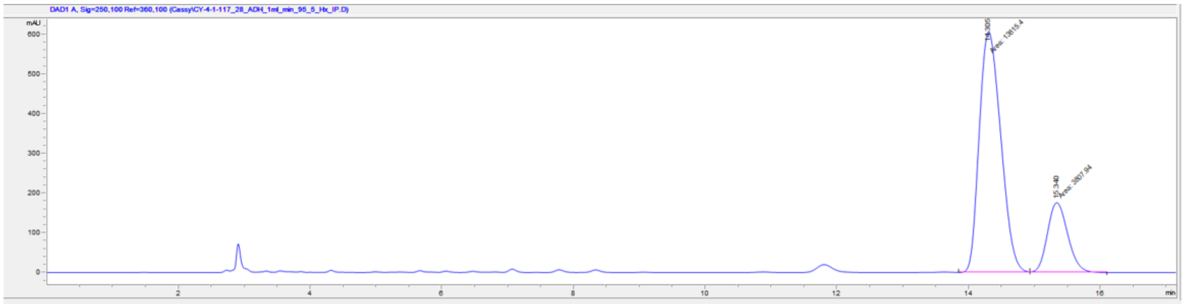

#	Time	Type	Area	Height	Width	Area%	Symmetry
1	14.305	MF	13815.4	607.4	0.3791	78.393	0.741
2	15.34	FM	3807.9	176.4	0.3598	21.607	0.818

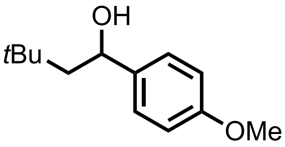

(*S*)-1-(4-methoxyphenyl)-3,3-dimethylbutan-1-ol (7):

Compound **7** was synthesized following the general procedure B, using *tert*-butyl bromide (54.8 mg, 0.4 mmol), vinylboronic acid pinacol ester (30.8 mg, 0.2 mmol), and 4-methoxyphenylmagnesium bromide solution (0.8 mL, 0.5 M solution in THF, 0.4 mmol), followed by the oxidation step using $NaBO_3$•$4H_2O$ (92.3 mg, 0.6 mmol), THF (2 mL), and H_2O (2 mL). The product **7** was obtained as a white solid (17.5 mg, 42% yield) after purification by flash column chromatography on silica gel with gradient 0% to 20% EtOAc in hexane. Spectra matched those reported previously.[10]

^{1}H NMR (400 MHz, $CDCl_3$): δ 7.28 – 7.21 (m, 2H), 6.90 – 6.80 (m, 2H), 4.76 (dd, *J* = 8.1, 4.0 Hz, 1H), 3.78 (s, 3H), 1.74 (dd, *J* = 14.4, 8.2 Hz, 1H), 1.59 – 1.55 (m, 2H), 0.95 (s, 9H);

mp: 50–52 °C;

$[\alpha]_D^{24.2}$ = -40.41 (*c* = 0.490, EtOAc, *l* = 50 mm);

HPLC (CHIRALPAK® AD-H (4.6 x 250 mm), 5 mic, hexane/2-propanol 90:10, 1 mL/min) t_R 6.027 (minor), 6.402 (major), 22:78 er, 56% ee.

Racemic Material

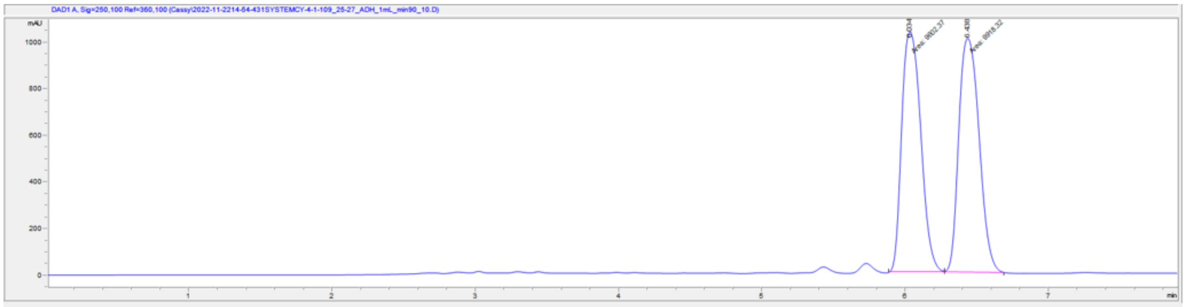

#	Time	Type	Area	Height	Width	Area%	Symmetry
1	6.034	MF	9602.4	1032.3	0.155	49.191	0.735
2	6.438	FM	9918.3	1005.5	0.1644	50.809	0.758

Enantioenriched Material

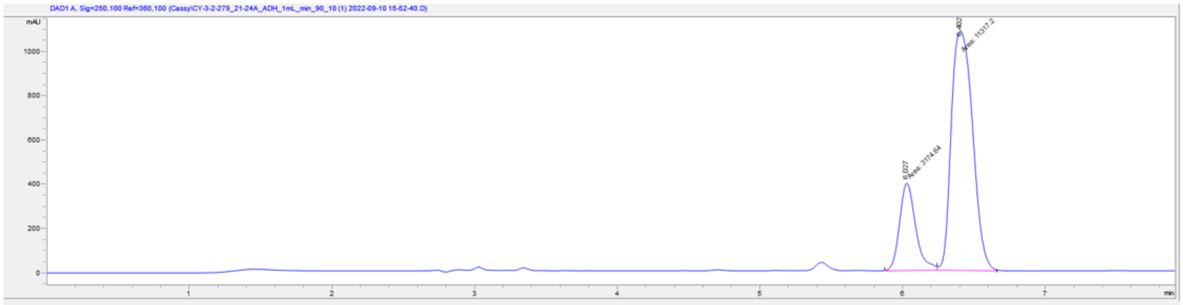

#	Time	Type	Area	Height	Width	Area%	Symmetry
1	6.027	MM	3174.6	399.1	0.1326	21.906	0.793
2	6.402	MM	11317.2	1086.6	0.1736	78.094	0.723

Using (*R*,*R*)-QuinoxP* instead of (*R*,*R*)-BenzP*.
HPLC (CHIRALPAK® AD-H (4.6 x 250 mm), 5 mic, hexane/2-propanol 90:10, 1 mL/min) t_R 6.696 (minor), 7.145 (major), 23:77 er, 54% ee.

Racemic Material

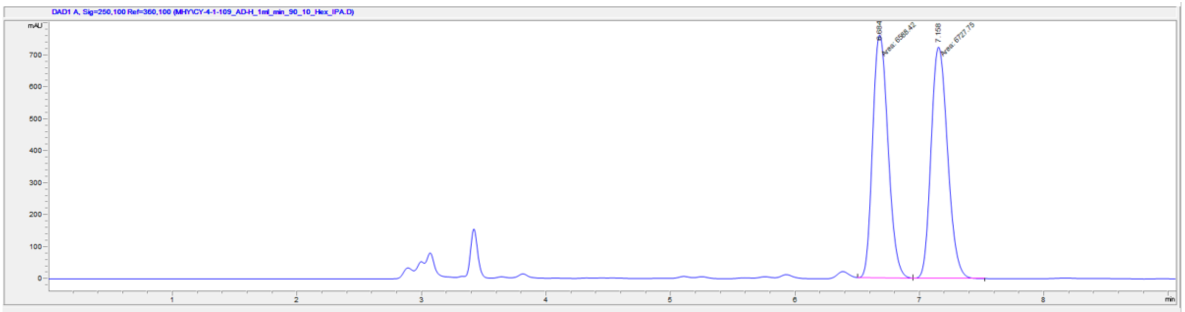

#	Time	Type	Area	Height	Width	Area%	Symmetry
1	6.684	MF	6588.4	764.2	0.1437	49.477	0.802
2	7.158	FM	6727.7	727.2	0.1542	50.523	0.802

Enantioenriched Material

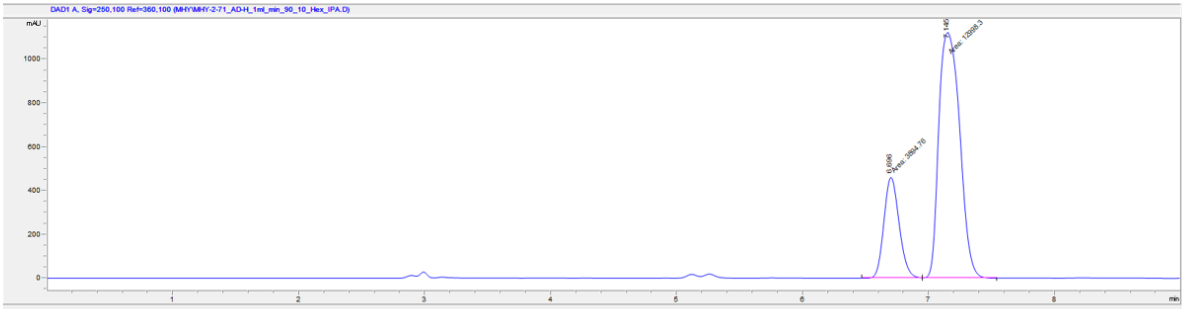

#	Time	Type	Area	Height	Width	Area%	Symmetry
1	6.696	MF	3894.8	462.1	0.1405	23.055	0.845
2	7.145	FM	12998.3	1125	0.1926	76.945	0.707

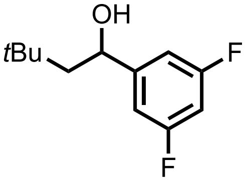

(*S*)-1-(3,5-difluorophenyl)-3,3-dimethylbutan-1-ol (8):

Compound **8** was synthesized following the general procedure B, using *tert*-butyl bromide (54.8 mg, 0.4 mmol), vinylboronic acid pinacol ester (30.8 mg, 0.2 mmol), and 3,5-difluorophenylmagnesium bromide solution (0.8 mL, 0.5 M solution in THF, 0.4 mmol), followed by the oxidation step using $NaBO_3$•$4H_2O$ (92.3 mg, 0.6 mmol), THF (2 mL), and H_2O (2 mL). The product **8** was obtained as a colorless liquid (21.4 mg, 49% yield) after purification by flash chromatography on silica gel with gradient 0% to 20% EtOAc in hexane.

^{1}H NMR (400 MHz, $CDCl_3$): δ = 6.88 – 6.85 (m, 2H), 6.71 – 6.65 (m, 1H), 4.81 (dd, *J* = 8.8 Hz, 3.2 Hz, 1H), 1.77 (b.s., 1H), 1.69 (dd, *J* = 14.8, 8.8 Hz, 1H), 1.56 – 1.52 (m, 1H), 1.01 (s, 9H);

^{13}C NMR (100 MHz, $CDCl_3$): δ = 163.3 (dd, *J* = 247.0 Hz, 12.5 Hz), 150.8 (t, *J* = 8.0 Hz), 108.6 (dd, *J* = 18.4 Hz, 6.8 Hz), 102.6 (t, *J* = 25.2 Hz), 71.8, 53.2, 30.7, 30.3;

^{19}F NMR (376 MHz, $CDCl_3$): δ = -109.62 (2F);

FTIR (cm^{-1}): 3379, 2954, 1596, 1463, 1117, 749;

HRMS (ESI^-): calcd for $C_{12}H_{16}F_2OCl$ $[M+Cl]^-$ *m/z* = 249.0852; found 249.0864;

$[\alpha]_D^{24.3}$ = -14.83 (*c* = 0.410, EtOAc, *l* = 50 mm);

HPLC (CHIRALPAK® AD-H (4.6 x 250 mm), 5 mic, hexane/2-propanol 98:2, 1 mL/min) t_R 6.484 (minor), 6.875 (major), 24:76 er, 52% ee.

Racemic Material

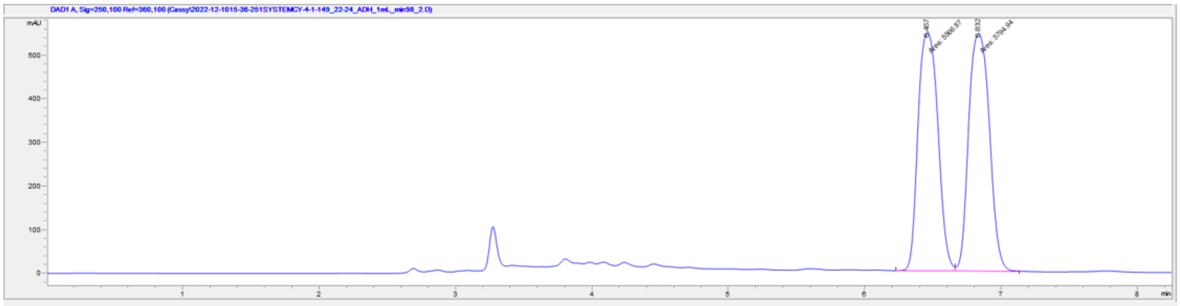

#	Time	Type	Area	Height	Width	Area%	Symmetry
1	6.457	MF	5566.9	549.9	0.1687	48.996	0.855
2	6.832	FM	5794.9	547.1	0.1765	51.004	0.871

Enantioenriched Material

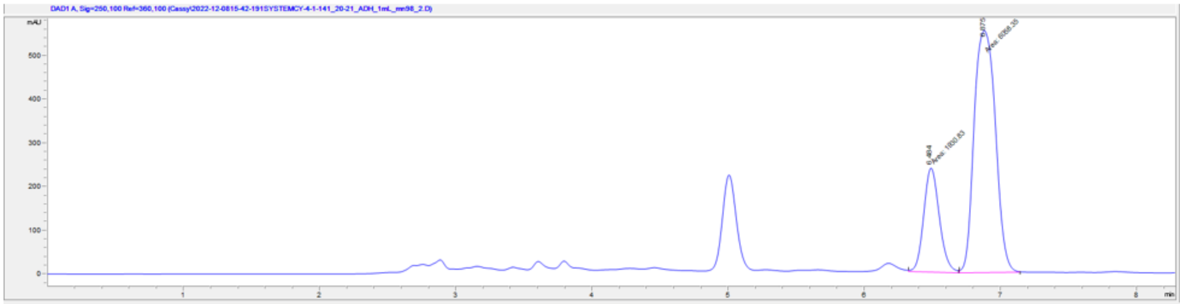

#	Time	Type	Area	Height	Width	Area%	Symmetry
1	6.484	MM	1930.8	239.3	0.1345	24.168	0.847
2	6.875	MM	6058.3	554.6	0.1821	75.832	0.877

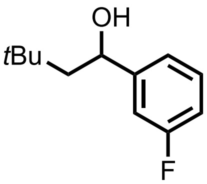

(*S*)-1-(3-fluorophenyl)-3,3-dimethylbutan-1-ol (9):

Compound **9** was synthesized following the general procedure B, using *tert*-butyl bromide (54.8 mg, 0.4 mmol), vinylboronic acid pinacol ester (30.8 mg, 0.2 mmol), and 3-fluorophenylmagnesium bromide solution (0.4 mL, 1 M solution in THF, 0.4 mmol), followed by the oxidation step using $NaBO_3{\bullet}4H_2O$ (92.3 mg, 0.6 mmol), THF (2 mL), and H_2O (2 mL). The product **9** was obtained as a colorless liquid (13.5 mg, 34% yield) after purification by flash chromatography on silica gel with gradient 0% to 40% EtOAc in hexane.

^{1}H NMR (400 MHz, $CDCl_3$): δ = 7.32 – 7.27 (m, 1H), 7.11 – 7.05 (m, 2H), 6.96 – 6.91 (m, 1H), 4.83 (dd, *J* = 8.5, 3.3 Hz, 1H), 1.75 – 1.70 (m, 2H), 1.58 (dd, *J* = 14.6, 3.3 Hz, 1H), 1.01 (s, 9H);

^{13}C NMR (100 MHz, $CDCl_3$): δ = 163.1 (d, *J* = 244.4 Hz), 149.3 (d, *J* = 6.5 Hz), 130.1 (d, *J* = 8.0 Hz), 121.4 (d, *J* = 2.8 Hz), 114.3 (d, *J* = 21.2 Hz), 112.8 (d, *J* = 21.6 Hz), 72.1 (d, *J* = 1.8 Hz), 53.2, 30.7, 30.3;

^{19}F NMR (376 MHz, $CDCl_3$): δ = -112.95;

FTIR (cm^{-1}): 3380, 2952, 1592, 1260, 749;

HRMS (ESI^+): calcd for $C_{12}H_{17}FONa$ $[M+Na]^+$ *m/z* = 219.1156; found 219.1159;

$[\alpha]_D^{23.9}$ = -14.93 (*c* = 0.730, EtOAc, *l* = 100 mm);

HPLC (CHIRALPAK® AD-H (4.6 x 250 mm), 5 mic, hexane/2-propanol 98:2, 0.5 mL/min) t_R 16.991 (minor), 17.828 (major), 20:80 er, 60% ee.

Racemic Material

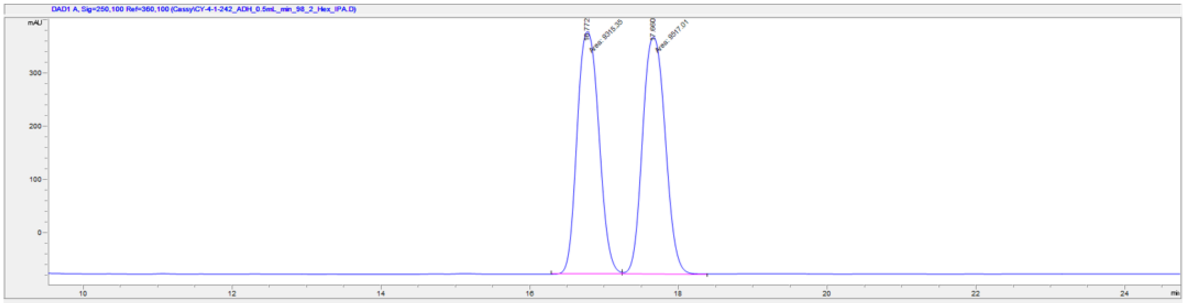

#	Time	Type	Area	Height	Width	Area%	Symmetry
1	16.772	MF	9315.3	456.7	0.3399	49.465	0.864
2	17.66	FM	9517	447.4	0.3545	50.535	0.88

Enantioenriched Material

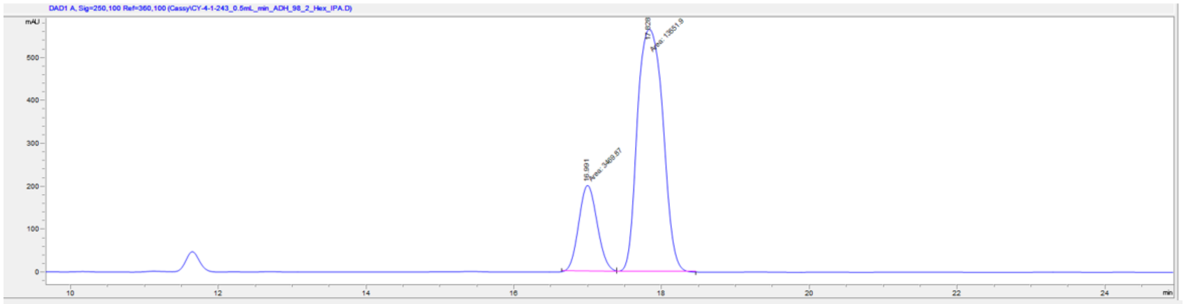

#	Time	Type	Area	Height	Width	Area%	Symmetry
1	16.991	MF	3469.9	199.8	0.2894	20.385	0.889
2	17.828	FM	13551.9	564.2	0.4003	79.615	0.857

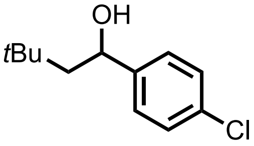

(*S*)-1-(4-chlorophenyl)-3,3-dimethylbutan-1-ol (10):

Compound **10** was synthesized following the general procedure B, using *tert*-butyl bromide (54.8 mg, 0.4 mmol), vinylboronic acid pinacol ester (30.8 mg, 0.2 mmol), and 4-chlorophenylmagnesium bromide solution (0.4 mL, 1.0 M solution in diethyl ether, 0.4 mmol), followed by the oxidation step using $NaBO_3$•$4H_2O$ (92.3 mg, 0.6 mmol), THF (2 mL), and H_2O (2 mL). The product **10** was obtained as a white solid (22.1 mg, 49% yield) after purification by flash chromatography on silica gel with gradient 0% to 20% EtOAc in hexane. Spectra matched those reported previously.[11]

^{1}H NMR (400 MHz, $CDCl_3$): δ = 7.28 – 7.22 (m, 4H), 4.78 (dd, *J* = 8.4 Hz, 3.2 Hz, 1H), 1.69 (dd, *J* = 14.8 Hz, 8.4 Hz, 1H), 1.54 – 1.50 (m, 2H), 0.96 (s, 9H);

$[\alpha]_D^{24.1}$ = -46.40 (*c* = 0.150, EtOAc, *l* = 50 mm);

HPLC (CHIRALPAK® AD-H (4.6 x 250 mm), 5 mic, hexane/2-propanol 95:5, 0.5 mL/min) t_R 14.179 (minor), 15.418 (major), 17:83 er, 66% ee.

Racemic Material

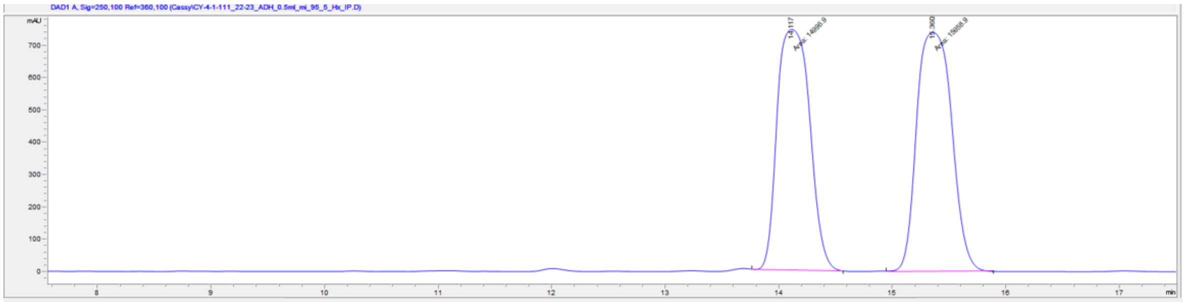

#	Time	Type	Area	Height	Width	Area%	Symmetry
1	14.117	MM	14996.9	746.6	0.3348	48.446	0.797
2	15.36	MM	15958.9	740.9	0.359	51.554	0.835

Enantioenriched Material

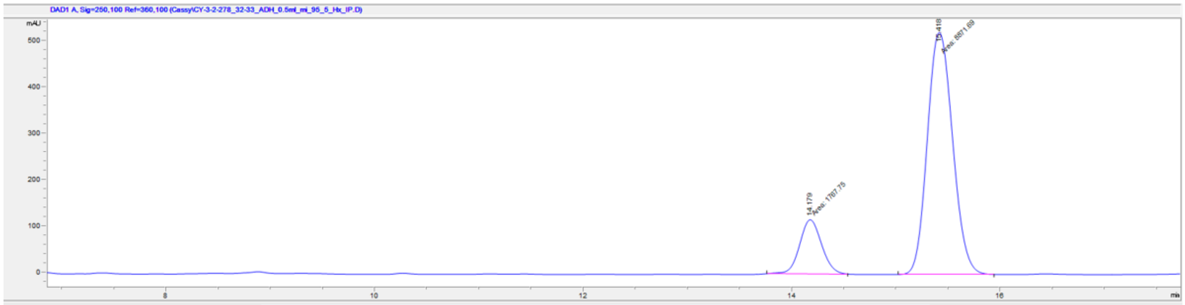

#	Time	Type	Area	Height	Width	Area%	Symmetry
1	14.179	MM	1767.8	118.3	0.249	16.615	0.909
2	15.418	MM	8871.7	524.5	0.2819	83.385	0.867

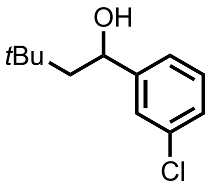

(*S*)-1-(3-chlorophenyl)-3,3-dimethylbutan-1-ol (11):

Compound **11** was synthesized following the general procedure B, using *tert*-butyl bromide (54.8 mg, 0.4 mmol), vinylboronic acid pinacol ester (30.8 mg, 0.2 mmol), and 3-chlorophenylmagnesium bromide solution (0.8 mL, 0.5 M solution in THF, 0.4 mmol), followed by the oxidation step using $NaBO_3$•$4H_2O$ (92.3 mg, 0.6 mmol), THF (2 mL), and H_2O (2 mL). The product **11** was obtained as a white solid (21.2 mg, 47% yield) after purification by flash chromatography on silica gel with gradient 0% to 20% EtOAc in hexane.

^{1}H NMR (400 MHz, $CDCl_3$): δ = 7.34 – 7.33 (m, 1H), 7.28 – 7.19 (m, 3H), 4.80 (dd, J = 8.6, 3.3 Hz, 1H), 1.74 – 1.69 (m, 2H), 1.56 (dd, J = 14.6, 3.3 Hz, 1H), 1.00 (s, 9H);

^{13}C NMR (100 MHz, $CDCl_3$): δ = 148.7, 134.5, 129.9, 127.5, 126.1, 124.0, 72.1, 53.2, 30.7, 30.3;

FTIR (cm^{-1}): 3378, 2952, 1476, 1365, 1067, 756;

HRMS (ESI$^-$): calcd for $C_{12}H_{17}Cl_2O$ $[M+Cl]^-$ m/z = 247.0651; found 247.0662;

mp: 52–54 °C;

$[\alpha]_D^{24.5}$ = -19.39 (c = 0.980, EtOAc, l = 100 mm);

HPLC (CHIRALCEL® OD-H (4.6 x 250 mm), 5 mic, hexane/2-propanol 95:5, 1 mL/min) t_R 6.088 (major), 9.547 (minor), 84:16 er, 68% ee.

Racemic Material

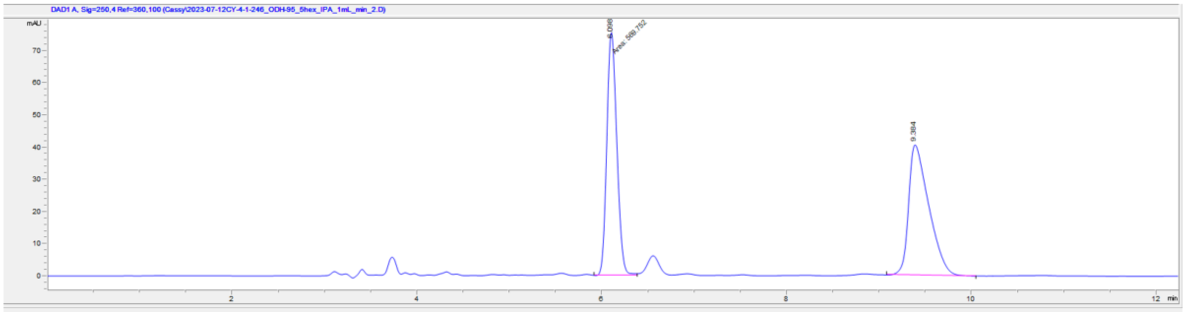

#	Time	Type	Area	Height	Width	Area%	Symmetry
1	6.098	MM	569.8	75.7	0.1254	48.042	0.8
2	9.384	BB	616.2	40.6	0.225	51.958	0.455

Enantioenriched Material

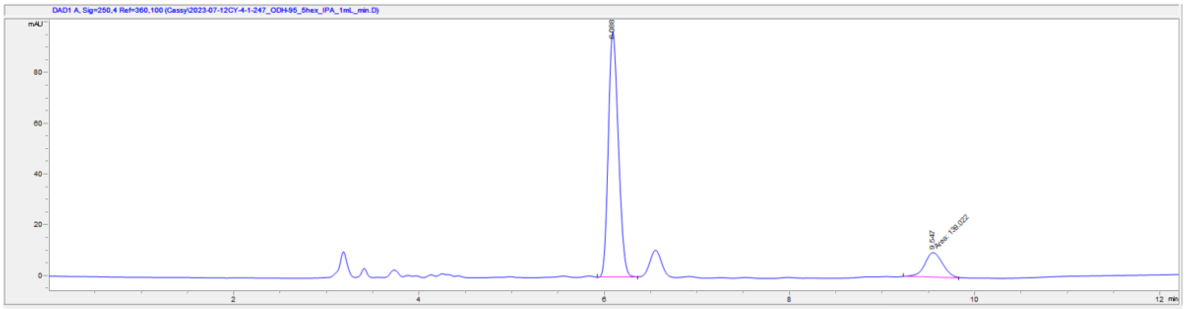

#	Time	Type	Area	Height	Width	Area%	Symmetry
1	6.088	VV	730.4	96.7	0.1182	84.009	0.75
2	9.547	MM	139	9.9	0.2339	15.991	0.875

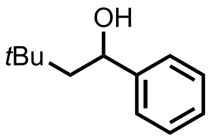

(*S*)-3,3-dimethyl-1-phenylbutan-1-ol (12):
Compound **12** was synthesized following the general procedure B, using *tert*-butyl bromide (54.8 mg, 0.4 mmol), vinylboronic acid pinacol ester (30.8 mg, 0.2 mmol), and phenylmagnesium bromide solution (0.4 mL, 1 M solution in THF, 0.4 mmol), followed by the oxidation step using $NaBO_3•4H_2O$ (92.3 mg, 0.6 mmol), THF (2 mL), and H_2O (2 mL). The product **12** was obtained as a white solid (14.9 mg, 42% yield) after purification by flash chromatography on silica gel with gradient 0% to 20% EtOAc in hexane. Spectra matched those reported previously.[12]
^{1}H NMR (400 MHz, $CDCl_3$): δ = 7.27 – 7.26 (m, 3H), 7.19 – 7.17 (m, 2H), 4.76 (dd, *J* = 8.4 Hz, 3.6 Hz, 1H), 1.68 (dd, *J* = 14.8 Hz, 8.4 Hz, 1H), 1.55 – 1.47 (m, 2H), 0.92 (s, 9H);
$[\alpha]_D^{18.9}$ = -56.0 (*c* = 0.135, EtOAc, *l* = 100 mm);
HPLC (CHIRALPAK® AS-H (4.6 x 250 mm), 5 mic, hexane/2-propanol 95:5, 1 mL/min) t_R 4.896 (minor), 5.221 (major), 14:86 er, 72% ee. (lit: $[\alpha]_D^{20}$ −71.0 (c = 1.05, THF, *l* = 100mm, ≥ 99% ee, (S)-enantiomer[12]) and $[\alpha]_D^{20}$ = -52.39 (c = 0.5, $CHCl_3$, l = 50 mm), (*S*)-enantiomer[10]).

Racemic Material

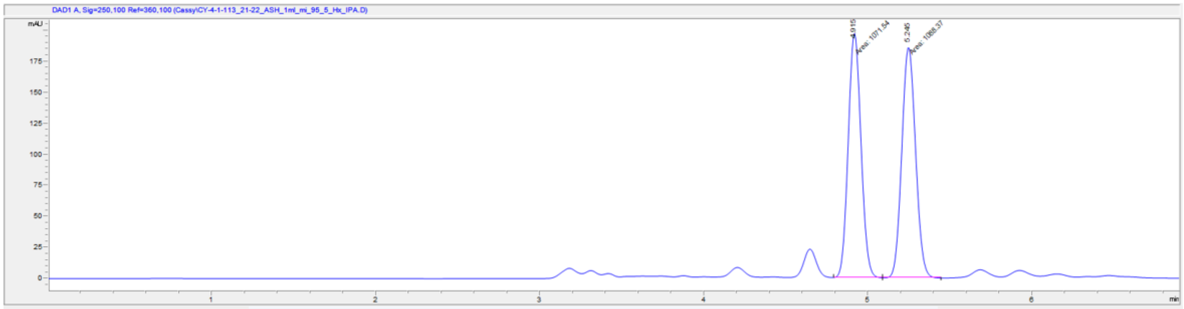

#	Time	Type	Area	Height	Width	Area%	Symmetry
1	4.915	MF	1071.5	198	0.0902	49.610	0.918
2	5.245	FM	1088.4	186.8	0.0971	50.390	0.917

Enantioenriched Material

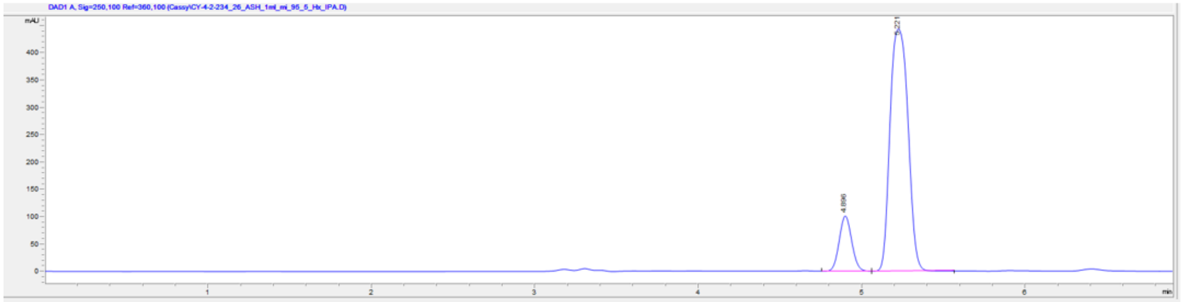

#	Time	Type	Area	Height	Width	Area%	Symmetry
1	4.896	VB	535.1	101.1	0.0817	13.641	0.915
2	5.221	BB	3387.6	443.3	0.1271	86.359	0.838

Using (*R*,*R*)-QuinoxP* instead of (*R*,*R*)-BenzP*.
HPLC (CHIRALPAK® AS-H (4.6 x 250 mm), 5 mic, hexane/2-propanol 95:5, 1 mL/min) t_R 4.891 (minor), 5.224 (major), 9:91 er, 82% ee.

Racemic Material

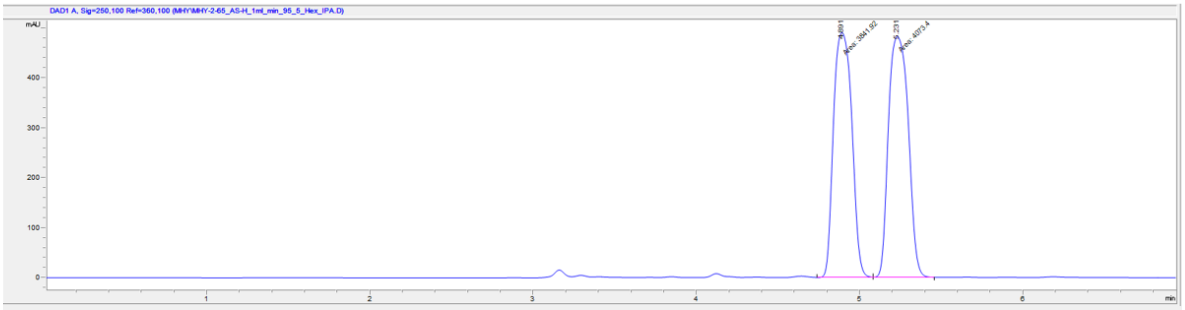

#	Time	Type	Area	Height	Width	Area%	Symmetry
1	4.891	MF	3841.9	490.2	0.1306	48.538	0.834
2	5.231	FM	4073.4	481.6	0.141	51.462	0.805

Enantioenriched Material

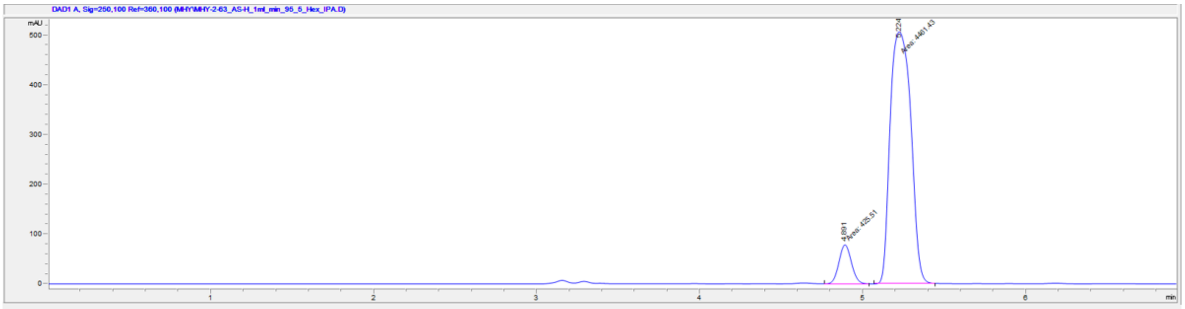

#	Time	Type	Area	Height	Width	Area%	Symmetry
1	4.891	MM	425.5	79.2	0.0896	8.707	0.916
2	5.224	MM	4461.4	506.5	0.1468	91.293	0.774

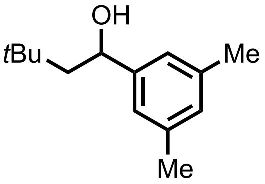

(*S*)-1-(3,5-dimethylphenyl)-3,3-dimethylbutan-1-ol (13):

Compound **13** was synthesized following the general procedure B, using *tert*-butyl bromide (54.8 mg, 0.4 mmol), vinylboronic acid pinacol ester (30.8 mg, 0.2 mmol), and 3,5-dimethylphenylmagnesium bromide solution (0.8 mL, 0.5 M solution in THF, 0.4 mmol), followed by the oxidation step using $NaBO_3\bullet 4H_2O$ (92.3 mg, 0.6 mmol), THF (2 mL), and H_2O (2 mL). The product **13** was obtained as a white solid (20.9 mg, 50% yield) after purification by flash chromatography on silica gel with gradient 0% to 20% EtOAc in hexane. Spectra matched those reported previously.[13]

^{1}H NMR (400 MHz, $CDCl_3$): δ = 6.96 (s, 2H), 6.91 (s, 1H), 4.78 – 4.75 (m, 1H), 2.32 (s, 6H), 1.75 (dd, *J* = 14.5, 8.6 Hz, 1H), 1.68 – 1.55 (m, 2H), 1.01 (s, 9H);

^{13}C NMR (100 MHz, $CDCl_3$): δ = 146.7, 138.2, 129.1, 123.6, 72.7, 53.0, 30.7, 30.3, 21.5;

FTIR (cm^{-1}): 3398, 2952, 2157, 2015, 1068;

HRMS (APCI$^+$): calcd for $C_{14}H_{21}$ $[M-H_2O+H]^+$ *m/z* = 189.1634; found 189.1638;

mp: 54–56 °C;

$[\alpha]_D^{22.5}$ = -2.84 (*c* = 0.570, EtOAc, *l* = 100 mm);

HPLC (CHIRALPAK® AD-H (4.6 x 250 mm), 5 mic, hexane/2-propanol 95:5, 0.5 mL/min) t_R 12.567 (major), 13.445 (minor), 66:33 er, 33% ee.

Racemic Material

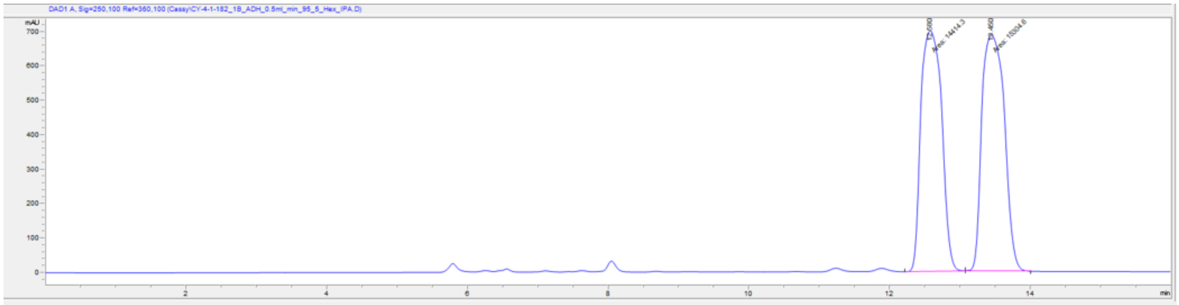

#	Time	Type	Area	Height	Width	Area%	Symmetry
1	12.58	MF	14414.3	699.1	0.3436	48.502	0
2	13.45	FM	15304.6	686	0.3719	51.498	0.724

Enantioenriched Material

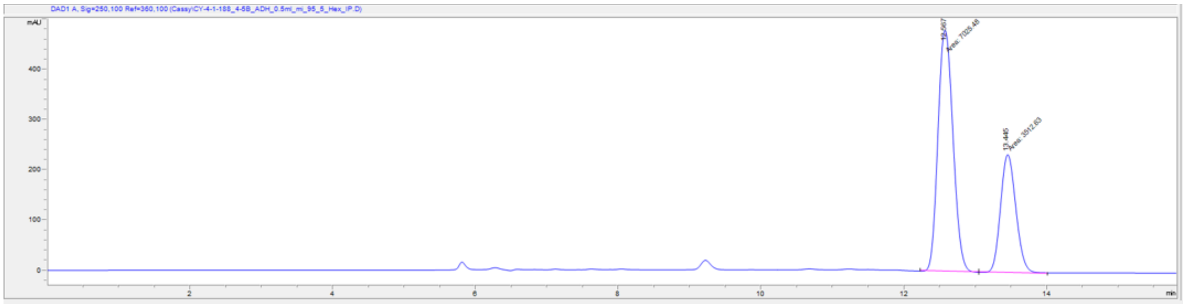

#	Time	Type	Area	Height	Width	Area%	Symmetry
1	12.567	MF	7025.5	479.3	0.2443	66.667	0.857
2	13.445	FM	3512.6	234.6	0.2495	33.333	0.847

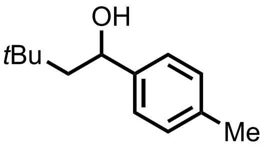

(*S*)-3,3-dimethyl-1-(p-tolyl)butan-1-ol (14):

Compound **14** was synthesized following the general procedure B, using *tert*-butyl bromide (54.8 mg, 0.4 mmol), vinylboronic acid pinacol ester (30.8 mg, 0.2 mmol), and *p*-tolylmagnesium bromide solution (0.4 mL, 1.0 M solution in THF, 0.4 mmol), followed by the oxidation step using $NaBO_3 \bullet 4H_2O$ (92.3 mg, 0.6 mmol), THF (2 mL), and H_2O (2 mL). The product **14** was obtained as a white solid (6.7 mg, 17% yield) after purification by flash chromatography on silica gel with gradient 0% to 20% EtOAc in hexane.

^{1}H NMR (400 MHz, $CDCl_3$): δ = 7.24 (d, *J* = 7.7 Hz, 2H), 7.15 (d, *J* = 7.7 Hz, 2H), 4.82 – 4.78 (m, 1H), 2.34 (s, 3H), 1.76 (dd, *J* = 14.4, 8.3 Hz, 1H), 1.64 – 1.57 (m, 2H), 0.99 (s, 9H);

^{13}C NMR (100 MHz, $CDCl_3$): δ = 143.7, 137.2, 129.3, 125.9, 72.5, 53.0, 30.6, 30.3, 21.2;

FTIR (cm^{-1}): 3385, 2950, 1513, 1475, 1363, 817;

HRMS (APCI$^+$): calcd for $C_{13}H_{19}$ $[M-H_2O+H]^+$ *m/z* = 175.1478; found 175.1481;

mp: 48–50 °C;

$[\alpha]_D^{24.7}$ = -11.40 (*c* = 0.760, EtOAc, *l* = 100 mm);

HPLC (CHIRALPAK® AD-H (4.6 x 250 mm), 5 mic, hexane/2-propanol 95:5, 1 mL/min) t_R 7.118 (minor), 7.685 (major), 37:63 er, 26% ee.

Racemic Material

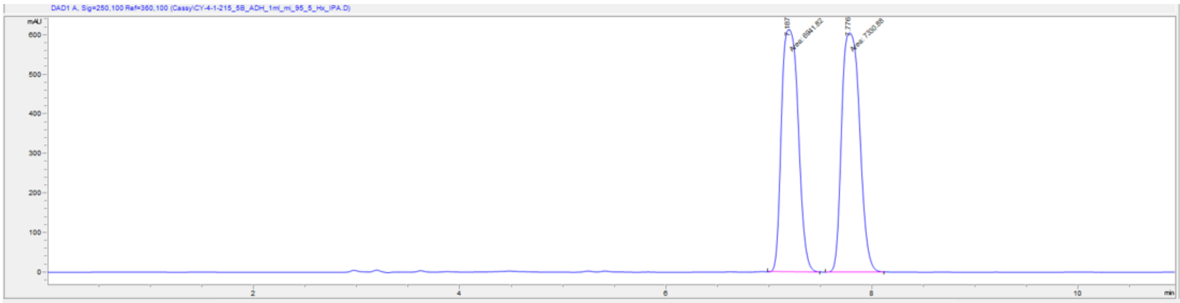

#	Time	Type	Area	Height	Width	Area%	Symmetry
1	7.187	MM	6941.8	612.5	0.1889	48.637	0.769
2	7.776	MM	7330.9	603.7	0.2024	51.363	0.765

Enantioenriched Material

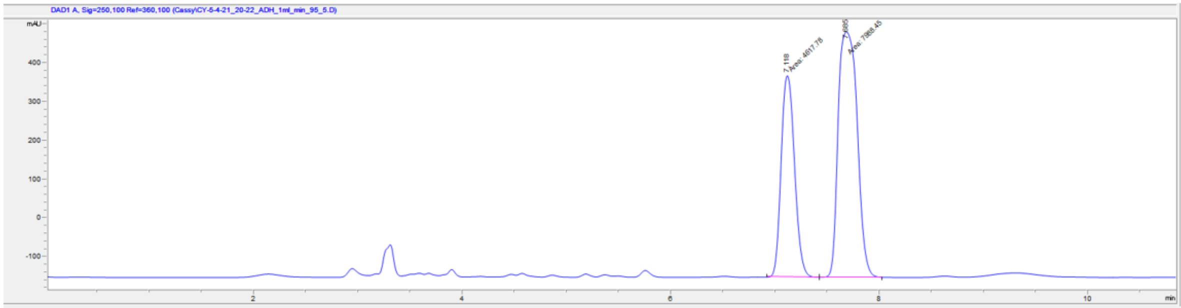

#	Time	Type	Area	Height	Width	Area%	Symmetry
1	7.118	MF	4617.8	518.2	0.1485	36.631	0.835
2	7.685	FM	7988.5	631.2	0.2109	63.369	0.747

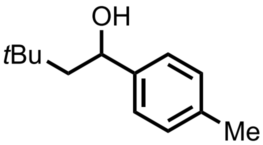

(*S*)-3,3-dimethyl-1-(p-tolyl)butan-1-ol (14'):

Compound **14'** was synthesized following the general procedure B, using *tert*-butyl iodide (73.6 mg, 0.4 mmol), vinylboronic acid pinacol ester (30.8 mg, 0.2 mmol), and *p*-tolylmagnesium bromide solution (0.4 mL, 1.0 M solution in THF, 0.4 mmol), followed by the oxidation step using $NaBO_3$•$4H_2O$ (92.3 mg, 0.6 mmol), THF (2 mL), and H_2O (2 mL). The product **14'** was obtained as a white solid (23.3mg, 61% yield) after purification by flash chromatography on silica gel with gradient 0% to 20% EtOAc in hexane.

Spectra matched **14.**

$[\alpha]_D^{22.7}$ = -12.37 (*c* = 0.760, EtOAc, *l* = 100 mm);

HPLC (CHIRALPAK® AD-H (4.6 x 250 mm), 5 mic, hexane/2-propanol 95:5, 1 mL/min) t_R 7.177 (minor), 7.757 (major), 39:61 er, 22% ee.

Racemic Material

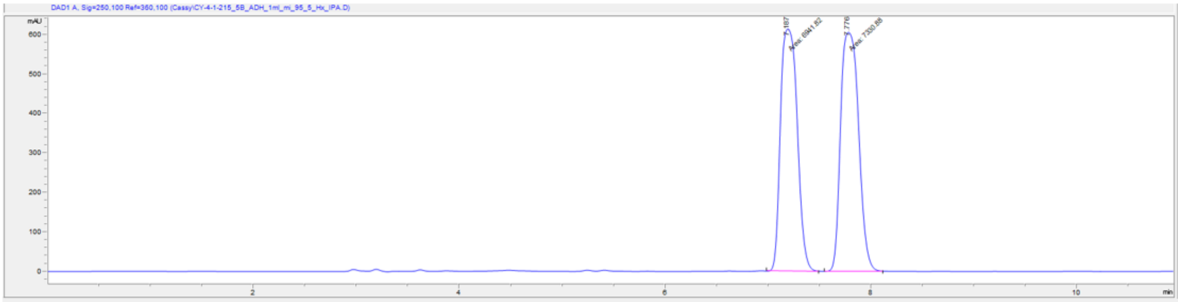

#	Time	Type	Area	Height	Width	Area%	Symmetry
1	7.187	MM	6941.8	612.5	0.1889	48.637	0.769
2	7.776	MM	7330.9	603.7	0.2024	51.363	0.765

Enantioenriched Material

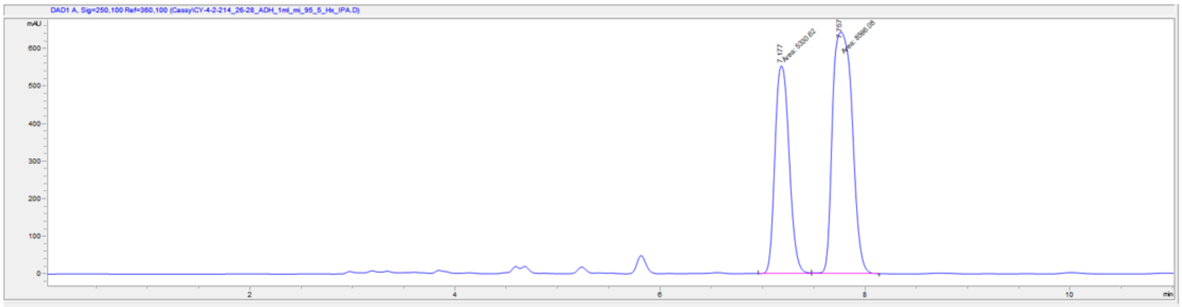

#	Time	Type	Area	Height	Width	Area%	Symmetry
1	7.177	MF	5330.6	553.2	0.1606	38.276	0.807
2	7.757	FM	8596.1	643.7	0.2226	61.724	0.727

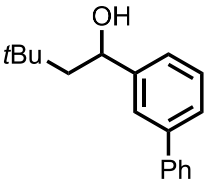

(*S*)-1-([1,1'-biphenyl]-3-yl)-3,3-dimethylbutan-1-ol (15):

Compound **15** was synthesized following the general procedure B, using *tert*-butyl bromide (54.8 mg, 0.4 mmol), vinylboronic acid pinacol ester (30.8 mg, 0.2 mmol), and 3-biphenylmagnesium bromide solution (0.8 mL, 0.5 M solution in THF, 0.4 mmol), followed by the oxidation step using $NaBO_3$•$4H_2O$ (92.3 mg, 0.6 mmol), THF (2 mL), and H_2O (2 mL). The product **15** was obtained as a white solid (29.5 mg, 58% yield) after purification by flash chromatography on silica gel with gradient 0% to 20% EtOAc in hexane.

^{1}H NMR (400 MHz, $CDCl_3$): δ = 7.52 – 7.48 (m, 3H), 7.39 – 7.16 (m, 6H), 4.83 – 4.80 (m, 1H), 1.76 – 1.65 (m, 2H), 1.56 (dd, *J* = 14.5, 3.4 Hz, 1H), 0.93 (s, 9H);

^{13}C NMR (100 MHz, $CDCl_3$): δ = 147.1, 141.5, 141.2, 129.0, 128.8, 127.4, 127.2, 126.2, 124.7, 124.7, 72.6, 53.0, 30.6, 30.2;

FTIR (cm^{-1}): 3396, 2950, 1477, 1363, 753;

HRMS (ESI^+): calcd for $C_{18}H_{22}ONa$ $[M+Na]^+$ *m/z* = 277.1563; found 277.1567;

mp: 58–60 °C;

$[\alpha]_D^{23.1}$ = -14.11 (*c* = 0.720, EtOAc, *l* = 100 mm);

HPLC (CHIRALCEL® OJ-H (4.6 x 250 mm), 5 mic, hexane/2-propanol 95:5, 1 mL/min) t_R 8.006 (major), 10.166 (minor), 83:17 er, 66% ee.

Racemic Material

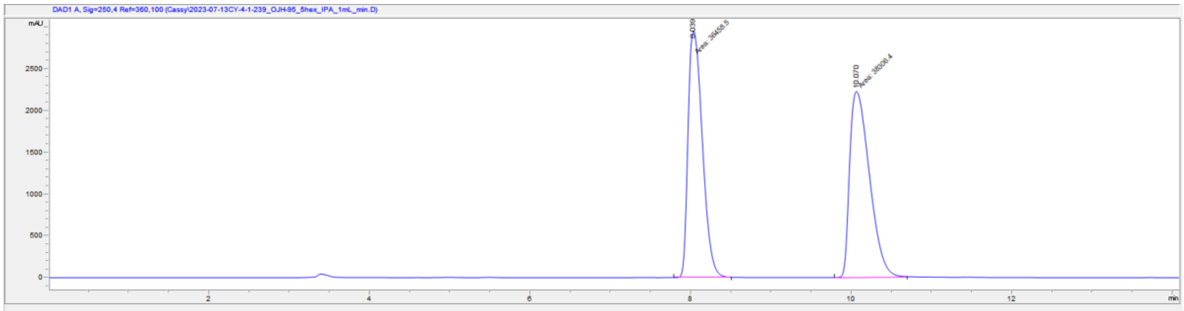

#	Time	Type	Area	Height	Width	Area%	Symmetry
1	8.039	MM	36458.5	2941.2	0.2066	48.764	0.595
2	10.07	MM	38306.4	2228	0.2865	51.236	0.509

Enantioenriched Material

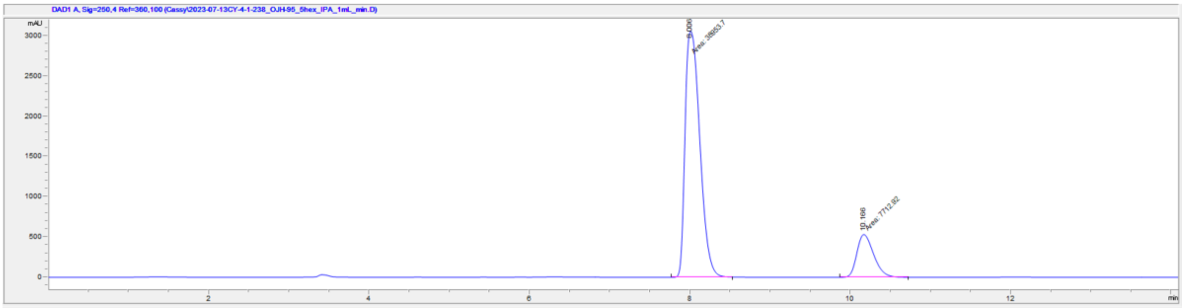

#	Time	Type	Area	Height	Width	Area%	Symmetry
1	8.006	MM	38953.7	3048.1	0.213	83.472	0.585
2	10.166	MM	7712.9	529.6	0.2427	16.528	0.69

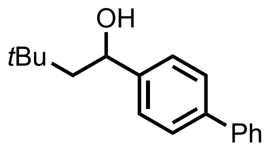

(*S*)-1-([1,1'-biphenyl]-4-yl)-3,3-dimethylbutan-1-ol (16):
Compound **16** was synthesized following the general procedure B, using *tert*-butyl bromide (54.8 mg, 0.4 mmol), vinylboronic acid pinacol ester (30.8 mg, 0.2 mmol), and 4-biphenylmagnesium bromide solution (0.8 mL, 0.5 M solution in THF, 0.4 mmol), followed by the oxidation step using $NaBO_3$•$4H_2O$ (92.3 mg, 0.6 mmol), THF (2 mL), and H_2O (2 mL). The product **16** was obtained as a white solid (35.4 mg, 69% yield) after purification by flash chromatography on silica gel with gradient 0% to 20% EtOAc in hexane. Spectra matched those reported previously.[14]
^{1}H NMR (400 MHz, $CDCl_3$): δ = 7.61 – 7.57 (m, 4H), 7.46 – 7.42 (m, 4H), 7.37 – 7.33 (m, 1H), 4.89 (dd, *J* = 8.4 Hz, 3.6 Hz, 1H), 1.81 (dd, *J* = 14.4 Hz, 8.4 Hz, 1H), 1.70 (b.s., 1H), 1.66 (dd, *J* = 14.8 Hz, 3.6 Hz, 1H), 1.03 (s, 9H);
$[\alpha]_D^{23.3}$ = -36.14 (*c* = 0.560, EtOAc, *l* = 50 mm);
HPLC (CHIRALPAK® AD-H (4.6 x 250 mm), 5 mic, hexane/2-propanol 98:2, 1 mL/min) t_R 14.310 (major), 16.204 (minor), 84:16, 68% ee.

Racemic Material

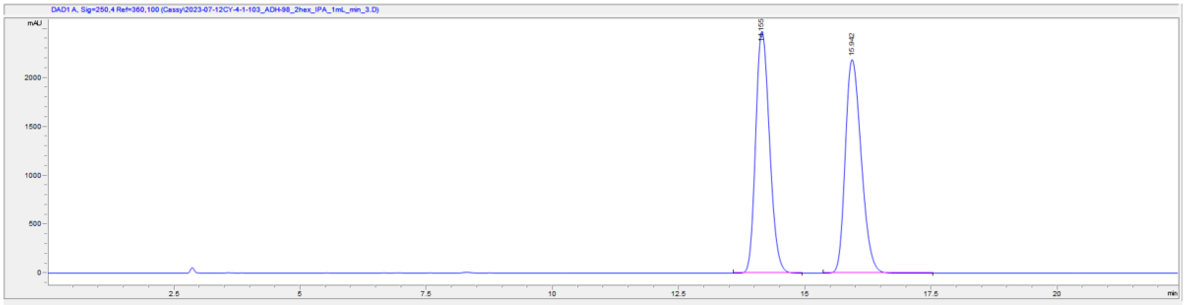

#	Time	Type	Area	Height	Width	Area%	Symmetry
1	14.155	BB	47364.7	2472.1	0.2995	49.717	0.786
2	15.942	BB	47903.5	2182.8	0.34	50.283	0.712

Enantioenriched Material

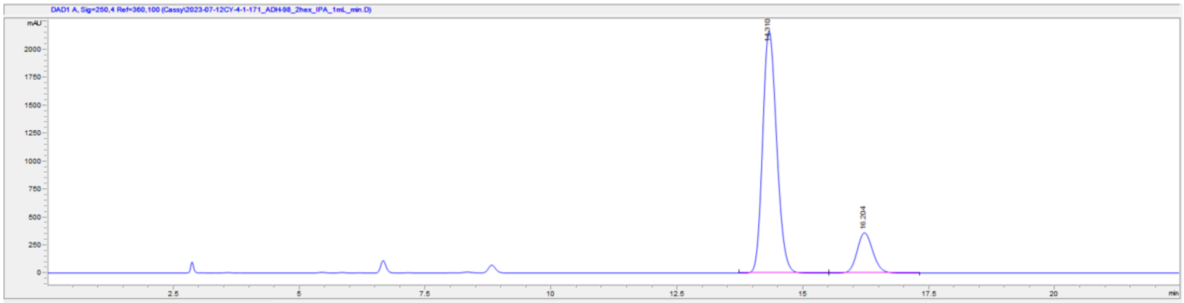

#	Time	Type	Area	Height	Width	Area%	Symmetry
1	14.31	BB	41700.6	2161.4	0.3011	84.247	0.793
2	16.204	BB	7797.3	359.5	0.335	15.753	0.854

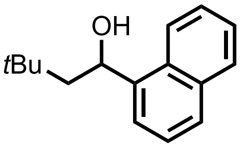

(*S*)-3,3-dimethyl-1-(naphthalen-1-yl)butan-1-ol (17):
Compound **17** was synthesized following the general procedure B, using *tert*-butyl bromide (54.8 mg, 0.4 mmol), vinylboronic acid pinacol ester (30.8 mg, 0.2 mmol), and 1-naphthylmagnesium bromide solution (1.6 mL, 0.25 M solution in THF, 0.4 mmol), followed by the oxidation step using $NaBO_3$•$4H_2O$ (92.3 mg, 0.6 mmol), THF (2 mL), and H_2O (2 mL). The product **17** was obtained as a yellow oil (27.8 mg, 61% yield) after purification by flash chromatography on silica gel with gradient 0% to 20% EtOAc in hexane.
^{1}H NMR (400 MHz, $CDCl_3$): δ = 8.13 (d, *J* = 8.5 Hz, 1H), 7.91 – 7.84 (m, 1H), 7.77 (d, *J* = 8.1 Hz, 1H), 7.67 (d, *J* = 7.1 Hz, 1H), 7.54 – 7.44 (m, 3H), 5.65 (dd, *J* = 8.7, 2.8 Hz, 1H), 1.85 (dd, *J* = 14.8, 8.7 Hz, 1H), 1.78 (dd, *J* = 14.8, 2.8 Hz, 1H), 1.68 (b.s., 1H), 1.11 (s, 9H);
^{13}C NMR (100 MHz, $CDCl_3$): δ = 142.4, 134.0, 130.1, 129.1, 127.9, 126.1, 125.7, 125.6, 123.3, 123.0, 69.5, 52.3, 31.2, 30.4;
FTIR (cm^{-1}): 3387, 2950, 1363, 1260, 1073, 768;
HRMS (APCI$^+$): calcd for $C_{16}H_{19}$ $[M-H_2O+H]^+$ *m/z* = 211.1468; found 211.1481;
$[\alpha]_D^{23.0}$ = -36.57 (*c* = 1.00, EtOAc, *l* = 100 mm);
HPLC (CHIRALPAK® AD-H (4.6 x 250 mm), 5 mic, hexane/2-propanol 98:2, 1 mL/min) t_R 11.067 (major), 13.339 (minor), 81:19 er, 62% ee.

Racemic Material

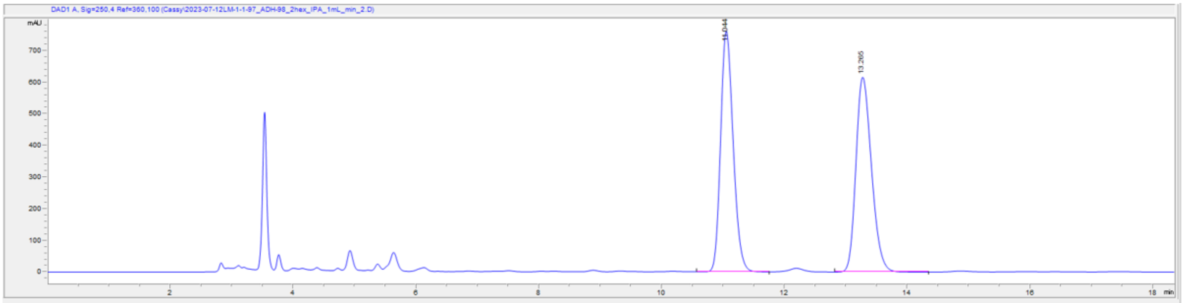

#	Time	Type	Area	Height	Width	Area%	Symmetry
1	11.044	BB	10793	760.2	0.2196	49.997	0.775
2	13.265	BB	10794.3	615.4	0.2726	50.003	0.737

Enantioenriched Material

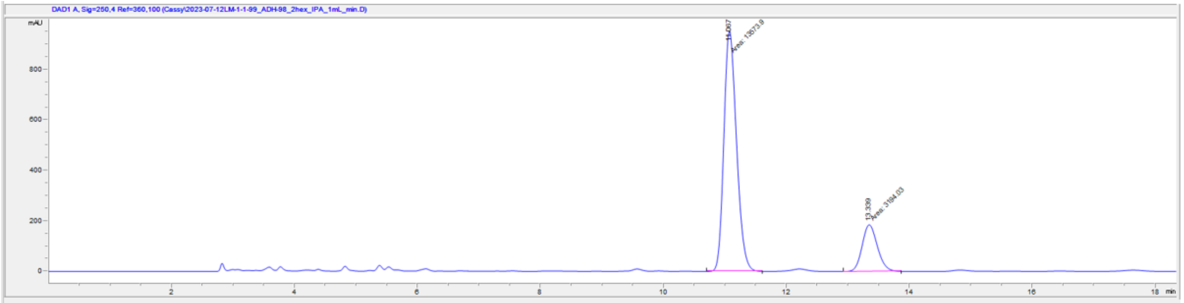

#	Time	Type	Area	Height	Width	Area%	Symmetry
1	11.067	MM	13573.9	951.4	0.2378	80.952	0.77
2	13.339	MM	3194	184.7	0.2883	19.048	0.84

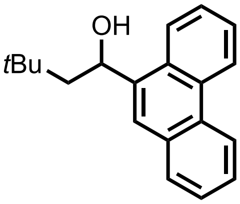

(*S*)-3,3-dimethyl-1-(phenanthren-9-yl)butan-1-ol (18):

Compound **18** was synthesized following the general procedure B, using *tert*-butyl bromide (54.8 mg, 0.4 mmol), vinylboronic acid pinacol ester (30.8 mg, 0.2 mmol), and 9-phenanthrylmagnesium bromide solution (0.8 mL, 0.5 M solution in THF, 0.4 mmol), followed by the oxidation step using $NaBO_3{\bullet}4H_2O$ (92.3 mg, 0.6 mmol), THF (2 mL), and H_2O (2 mL). The product **18** was obtained as a white solid (33.5 mg, 60% yield) after purification by flash chromatography on silica gel with gradient 0% to 20% EtOAc in hexane. Absolute stereochemistry was determined from the X-ray crystal structure of the alcohol product.

^{1}H NMR (400 MHz, $CDCl_3$): δ = 8.76 – 8.74 (m, 1H), 8.66 (d, *J* = 7.9 Hz, 1H), 8.16 (dd, *J* = 8.0, 1.6 Hz, 1H), 7.94 (s, 1H), 7.89 (dd, *J* = 7.5, 1.8 Hz, 1H), 7.69 – 7.54 (m, 4H), 5.65 (dt, *J* = 7.2, 3.6 Hz, 1H), 1.90 – 1.89 (m, 1H), 1.87 – 1.84 (m, 2H), 1.15 (s, 9H);

^{13}C NMR (100 MHz, $CDCl_3$): δ = 140.5, 131.7, 131.0, 130.1, 129.5, 128.8, 126.9, 126.7, 126.7, 126.4, 124.0, 123.7, 123.6, 122.6, 69.6, 52.0, 31.2, 30.4;

FTIR (cm^{-1}): 3426, 2949, 1363, 1246, 1075, 744;

HRMS (ESI$^+$): calcd for $C_{20}H_{22}ONa$ $[M+Na]^+$ *m/z* = 301.1563; found 301.1563;

mp: 124–126 °C;
$[\alpha]_D^{23.0}$ = -31.79 (c = 0.855, EtOAc, l = 100 mm);
HPLC (CHIRALPAK® IC (4.6 x 250 mm), 5 mic, hexane/2-propanol 90:10, 1 mL/min) t_R 5.702 (major), 7.046 (minor), 81:19 er, 62% ee.

Racemic Material

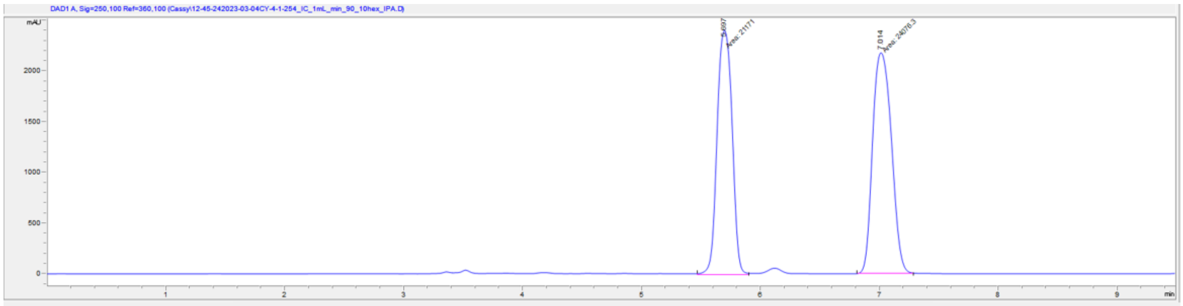

#	Time	Type	Area	Height	Width	Area%	Symmetry
1	5.697	MM	21171	2409.6	0.1464	46.790	0.932
2	7.014	MM	24076.3	2181.3	0.184	53.210	0.786

Enantioenriched Material

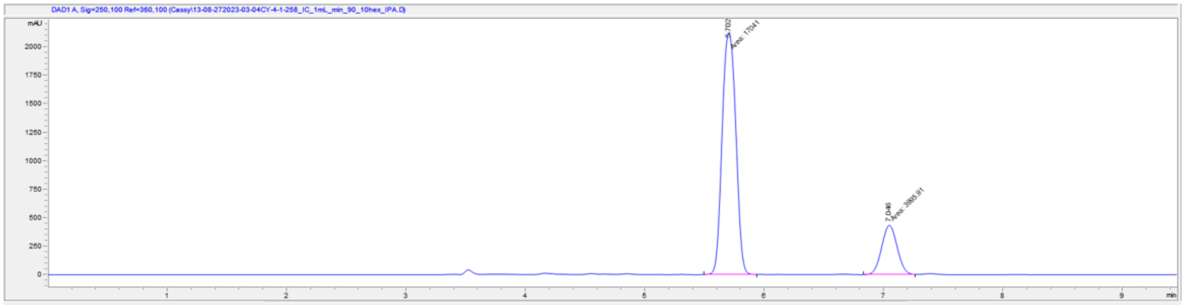

#	Time	Type	Area	Height	Width	Area%	Symmetry
1	5.702	MM	17041	2135.7	0.133	81.005	0.927
2	7.046	MM	3995.9	436.8	0.1525	18.995	0.943

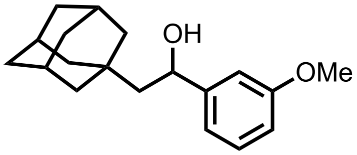

(*S*)-2-((3R,5R,7R)-adamantan-1-yl)-1-(3-methoxyphenyl)ethan-1-ol (19):
Compound **19** was synthesized following the general procedure B, using 1-Iodoadamantane (104.8 mg, 0.4 mmol), vinylboronic acid pinacol ester (30.8 mg, 0.2 mmol), and 3-methoxyphenylmagnesium bromide solution (0.4 mL, 1.0 M solution in THF, 0.4 mmol), followed by the oxidation step using $NaBO_3{\bullet}4H_2O$ (92.3 mg, 0.6 mmol), THF (2 mL), and H_2O (2 mL). The product **19** was obtained as a viscous yellow oil (30.6 mg, 53% yield) after purification by flash chromatography on silica gel with gradient 0% to 20% EtOAc in hexane.

(*S*)-2-((3R,5R,7R)-adamantan-1-yl)-1-(3-methoxyphenyl)ethan-1-ol (19) (gram scale):
Compound **19** (gram scale) was synthesized following the general procedure B, using 1-Iodoadamantane (3.4 g, 13 mmol), vinylboronic acid pinacol ester (1 g, 6.5 mmol), and 3-

methoxyphenylmagnesium bromide solution (13.0 mL, 1.0 M solution in THF, 13.0 mmol), followed by the oxidation step using $NaBO_3 \cdot 4H_2O$ (2.99 g, 19.4 mmol), THF (65.0 mL), and H_2O (65.0 mL). The product **19** was obtained as a viscous yellow oil (1.008 g, 54% yield) after purification by flash chromatography on silica gel with gradient 0% to 20% EtOAc in hexane.

^{1}H NMR (400 MHz, $CDCl_3$): δ = 7.27 – 7.23 (m, 1H), 6.92 – 6.90 (m, 2H), 6.81 – 6.78 (m, 1H), 4.87 (dd, J = 9.6 Hz, 2.4 Hz, 1H), 3.82 (s, 3H), 1.97 (s, 3H), 1.73 – 1.58 (m, 15H);
^{13}C NMR (100 MHz, $CDCl_3$): δ = 159.8, 148.4, 129.5, 118.0, 112.7, 111.2, 70.9, 55.3, 54.1, 43.0, 37.1, 32.6, 28.7;
FTIR (cm^{-1}): 3396, 2895, 1600, 1451, 1256, 1045, 697;
HRMS (APCI$^-$): calcd for $C_{19}H_{26}O_2$ $[M]^-$ m/z = 285.1849; found 285.1860;
$[\alpha]_D^{23.2}$ = -9.30 (c = 0.830, EtOAc, l = 100 mm);
HPLC (CHIRALPAK® AD-H (4.6 x 250 mm), 5 mic, hexane/2-propanol 90:10, 1 mL/min) t_R 9.516 (major), 10.340 (minor), 85:15 er, 70% ee.

Racemic Material

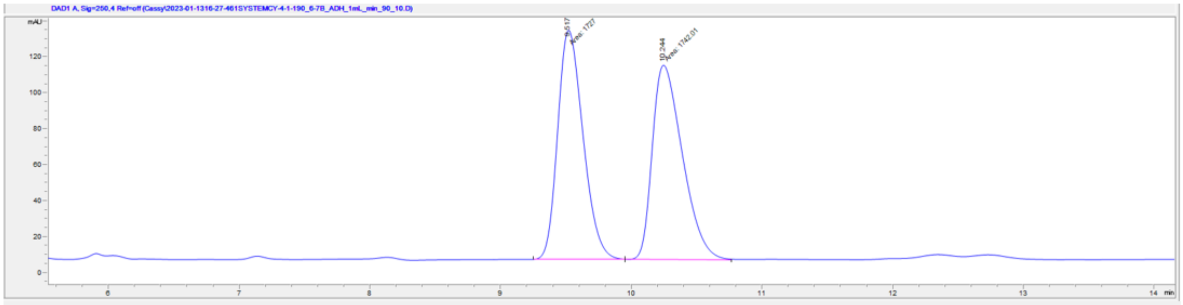

#	Time	Type	Area	Height	Width	Area%	Symmetry
1	9.517	MF	1727	127.7	0.2255	49.784	0.737
2	10.244	FM	1742	108.4	0.2679	50.216	0.58

Enantioenriched Material

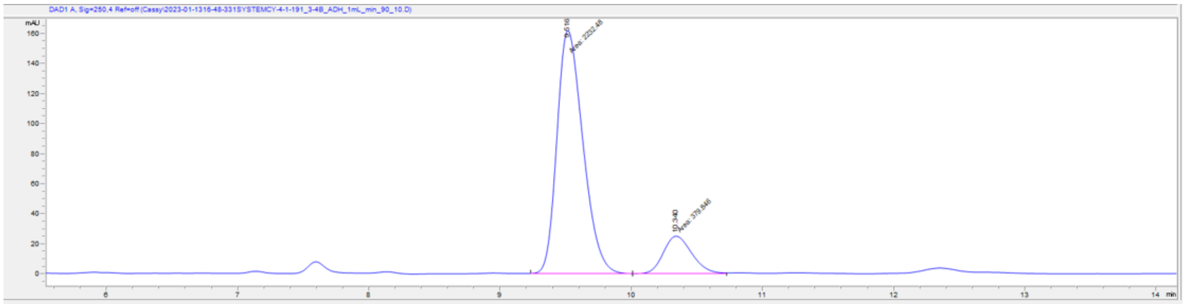

#	Time	Type	Area	Height	Width	Area%	Symmetry
1	9.516	MF	2232.5	161.5	0.2304	85.459	0.706
2	10.34	FM	379.8	25	0.2535	14.541	0.793

Gram scale HPLC (CHIRALPAK® AD-H (4.6 x 250 mm), 5 mic, hexane/2-propanol 90:10, 1 mL/min) t_R 10.093 (major), 10.899 (minor), 81:19 er, 62% ee.

Enantioenriched Material

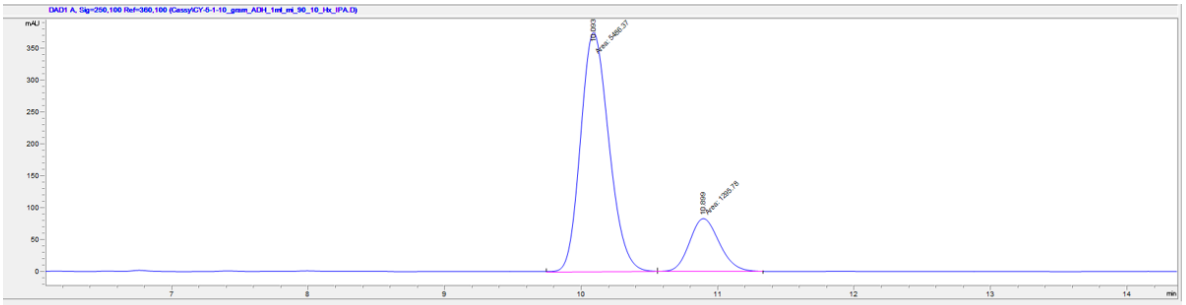

#	Time	Type	Area	Height	Width	Area%	Symmetry
1	10.093	MF	5486.4	377.4	0.2423	80.894	0.815
2	10.899	FM	1295.8	83.9	0.2573	19.106	0.848

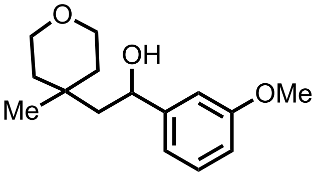

(*S*)-1-(3-methoxyphenyl)-2-(4-methyltetrahydro-2H-pyran-4-yl)ethan-1-ol (20):

Compound **20** was synthesized following the general procedure B, using 4-bromo-4-methyltetrahydropyran (71.6 mg, 0.4 mmol), vinylboronic acid pinacol ester (30.8 mg, 0.2 mmol), and 3-methoxyphenylmagnesium bromide (0.4 mL, 1 M solution in THF, 0.4 mmol), followed by the oxidation step using $NaBO_3 \bullet 4H_2O$ (92.3 mg, 0.6 mmol), THF (2 mL), and H_2O (2 mL). The product **20** was obtained as a colorless oil (34 mg, 68% yield) after purification by column chromatography on silica gel with gradient 0% to 20% EtOAc in DCM.

^{1}H NMR (400 MHz, $CDCl_3$): δ = 7.26 (t, *J* = 8.0 Hz, 1H), 6.92 – 6.90 (m, 2H), 6.81 (ddd, *J* = 8.2, 2.6, 1.1 Hz, 1H), 4.86 (dd, *J* = 8.8, 3.1 Hz, 1H), 3.82 (s, 3H), 3.76 – 3.59 (m, 4H), 1.88 (dd, *J*= 14.6, 8.7 Hz, 1H), 1.72 – 1.65 (m, 1H), 1.61 (dd, *J* = 14.6, 3.2 Hz, 1H), 1.54 – 1.43 (m, 2H), 1.36 – 1.30 (m, 1H), 1.13 (s, 3H);

^{13}C NMR (100 MHz, $CDCl_3$): δ = 159.9, 148.3, 129.7, 118.1, 112.9, 111.4, 71.6, 64.0, 63.9, 55.4, 51.4, 38.4, 38.3, 30.9, 24.4;

FTIR (cm^{-1}): 3403, 2920, 1585, 1435, 1256, 1038, 697;

HRMS (ESI$^+$): calcd for $C_{15}H_{21}O_2$ $[M+H-H_2O]^+$ *m/z* = 233.1536; found 233.1537;

$[\alpha]_D^{21.6}$ = -38.43 (*c* = 0.370, EtOAc, *l* = 100 mm);

HPLC (CHIRALPAK® AD-H (4.6 x 250 mm), 5 mic, hexane/2-propanol 85:15, 1 mL/min) t_R 7.886 (major), 12.338 (minor), 78:22 er, 56% ee.

Racemic Material

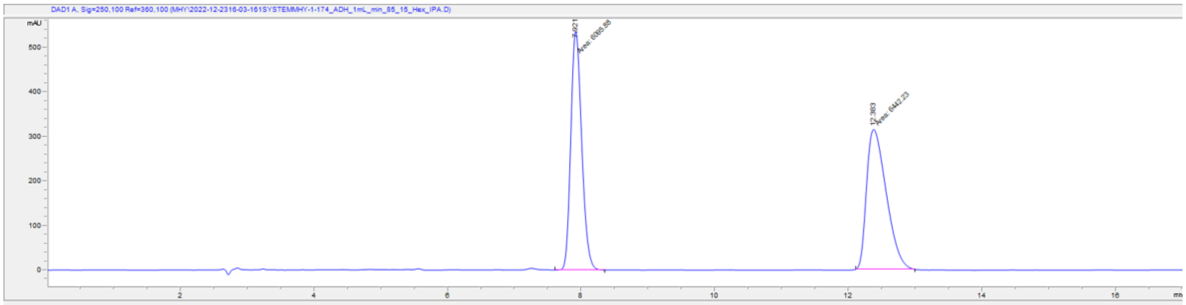

#	Time	Type	Area	Height	Width	Area%	Symmetry
1	7.921	MM	6095.9	536.5	0.1894	48.619	0.796
2	12.383	MM	6442.2	314.4	0.3415	51.381	0.552

Enantioenriched Material

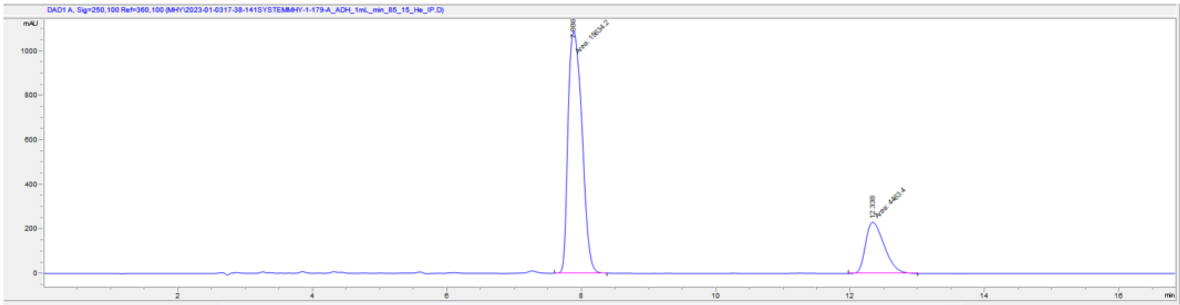

#	Time	Type	Area	Height	Width	Area%	Symmetry
1	7.886	MM	15634.2	1084.9	0.2402	77.791	0.644
2	12.338	MM	4463.4	230.1	0.3233	22.209	0.611

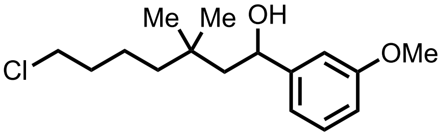

(*S*)-7-chloro-1-(3-methoxyphenyl)-3,3-dimethylheptan-1-ol (21):
Compound **21** was synthesized following the general procedure B, using 5-bromo-1-chloro-5-methylhexane (85.4 mg, 0.4 mmol), vinylboronic acid pinacol ester (30.8 mg, 0.2 mmol), and 3-methoxyphenylmagnesium bromide (0.4 mL, 1 M solution in THF, 0.4 mmol), followed by the oxidation step using $NaBO_3$•$4H_2O$ (92.3 mg, 0.6 mmol), THF (2 mL), and H_2O (2 mL). The product **21** was obtained as a colorless oil (34 mg, 60% yield) after purification by column chromatography on silica gel with gradient 5% to 15% EtOAc in hexanes.
^{1}H NMR (400 MHz, $CDCl_3$): δ = 7.25 (t, *J* = 8.0 Hz, 1H), 6.92 – 6.90 (m, 2H), 6.82 – 6.79 (m, 1H), 4.80 (dd, *J* = 8.5, 3.4 Hz, 1H), 3.82 (s, 3H), 3.53 (t, *J* = 6.7 Hz, 2H), 1.78 – 1.68 (m, 3H), 1.58 (dd, *J* = 14.6, 3.5 Hz, 1H), 1.48 – 1.26 (m, 4H), 0.98 (s, 3H), 0.97 (s, 3H);
^{13}C NMR (100 MHz, $CDCl_3$): δ = 159.9, 148.4, 129.7, 118.2, 112.9, 111.4, 72.2, 55.4, 50.7, 45.2, 42.0, 33.5, 33.1, 28.0, 27.9, 21.6;
FTIR (cm^{-1}): 3424, 2953, 1586, 1465, 1258, 1043, 698;
HRMS (APCI^{+}): calcd for $C_{16}H_{24}ClO$ $[M+H-H_2O]^{+}$ *m/z* = 267.1510; found 267.1509;
$[\alpha]_D^{21.3}$ = -29.85 (*c* = 0.475, EtOAc, *l* = 100 mm);
HPLC (CHIRALPAK® AD-H (4.6 x 250 mm), 5 mic, hexane/2-propanol 92:8, 1 mL/min) t_R 8.015 (major), 8.721 (minor), 79:21 er, 58% ee.

Racemic Material

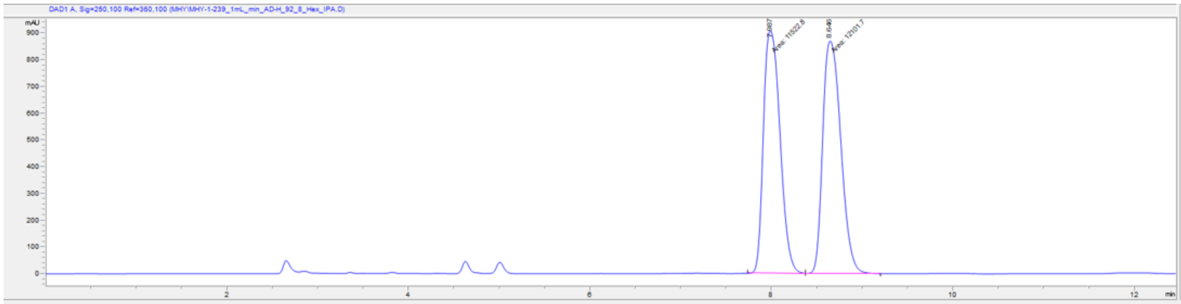

#	Time	Type	Area	Height	Width	Area%	Symmetry
1	7.987	MF	11522.8	906.5	0.2118	48.775	0.697
2	8.646	FM	12101.7	869.3	0.232	51.225	0.683

Enantioenriched Material

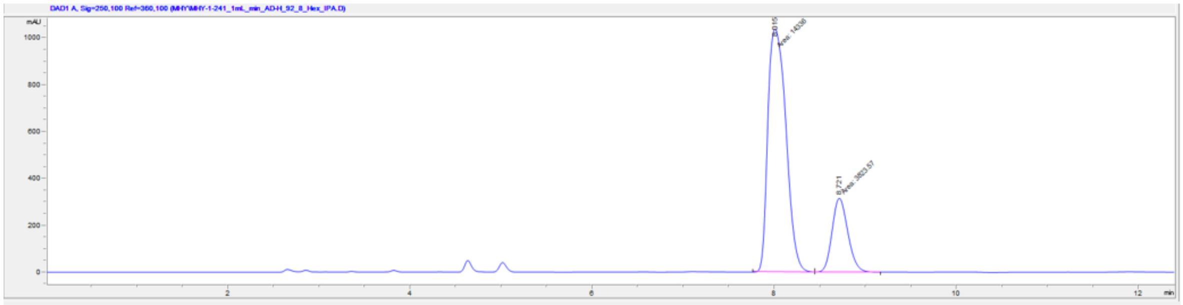

#	Time	Type	Area	Height	Width	Area%	Symmetry
1	8.015	MF	14336	1034.6	0.2309	78.945	0.637
2	8.721	FM	3823.6	315.4	0.2021	21.055	0.814

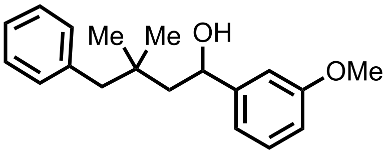

1-(3-methoxyphenyl)-3,3-dimethyl-4-phenylbutan-1-ol (22):

Compound **22** was synthesized following the modified general procedure B, using (2-bromo-2-methylpropyl)benzene (107 mg, 0.5 mmol), vinylboronic acid pinacol ester (30.8 mg, 0.2 mmol), and 3-methoxyphenylmagnesium bromide (0.4 mL, 1 M solution in THF, 0.4 mmol), followed by the oxidation step using $NaBO_3$•$4H_2O$ (92.3 mg, 0.6 mmol), THF (2 mL), and H_2O (2 mL). The product **22** was obtained as a colorless oil (33 mg, 58% yield) after purification by column chromatography on silica gel with gradient 5% to 15% EtOAc in hexanes.

^{1}H NMR (400 MHz, $CDCl_3$): δ = 7.28 – 7.24 (m, 3H), 7.22 – 7.15 (m, 3H), 6.94 – 6.92 (m, 2H), 6.81 (ddd, *J* = 8.3, 2.5, 1.2 Hz, 1H), 4.90 (dd, *J* = 8.7, 3.2 Hz, 1H), 3.82 (s, 3H), 2.63 (q, *J* = 13.0 Hz, 2H), 1.82 (dd, *J* = 14.6, 8.6 Hz, 1H), 1.68 (b.s., 1H), 1.62 (dd, *J* = 14.6, 3.2 Hz, 1H), 1.03 (s, 3H), 0.96 (s, 3H);

^{13}C NMR (100 MHz, $CDCl_3$): δ = 159.9, 148.3, 139.2, 130.9, 129.7, 127.8, 126.0, 118.2, 113.0, 111.5, 72.3, 55.4, 51.2, 49.4, 34.5, 27.4;

FTIR (cm^{-1}): 3424, 2928, 1585, 1466, 1257, 1046, 700;

HRMS (APCI$^+$): calcd for $C_{19}H_{23}O$ $[M+H-H_2O]^+$ m/z = 267.1743; found 267.1742;

$[\alpha]_D^{21.2}$ = -29.11 (*c* = 0.325, EtOAc, *l* = 100 mm);

HPLC (CHIRALPAK® AD-H (4.6 x 250 mm), 5 mic, hexane/2-propanol 94:6, 1 mL/min) t_R 11.458 (major), 12.661 (minor), 76:24 er, 52% ee.

Racemic Material

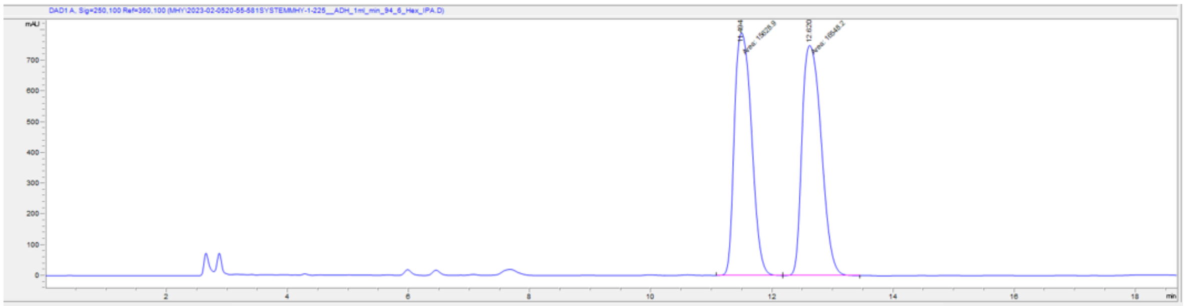

#	Time	Type	Area	Height	Width	Area%	Symmetry
1	11.494	MF	15628.9	791	0.3293	48.572	0.695
2	12.62	FM	16548.2	750.2	0.3677	51.428	0.647

Enantioenriched Material

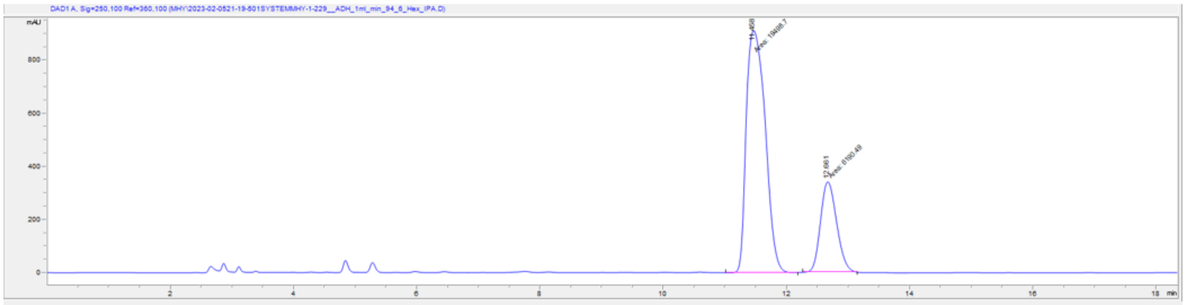

#	Time	Type	Area	Height	Width	Area%	Symmetry
1	11.458	MM	19498.7	911.6	0.3565	75.902	0.625
2	12.661	MM	6190.5	339.7	0.3037	24.098	0.802

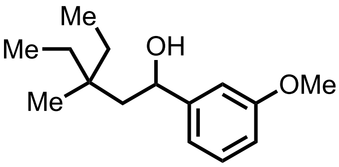

(*S*)-3-ethyl-1-(3-methoxyphenyl)-3-methylpentan-1-ol (23):

Compound **23** was synthesized following the general procedure B, using 3-bromo-3-methylpentane (66.0 mg, 0.4 mmol), vinylboronic acid pinacol ester (30.8 mg, 0.2 mmol), and 3-methoxyphenylmagnesium bromide (0.4 mL, 1 M solution in THF, 0.4 mmol), followed by the oxidation step using $NaBO_3$•$4H_2O$ (92.3 mg, 0.6 mmol), THF (2 mL), and H_2O (2 mL). The product **23** was obtained as a colorless oil (24 mg, 51% yield) after purification by column chromatography on silica gel with gradient 5% to 10% EtOAc in hexanes.

^{1}H NMR (400 MHz, $CDCl_3$): δ = 7.25 (t, *J* = 8.0 Hz, 1H), 6.92 (dt, *J* = 3.9, 1.6 Hz, 2H), 6.80 (ddd, *J* = 8.1, 2.6, 1.1 Hz, 1H), 4.79 (dd, *J* = 8.4, 3.2 Hz, 1H), 3.82 (s, 3H), 1.73 (dd, *J* = 14.8, 8.5 Hz, 1H), 1.66 (b.s., 1H), 1.56 (dd, *J* = 14.8, 3.3 Hz, 1H), 1.40 – 1.29 (m, 4H), 0.91 (s, 3H), 0.81 (td, *J* = 7.5, 1.2 Hz, 6H);

^{13}C NMR (100 MHz, $CDCl_3$): δ = 159.9, 148.7, 129.7, 118.2, 112.8, 111.4, 72.1, 55.4, 47.9, 35.5, 31.7, 31.6, 24.7, 8.1;

FTIR (cm^{-1}): 3432, 2961, 1586, 1456, 1259, 1046, 782, 695;

HRMS (ESI$^+$): calcd for $C_{15}H_{23}O$ $[M+H-H_2O]^+$ *m/z* = 219.1743; found 219.1745;

$[\alpha]_D^{21.9}$ = -37.54 (*c* = 0.570, EtOAc, *l* = 100 mm);

HPLC (CHIRALCEL® OD-H (4.6 x 250 mm), 5 mic, hexane/2-propanol 95:5, 1 mL/min) t_R 10.751 (major), 21.683 (minor), 84:16 er, 68% ee.

Racemic Material

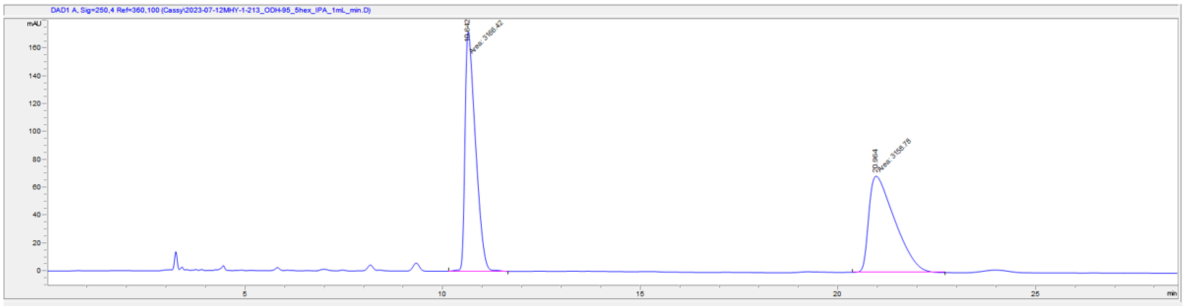

#	Time	Type	Area	Height	Width	Area%	Symmetry
1	10.642	MM	3166.4	171.9	0.3071	50.060	0.437
2	20.964	MM	3158.8	69	0.7625	49.940	0.381

Enantioenriched Material

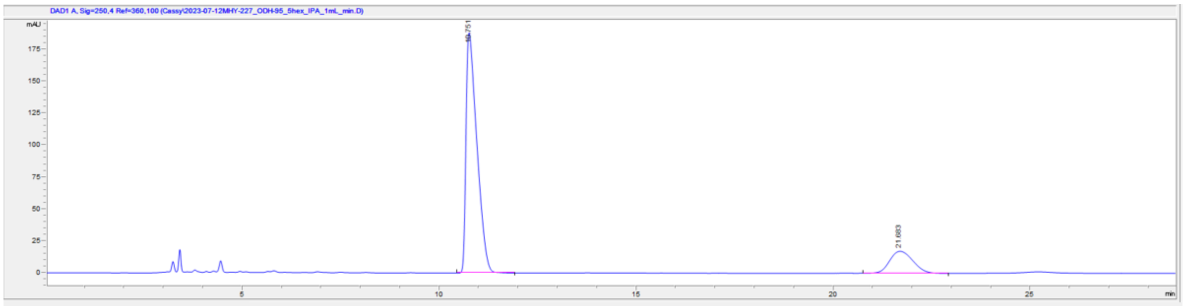

#	Time	Type	Area	Height	Width	Area%	Symmetry
1	10.751	BB	3601.3	188	0.2855	83.637	0.372
2	21.683	BB	704.6	17.2	0.6461	16.363	0.786

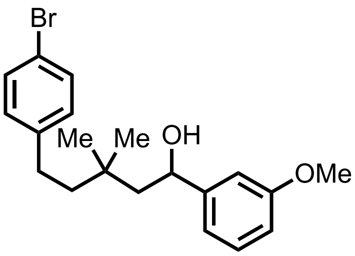

(*S*)-5-(4-bromophenyl)-1-(3-methoxyphenyl)-3,3-dimethylpentan-1-ol (24):
Compound **24** was synthesized following the modified general procedure B, using $Fe(acac)_3$ (14.1 mg, 0.04 mmol), (*R*,*R*)-BenzP* (22.6 mg, 0.08 mmol), 1-bromo-4-(3-bromo-3-methylbutyl)benzene (122 mg, 0.4 mmol), vinylboronic acid pinacol ester (30.8 mg, 0.2 mmol), and 3-methoxyphenylmagnesium bromide (0.4 mL, 1 M solution in THF, 0.4 mmol), followed by the oxidation step using $NaBO_3•4H_2O$ (92.3 mg, 0.6 mmol), THF (2 mL), and H_2O (2 mL). The product **24** was obtained as a colorless oil (46 mg, 61% yield) after purification by column chromatography on silica gel with gradient 0% to 2% EtOAc in DCM.
^{1}H NMR (400 MHz, $CDCl_3$): δ = 7.38 – 7.37 (m, 2H), 7.27 (t, *J* = 8.0 Hz, 1H), 7.03 (d, *J* = 8.3 Hz, 2H), 6.94 – 6.92 (m, 2H), 6.82 (ddd, *J* = 8.2, 2.6, 1.0 Hz, 1H), 4.84 (dd, *J* = 8.7, 3.2 Hz, 1H), 3.82 (s, 3H), 2.54 (dtd, *J* = 44.3, 13.0, 5.2 Hz, 2H), 1.85 (dd, *J* = 14.6, 8.5 Hz, 1H), 1.72 (b.s., 1H), 1.66 – 1.50 (m, 3H), 1.06 (s, 3H), 1.04 (s, 3H);
^{13}C NMR (100 MHz, $CDCl_3$): δ = 160.0, 148.4, 142.5, 131.4, 130.3, 129.8, 119.3, 118.2, 112.9, 111.5, 72.2, 55.4, 50.6, 44.9, 33.3, 30.3, 28.0;

FTIR (cm^{-1}): 3429, 2953, 1585, 1487, 1260, 1011, 803, 698;
HRMS ($APCI^+$): calcd for $C_{20}H_{24}BrO$ $[M+H-H_2O]^+$ m/z = 359.1005; found 359.1003;
$[\alpha]_D^{21.5}$ = -22.88 (c = 0.430, EtOAc, l = 100 mm);
HPLC (CHIRALPAK® AD-H (4.6 x 250 mm), 5 mic, hexane/2-propanol 92:8, 1 mL/min) t_R 10.676 (major), 11.823 (minor), 80:20 er, 60% ee.

Racemic Material

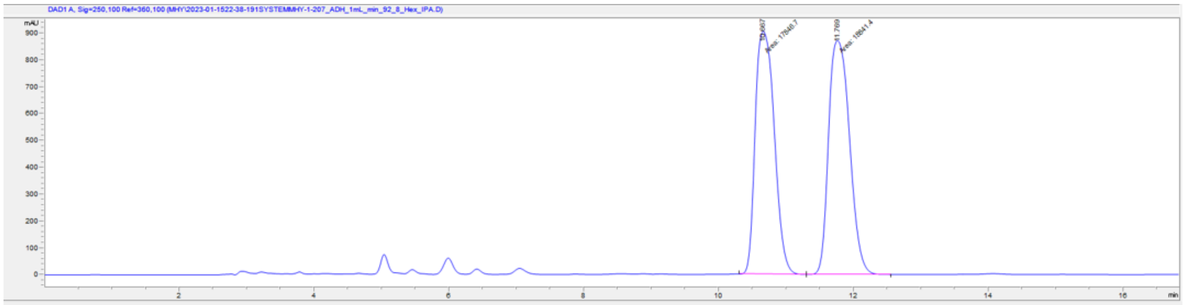

#	Time	Type	Area	Height	Width	Area%	Symmetry
1	10.667	MF	17846.7	904.2	0.3289	48.644	0.705
2	11.769	FM	18841.4	871.3	0.3604	51.356	0.719

Enantioenriched Material

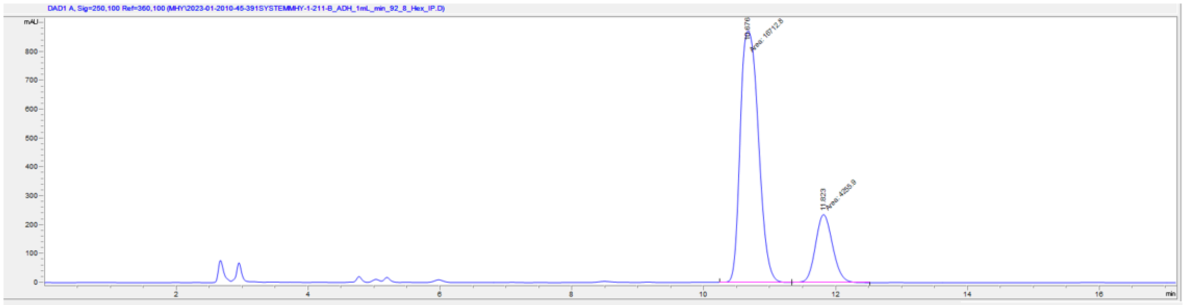

#	Time	Type	Area	Height	Width	Area%	Symmetry
1	10.676	MF	16712.8	872.1	0.3194	79.704	0
2	11.823	FM	4255.9	235.4	0.3013	20.296	0.865

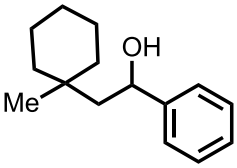

(*S*)-2-(1-methylcyclohexyl)-1-phenylethan-1-ol (25):
Compound **25** was synthesized following the general procedure B, using 1-bromo-1-methylcyclohexane (70.8 mg, 0.4 mmol), vinylboronic acid pinacol ester (30.8 mg, 0.2 mmol), and phenylmagnesium bromide (0.4 mL, 1 M solution in THF, 0.4 mmol), followed by the oxidation step using $NaBO_3•4H_2O$ (92.3 mg, 0.6 mmol), THF (2 mL), and H_2O (2 mL). The product **25** was obtained as a colorless oil (22 mg, 50% yield) after purification by column chromatography on silica gel with gradient 5% to 12% EtOAc in hexanes. Spectra matched those reported previously.[15]
^{1}H NMR (400 MHz, $CDCl_3$): δ = 7.37 – 7.24 (m, 5H), 4.87 (dd, J = 8.3, 3.5 Hz, 1H), 1.79 (dd, J = 14.7, 8.3 Hz, 1H), 1.64 (dd, J = 14.7, 3.5 Hz, 1H), 1.51 – 1.25 (m, 10H), 1.01 (s, 3H);
$[\alpha]_D^{21.4}$ = -41.65 (c = 0.875, EtOAc, l = 100 mm);

HPLC (CHIRALCEL® OD-H (4.6 x 250 mm), 5 mic, hexane/2-propanol 98:2, 1 mL/min) t_R 8.276 (major), 11.159 (minor), 85:15 er, 70% ee.

Racemic Material

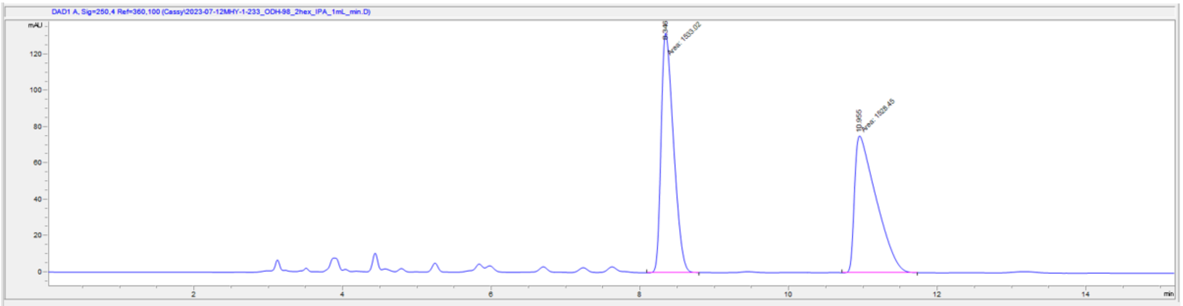

#	Time	Type	Area	Height	Width	Area%	Symmetry
1	8.346	MM	1533	132.1	0.1934	50.075	0.567
2	10.955	MM	1528.5	75.4	0.338	49.925	0.295

Enantioenriched Material

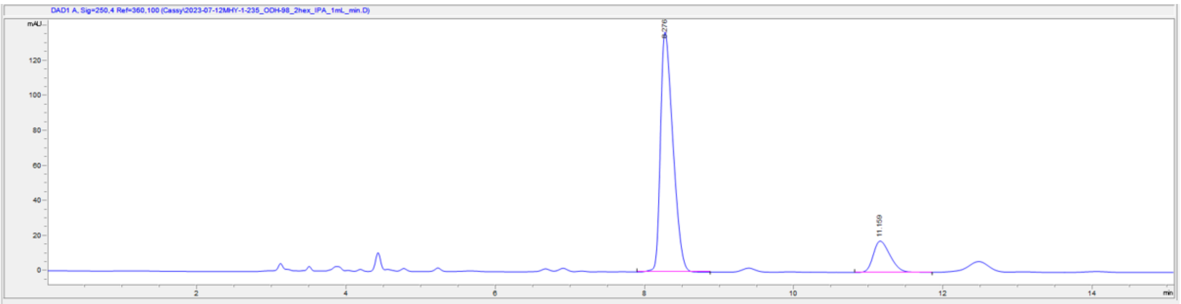

#	Time	Type	Area	Height	Width	Area%	Symmetry
1	8.276	BB	1586.8	137.1	0.1771	84.959	0.555
2	11.159	BB	280.9	17.9	0.2432	15.041	0.664

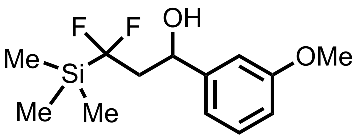

(*S*)-3,3-difluoro-1-(3-methoxyphenyl)-3-(trimethylsilyl)propan-1-ol (26):

Compound **26** was synthesized following the modified general procedure B, using $Fe(acac)_3$ (14.1 mg, 0.04 mmol), (*R*,*R*)-BenzP* (22.6 mg, 0.08 mmol), (bromodifluoromethyl)trimethylsilane (81.2 mg, 0.4 mmol), vinylboronic acid pinacol ester (30.8 mg, 0.2 mmol), and 3-methoxyphenylmagnesium bromide (0.4 mL, 1 M solution in THF, 0.4 mmol), followed by the oxidation step using $NaBO_3$•$4H_2O$ (92.3 mg, 0.6 mmol), THF (2 mL), and H_2O (2 mL). The product **26** was obtained as a colorless oil (21 mg, 38% yield) after purification by column chromatography on silica gel with gradient 5% to 10% EtOAc in hexanes.

^{1}H NMR (400 MHz, $CDCl_3$): δ = 7.30 – 7.26 (m, 1H), 6.97 – 6.94 (m, 2H), 6.84 – 6.81 (m, 1H), 5.23 (dd, *J* = 9.4, 2.2 Hz, 1H), 3.83 (s, 3H), 2.46 – 2.02 (m, 3H), 0.20 (s, 9H);

^{13}C NMR (100 MHz, $CDCl_3$): δ = 160.0, 145.7, 130.8 (t, *J* = 257.5 Hz), 129.7, 118.0, 113.3, 111.3, 68.6 (dd, *J* = 7.9, 5.8 Hz), 55.4, 45.3 (t, *J* = 18.5 Hz), -4.5;

^{19}F NMR (376 MHz, $CDCl_3$): δ = -113.15 (ddd, *J* = 315.0, 30.2, 12.5 Hz, 1F), -114.62 (ddd, *J* = 315.5, 32.5, 12.7 Hz, 1F);

FTIR (cm^{-1}): 3460, 2958, 1601, 1488, 1252, 1156, 1122, 1043, 844;

HRMS (ESI$^+$): calcd for $C_{13}H_{24}F_2O_2NSi$ $[M+NH_4]^+$ *m/z* = 292.1539; found 292.1533;

$[\alpha]_D^{21.3}$ = -27.84 (*c* = 0.755, EtOAc, *l* = 100 mm);
HPLC (CHIRALPAK® AD-H (4.6 x 250 mm), 5 mic, hexane/2-propanol 98.5:1.5, 1 mL/min) t_R 16.167 (major), 17.433 (minor), 75:25 er, 50% ee.

Racemic Material

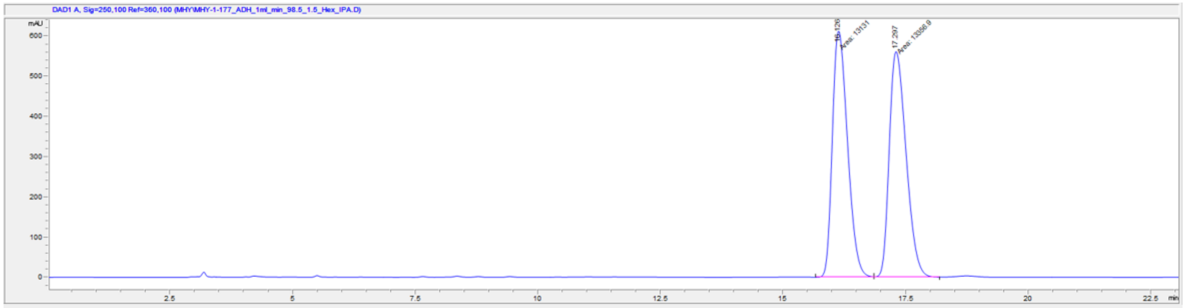

#	Time	Type	Area	Height	Width	Area%	Symmetry
1	16.126	MF	13131	610.1	0.3587	49.573	0.68
2	17.297	FM	13356.9	559.7	0.3978	50.427	0.662

Enantioenriched Material

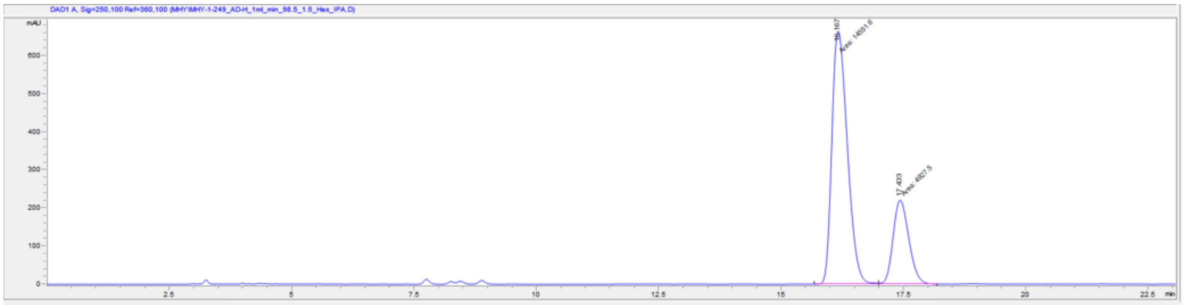

#	Time	Type	Area	Height	Width	Area%	Symmetry
1	16.167	MF	14551.6	662.2	0.3662	74.704	0.661
2	17.433	FM	4927.5	220	0.3733	25.296	0.79

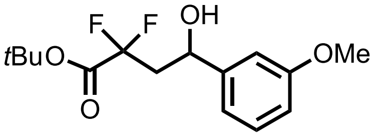

(*S*)-tert-butyl (R)-2,2-difluoro-4-hydroxy-4-(3-methoxyphenyl)butanoate (27):
Compound **27** was synthesized following the modified general procedure B, using *tert*-butyl 2-bromo-2,2-difluoroacetate (185 mg, 0.8 mmol), vinylboronic acid pinacol ester (30.8 mg, 0.2 mmol), and 3-methoxyphenylmagnesium bromide (0.9 mL, 1 M solution in THF, 0.9 mmol). The Bpin derivative (35% crude yield by ^{1}H NMR analysis) was quickly purified by flash chromatography on silica gel with EtOAc/DCM/Hexanes (1:2:20) before undergoing the oxidation step because the final alcohol product overlapped with side products generated in the first step. The semi-pure Bpin derivative was treated with $NaBO_3 \cdot 4H_2O$ (92.3 mg, 0.6 mmol) in THF (1 mL) and H_2O (1 mL) to provide **27** as a colorless oil (10 mg, 16% yield) after purification by column chromatography on silica gel with gradient 0% to 2% EtOAc in DCM.
^{1}H NMR (400 MHz, $CDCl_3$): δ = 7.23 (d, *J* = 7.7 Hz, 1H), 6.91 – 6.89 (m, 2H), 6.81 – 6.78 (m, 1H), 4.98 (dt, *J* = 10.2, 3.0 Hz, 1H), 3.78 (s, 3H), 2.65 – 2.50 (m, 1H), 2.36 – 2.25 (m, 1H), 2.19 (d, *J* = 3.4 Hz, 1H), 1.50 (s, 9H);

^{13}C NMR (100 MHz, CDCl$_3$): δ = 163.3 (dd, *J* = 32.6, 31.0 Hz), 160.1, 144.8, 129.9, 117.9, 115.3 (dd, *J* = 251, 248 Hz), 113.7, 111.3, 84.7, 68.9 (dd, *J* = 7.8, 3.8 Hz), 55.4, 43.9 (t, *J* = 22.6 Hz), 27.9;
^{19}F NMR (376 MHz, CDCl$_3$): δ = -102.10 (ddd, *J* = 260.9, 14.0, 10.6 Hz, 1F), -107.03 (ddd, *J* = 262.0, 21.5, 13.2 Hz, 1F);
FTIR (cm^{-1}): 3500, 2981, 1752, 1602, 1370, 1156, 1082, 793;
HRMS (ESI$^+$): calcd for $C_{15}H_{24}F_2O_4N$ [M+NH$_4$]$^+$ *m/z* = 320.1668; found 320.1662;
$[\alpha]_D^{21.1}$ = -31.35 (*c* = 0.490, EtOAc, *l* = 100 mm);
HPLC (CHIRALCEL® OD-H (4.6 x 250 mm), 5 mic, hexane/2-propanol 96:4, 1 mL/min) t_R 12.017 (major), 19.397 (minor), 74:26 er, 48% ee.

Racemic Material

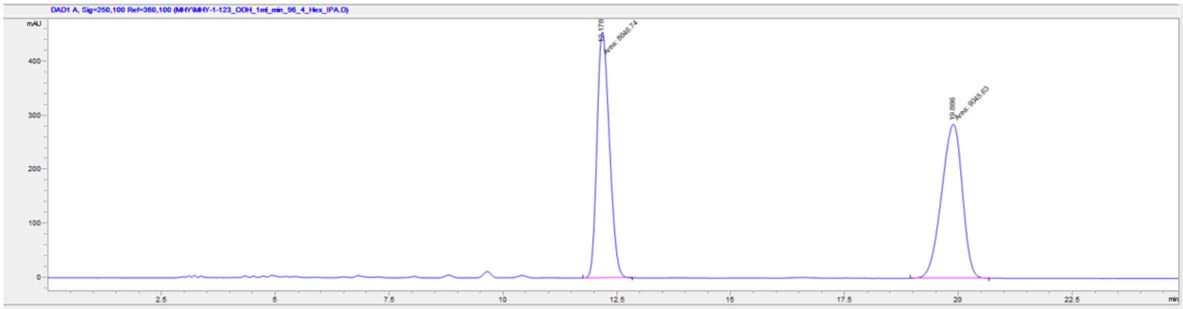

#	Time	Type	Area	Height	Width	Area%	Symmetry
1	12.178	MM	8646.7	454.6	0.317	48.873	0.737
2	19.886	MM	9045.6	284.8	0.5293	51.127	1.265

Enantioenriched Material

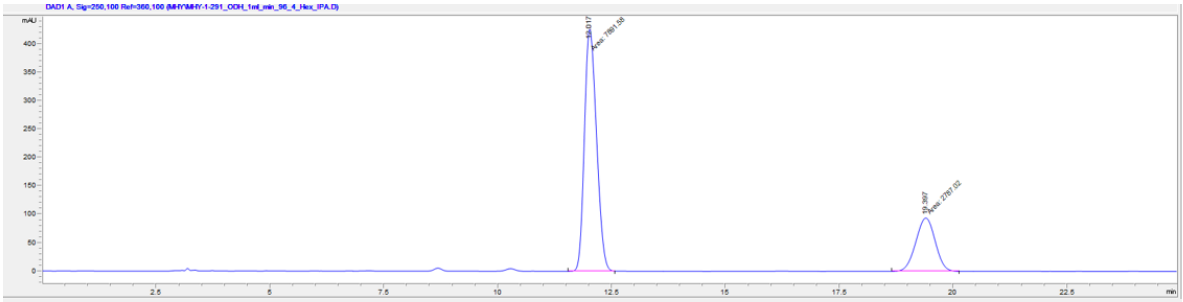

#	Time	Type	Area	Height	Width	Area%	Symmetry
1	12.017	MM	7891.6	426.9	0.3081	73.901	0.756
2	19.397	MM	2787	94.1	0.4934	26.099	1.032

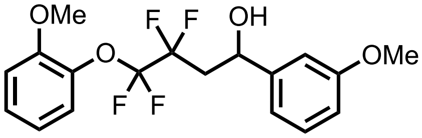

(*S*)-3,3,4,4-tetrafluoro-1-(4-fluorophenyl)-4-(3-methoxyphenoxy)butan-1-ol (28):
Compound **28** was synthesized following the general procedure B, using 1-(2-bromo-1,1,2,2-tetrafluoroethoxy)-2-methoxybenzene (121.22 mg, 0.4 mmol), vinylboronic acid pinacol ester (30.8 mg, 0.2 mmol), and 4-fluorophenylmagnesium bromide (0.4 mL, 1 M solution in THF, 0.4 mmol), followed by the oxidation step using NaBO$_3$•4H$_2$O (92.3 mg, 0.6 mmol), THF (2 mL), and H$_2$O (2 mL). The product **28** was obtained as a clear oil (32.8 mg, 44% yield) after purification by column chromatography on silica gel with gradient 0% to 20% EtOAc in hexane.

^{1}H NMR (400 MHz, CDCl$_3$): δ = 7.30 – 7.20 (m, 3H), 7.00 – 6.90 (m, 4H), 6.84 (ddd, *J* = 8.2, 2.4, 1.2 Hz, 1H), 5.27 (d, *J* = 9.1 Hz, 1H), 3.81 (s, 3H), 3.81 (s, 3H), 2.78 – 2.51 (m, 3H);
^{13}C NMR (100 MHz, CDCl$_3$): δ = 159.9, 152.2, 145.0, 137.6, 129.8, 127.6, 123.8, 120.7, 120.3 – 114.2 (m), 117.9, 113.5, 112.7, 111.2, 68.1 (t, *J* = 3.7 Hz), 56.0, 55.3, 41.3 (t, *J* = 20 Hz);
^{19}F NMR (376 MHz, CDCl$_3$): δ = -88.02 to -88.85 (m, 2F), -113.53 to -115.64 (m, 2F);
FTIR (cm^{-1}): 3458, 2946, 1602, 1503, 1170, 1107;
HRMS (ESI$^+$): calcd for $C_{18}H_{22}F_4O_4N$ $[M+NH_4]^+$ *m/z* = 392.1479; found 392.1472;
$[\alpha]_D^{23.2}$ = -1.55 (*c* = 0.710, EtOAc, *l* = 100mm);
HPLC (CHIRALCEL® OD-H (4.6 x 250 mm), 5 mic, hexane/2-propanol 90:10, 1 mL/min) t_R 9.878 (major), 13.129 (minor), 76:24 er, 52 ee.

Racemic Material

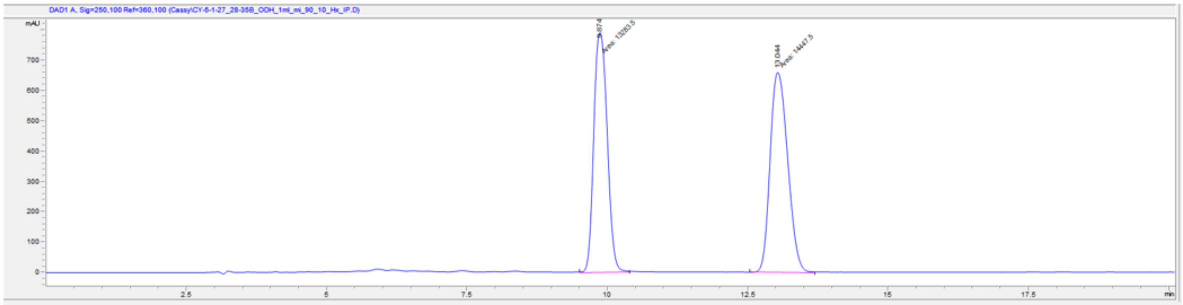

#	Time	Type	Area	Height	Width	Area%	Symmetry
1	9.874	MM	13283.5	794.3	0.2787	47.901	0.823
2	13.044	MM	14447.5	661.6	0.364	52.099	0.744

Enantioenriched Material

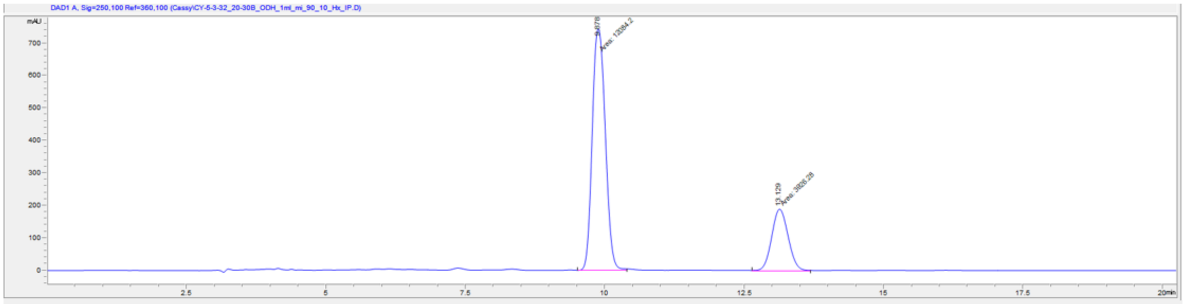

#	Time	Type	Area	Height	Width	Area%	Symmetry
1	9.878	MM	12084.2	742.3	0.2713	75.477	0.834
2	13.129	MM	3926.3	189.7	0.345	24.523	0.902

7. Experimental Spectra

Compound S1. ^1H NMR ($CDCl_3$, 400 MHz).

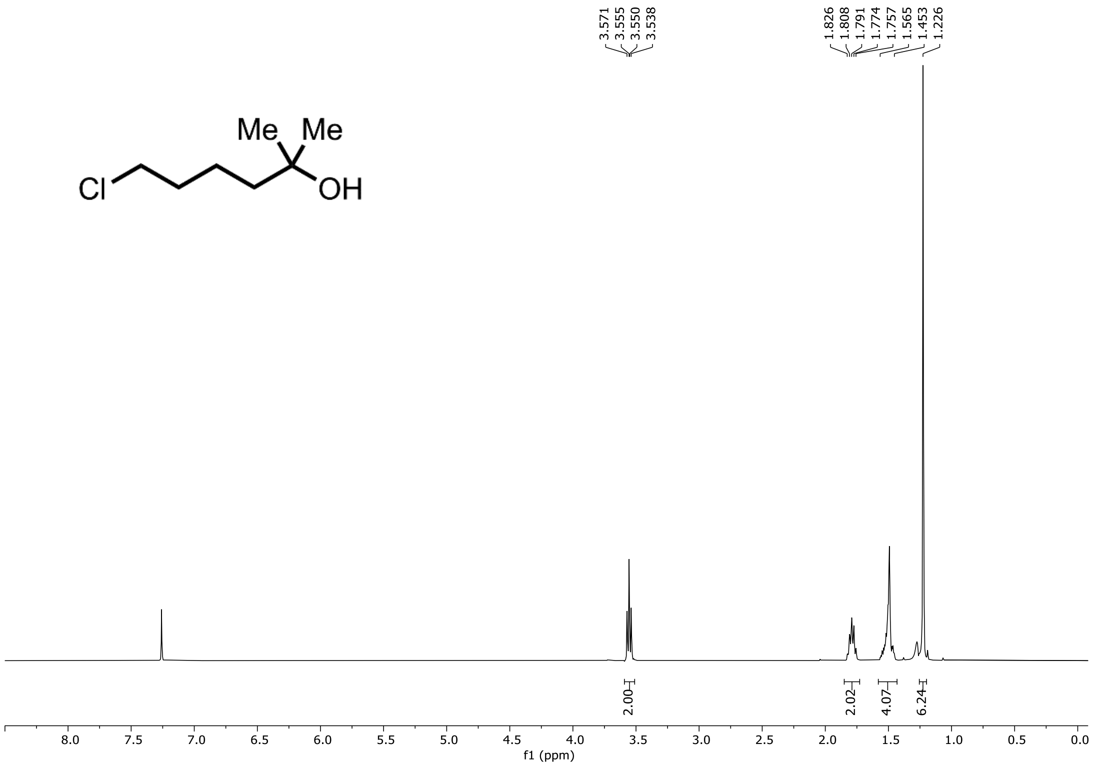

Compound S2. ^1H NMR ($CDCl_3$, 400 MHz).

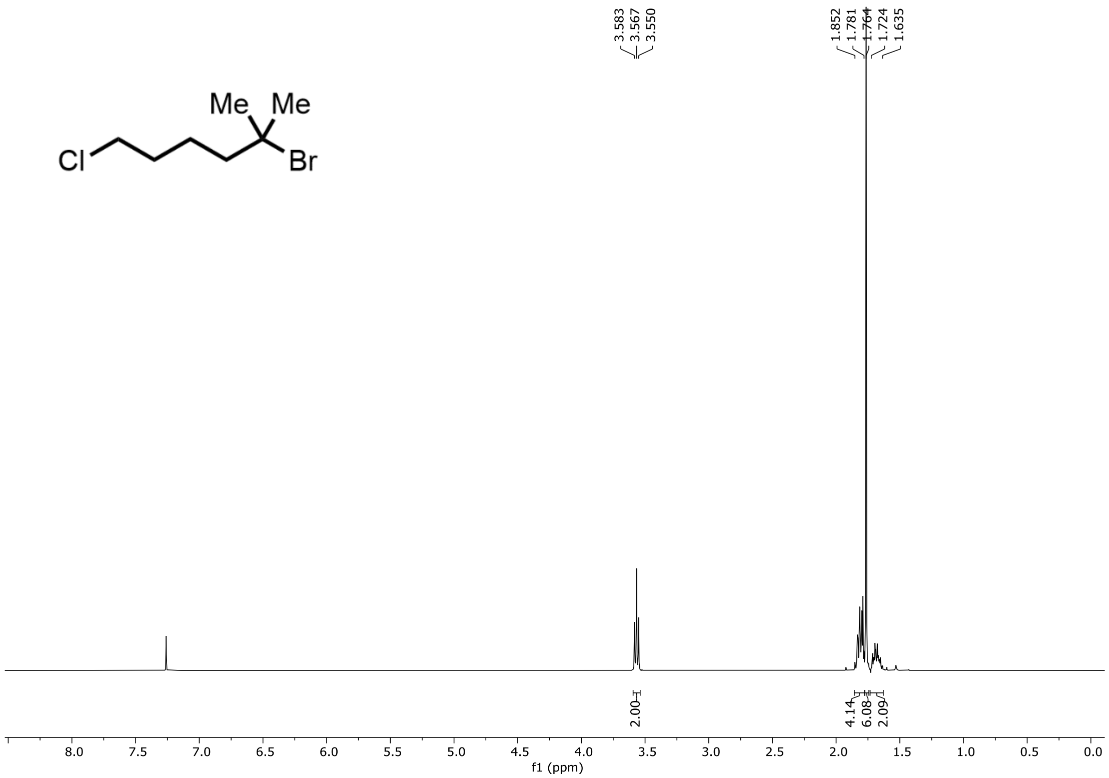

Compound S3. ^{1}H NMR ($CDCl_3$, 400 MHz).

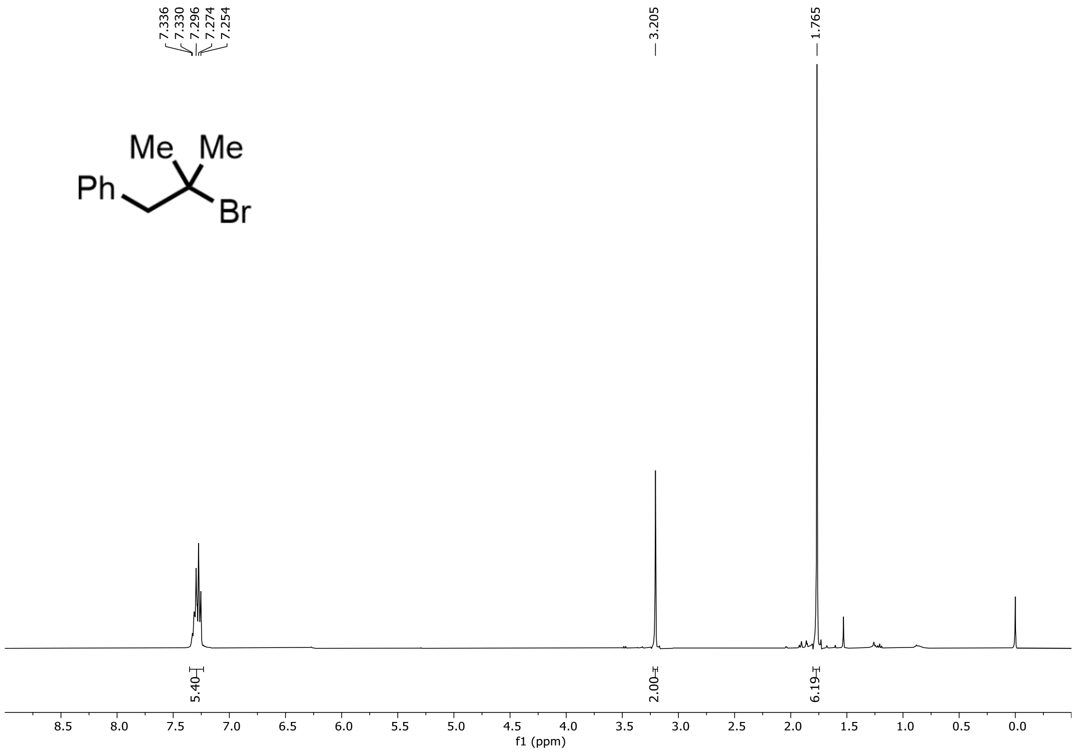

Compound S5. Top ^{1}H NMR ($CDCl_3$, 400 MHz). Bottom: ^{13}C NMR ($CDCl_3$, 100 MHz).

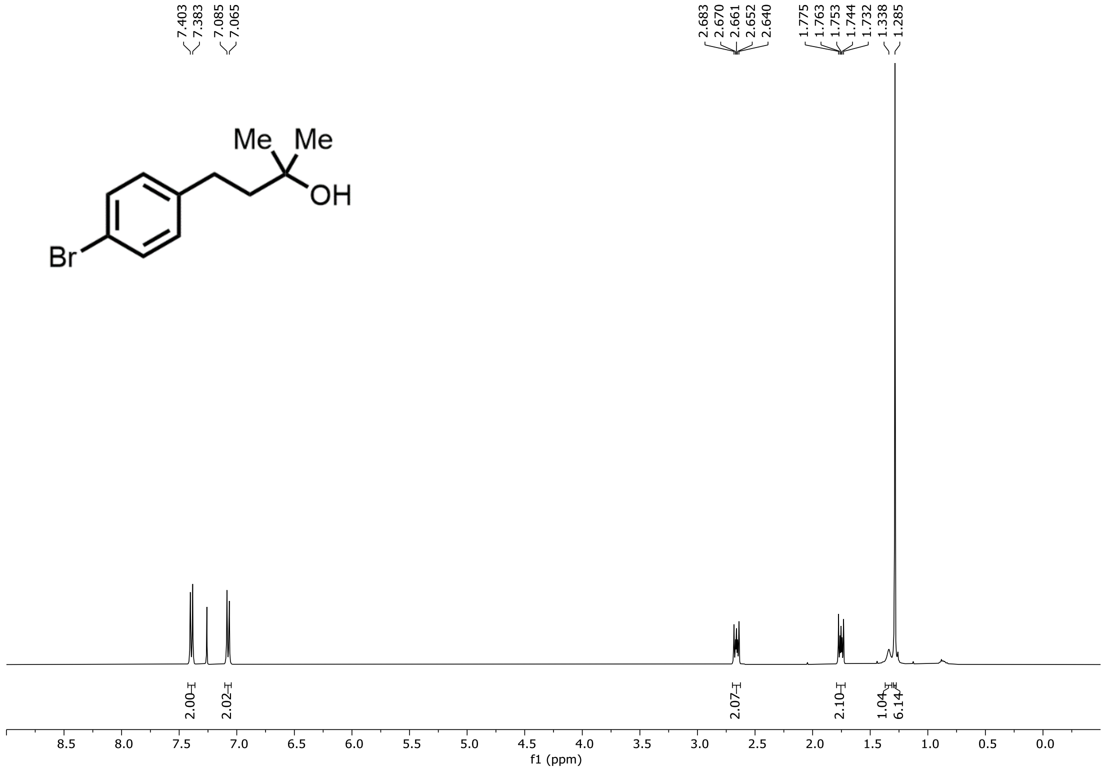

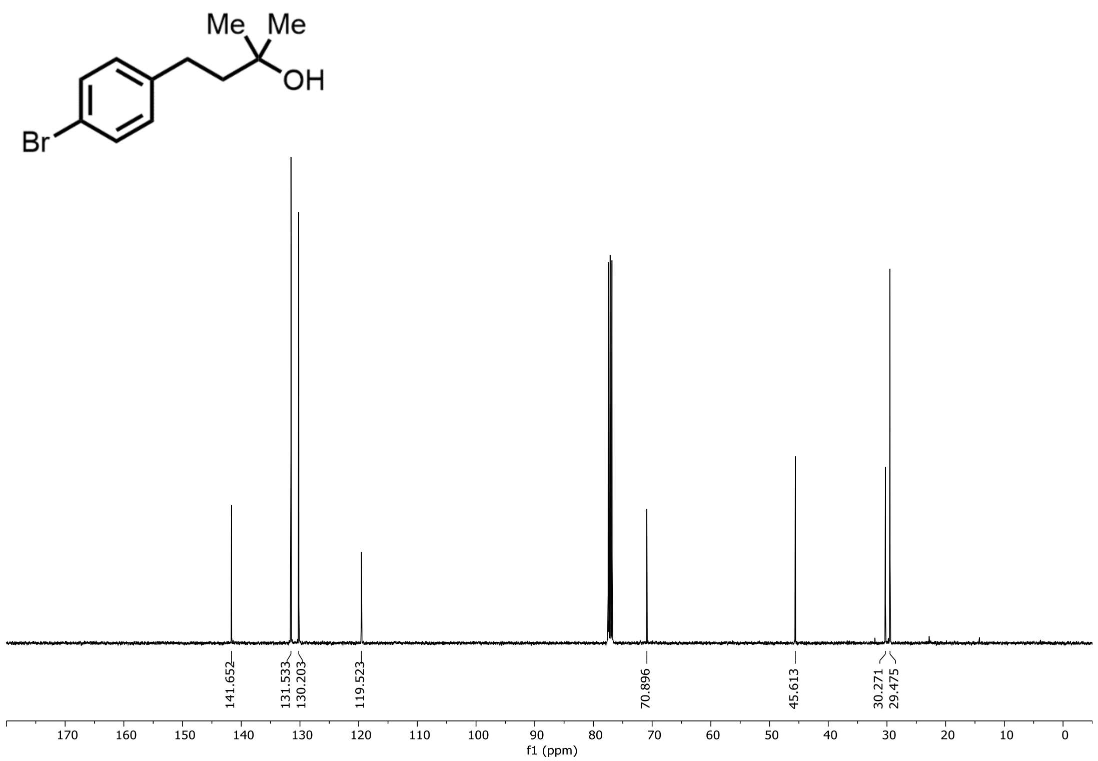

Compound S6. Top: ^{1}H NMR ($CDCl_3$, 400 MHz). Bottom: ^{13}C NMR ($CDCl_3$, 100 MHz)

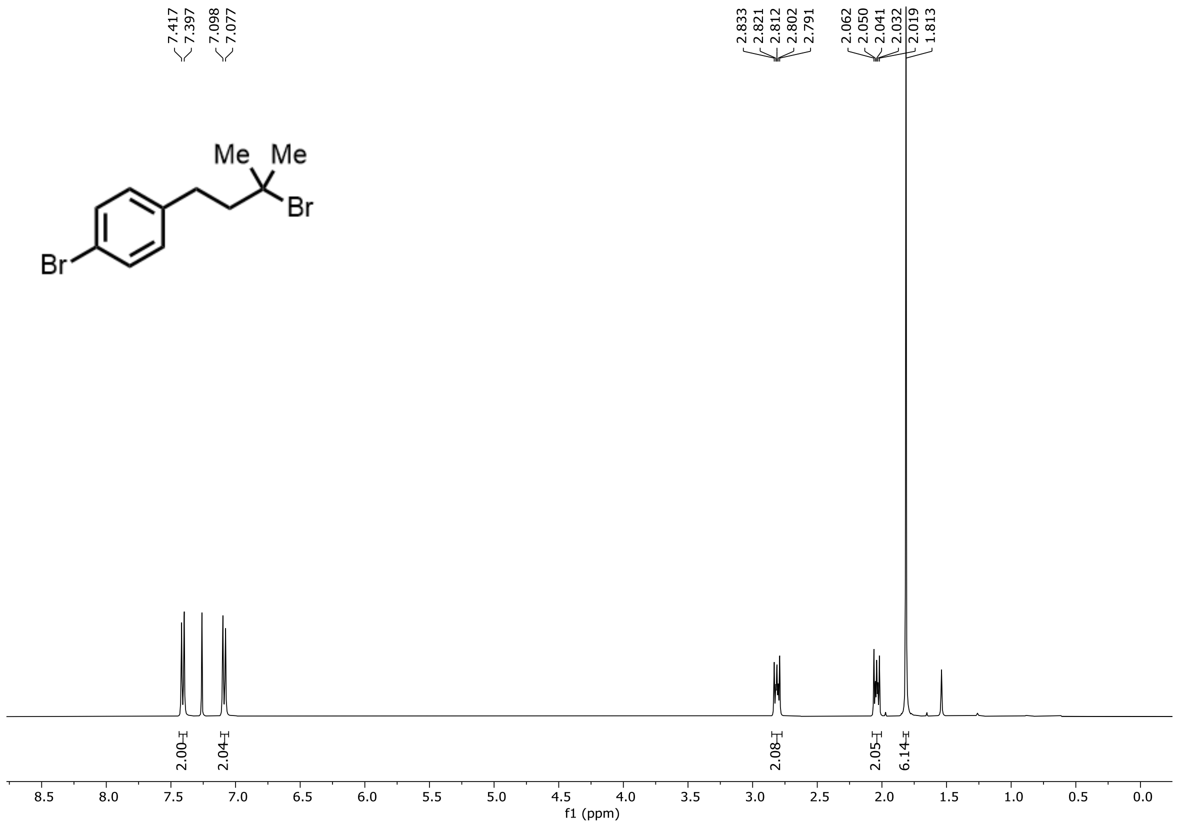

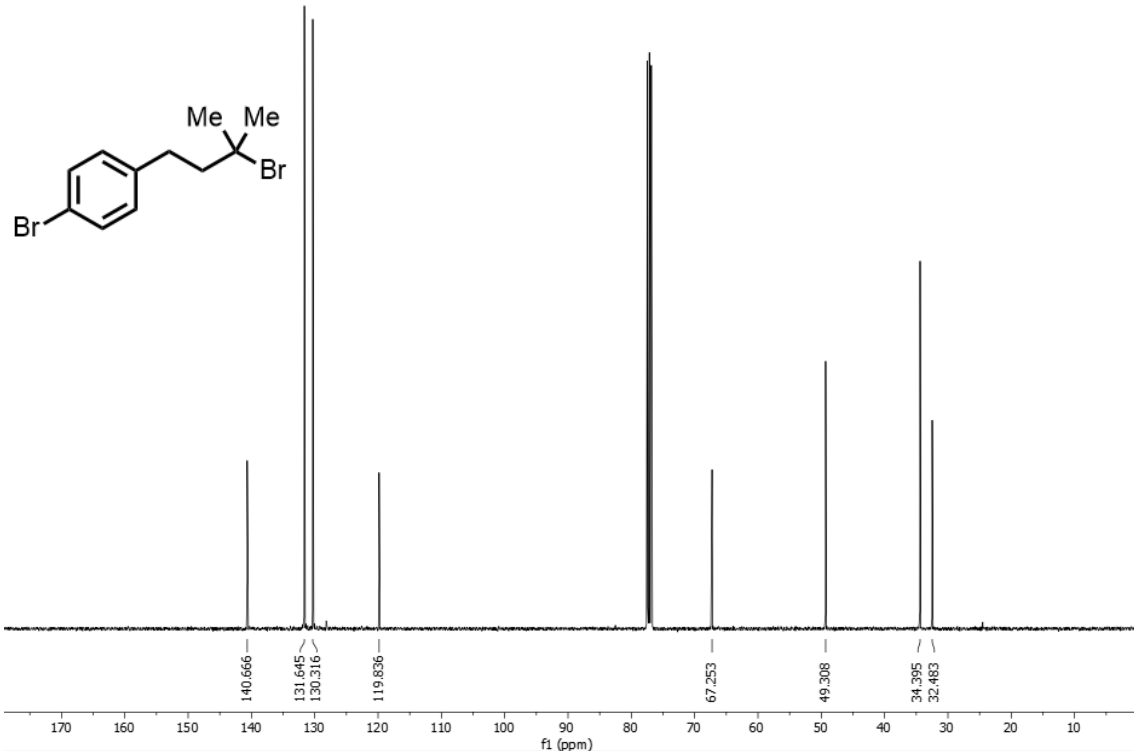

Compound S7. ^{1}H NMR ($CDCl_3$, 400 MHz).

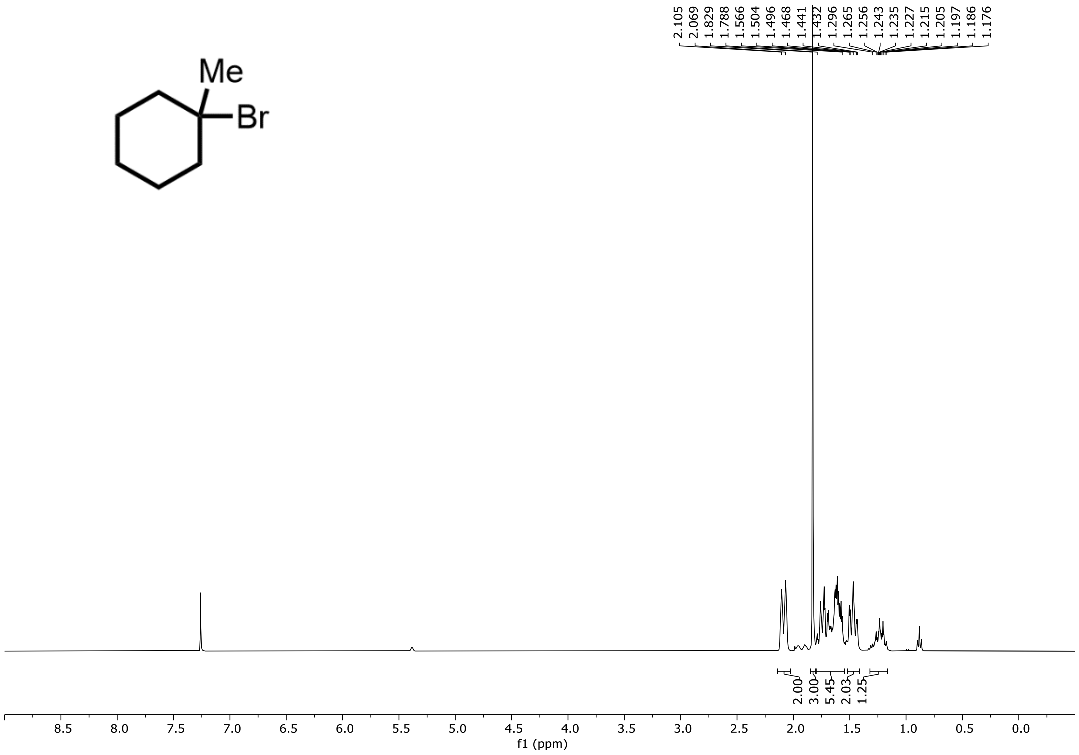

Compound S8. ^{1}H NMR ($CDCl_3$, 400 MHz).

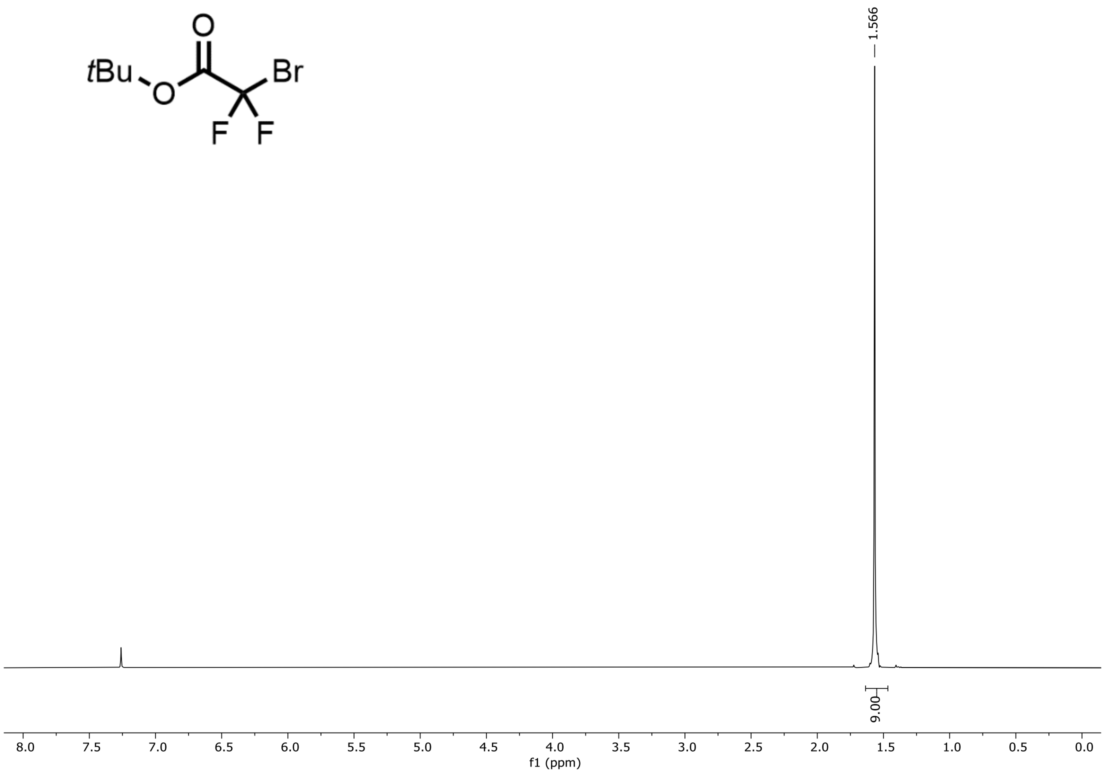

Compound 4. Top: ^{1}H NMR ($CDCl_3$, 400 MHz). Bottom: ^{13}C NMR ($CDCl_3$, 100 MHz)

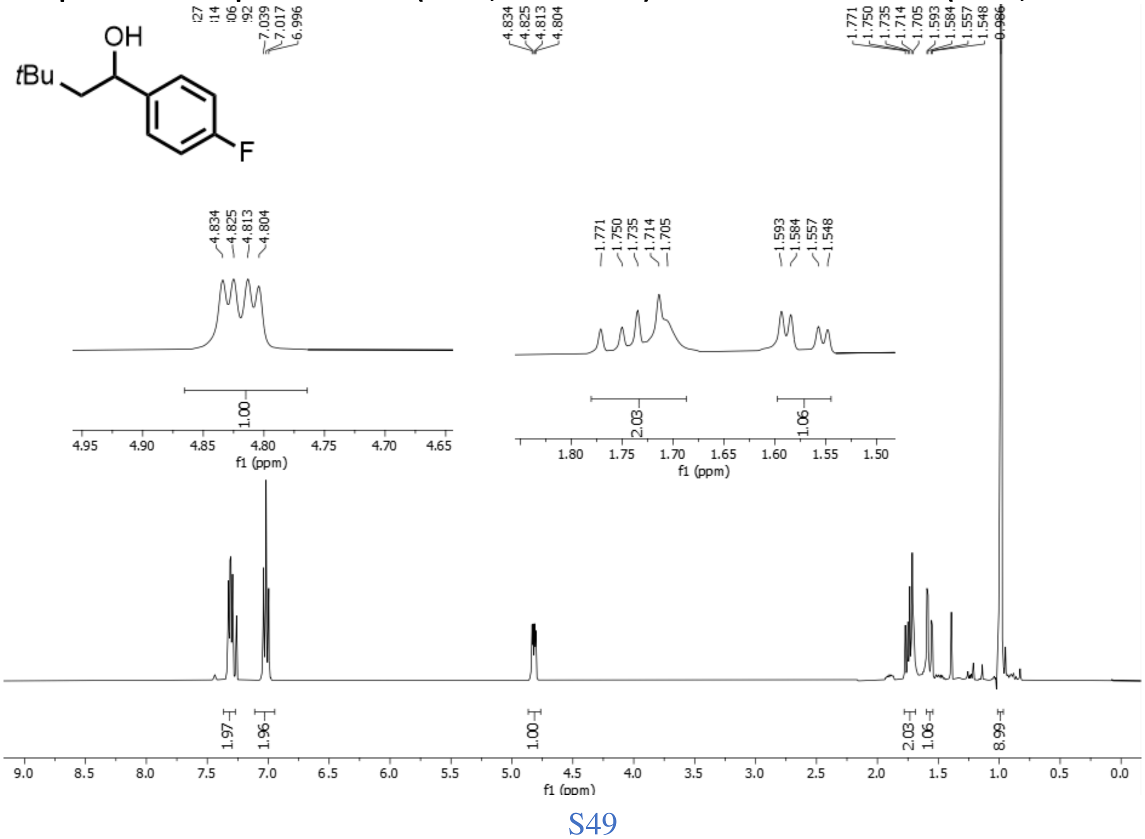

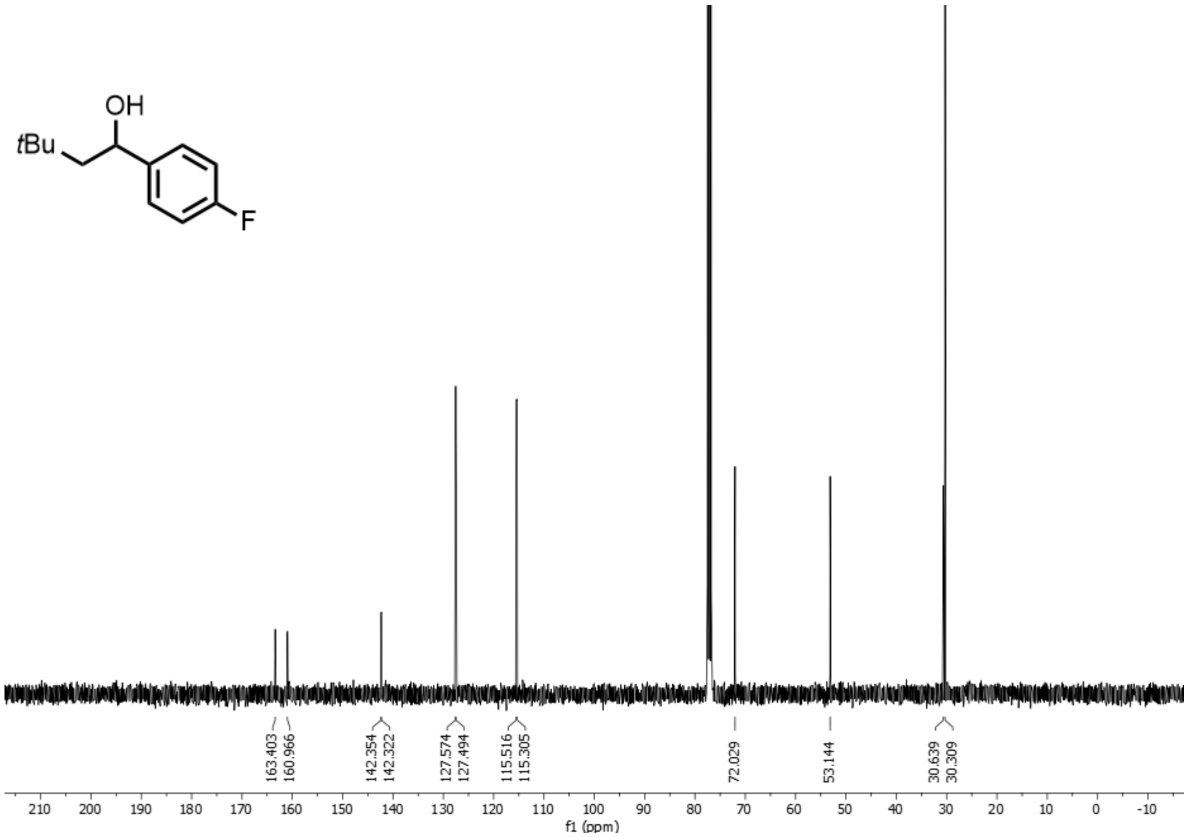

Compound 4. ^{19}F NMR ($CDCl_3$, 376 MHz)

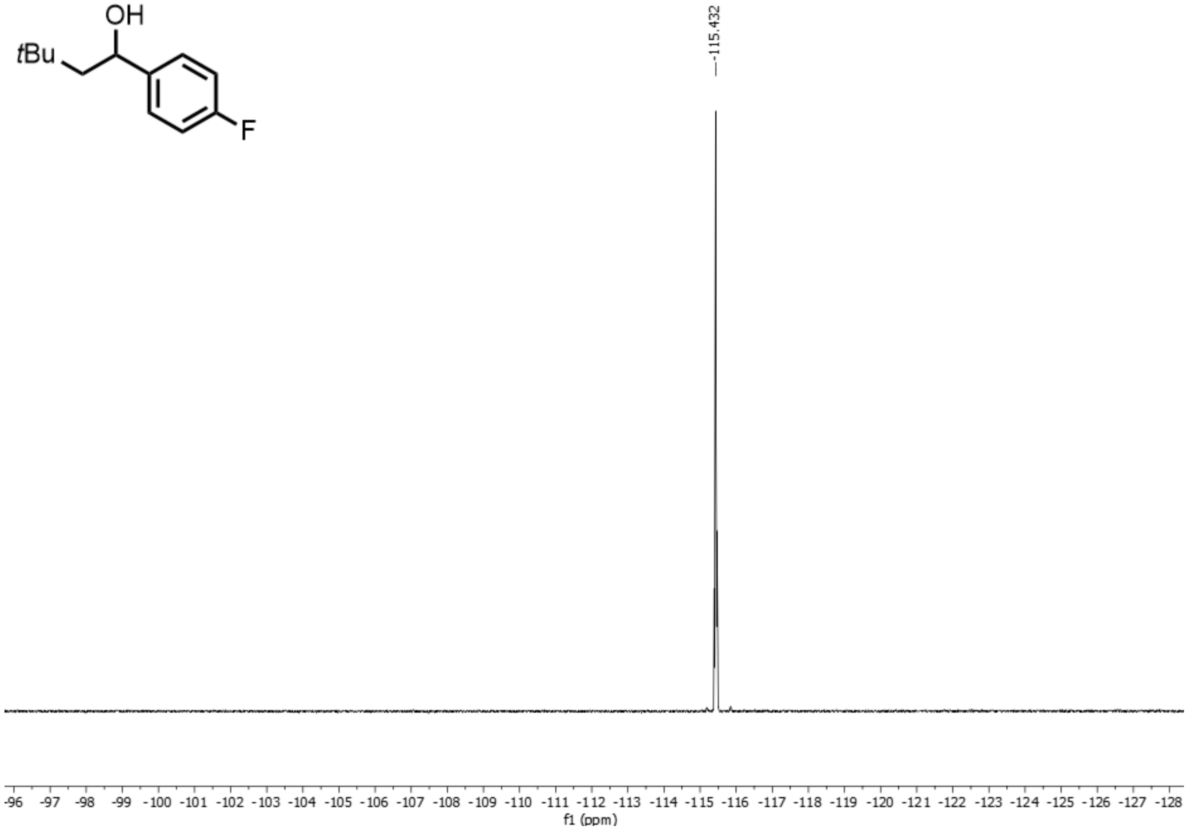

Compound 5. Top: ^{1}H NMR ($CDCl_3$, 400 MHz). Bottom: ^{13}C NMR ($CDCl_3$, 100 MHz)

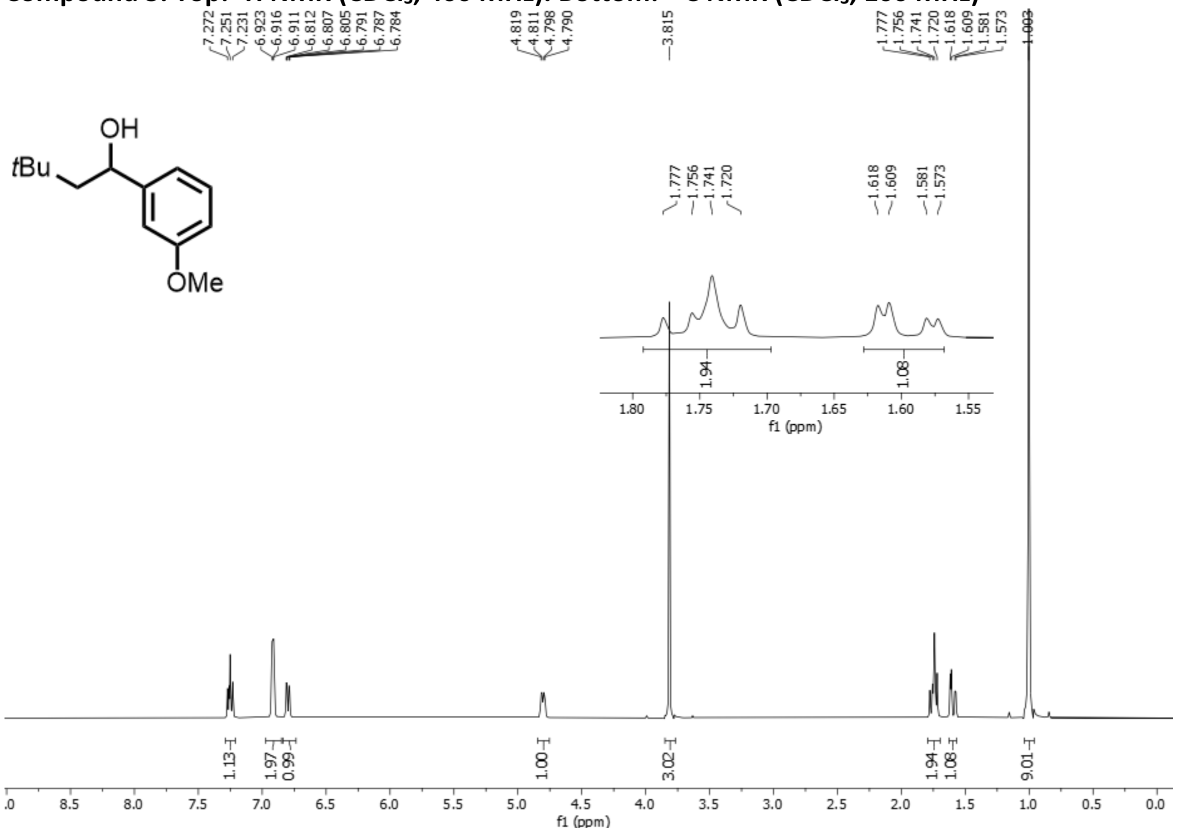

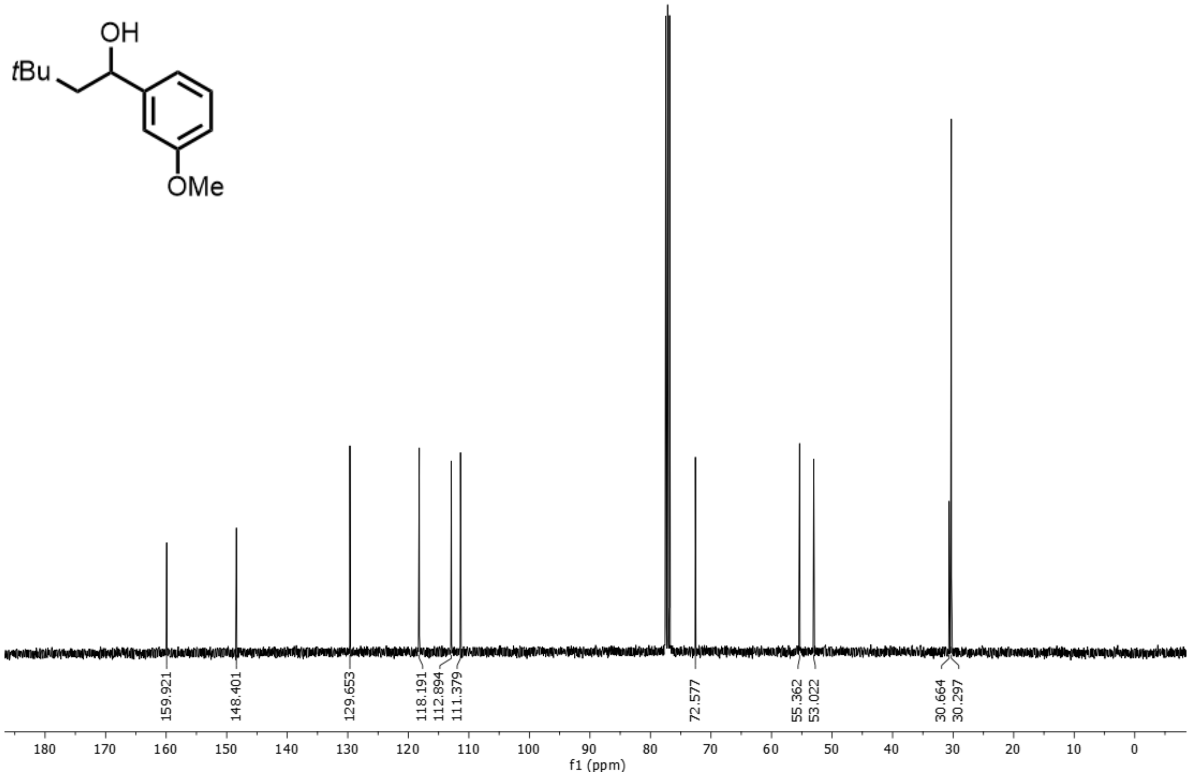

Compound 6. Top: ^{1}H NMR ($CDCl_3$, 400 MHz). Bottom: ^{13}C NMR ($CDCl_3$, 100 MHz)

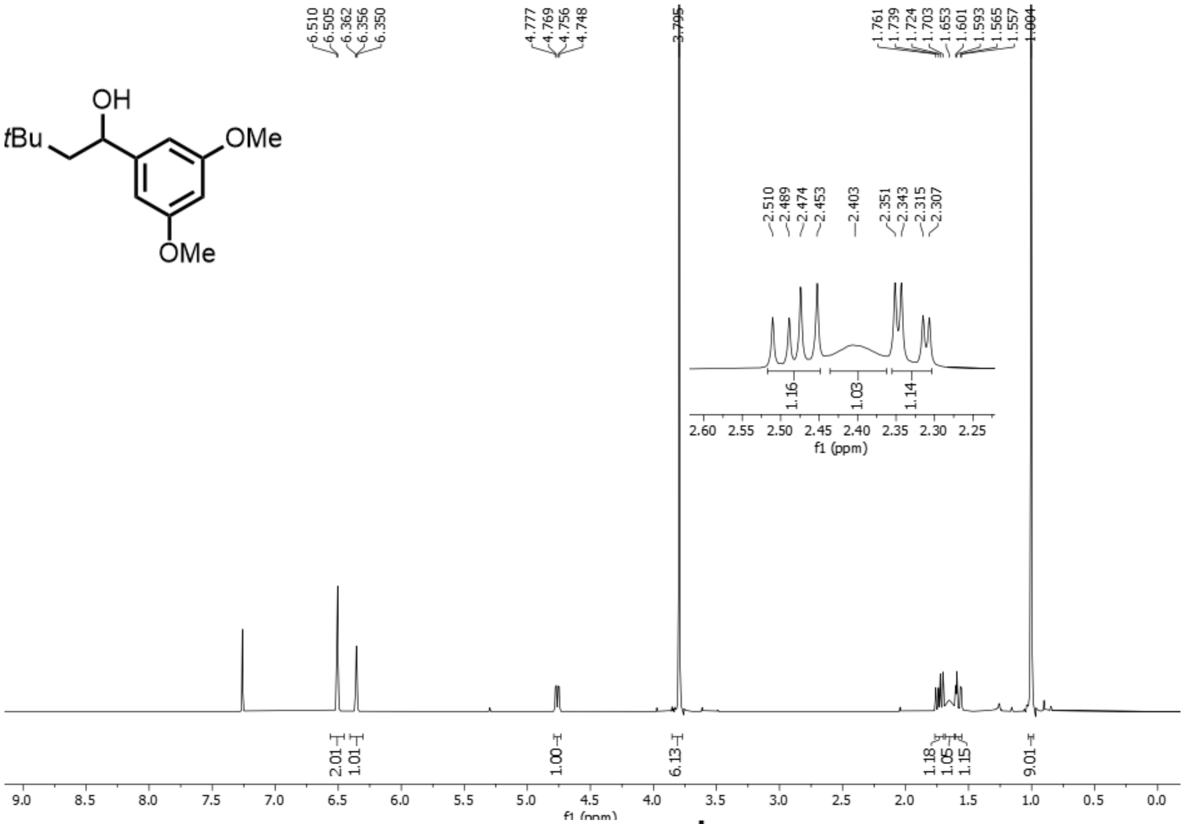

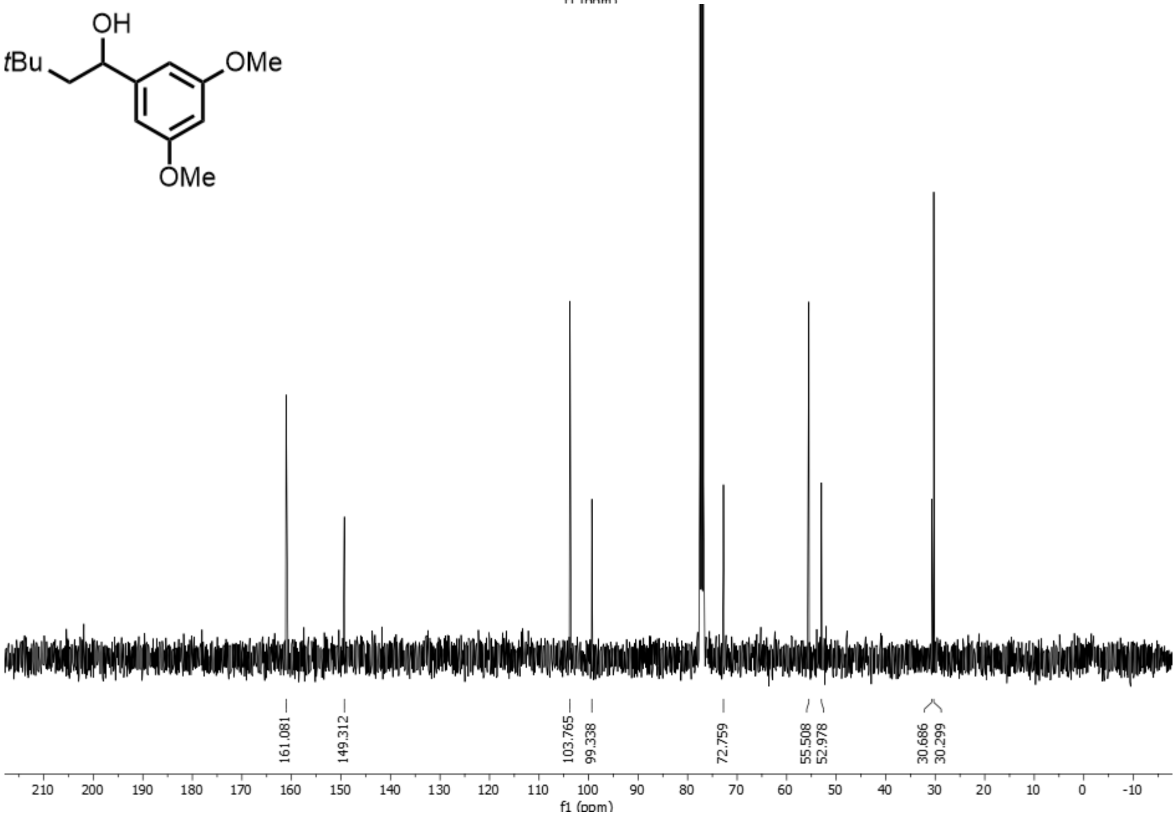

Compound 7. ^{1}H NMR ($CDCl_3$, 400 MHz).

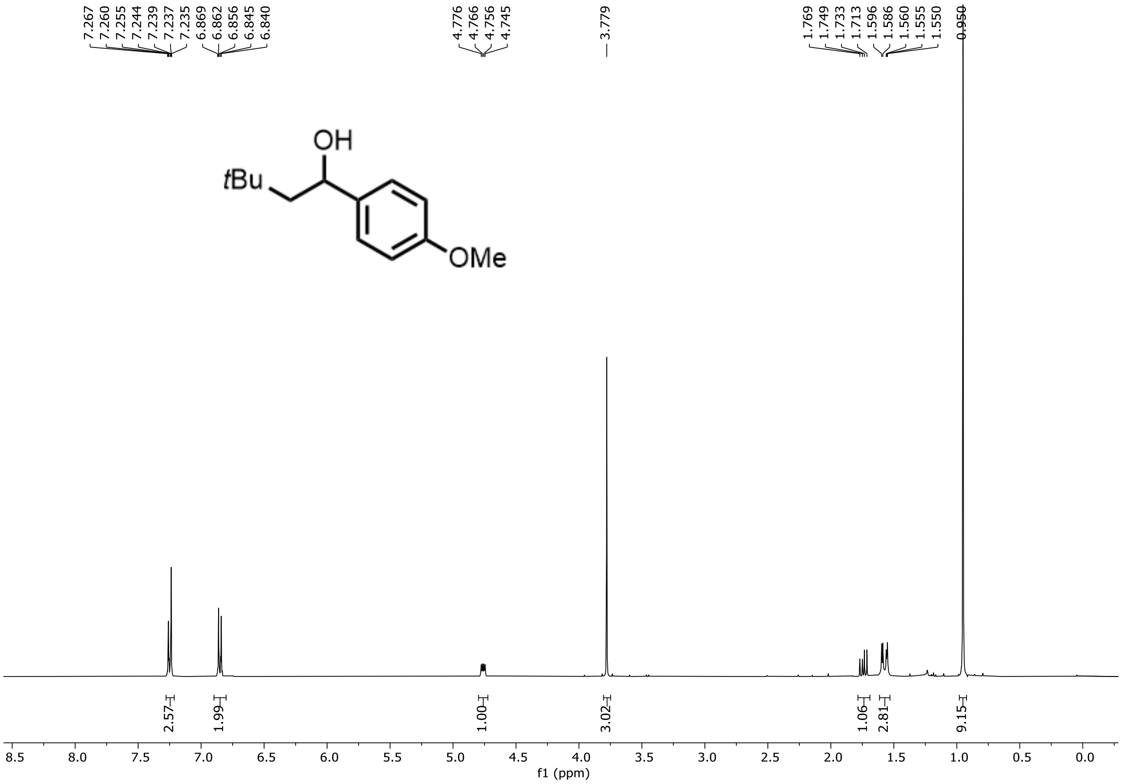

Compound 8. Top: ^{1}H NMR ($CDCl_3$, 400 MHz). Bottom: ^{13}C NMR ($CDCl_3$, 100 MHz)

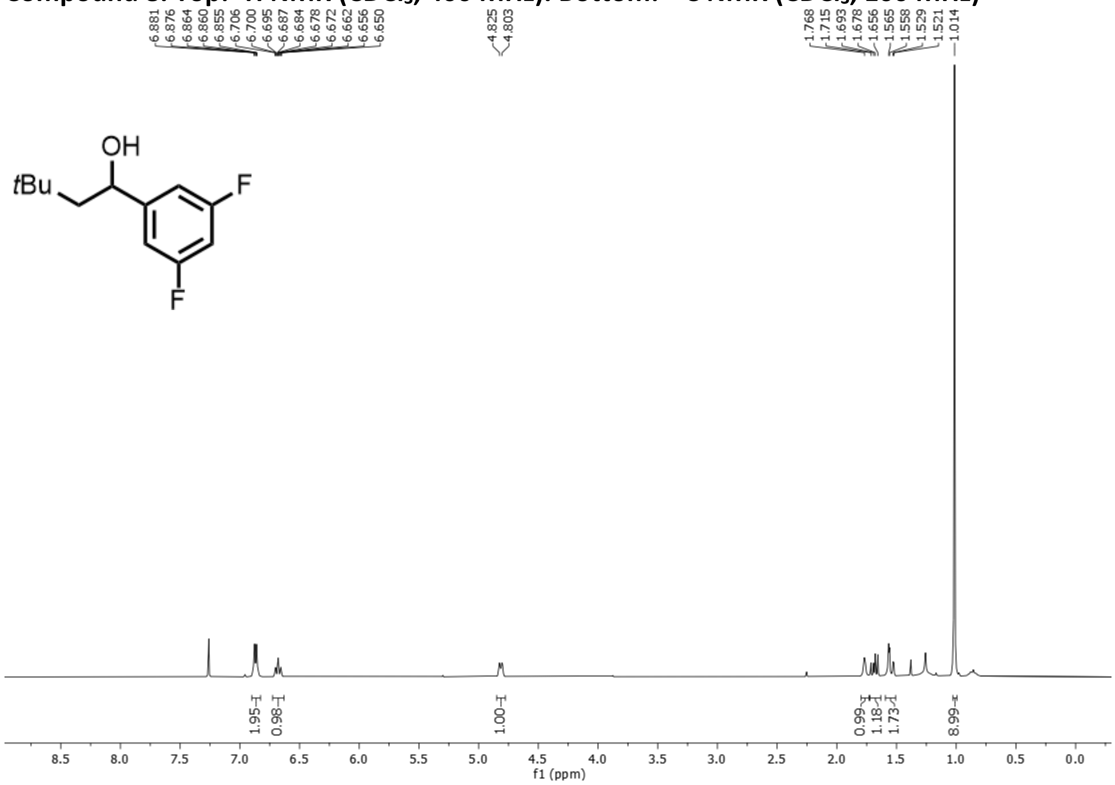

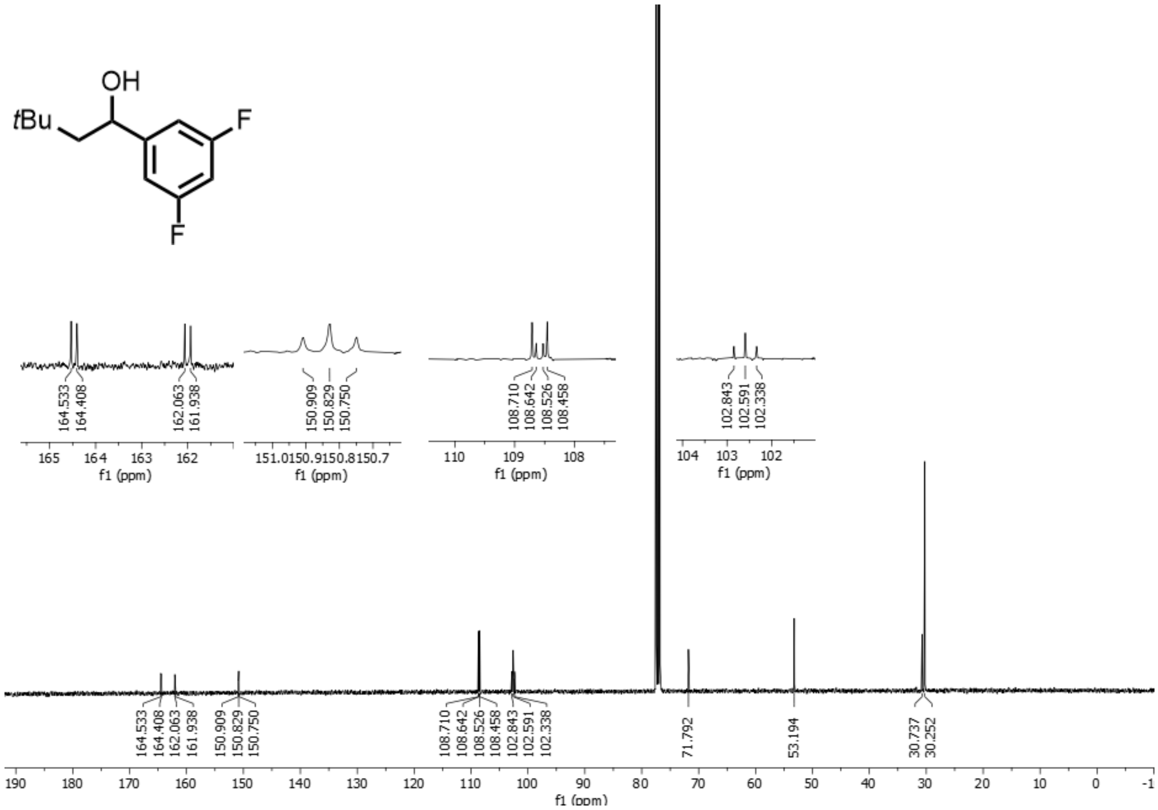

Compound 8. ^{19}F NMR ($CDCl_3$, 376 MHz)

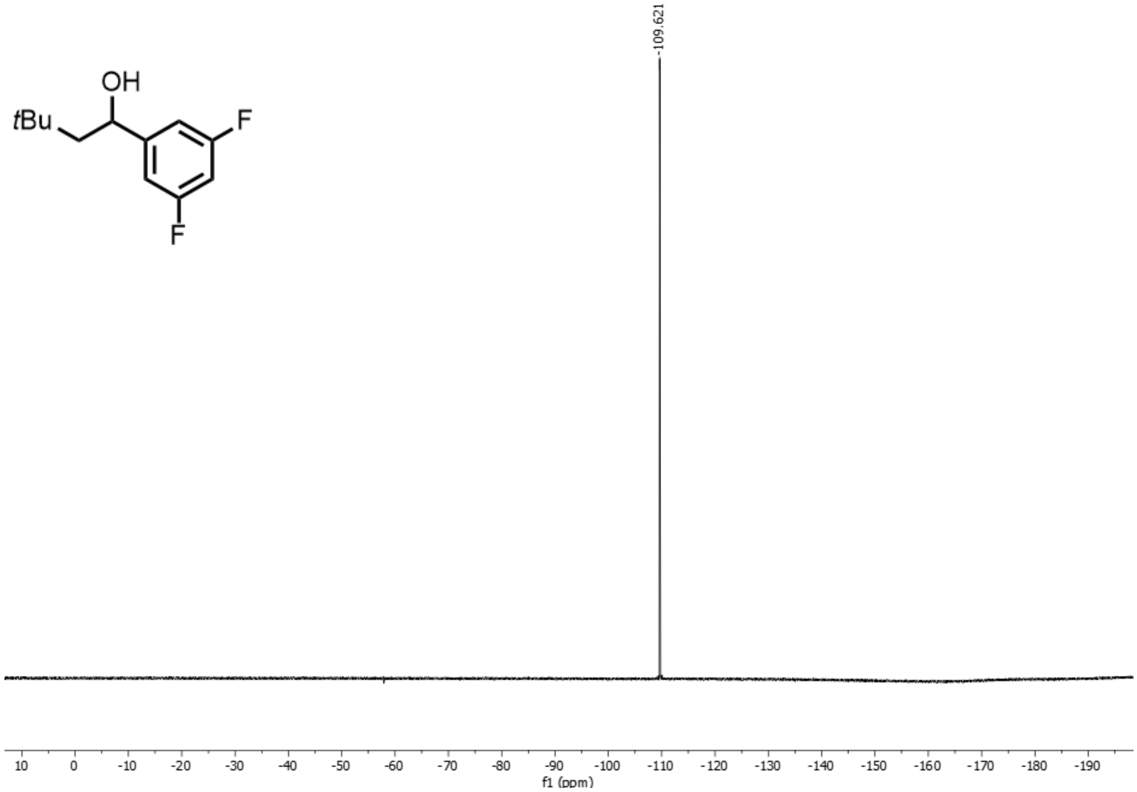

Compound 9. Top: ^{1}H NMR ($CDCl_3$, 400 MHz). Bottom: ^{13}C NMR ($CDCl_3$, 100 MHz)

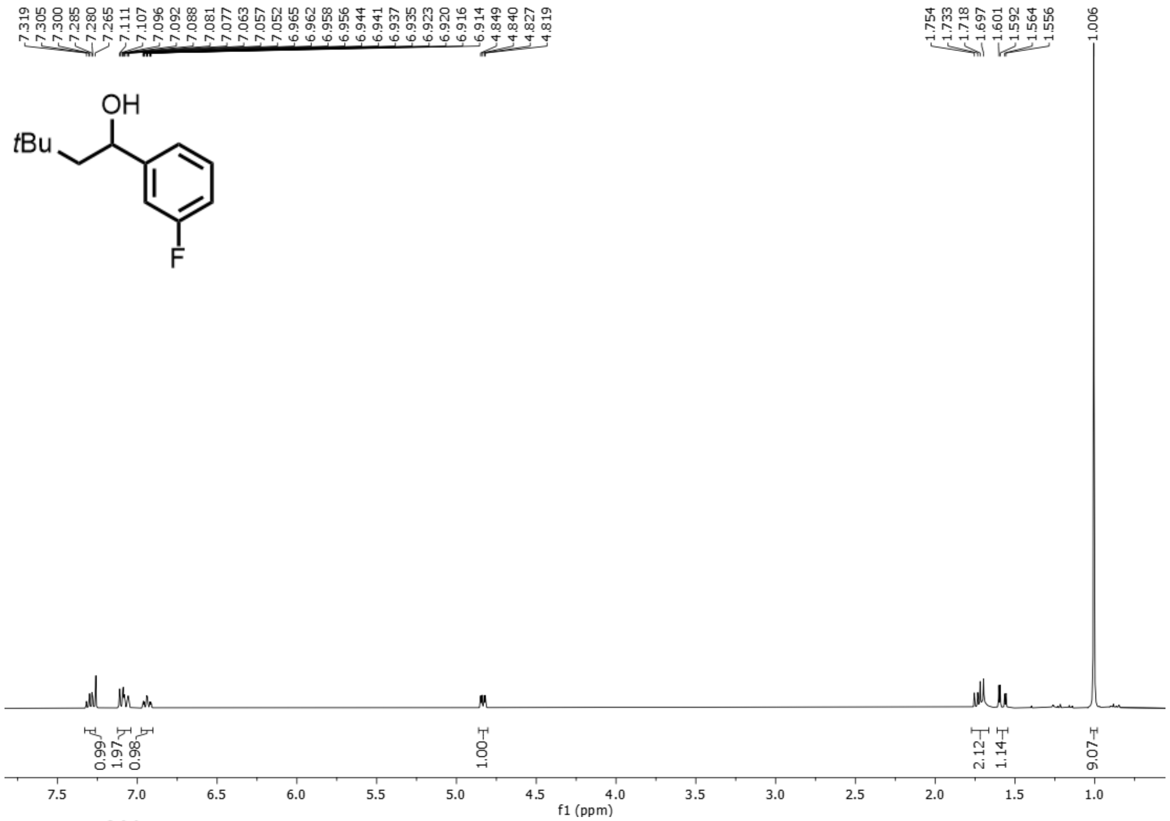

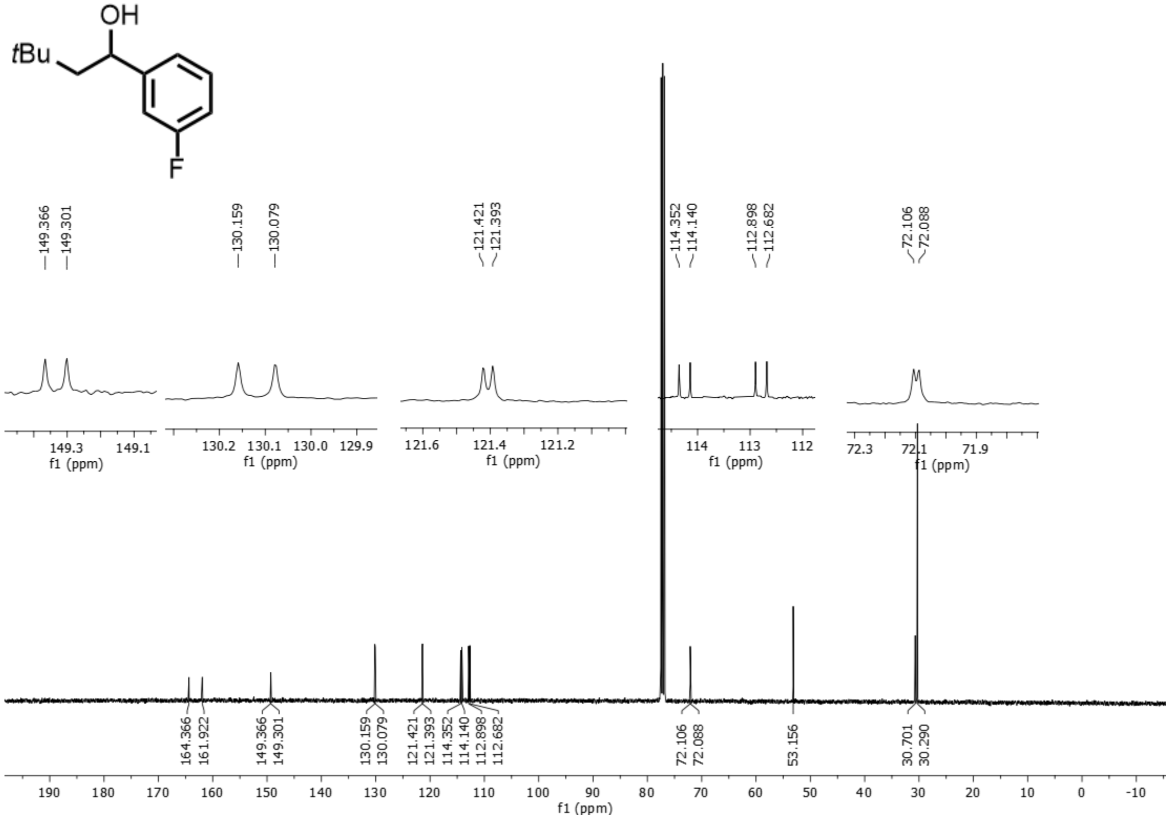

Compound 9. ^{19}F NMR ($CDCl_3$, 376 MHz)

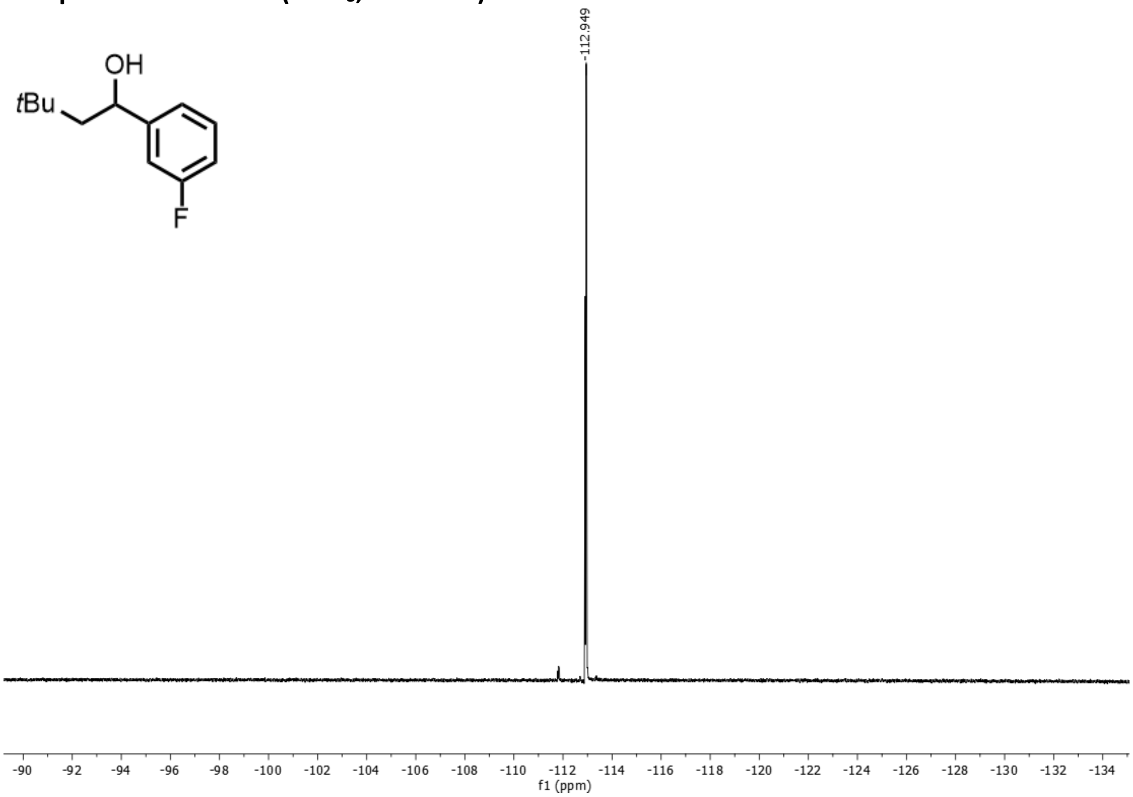

Compound 10. ^{1}H NMR ($CDCl_3$, 400 MHz).

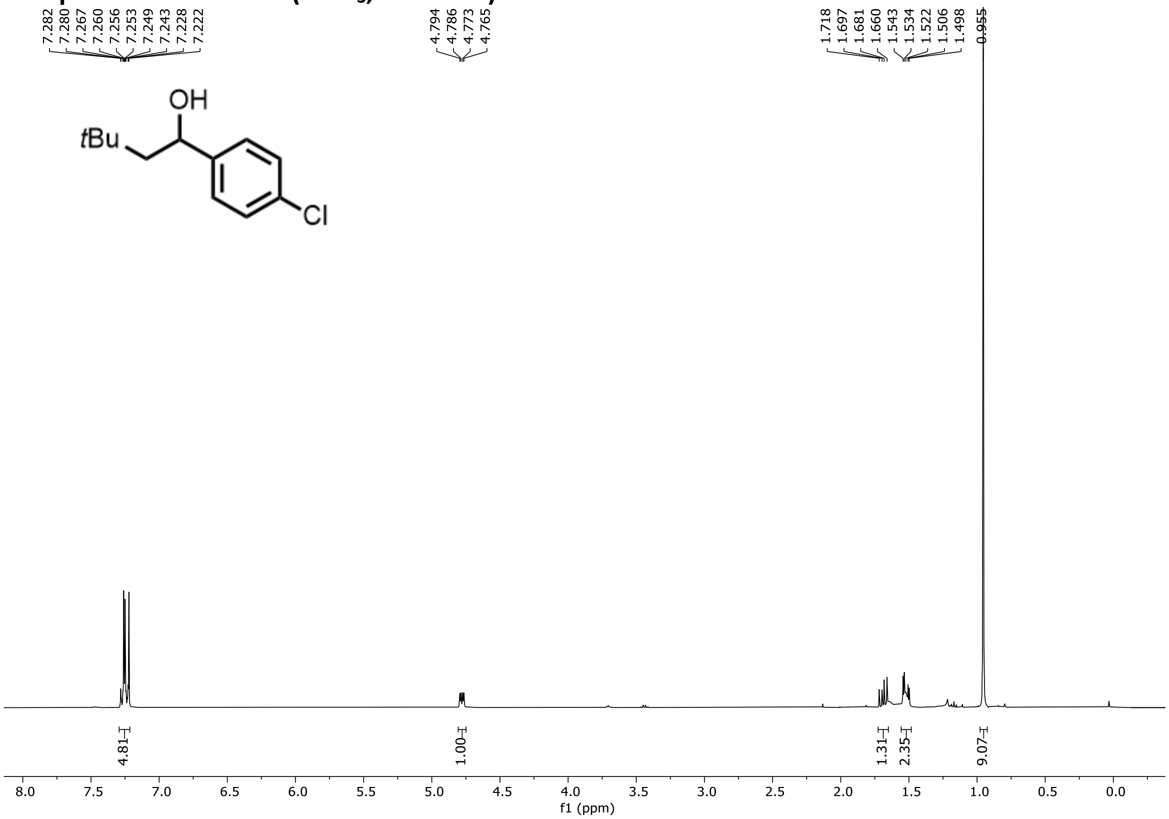

Compound 11. Top: ^{1}H NMR ($CDCl_3$, 400 MHz). Bottom: ^{13}C NMR ($CDCl_3$, 100 MHz)

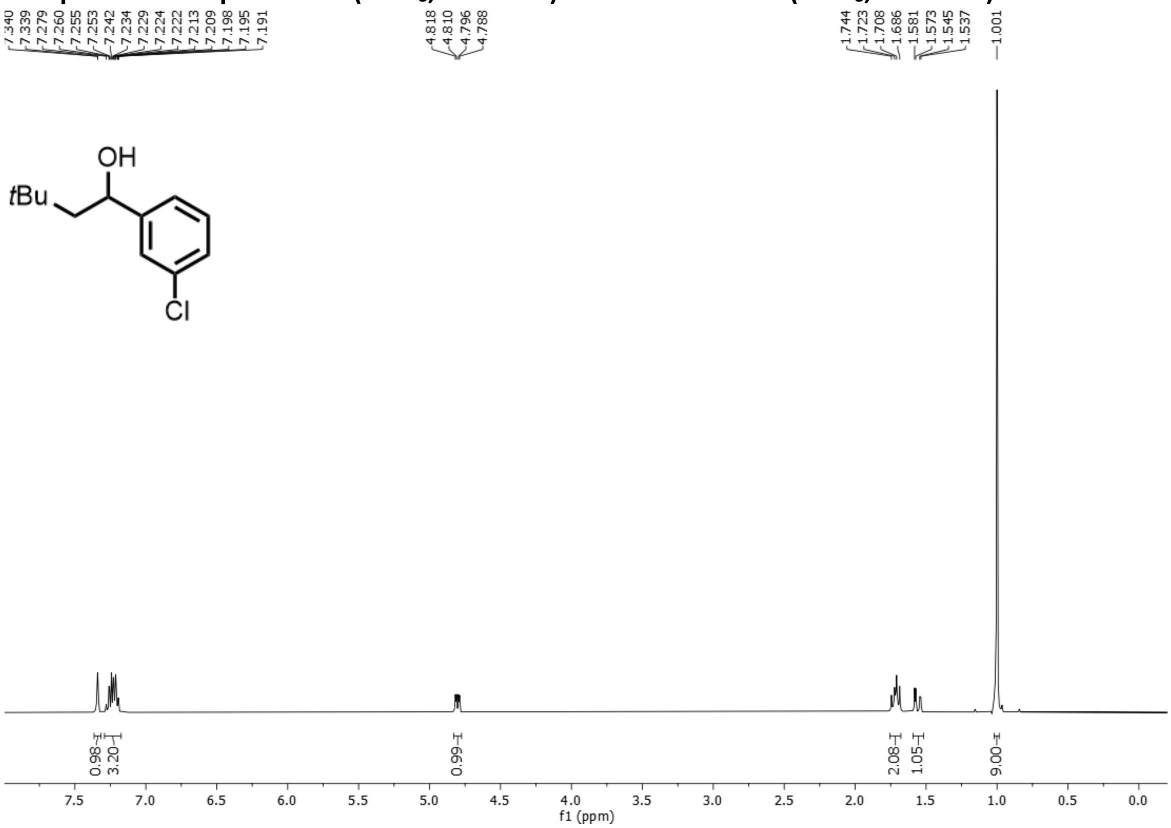

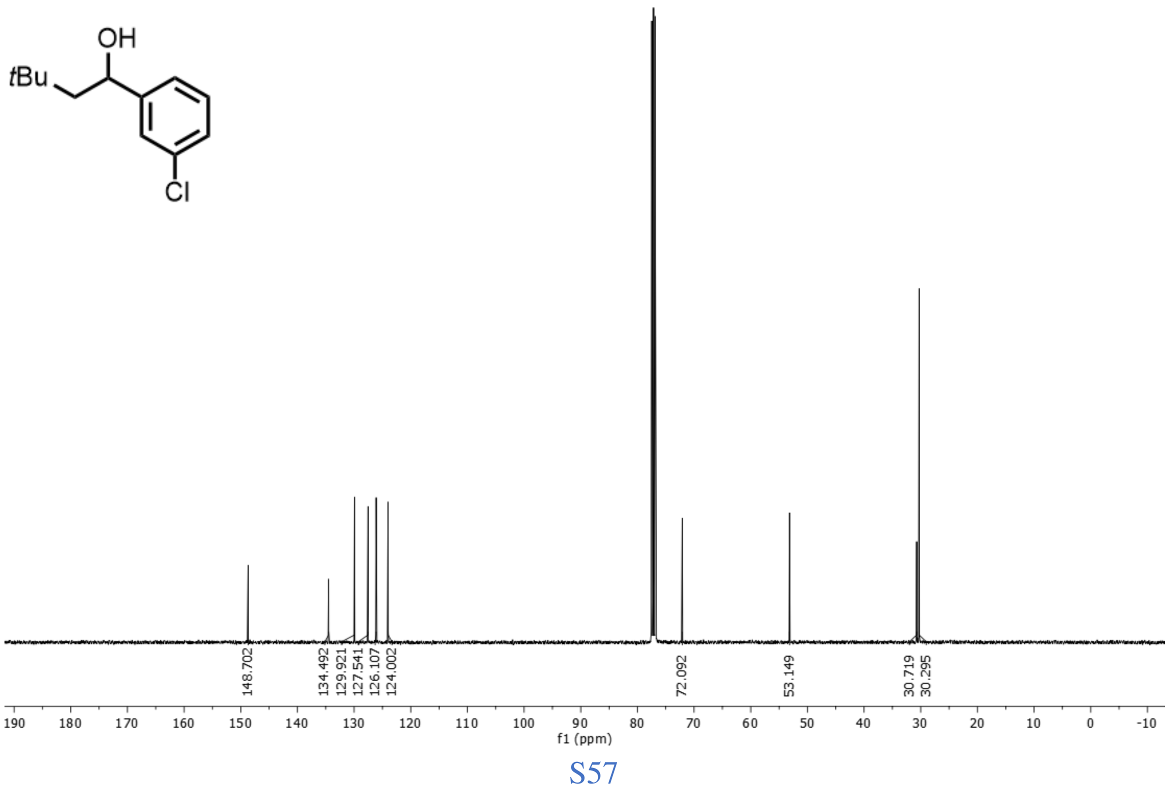

Compound 12. ^1H NMR ($CDCl_3$, 400 MHz).

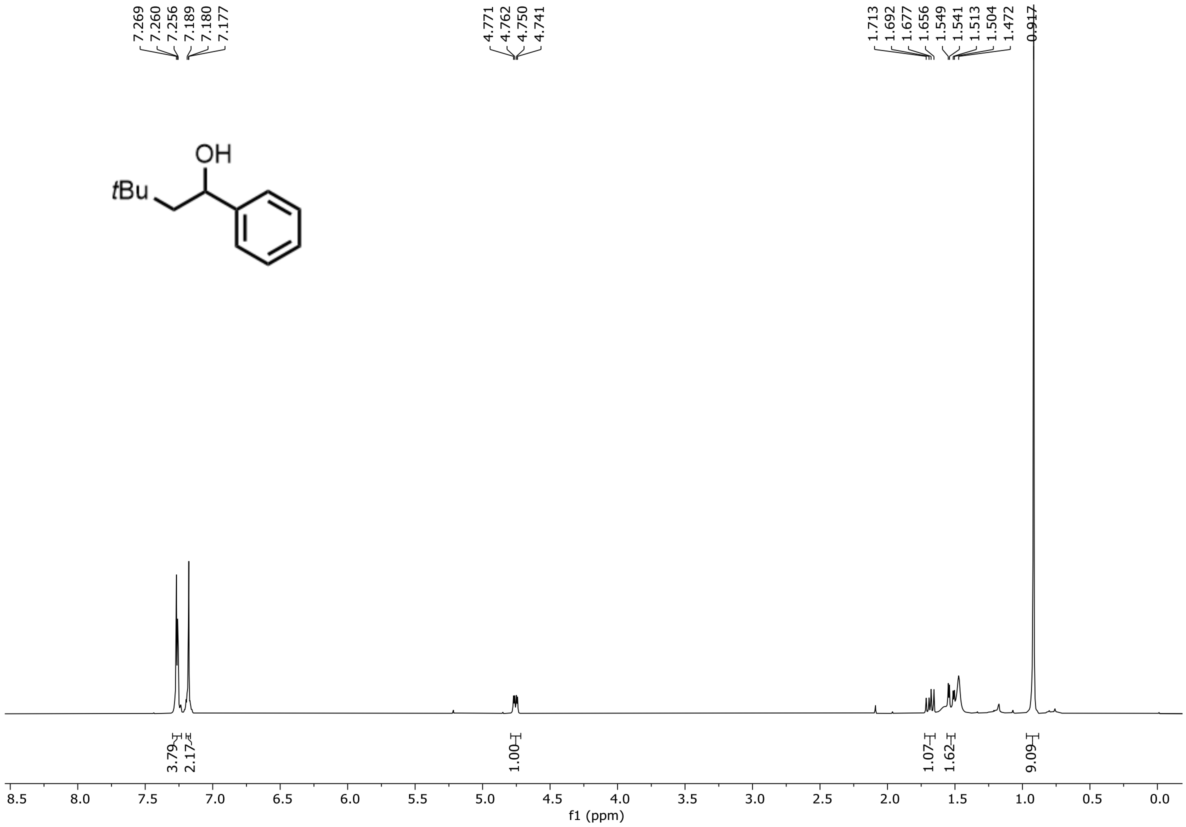

Compound 13. Top: ^{1}H NMR ($CDCl_3$, 400 MHz). Bottom: ^{13}C NMR ($CDCl_3$, 100 MHz)

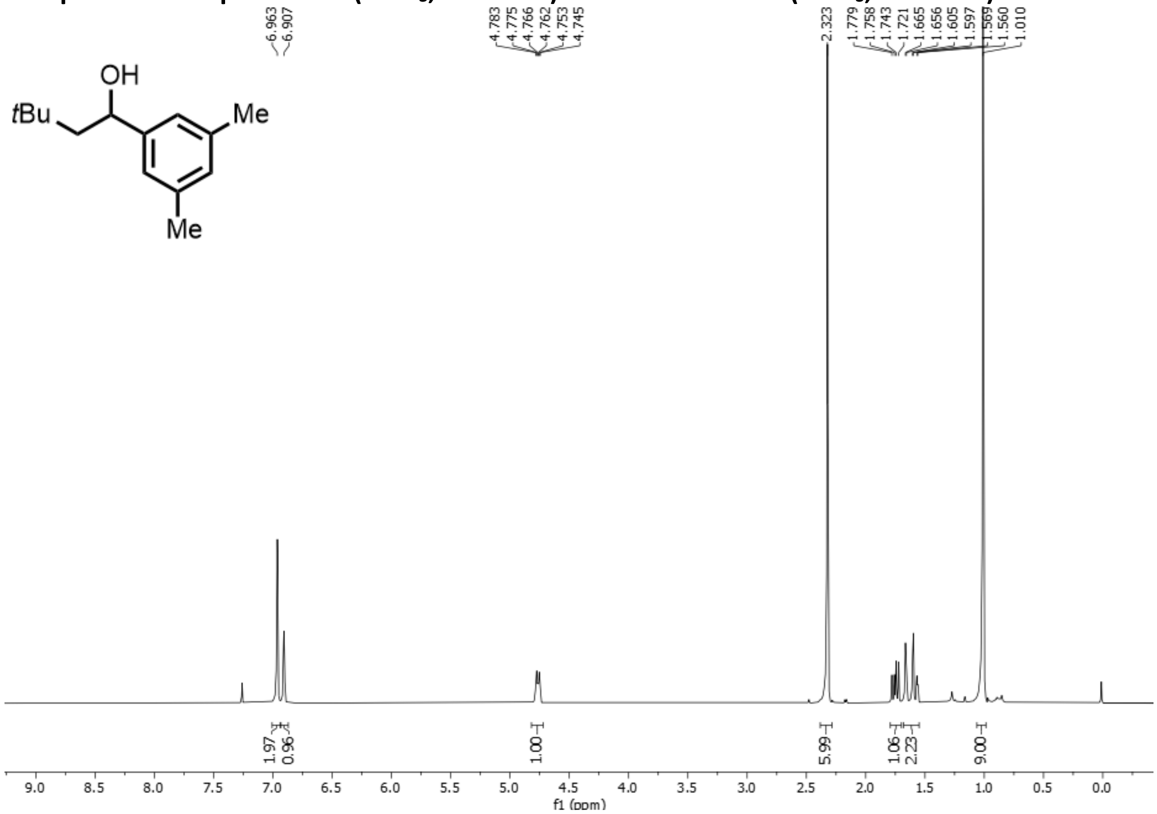

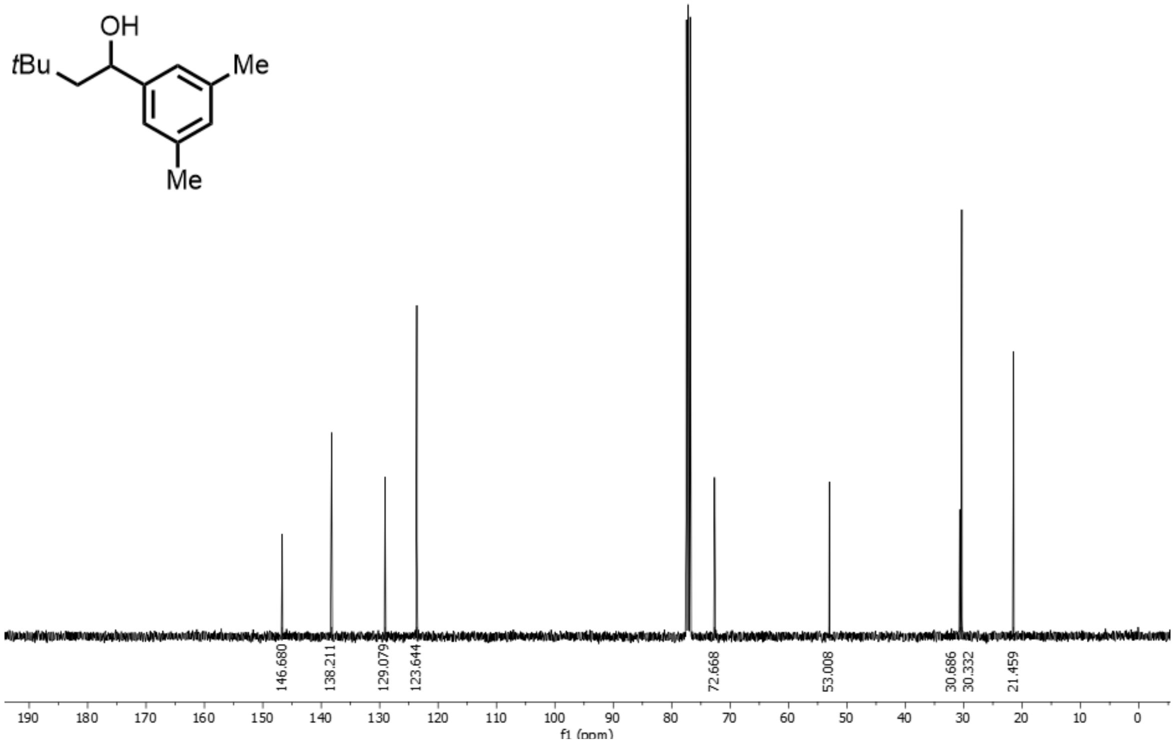

Compound 14. Top: ^{1}H NMR ($CDCl_3$, 400 MHz). Bottom: ^{13}C NMR ($CDCl_3$, 100 MHz)

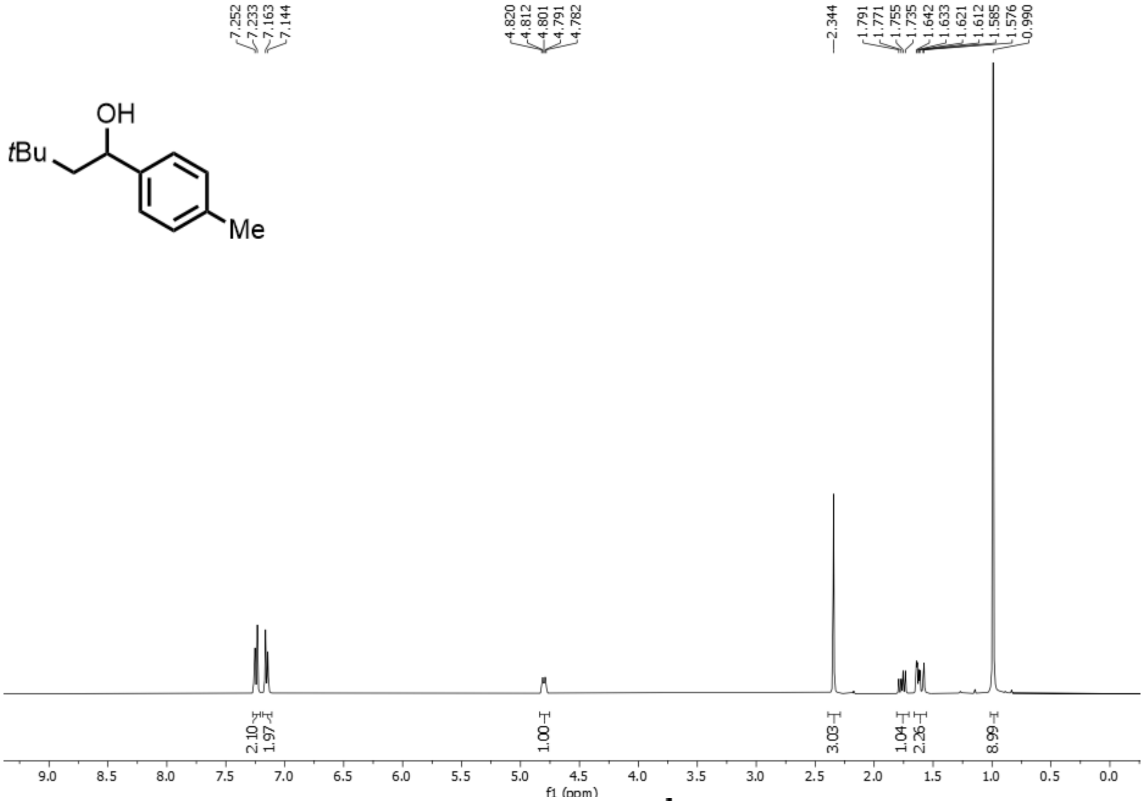

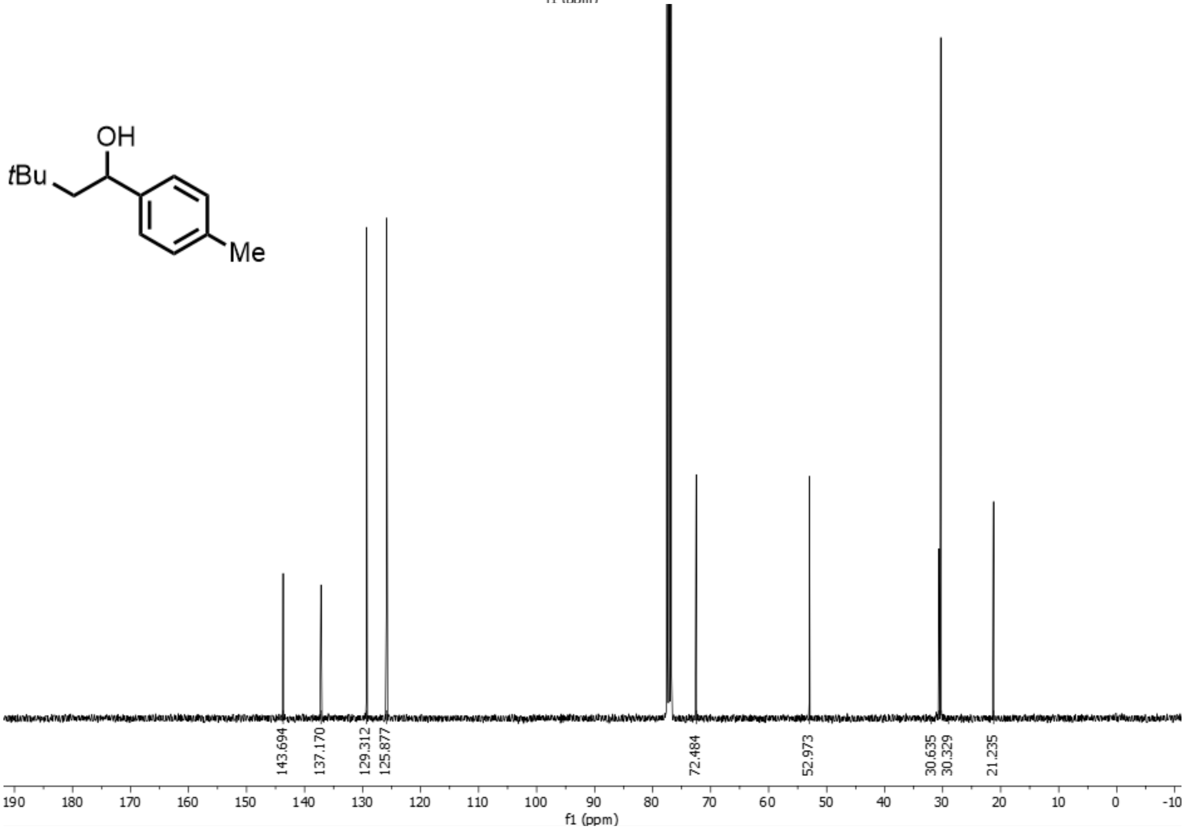

Compound 15. Top: ^{1}H NMR ($CDCl_3$, 400 MHz). Bottom: ^{13}C NMR ($CDCl_3$, 100 MHz)

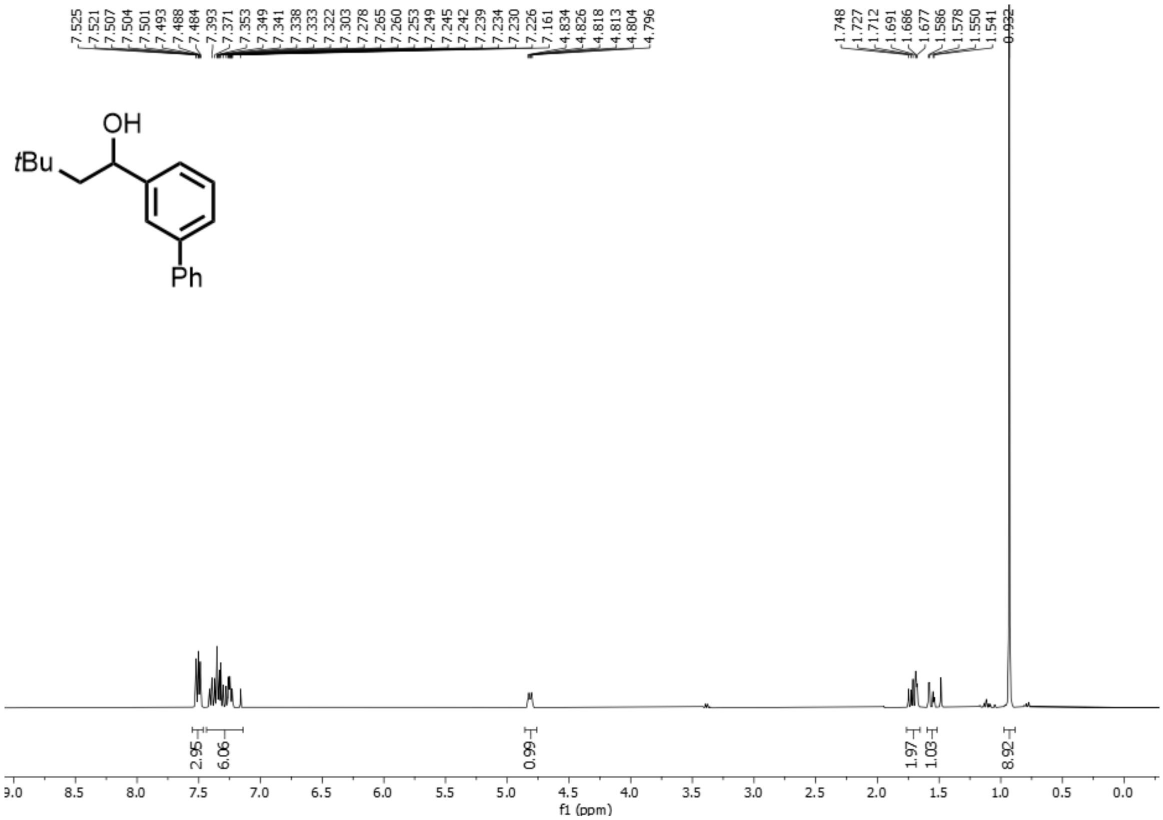

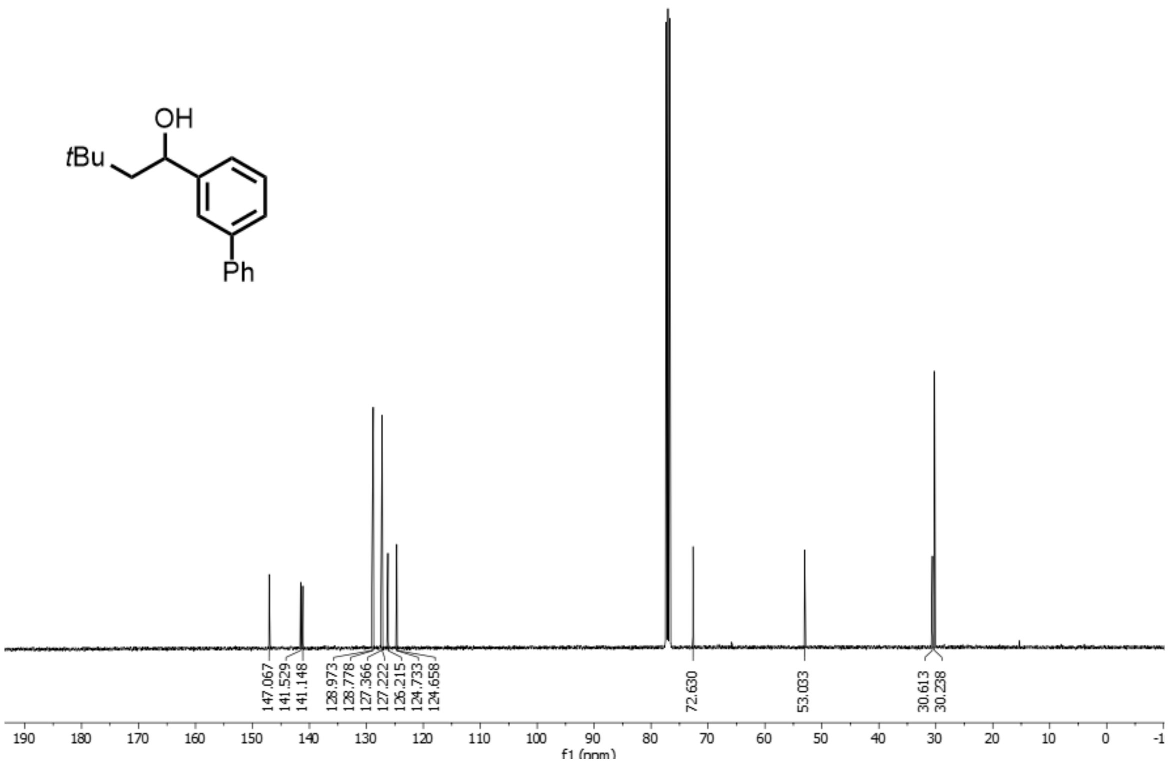

Compound 16. ^{1}H NMR ($CDCl_3$, 400 MHz).

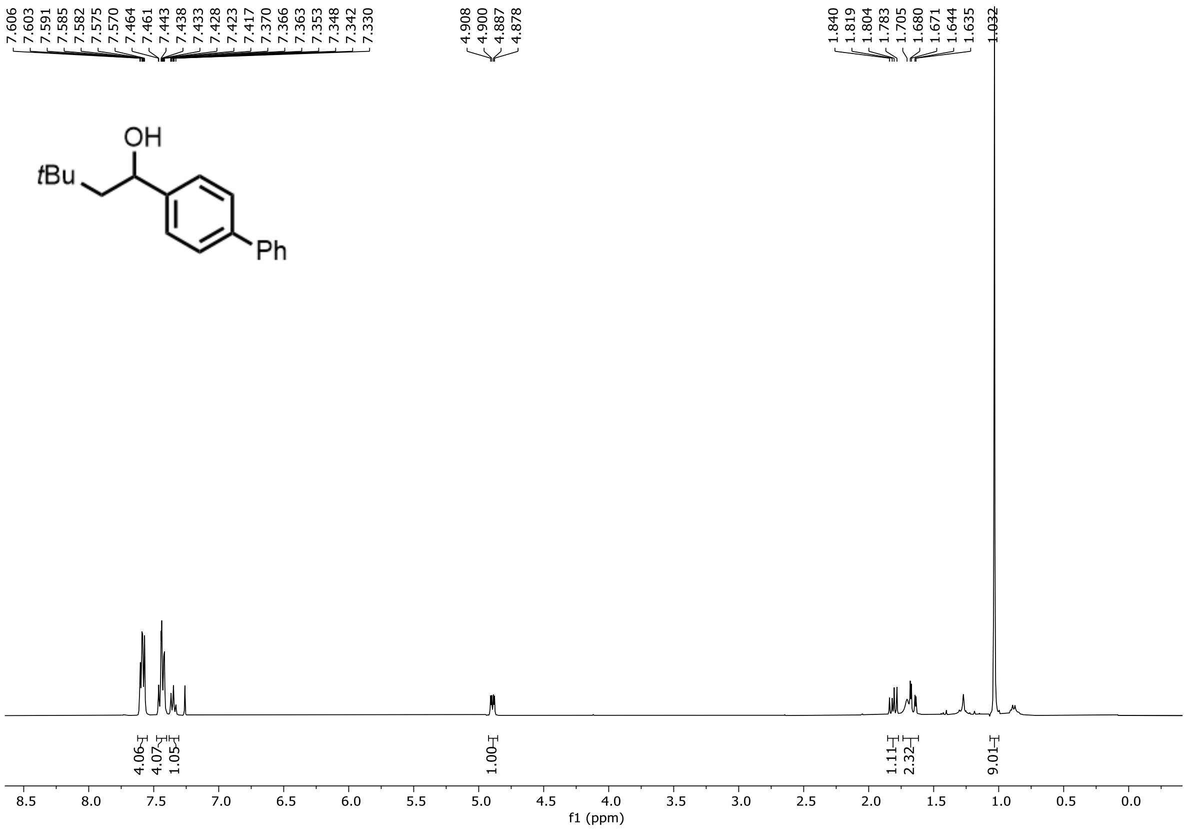

Compound 17. Top: ^{1}H NMR ($CDCl_3$, 400 MHz). Bottom: ^{13}C NMR ($CDCl_3$, 100 MHz)

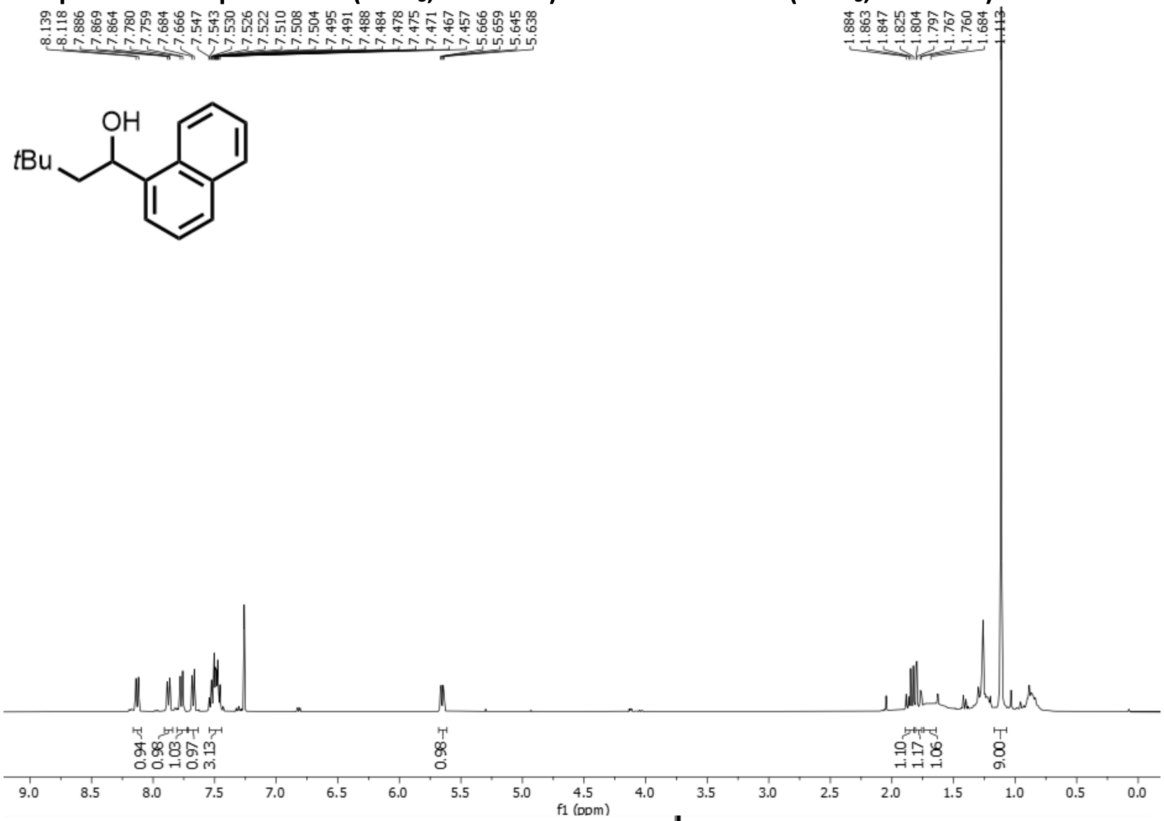

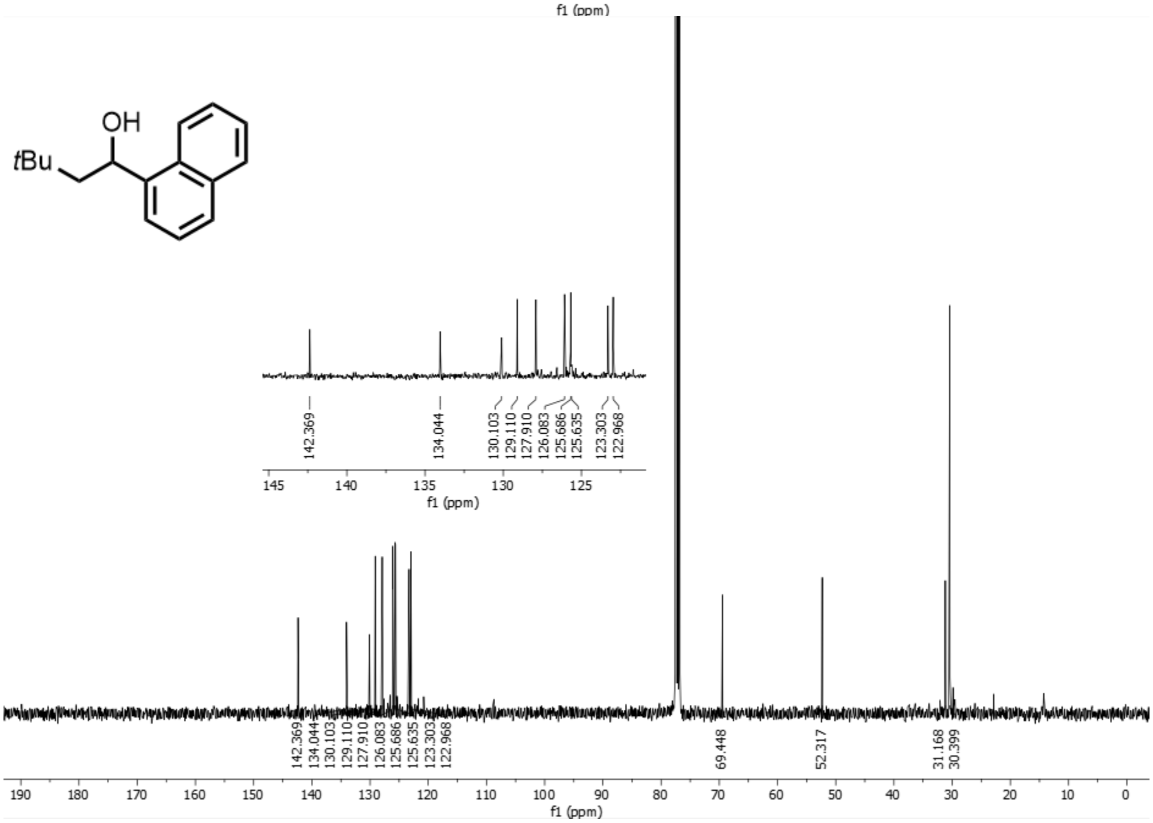

Compound 18. Top: ^{1}H NMR ($CDCl_3$, 400 MHz). Bottom: ^{13}C NMR ($CDCl_3$, 100 MHz)

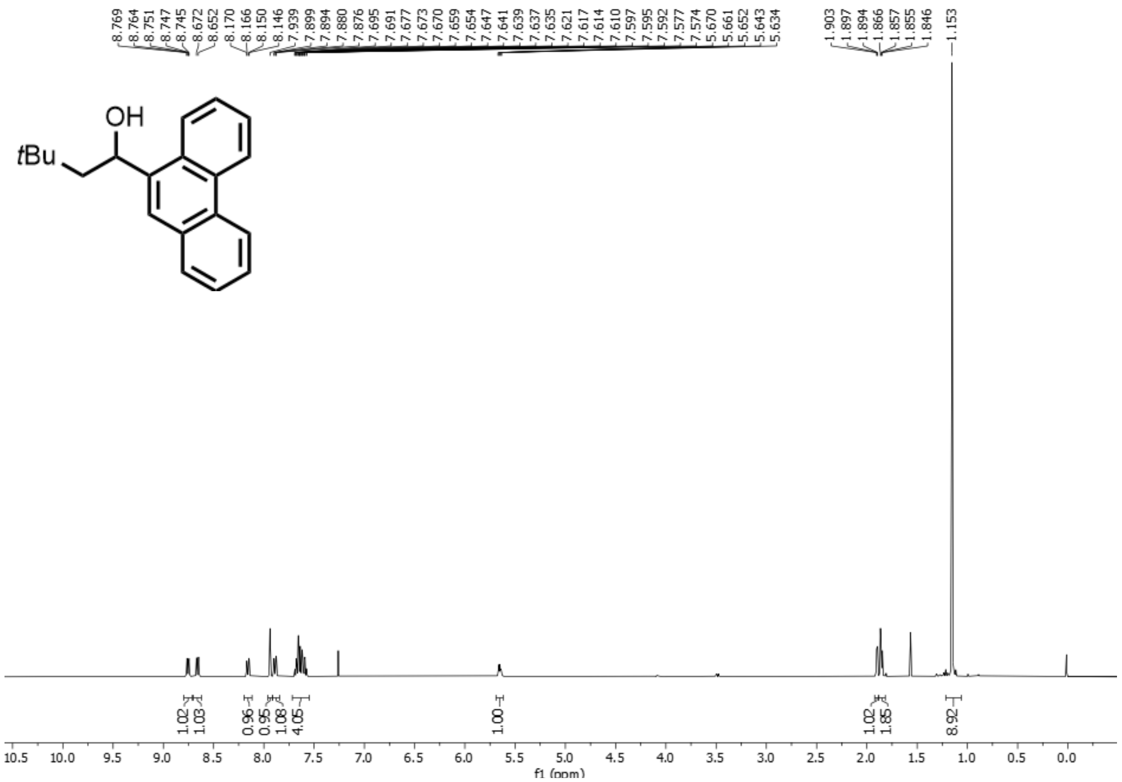

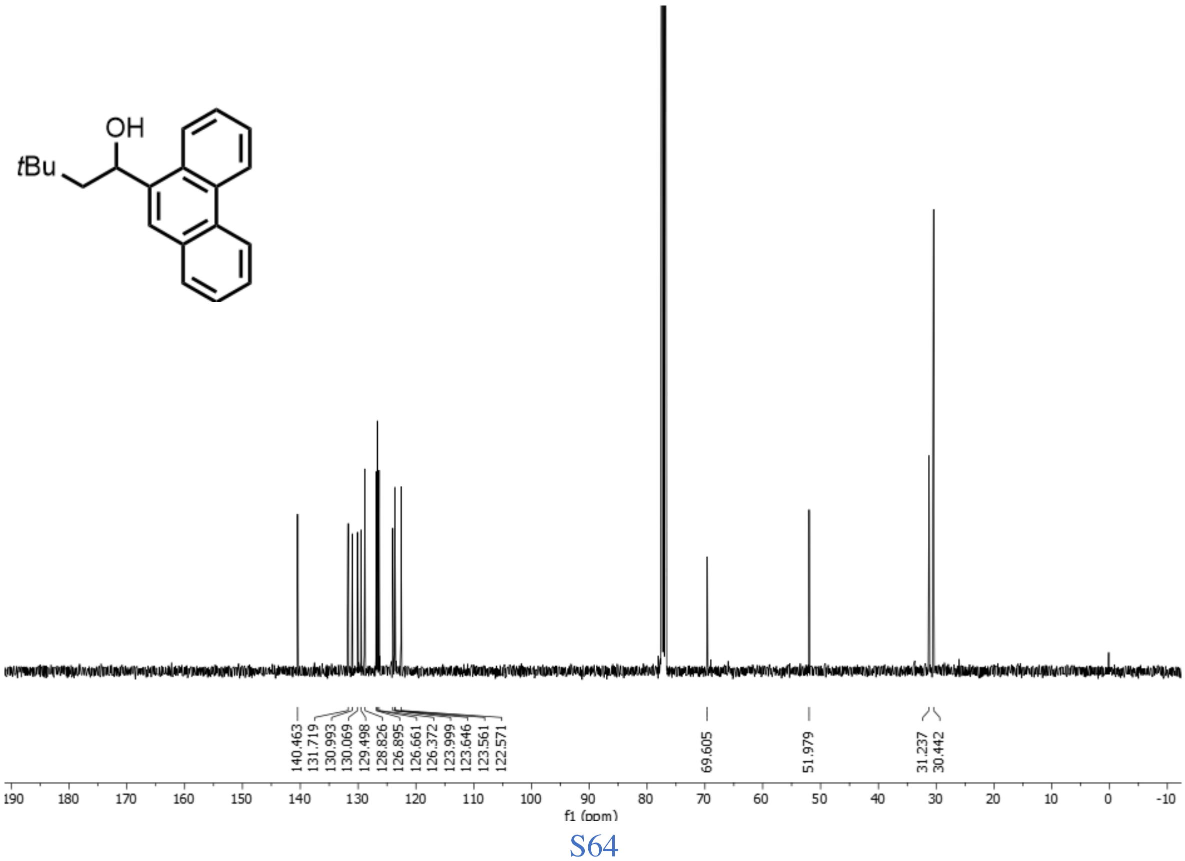

Compound 19. Top: ^{1}H NMR ($CDCl_3$, 400 MHz). Bottom: ^{13}C NMR ($CDCl_3$, 100 MHz)

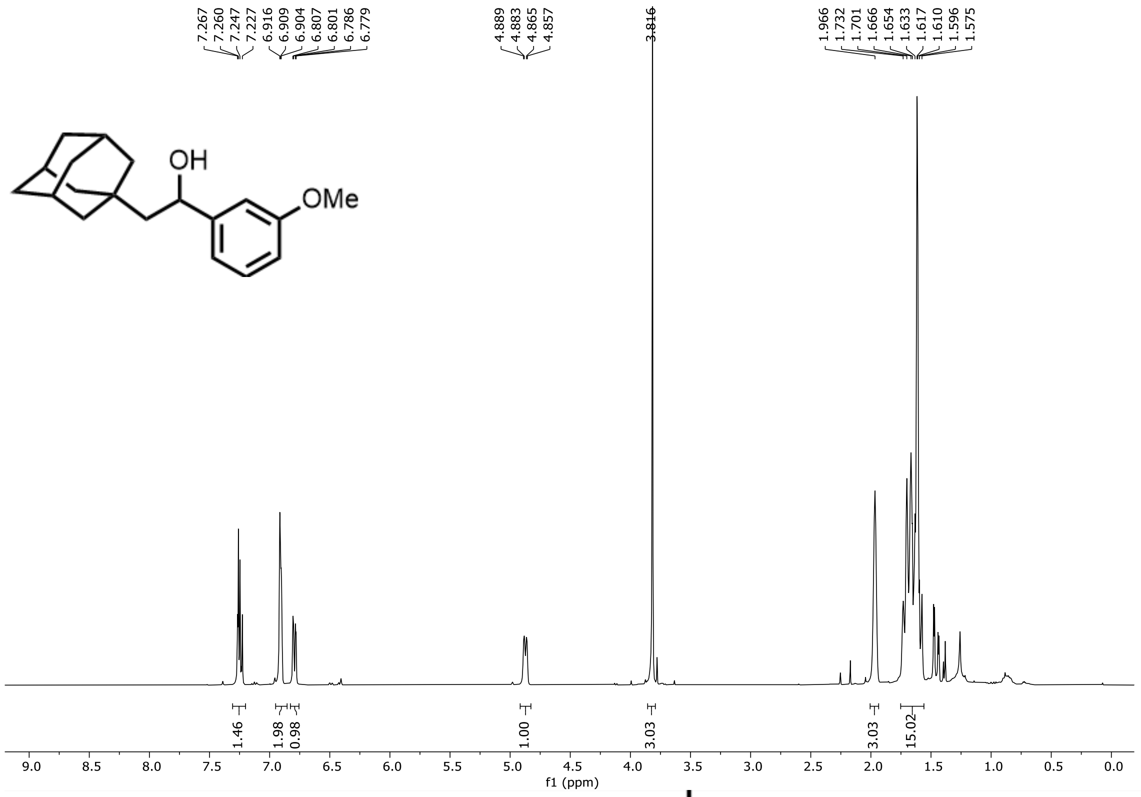

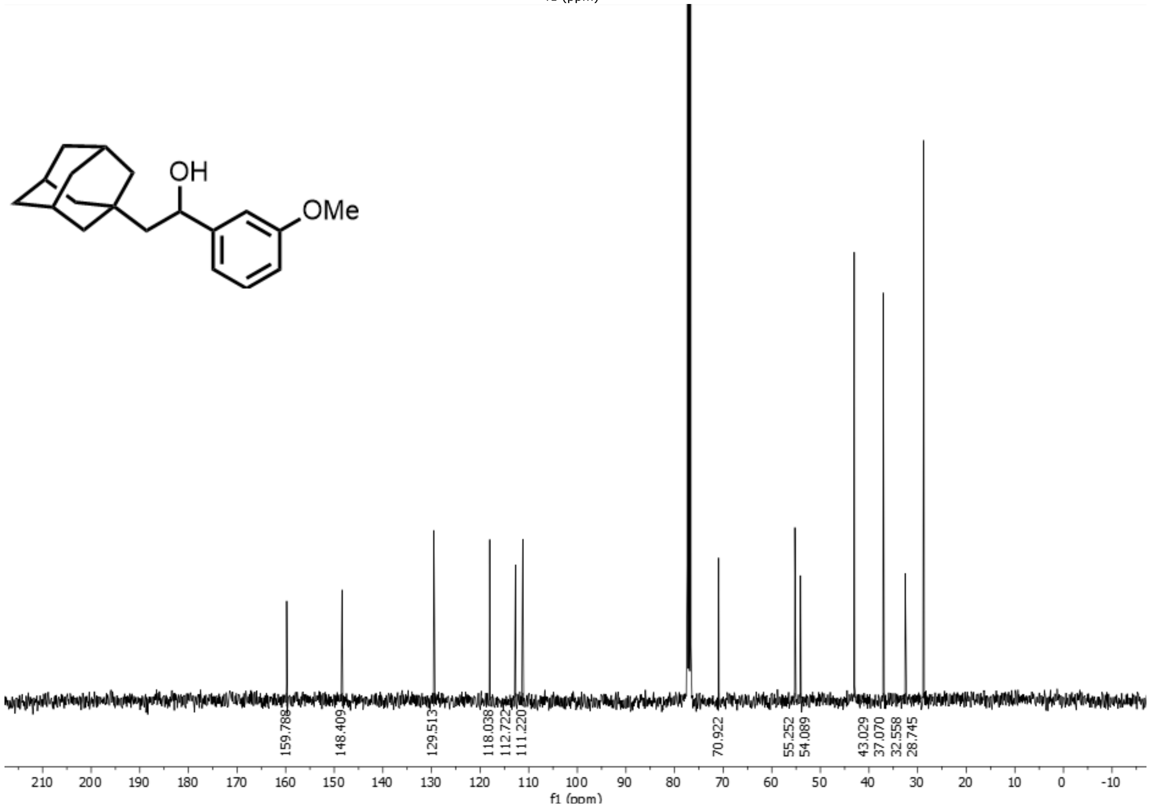

Compound 20. Top: ^{1}H NMR ($CDCl_3$, 400 MHz). Bottom: ^{13}C NMR ($CDCl_3$, 100 MHz)

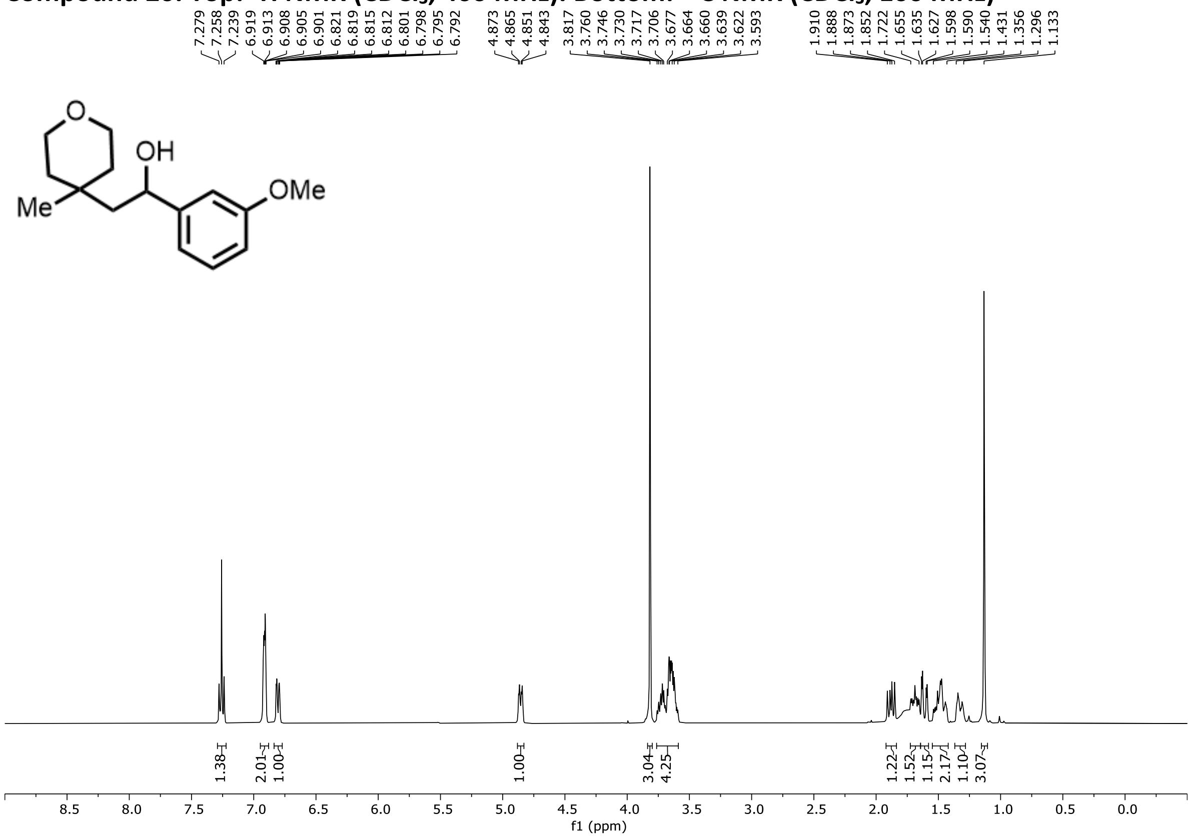

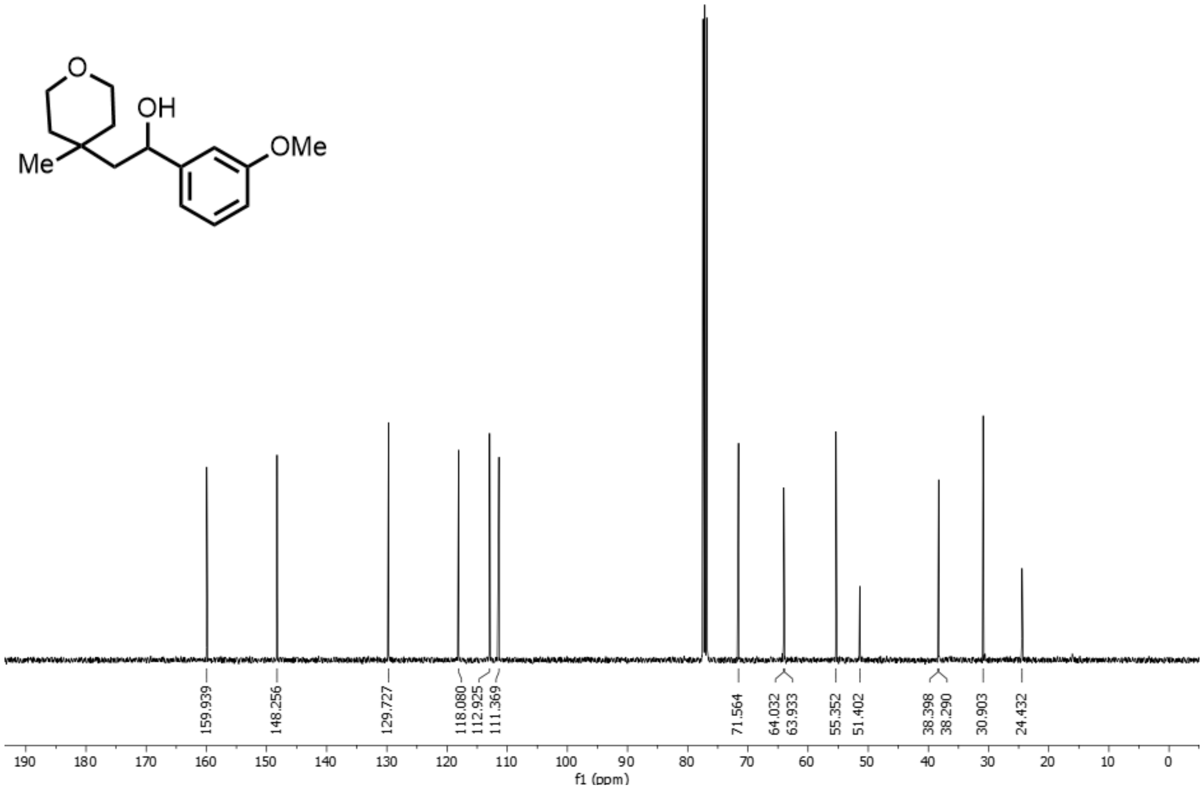

Compound 21. Top: ^{1}H NMR ($CDCl_3$, 400 MHz). Bottom: ^{13}C NMR ($CDCl_3$, 100 MHz)

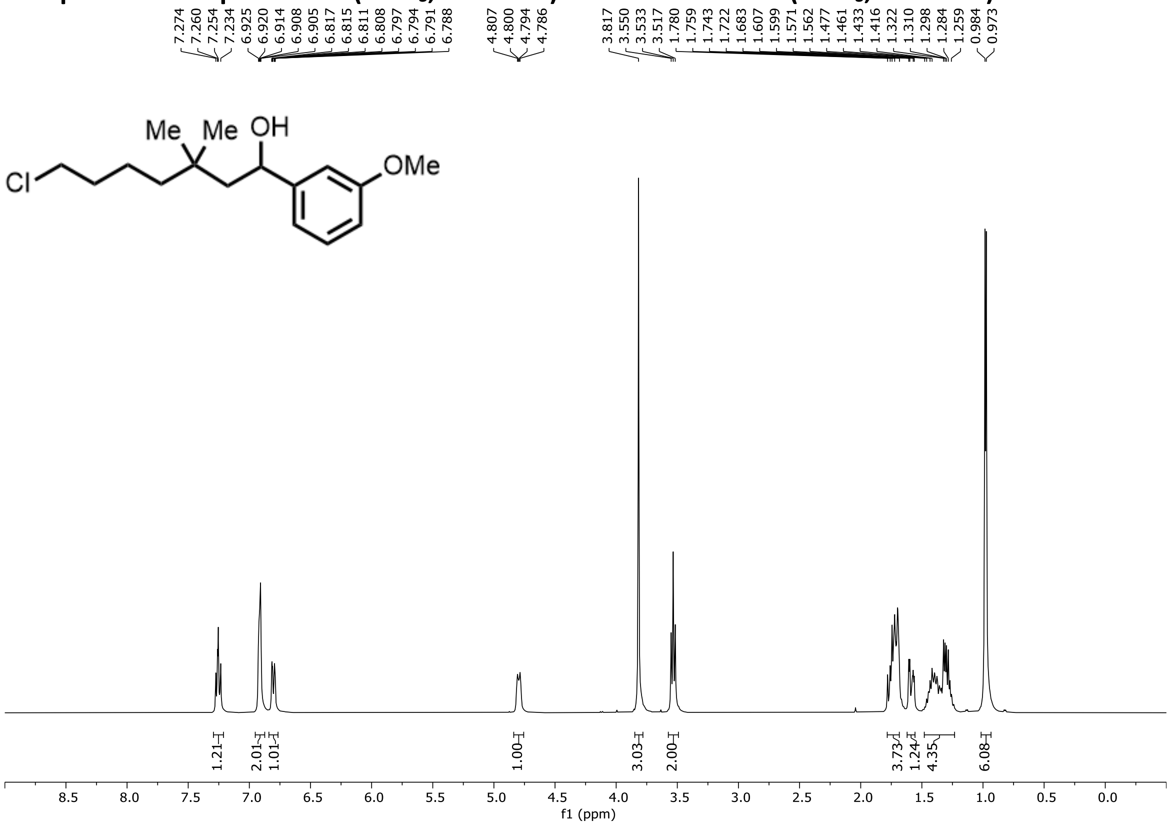

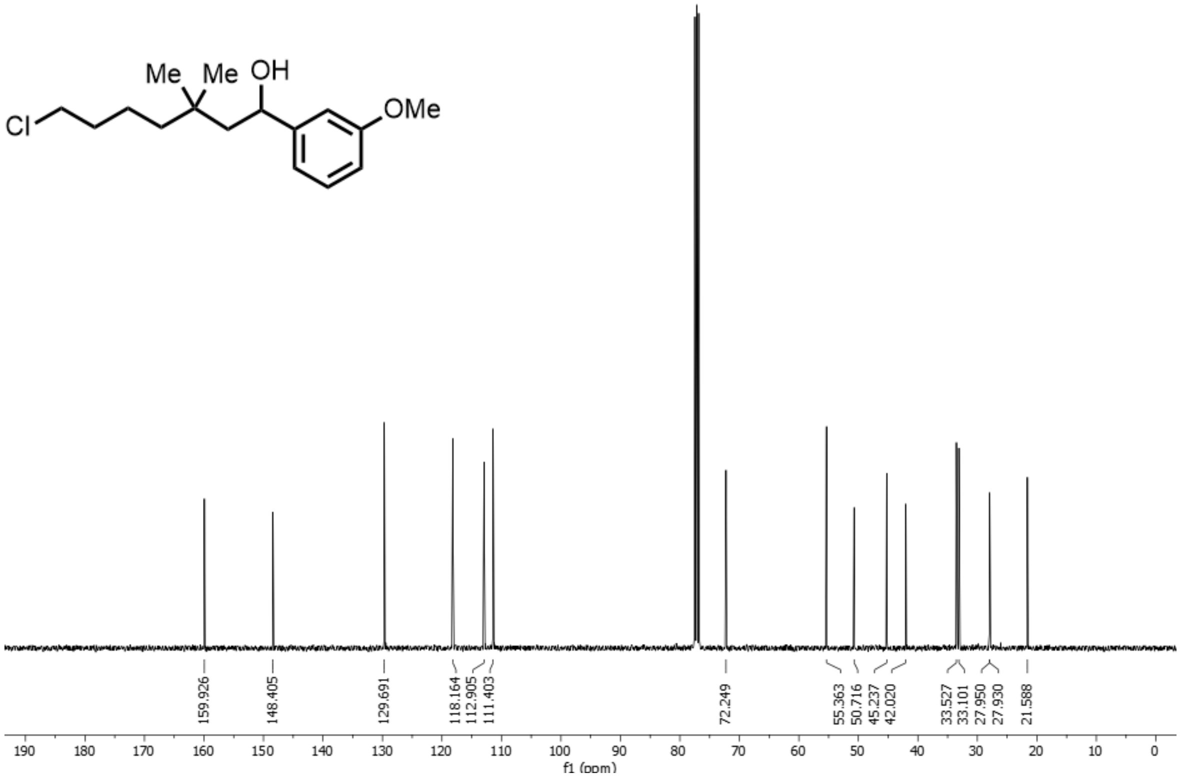

Compound 22. Top: ^{1}H NMR ($CDCl_3$, 400 MHz). Bottom: ^{13}C NMR ($CDCl_3$, 100 MHz)

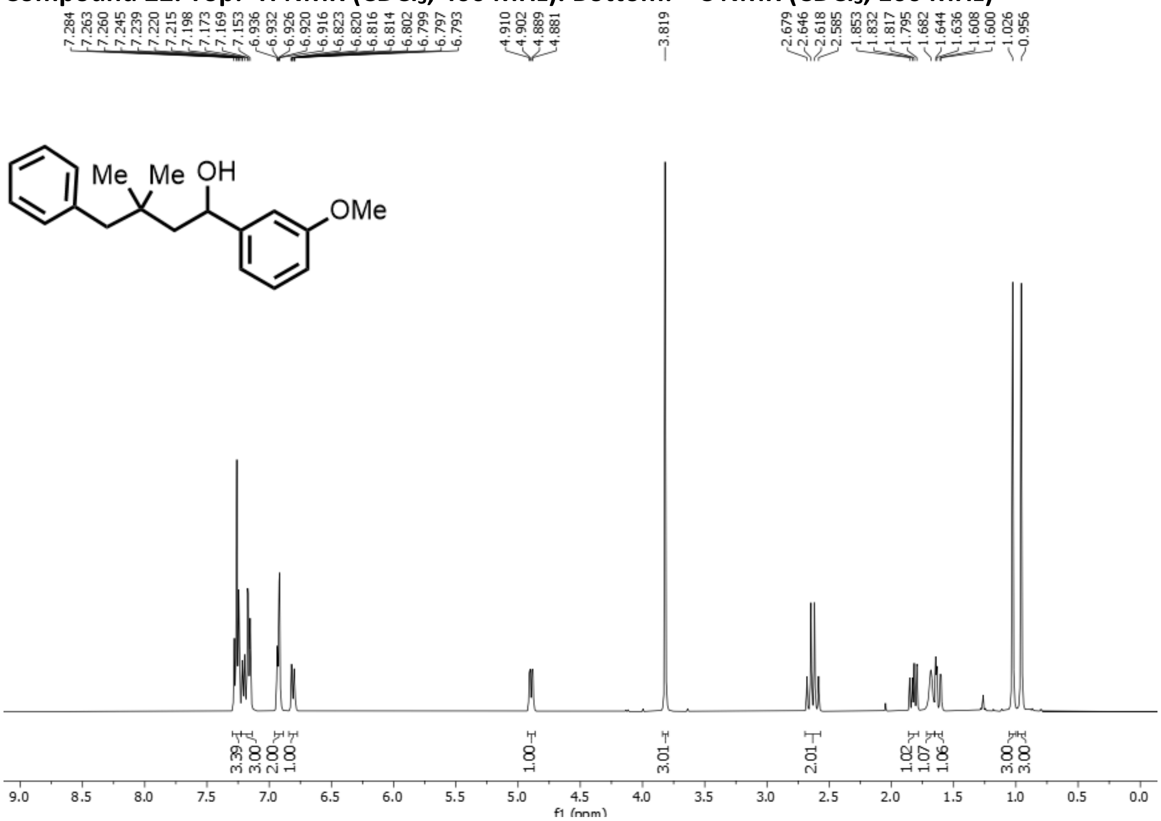

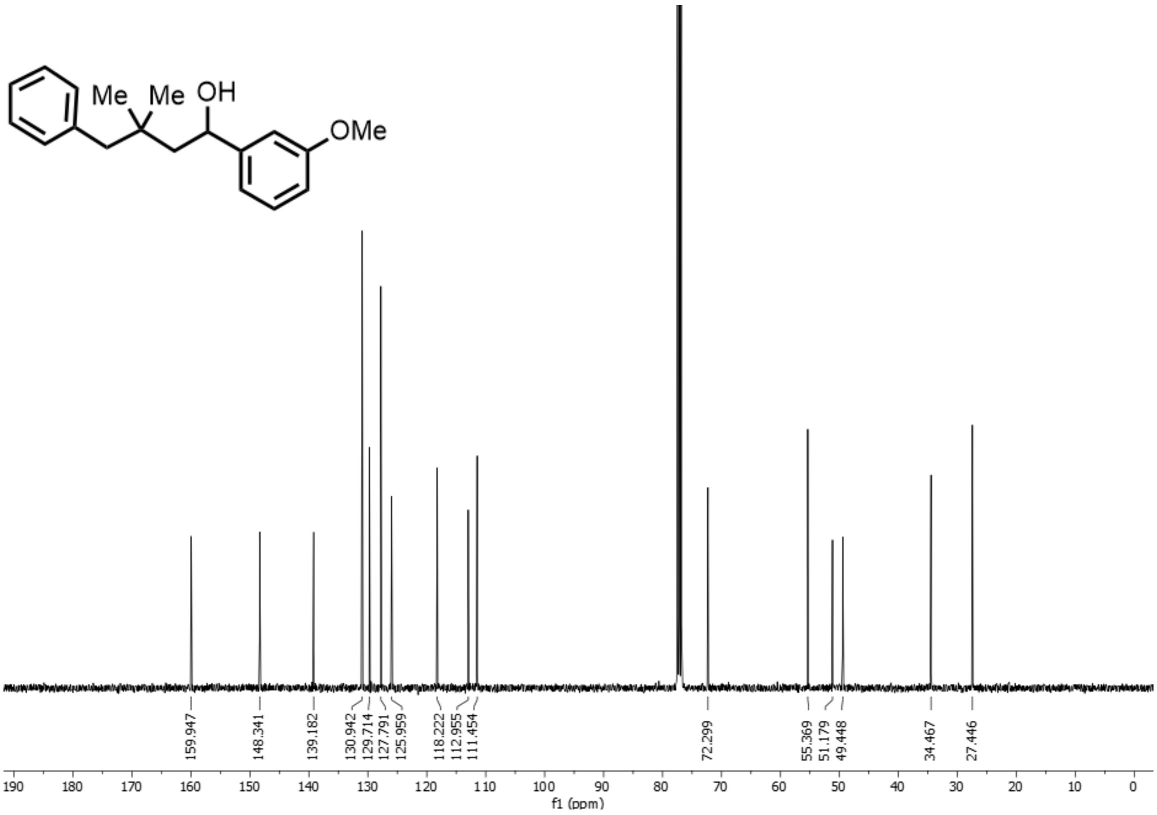

Compound 23. Top: ^{1}H NMR ($CDCl_3$, 400 MHz). Bottom: ^{13}C NMR ($CDCl_3$, 100 MHz)

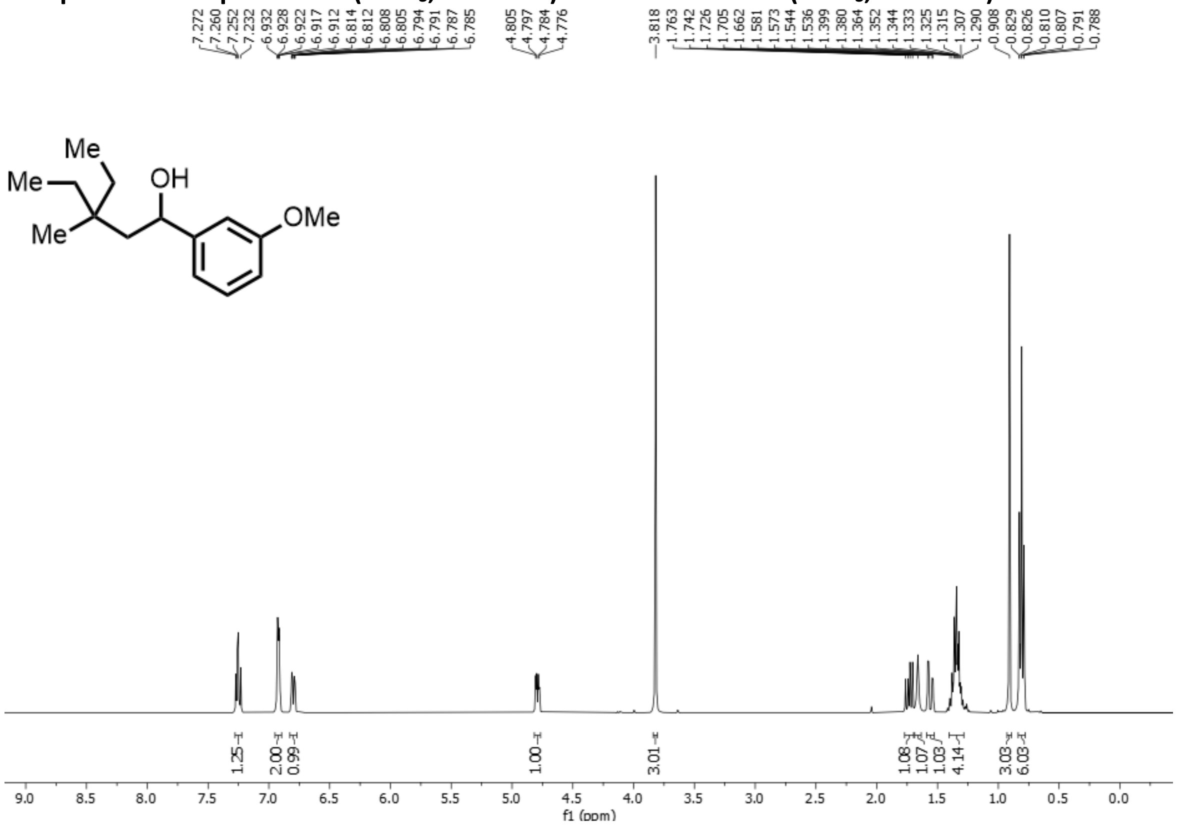

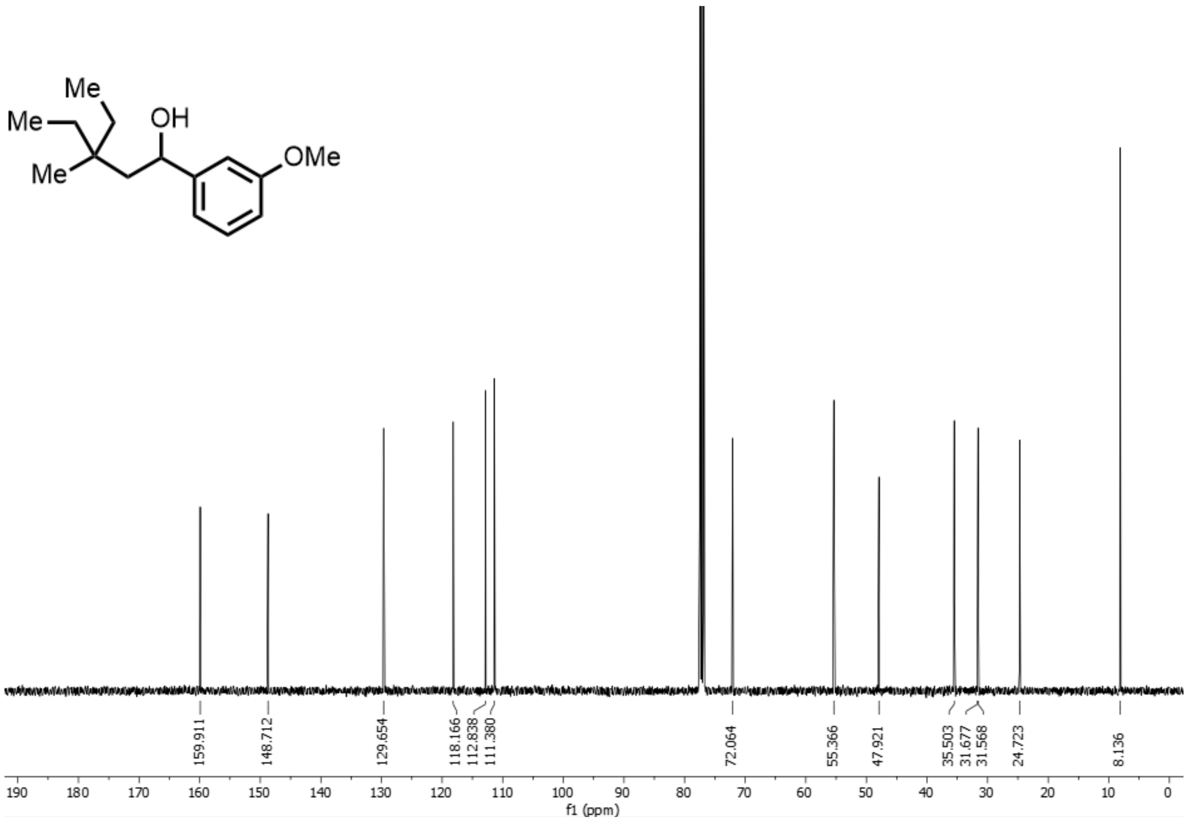

Compound 24. Top: ^{1}H NMR ($CDCl_3$, 400 MHz). Bottom: ^{13}C NMR ($CDCl_3$, 100 MHz)

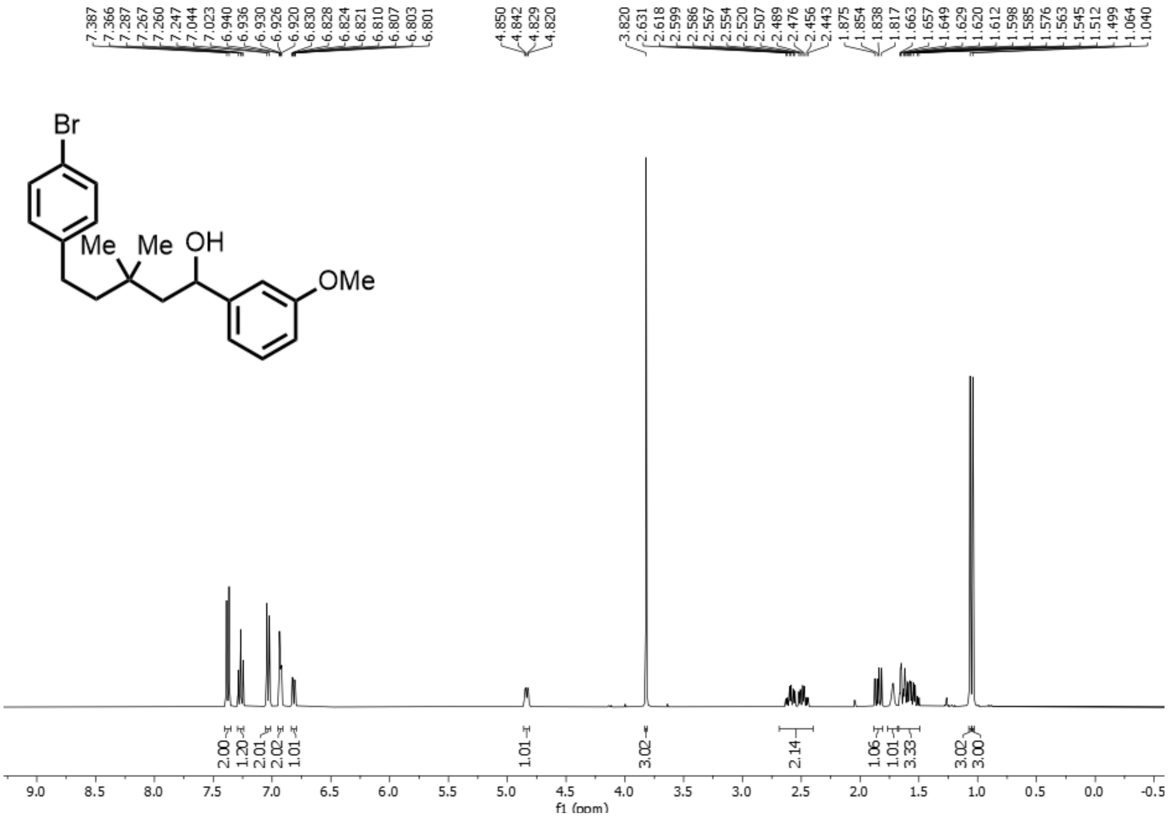

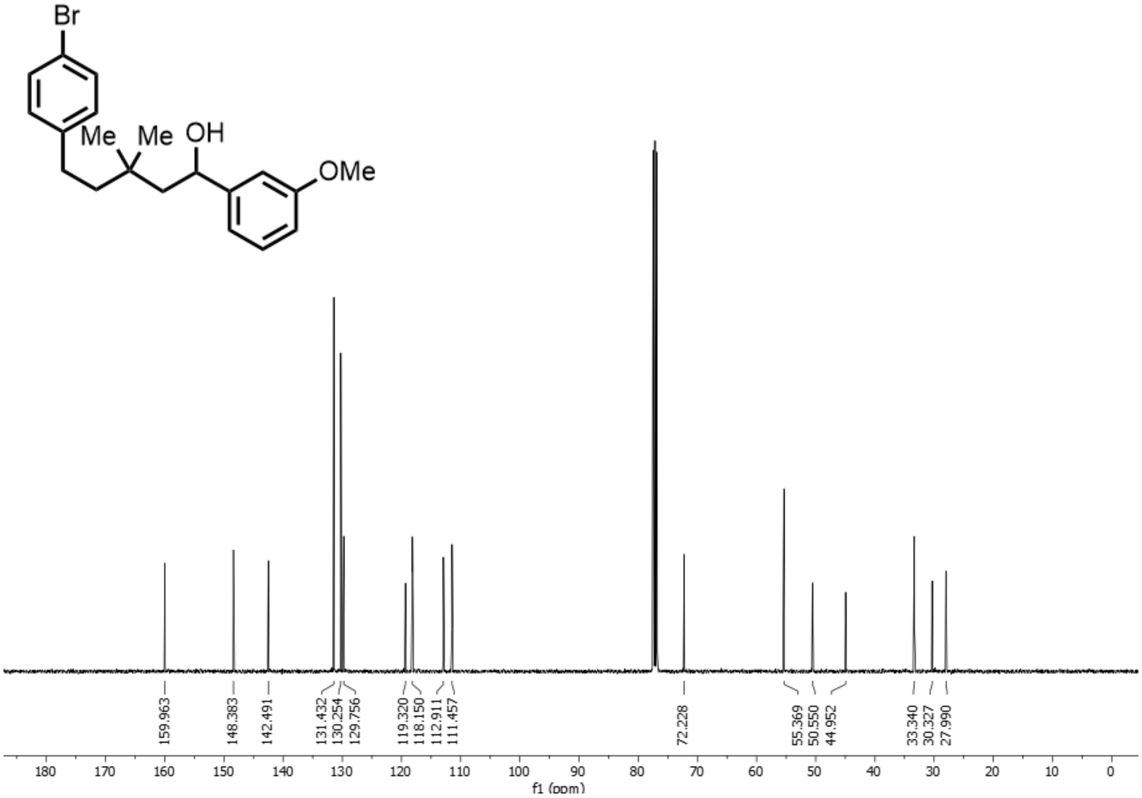

Compound 25. ^{1}H NMR ($CDCl_3$, 400 MHz).

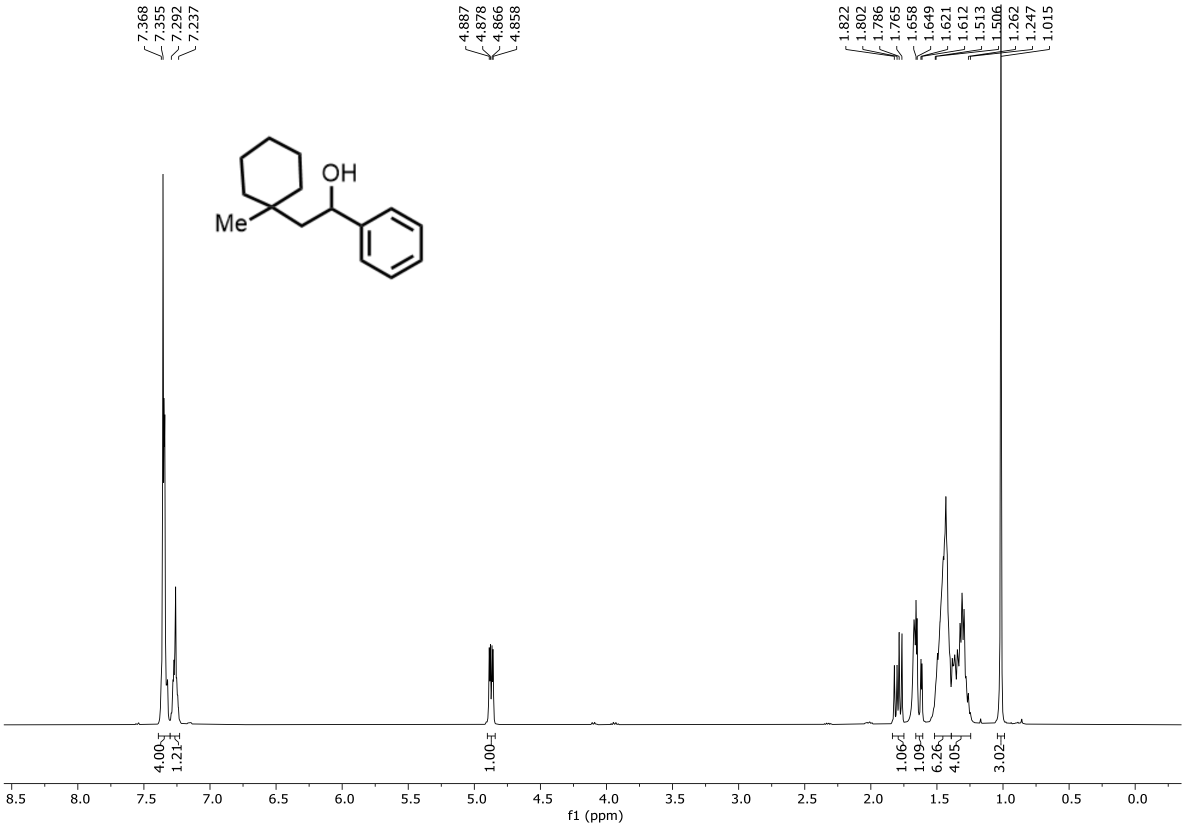

Compound 26. Top: ^{1}H NMR ($CDCl_3$, 400 MHz). Bottom: ^{13}C NMR ($CDCl_3$, 100 MHz)

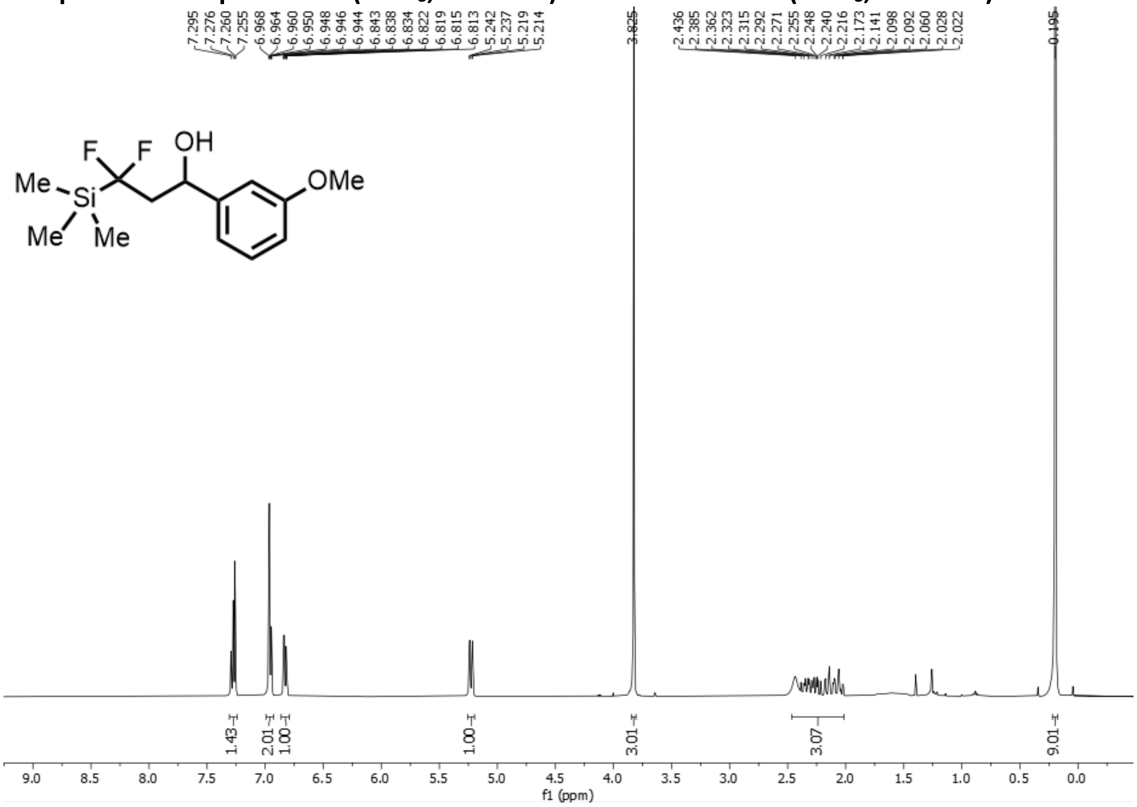

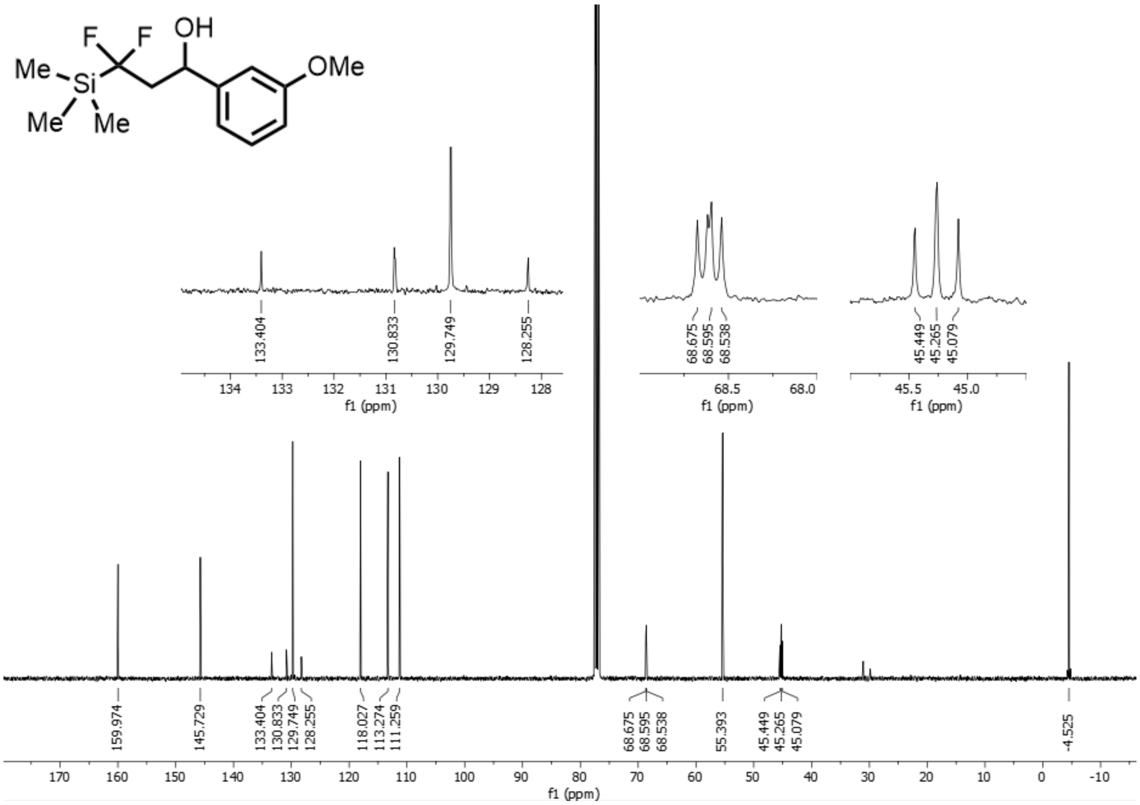

Compound 26. ^{19}F **NMR ($CDCl_3$, 376 MHz)**

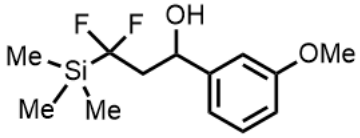

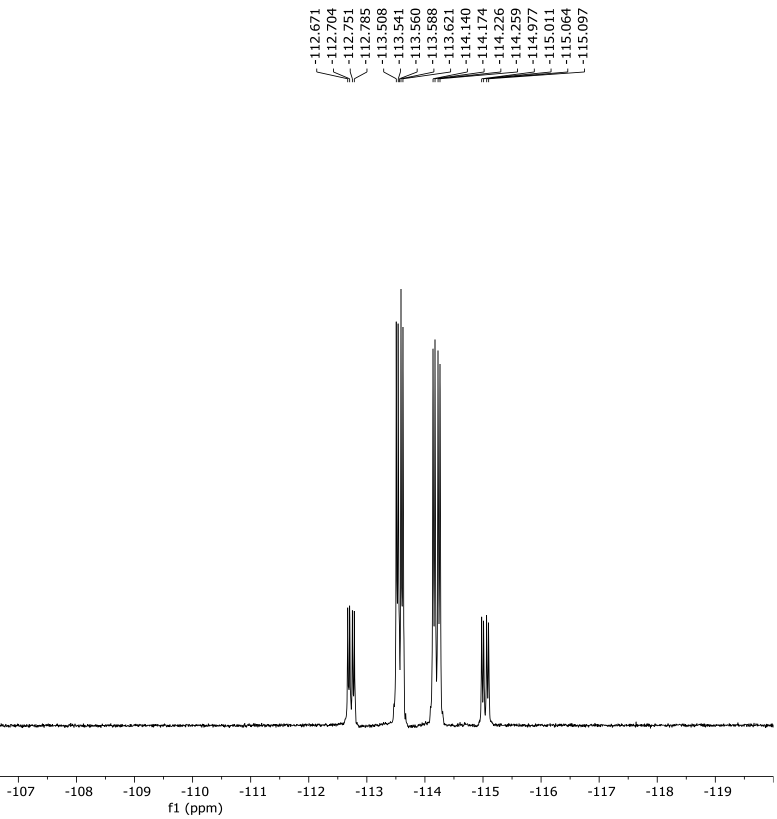

Compound 27. Top: ^{1}H NMR ($CDCl_3$, 400 MHz). Bottom: ^{13}C NMR ($CDCl_3$, 100 MHz)

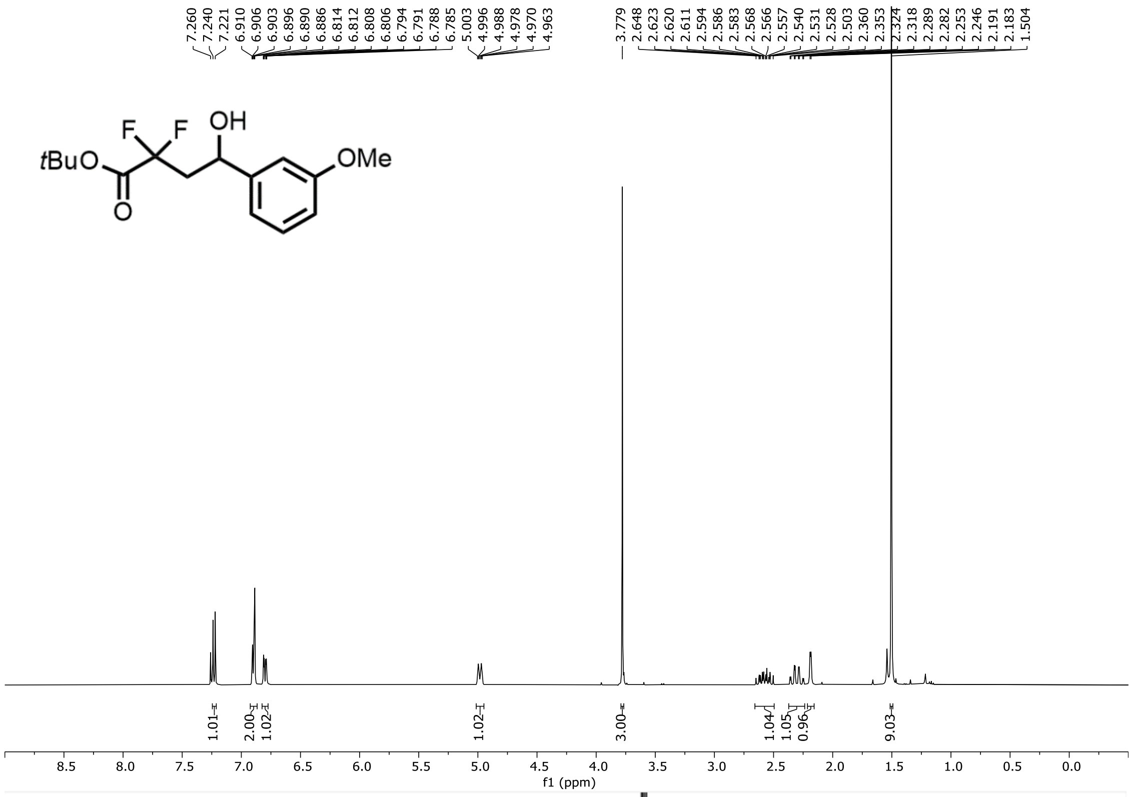

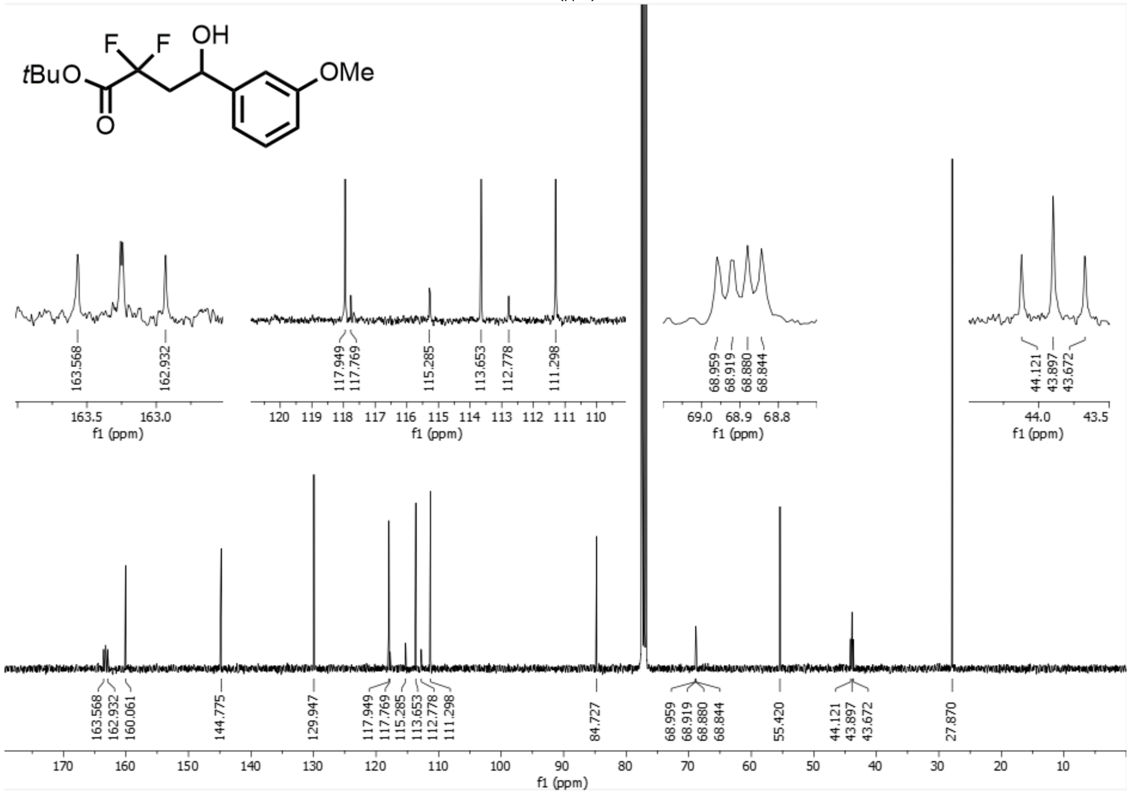

Compound 27. ^{19}F NMR ($CDCl_3$, 376 MHz)

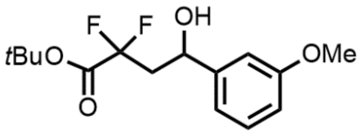

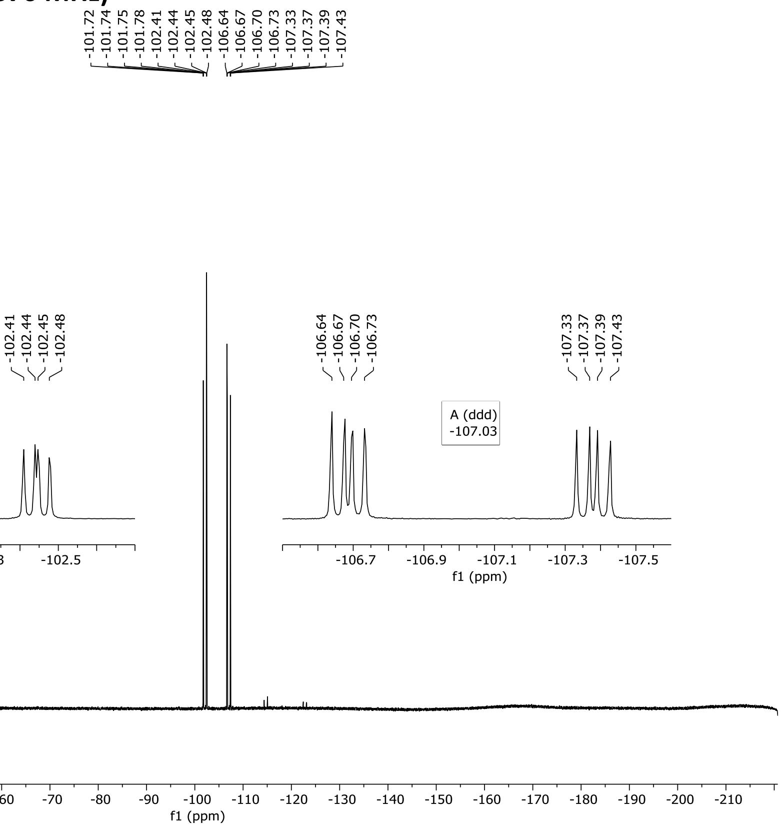

Compound 28. Top: ^{1}H NMR ($CDCl_3$, 400 MHz). Bottom: ^{13}C NMR ($CDCl_3$, 100 MHz)

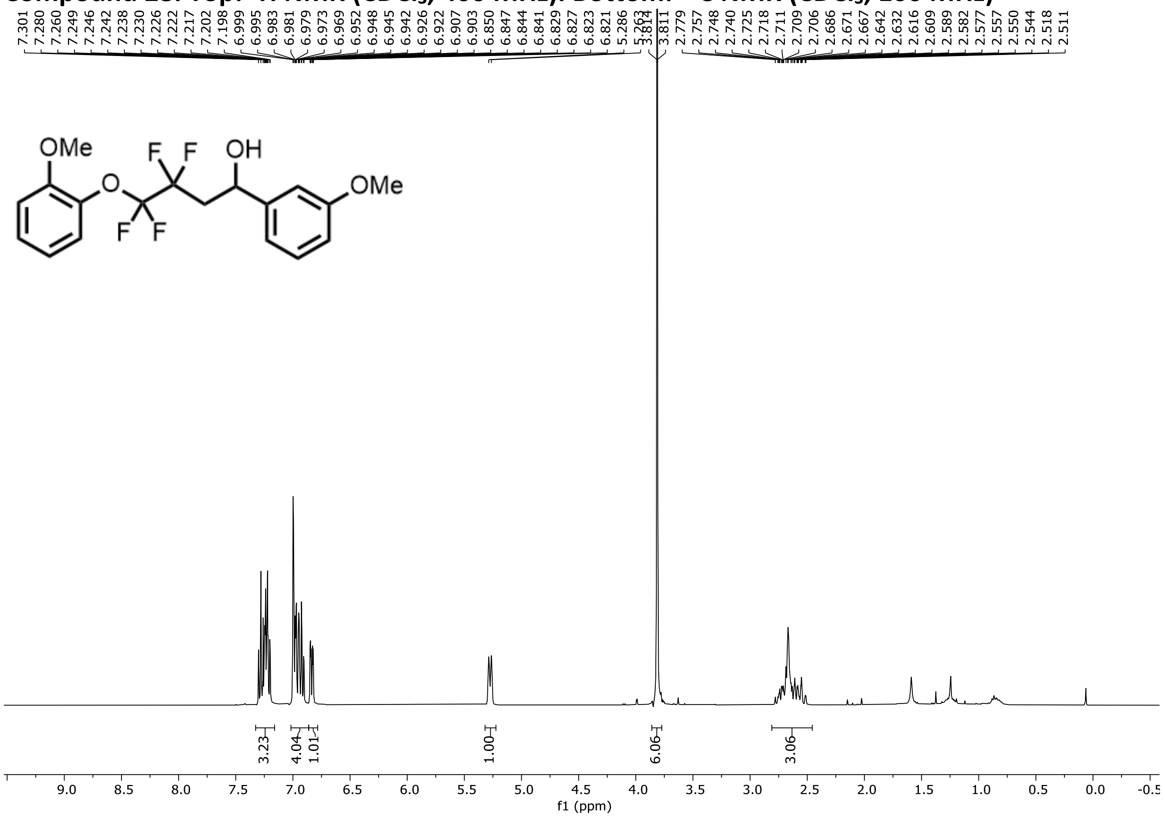

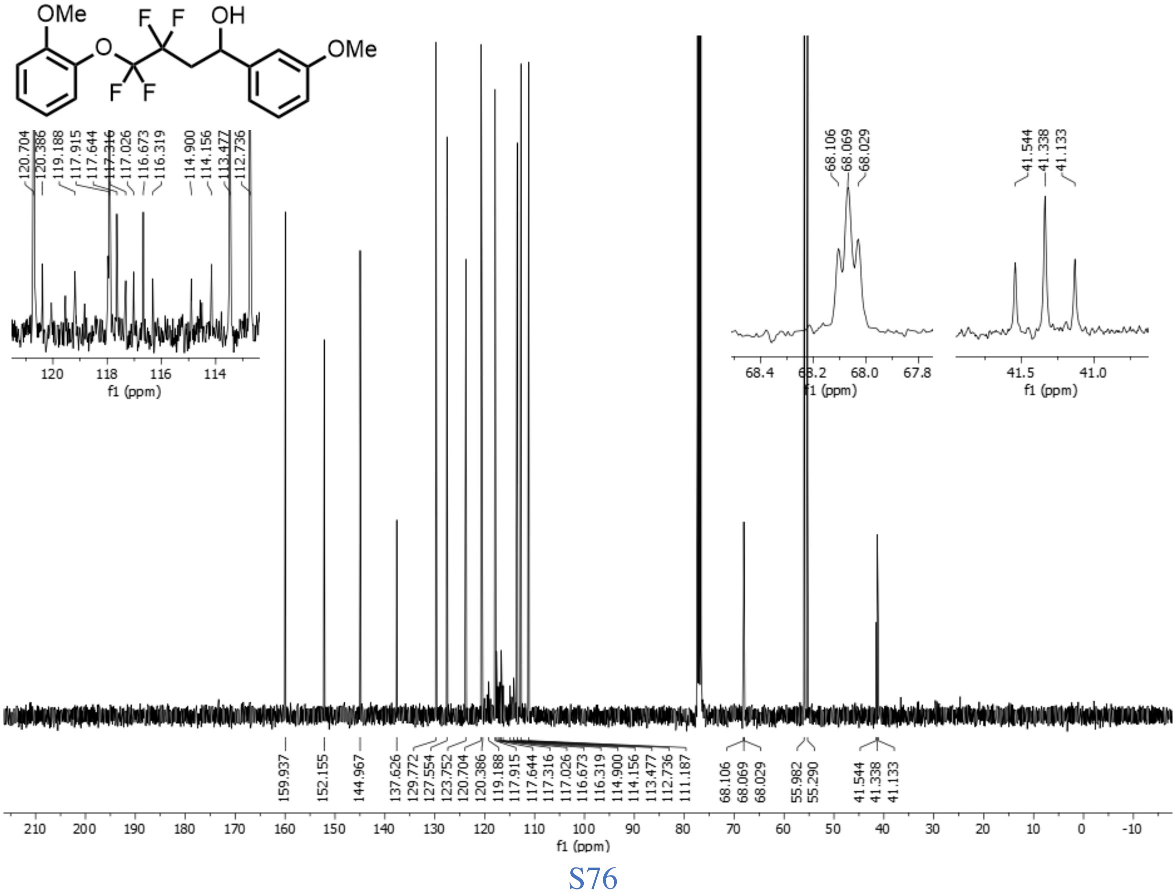

Compound 28. ^{19}**F NMR (CDCl$_3$, 376 MHz)**

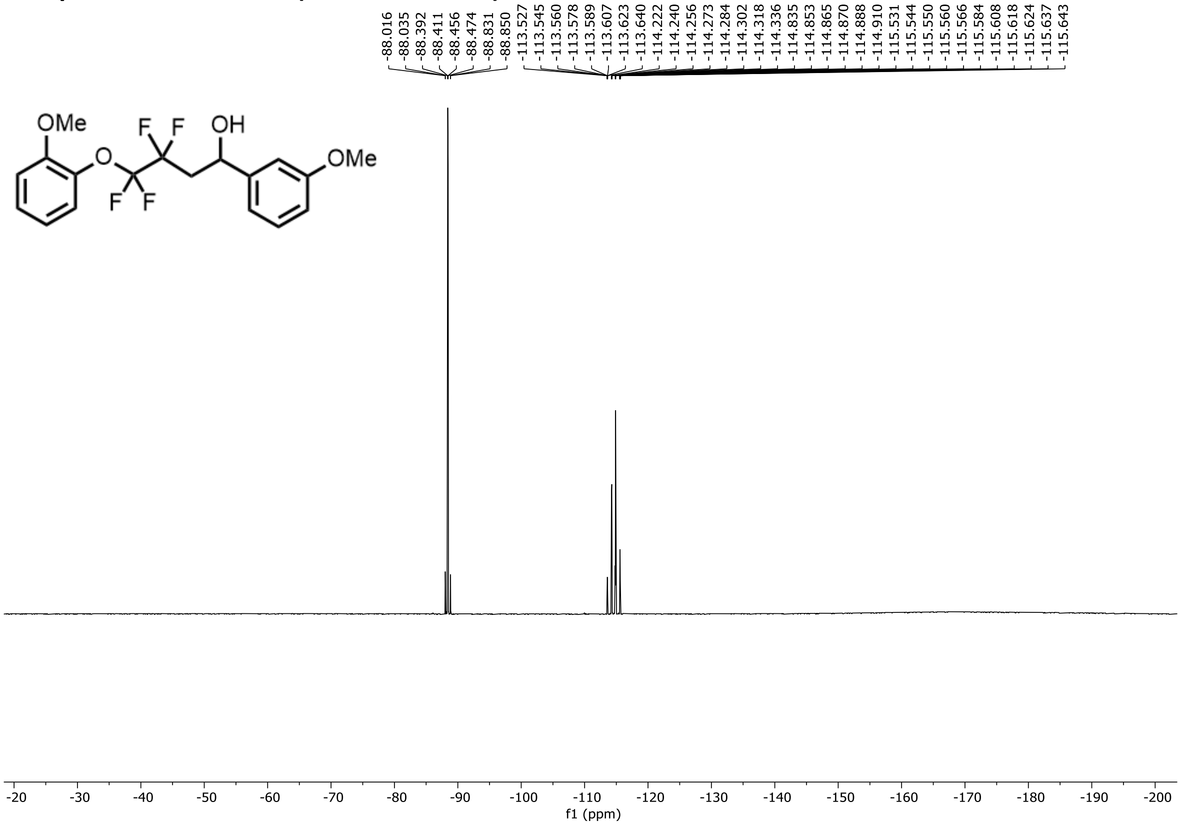

8. Crystallographic Data

Compound 18.

A single crystal of **18** (**CCDC 2268886**) was prepared by recrystallization in hexanes and a small amount of isopropanol. ORTEP diagram of 18 with 50% ellipsoid dimensions 0.33 × 0.13 × 0.05 mm^3.

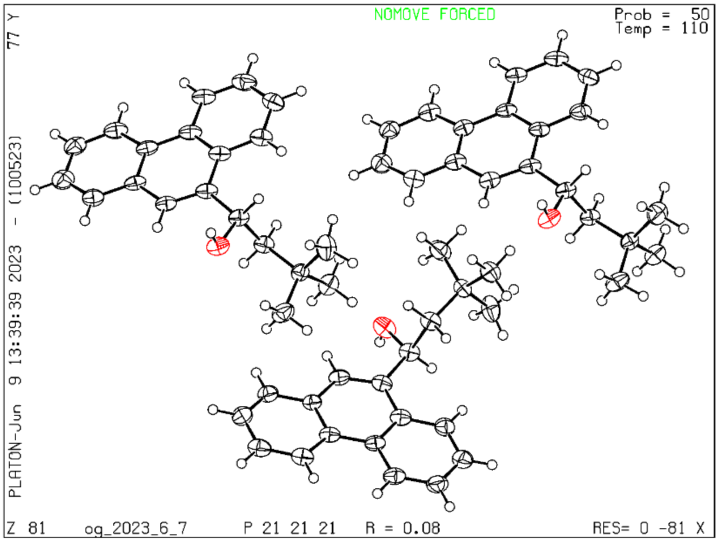

Table S5. Crystal Data of compound **18**

Formula	$C_{20}H_{22}O$
$D_{calc.}$/ g cm^{-3}	1.202
μ/mm^{-1}	0.551
Formula Weight	278.37
Color	colorless
Shape	plate-shaped
Size/mm^3	0.33×0.13×0.05
T/K	110.00(10)
Crystal System	orthorhombic
Flack Parameter	-0.19(13)
Hooft Parameter	-0.10(12)
Space Group	$P2_12_12_1$
a/Å	5.66110(10)
b/Å	26.2179(4)
c/Å	31.0807(6)
α/°	90
β/°	90
γ/°	90
V/Å^3	4613.06(14)
Z	12
Z'	3
Wavelength/Å	1.54184
Radiation type	Cu K_α
Θ_{min}/°	2.205
Θ_{max}/°	78.668
Measured Refl's.	71960
Indep't Refl's	9672
Refl's I≥2 σ(I)	8951
R_{int}	0.0871
Parameters	580
Restraints	0
Largest Peak	0.483
Deepest Hole	-0.264
GooF	1.035
wR_2 (all data)	0.2328
wR_2	0.2276
R_1 (all data)	0.0882
R_1	0.0844

Experimental. Single colorless plate-shaped crystals of OG_2023_6_7 were used as supplied. A suitable crystal with dimensions 0.33 × 0.13 × 0.05 mm^3 was selected and mounted on a Bruker Photon 2 area detector diffractometer. The crystal was kept at a steady T = 110.00(10) K during data collection. The structure was solved with the ShelXT 2018/2 (Sheldrick, 2018) solution program using dual methods and by using Olex2 1.5 (Dolomanov et al., 2009) as the graphical interface. The model was refined with XL (Sheldrick, 2008) using full matrix least squares minimization on $\boldsymbol{F}^2$. The Flack parameter was refined to -0.19(13). Determination of absolute structure using Bayesian statistics on Bijvoet differences using the Olex2 results in -0.10(12).

Crystal Data. $C_{20}H_{22}O$, M_r = 278.37, orthorhombic, $P2_12_12_1$ (No. 19), a = 5.66110(10) Å, b = 26.2179(4) Å, c = 31.0807(6) Å, $\alpha = \beta = \gamma = 90°$, V = 4613.06(14) Å^3, T = 110.00(10) K, Z = 12, Z' = 3, μ(Cu K_α) = 0.551, 71960 reflections measured, 9672 unique (R_{int} = 0.0871) which were used in all calculations. The final wR_2 was 0.2328 (all data) and R_1 was 0.0844 (I≥2 σ(I)).

9. Computational Methods, Energies, and Coordinates

All geometry optimizations of intermediates and transition states were achieved using spin-unrestricted UB3LYP[16]-D3[17]/6-31G(d)[18]method, in THF solvent using the SMD solvent model[19] with "opt=noeigen" and "guess=mix" keywords as implemented in Gaussian16[20]. Frequency calculations were also done for all the stationary points, and transition states were characterized by the presence of one unique imaginary frequency, which suggested that they were first-order saddle points on the potential energy surface. Intrinsic Reaction Coordinate (IRC) calculations were done on the transition states to verify that they were the correct transition state associated with the reaction. The endpoint geometries obtained from the IRC calculations were further optimized to verify the authenticity of the transition state. The thermochemistry: enthalpy (ΔH) and free energy (ΔG) were obtained at the temperature of 298 K. All structural figures were generated with CYLview.[21] Distances in structural figures are shown in Å and energies are in kcal/mol. Noncovalent interaction (NCI) analysis, also known as reduce density gradient (RDG) method, was performed on Multiwfn to study the possible effect of noncovalent interaction in the relevant transition states.[22] Extension distance of 0 Bohr, medium quality grid (totally about 512000 points) were set by default. Further visualization of the color-filled RDG isosurface was realized by VMD, where RDG isosurface and color range were set as 0.5, and -0.035 to 0.2, respectively.[23]

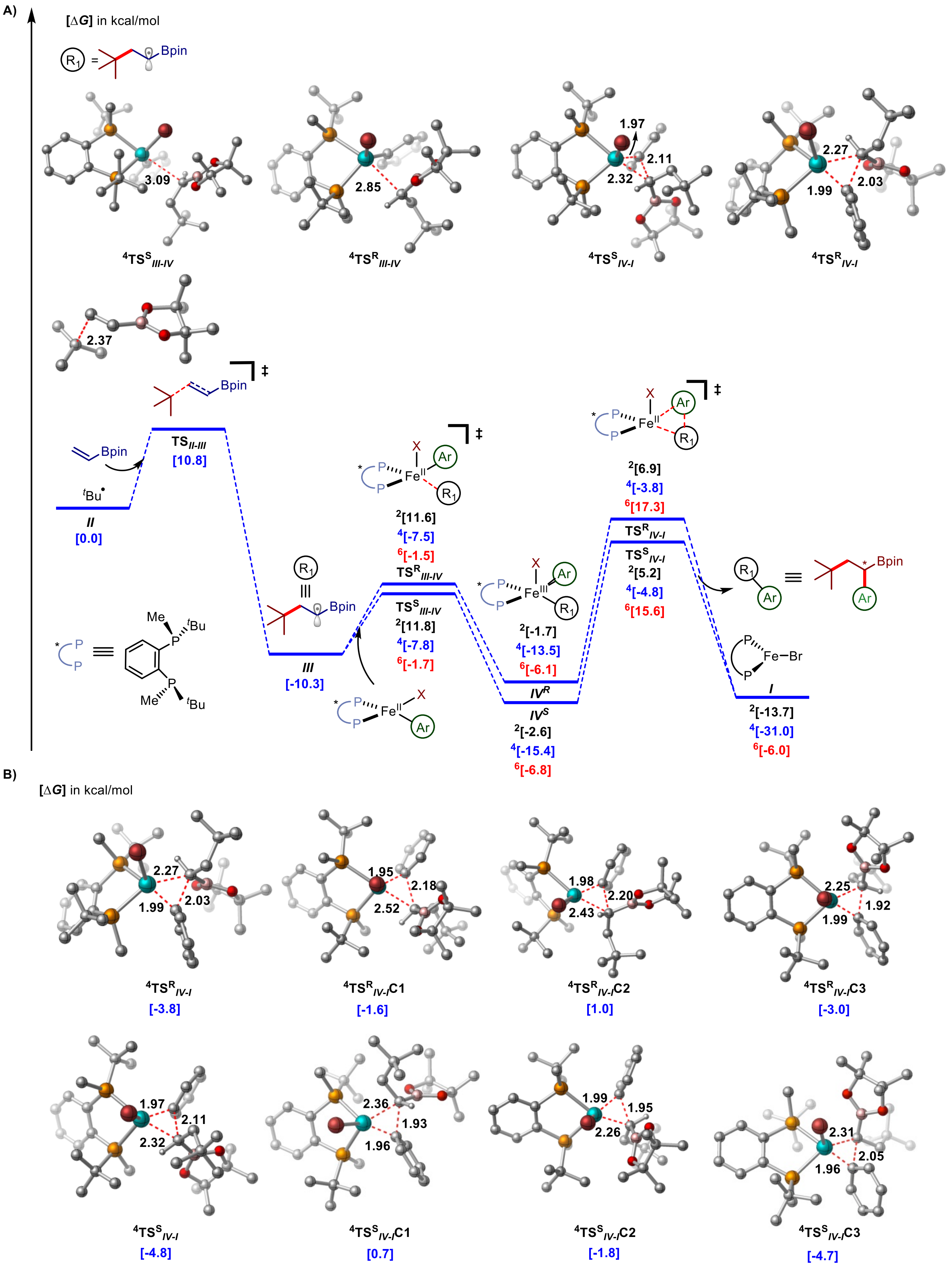

Figure S4. (A) Full Gibbs free energy (UB3LYP-D3/6-31G(d)-THF(SMD); kcal/mol) profile including the enantiomeric transition states for Giese addition and radical recombination by monophenylated

iron(II) leading to the formation of Fe(I)-Br complex along with the desired organic product by reductive elimination for BenzP* ligand. Multiplicities in superscripts. Stereochemistry is also in superscripts. (B) Conformational search for the C-C bond formation TS in quartet spin state.

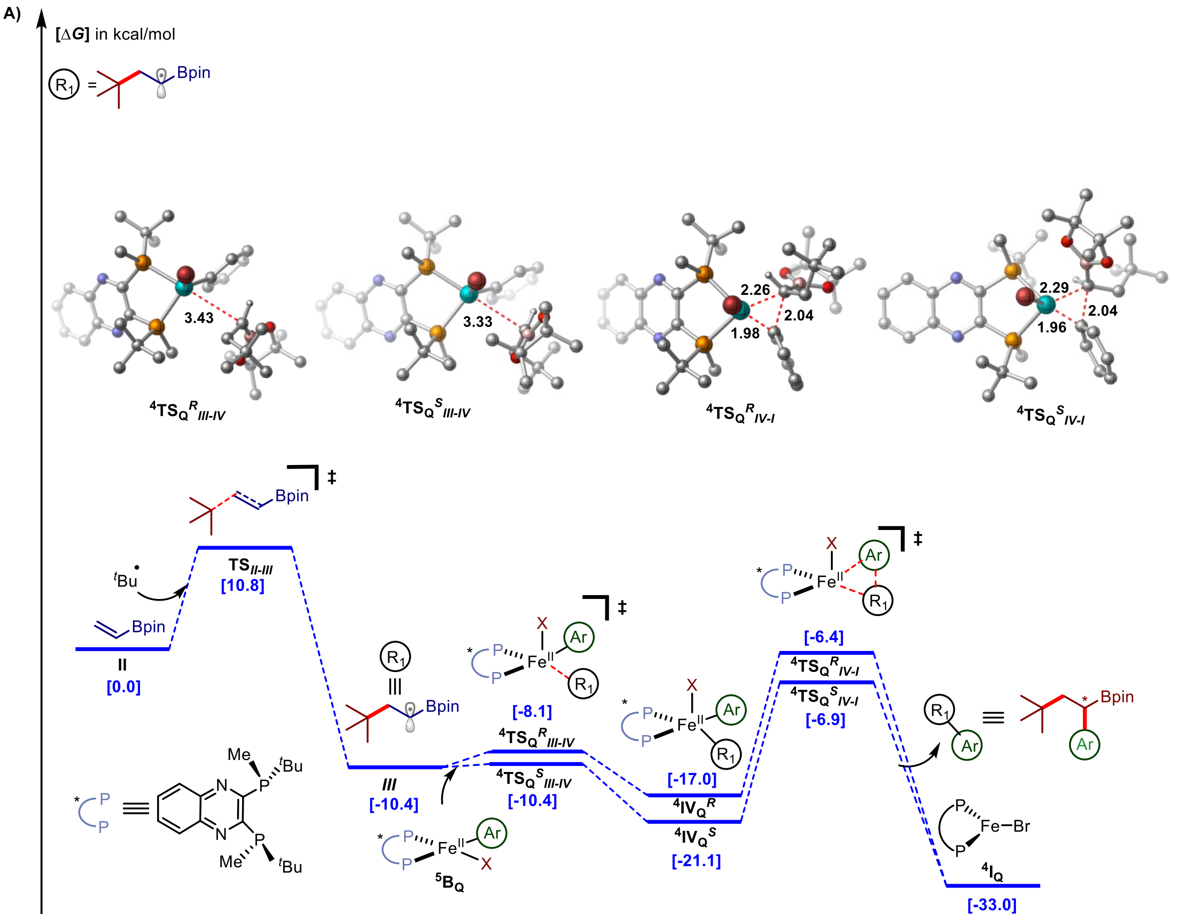

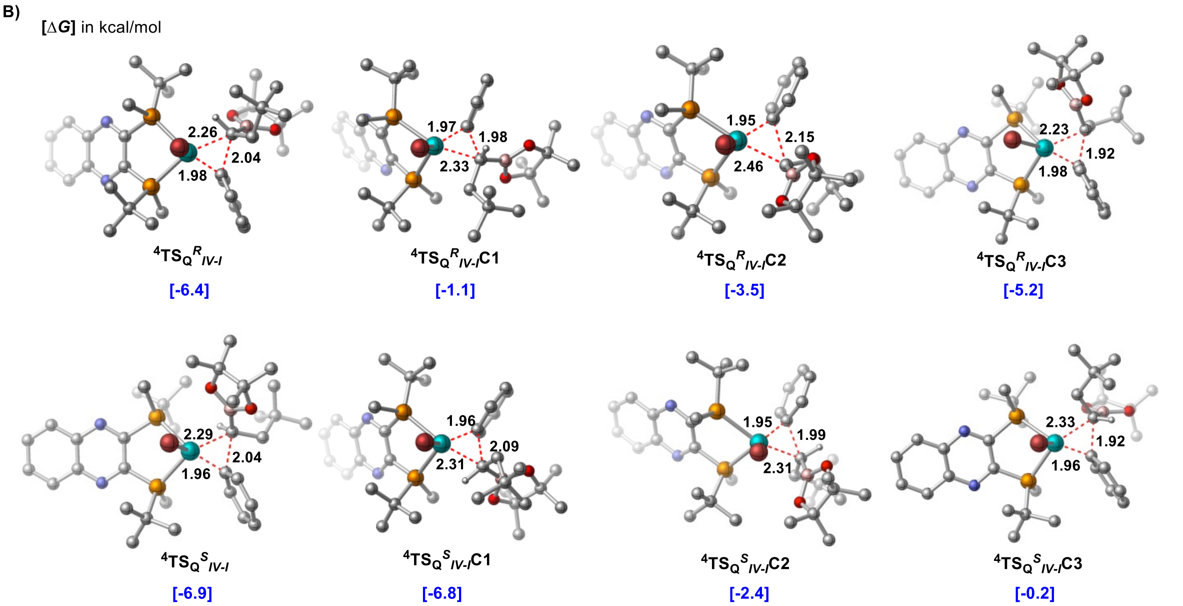

Figure S5. (A) Full Gibbs free energy (UB3LYP-D3/6-31G(d)-THF(SMD); kcal/mol) profile including the enantiomeric transition states for Giese addition and radical recombination by monophenylated

iron(II) leading to the formation of Fe(I)-Br complex along with the desired organic product by reductive elimination for QuinoxP* ligand. Multiplicities in superscripts. Stereochemistry is also in superscripts. (B) Conformational search for the C-C bond formation TS in quartet spin state.

We have done NBO calculation on the lowest energy stereo-determining transition state and the C-H-O interaction energy for the TS(S) is found to be 1.2 kcal/mol. Based on the Second Order Perturbation Theory Analysis of Fock Matrix in NBO Basis, we see a moderately strong interaction between the lone pair of the Oxygen atom and the anti-bonding orbital of the adjacent C-H bond (~1.2 kcal/mol).

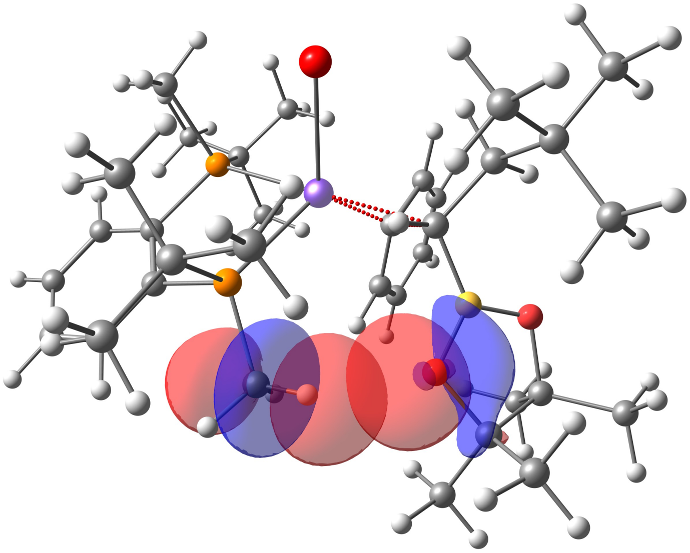

Such C-H-O interactions with pinacol oxygen are previously reported in the literature. [24]

Table S6. Cartesian coordinates (xyz format) and energies of all the structures involved in each reaction mechanism calculated at the UB3LYP-D3/6-31G(d)-SMD(THF) level of theory.

1
E(scf) = -2729.58935808 a.u.
ν_{min} = 257.9 cm^{-1}

Br	0.000000	0.000129	-1.208649	H	1.305187	0.753144	2.369634
C	0.000000	0.000093	0.842416	H	2.164454	0.223144	0.908184
C	-1.266065	0.730867	1.271935	H	1.275796	1.762884	0.908576
H	-1.275796	1.762884	0.908576	C	0.000000	-1.461669	1.272007
H	-2.164454	0.223144	0.908184	H	-0.888969	-1.986045	0.908638
H	-1.305187	0.753144	2.369634	H	0.888969	-1.986045	0.908638
C	1.266065	0.730867	1.271935	H	0.000000	-1.506539	2.369701

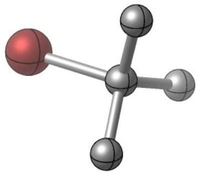

Zero-point correction= 0.122884 (Hartree/Particle)
Thermal correction to Energy= 0.129495
Thermal correction to Enthalpy= 0.130439
Thermal correction to Gibbs Free Energy= 0.092315
Sum of electronic and zero-point Energies= -2729.466474
Sum of electronic and thermal Energies= -2729.459863
Sum of electronic and thermal Enthalpies= -2729.458919
Sum of electronic and thermal Free Energies= -2729.497

[1]A
E(scf) = -7716.56065738 a.u.
ν_{min} = 11.5 cm^{-1}

Br	9.444474	6.184127	14.172327	H	6.355438	8.226635	10.847095
Br	8.836975	10.012343	13.648803	H	6.287907	6.490773	10.493940
Fe	7.918913	7.976579	14.443381	H	7.469556	7.112599	11.660525
P	5.801255	8.262343	13.753318	C	3.985285	7.143513	11.837201
P	7.060347	7.420248	16.441095	H	3.218928	6.940826	12.591590
C	4.651487	7.934880	15.151745	H	3.851646	6.412545	11.029012
C	3.261251	8.102191	15.068205	H	3.806772	8.138052	11.414004
H	2.812075	8.514368	14.170424	C	5.307747	9.910997	13.125191
C	2.441868	7.735561	16.135788	H	5.532141	10.648827	13.900810
H	1.365912	7.866076	16.060174	H	4.242093	9.956416	12.882209
C	3.002811	7.189036	17.293082	H	5.900625	10.154182	12.240524
H	2.363809	6.882678	18.116655	C	7.556913	8.714486	17.722556
C	4.386452	7.048611	17.399700	C	7.028872	10.076153	17.229635
H	4.811375	6.633749	18.308081	H	7.435722	10.337431	16.246386
C	5.222512	7.444253	16.345128	H	7.333118	10.859249	17.935449
C	5.403834	7.001036	12.407006	H	5.934579	10.087273	17.165841
C	5.587607	5.600114	13.021425	C	7.015226	8.418308	19.128487
H	6.602457	5.457508	13.410268	H	5.922073	8.455522	19.161528
H	5.416969	4.840324	12.248390	H	7.391095	9.181353	19.822580
H	4.877027	5.418586	13.835820	H	7.344556	7.442712	19.502725
C	6.444424	7.228485	11.290840	C	9.099390	8.724044	17.743783

H	9.509099	7.767282	18.087179	H	7.159073	5.017096	16.407054
H	9.447100	9.504278	18.432309	H	6.944946	5.589599	18.082689
H	9.518938	8.937271	16.754029	H	8.541666	5.694415	17.282110
C	7.461962	5.773967	17.136299				

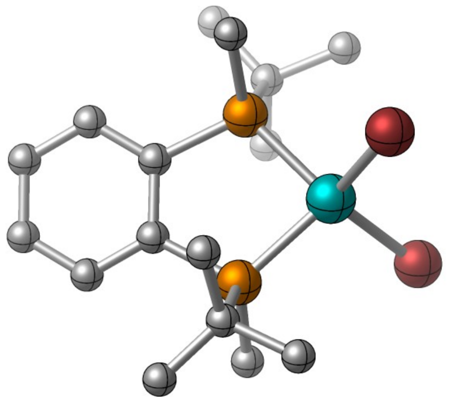

Zero-point correction= 0.411060 (Hartree/Particle)
Thermal correction to Energy= 0.438545
Thermal correction to Enthalpy= 0.439489
Thermal correction to Gibbs Free Energy= 0.353308
Sum of electronic and zero-point Energies= -7716.149597
Sum of electronic and thermal Energies= -7716.122112
Sum of electronic and thermal Enthalpies= -7716.121168
Sum of electronic and thermal Free Energies= -7716.207349

^{1}B
E(scf) = -5376.41683073 a.u.
ν_{min} = 21.0 cm^{-1}

Br	3.044631	6.158846	12.844486	H	8.773046	6.225914	12.499725
Fe	4.254092	7.977295	13.732179	C	7.324738	6.960457	13.926065
P	5.309603	7.750585	15.682714	C	4.393432	6.528354	16.820584
P	6.252022	7.874163	12.740250	C	4.522731	5.110513	16.238103
C	6.960582	7.014670	15.288151	H	4.195389	5.075808	15.194414
C	7.825074	6.472235	16.250171	H	3.887001	4.423421	16.811823
H	7.583977	6.545522	17.304976	H	5.552343	4.741028	16.292639
C	9.001331	5.828687	15.865393	C	2.916832	6.977275	16.776121
H	9.657584	5.405587	16.621157	H	2.796650	8.015586	17.109603
C	9.327155	5.718043	14.511291	H	2.316003	6.340184	17.437913
H	10.232256	5.199032	14.207626	H	2.504812	6.892756	15.764514
C	8.498143	6.291808	13.547754	C	4.870527	6.537149	18.282785

H	5.910555	6.216092	18.389015	H	6.998706	11.084834	10.926706
H	4.255208	5.831925	18.857238	H	5.456628	10.373917	11.423577
H	4.761723	7.520537	18.751079	C	6.290955	6.846244	11.213687
C	5.757232	9.139449	16.808994	H	5.971135	5.834908	11.478189
H	6.191519	9.953535	16.224544	H	7.277893	6.805094	10.743411
H	6.486941	8.812876	17.556097	H	5.569918	7.251634	10.497995
H	4.867986	9.510075	17.325954	C	3.714621	9.828076	13.694065
C	7.232814	9.436270	12.309999	C	3.125902	10.138928	12.441565
C	7.247047	10.335892	13.556798	H	3.205047	9.429606	11.615080
H	6.234510	10.621798	13.854339	C	2.448632	11.337638	12.204246
H	7.812247	11.251889	13.340976	H	2.018150	11.533949	11.224017
H	7.731152	9.835949	14.403571	C	2.323113	12.283894	13.226594
C	8.679727	9.140473	11.880551	H	1.797643	13.219442	13.049935
H	9.267743	8.704374	12.693732	C	2.880970	12.008901	14.476415
H	9.162809	10.083363	11.591869	H	2.786683	12.732789	15.284158
H	8.733338	8.469135	11.016687	C	3.562262	10.805534	14.698143
C	6.491035	10.139369	11.158266	H	3.984656	10.636658	15.683190
H	6.488639	9.534634	10.244083				

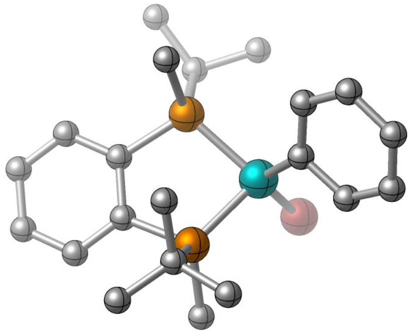

Zero-point correction= 0.499479 (Hartree/Particle)
Thermal correction to Energy= 0.530692
Thermal correction to Enthalpy= 0.531636
Thermal correction to Gibbs Free Energy= 0.438186
Sum of electronic and zero-point Energies= -5375.917352
Sum of electronic and thermal Energies= -5375.886139
Sum of electronic and thermal Enthalpies= -5375.885194
Sum of electronic and thermal Free Energies= -5375.978645

^{1}C

E(scf) = -3036.27986455 a.u.

ν_{min} = 35.7 cm^{-1}

C	-1.509724	-1.053381	0.162788	C	-1.887497	-2.090834	1.027130

C	-2.332980	-0.732889	-0.940923	H	2.405879	6.337811	-1.403694
C	-3.541674	-1.420149	-1.115884	C	1.275358	-1.267546	1.013918
C	-3.079207	-2.788324	0.822970	H	2.232784	-0.750107	1.133292
H	-3.356276	-3.595551	1.495790	H	0.998008	-1.715501	1.971782
C	-3.913541	-2.443145	-0.241831	H	1.411989	-2.058015	0.274594
H	-4.848179	-2.975067	-0.399025	C	-2.910212	1.943153	-1.759924
H	-1.249939	-2.363811	1.862136	H	-2.772211	2.746938	-2.488407
H	-4.196483	-1.169720	-1.943717	H	-3.942013	1.583655	-1.817672
P	0.033104	-0.057252	0.379023	H	-2.738438	2.346306	-0.762159
P	-1.704324	0.580352	-2.088981	C	1.018948	1.813443	2.171979
Fe	0.437012	0.825139	-1.604562	H	1.338054	2.379523	1.290766
C	-0.285025	1.037365	1.904054	H	0.855966	2.528684	2.989116
C	-2.133350	0.118562	-3.894748	H	1.837596	1.150919	2.475352
C	1.093411	-0.868326	-2.378598	C	-1.407597	2.027856	1.563382
C	0.854793	-2.252421	-2.216929	H	-2.343818	1.507991	1.330229
C	2.112065	-0.539038	-3.305564	H	-1.595201	2.682738	2.424629
C	1.564575	-3.229637	-2.918945	H	-1.132471	2.658524	0.714722
H	0.067176	-2.579586	-1.539401	C	-0.683695	0.246557	3.161445
C	2.834417	-1.503756	-4.020353	H	-0.816788	0.947827	3.996362
H	2.351312	0.510863	-3.500913	H	-1.631205	-0.284778	3.026340
C	2.563116	-2.859389	-3.827730	H	0.079073	-0.477312	3.466295
H	1.337086	-4.283375	-2.764818	C	-1.879502	-1.372215	-4.167097
H	3.604289	-1.196929	-4.726474	H	-2.041501	-1.574113	-5.234507
H	3.116666	-3.616925	-4.377860	H	-0.857091	-1.663427	-3.922824
C	0.697795	2.766742	-1.374497	H	-2.561137	-2.015111	-3.600569
C	-0.049956	3.964931	-1.407562	C	-1.162031	0.967289	-4.743188
C	2.106537	2.943120	-1.366672	H	-1.369136	0.814944	-5.810797
C	0.547155	5.230353	-1.416844	H	-1.267170	2.038873	-4.531347
H	-1.136185	3.917978	-1.413016	H	-0.120939	0.682267	-4.557252
C	2.724256	4.198816	-1.375409	C	-3.577382	0.464532	-4.306022
H	2.761341	2.063938	-1.327531	H	-3.728530	0.157029	-5.349374
C	1.939712	5.355104	-1.399528	H	-4.327427	-0.056316	-3.702267
H	-0.074805	6.124192	-1.437378	H	-3.782900	1.537740	-4.252487
H	3.810152	4.276702	-1.359079				

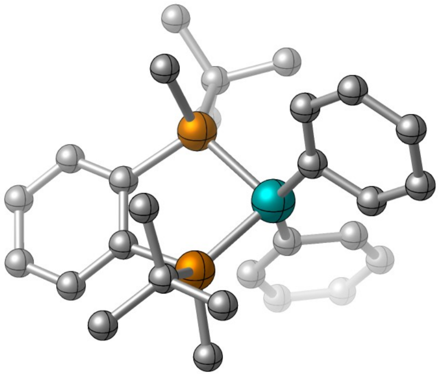

Zero-point correction= 0.588847 (Hartree/Particle)
Thermal correction to Energy= 0.623009
Thermal correction to Enthalpy= 0.623953
Thermal correction to Gibbs Free Energy= 0.526372
Sum of electronic and zero-point Energies= -3035.691018
Sum of electronic and thermal Energies= -3035.656856
Sum of electronic and thermal Enthalpies= -3035.655912
Sum of electronic and thermal Free Energies= -3035.753493

2

E(scf) = -489.312194093 a.u.

ν_{min} = 63.6cm^{-1}

C	1.616814	3.477865	-0.771316	H	5.907687	-0.835889	-3.919616
C	1.740740	2.655195	-1.822197	H	4.374815	-1.287876	-3.140307
C	4.641630	0.854576	-3.438987	C	6.614024	1.883206	-2.066925
C	5.234853	1.234987	-2.031436	H	6.936841	2.120527	-1.047516
B	3.088127	1.984017	-2.208079	H	7.348546	1.198693	-2.505648
O	4.269528	2.215928	-1.543429	H	6.610736	2.810471	-2.644854
O	3.213285	1.097237	-3.249817	C	5.219349	0.071786	-1.034319
C	5.097874	1.792326	-4.561307	H	5.964178	-0.687799	-1.292852
H	6.151862	1.634089	-4.811594	H	5.450226	0.456080	-0.035493
H	4.497499	1.596335	-5.455606	H	4.233914	-0.403963	-0.995249
H	4.958092	2.841868	-4.282253	H	2.467370	3.710723	-0.131958
C	4.840000	-0.599652	-3.850129	H	0.674069	3.952927	-0.501478
H	4.389112	-0.770672	-4.833569	H	0.858217	2.451650	-2.431862

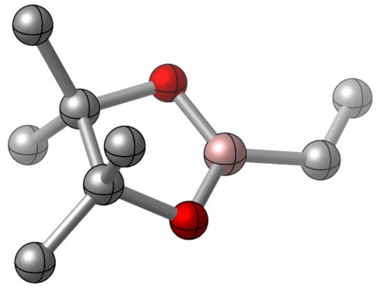

Zero-point correction= 0.225552 (Hartree/Particle)
Thermal correction to Energy= 0.237477
Thermal correction to Enthalpy= 0.238421
Thermal correction to Gibbs Free Energy= 0.188925
Sum of electronic and zero-point Energies= -489.086643
Sum of electronic and thermal Energies= -489.074717
Sum of electronic and thermal Enthalpies= -489.073773
Sum of electronic and thermal Free Energies= -489.123269

²*I*

E(scf) = -5144.74857822 a.u.

ν_{min} = 20.8 cm^{-1}

C	-3.056620	-2.272353	-0.240662	H	-0.155096	-1.560152	2.855774
C	-3.444873	-0.978114	-0.644323	C	-2.971884	-1.468816	-3.907279
P	-1.280851	-2.706523	-0.544737	H	-3.608432	-2.110710	-3.287478
P	-2.208177	-0.057275	-1.668083	H	-1.958788	-1.893714	-3.902154
Fe	-0.281102	-1.126093	-1.700343	H	-3.347182	-1.511465	-4.938032
C	-1.387708	-4.416048	-1.229167	C	-2.025954	0.836343	-4.272028
C	-0.529401	-2.903603	1.187932	H	-2.016598	1.886500	-3.958271
C	-2.973064	-0.013333	-3.402645	H	-2.352904	0.804424	-5.319690
C	-2.331060	1.667593	-1.029043	H	-0.996598	0.459914	-4.227049
C	-1.194713	-3.970609	2.067766	C	-4.394472	0.559978	-3.474144
H	-2.226450	-3.708650	2.320349	H	-4.720563	0.595099	-4.522738
H	-0.639542	-4.058236	3.011761	H	-4.448764	1.581349	-3.080724
H	-1.192292	-4.959229	1.594744	H	-5.112149	-0.059507	-2.927795
C	0.953938	-3.258286	0.968487	H	-3.335270	2.096742	-1.106838
H	1.483391	-3.258727	1.930342	H	-1.626934	2.304267	-1.573544
H	1.436603	-2.528525	0.308503	H	-2.032706	1.652059	0.024667
H	1.072996	-4.253008	0.522313	H	-1.842614	-4.351156	-2.223623
C	-0.632570	-1.525928	1.867774	H	-0.379474	-4.827569	-1.340974
H	-1.676072	-1.219785	2.005537	H	-1.984434	-5.098165	-0.615195
H	-0.129469	-0.751964	1.274747	Br	1.941667	-0.532515	-1.962749

C	-4.725589	-0.507006	-0.321137	C	-5.275212	-2.639178	0.678908
H	-5.024420	0.499893	-0.597141	H	-5.990406	-3.292446	1.172010
C	-5.634723	-1.331065	0.344617	C	-3.990858	-3.105256	0.391974
H	-6.626532	-0.958618	0.587459	H	-3.721841	-4.120088	0.667581

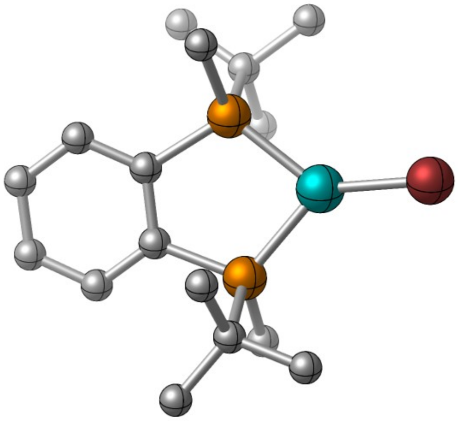

Zero-point correction= 0.408136 (Hartree/Particle)
Thermal correction to Energy= 0.433951
Thermal correction to Enthalpy= 0.434896
Thermal correction to Gibbs Free Energy= 0.352650
Sum of electronic and zero-point Energies= -5144.340443
Sum of electronic and thermal Energies= -5144.314627
Sum of electronic and thermal Enthalpies= -5144.313683
Sum of electronic and thermal Free Energies= -5144.395928

^{2}*IV^{R}*

E(scf) = -6023.64910366 a.u.

ν_{min} = 21.1 cm^{-1}

C	-2.436381	-0.701920	0.494030	C	0.558115	1.315199	0.521089
C	-2.947693	0.480719	-0.088587	C	1.938118	1.264932	0.809974
P	-0.810796	-1.300423	-0.144476	C	-0.273613	1.840671	1.524672
P	-1.942562	1.325981	-1.395318	C	2.455705	1.701560	2.031701
Fe	0.164982	0.625942	-1.278588	H	2.633764	0.911849	0.053304
C	-1.360366	-2.732895	-1.174448	C	0.235269	2.297805	2.746370
C	0.195035	-2.196283	1.212970	H	-1.348983	1.877742	1.387222
C	-2.874683	1.084649	-3.043516	C	1.603955	2.226928	3.008720
C	-2.201794	3.114737	-1.060466	H	3.526855	1.643607	2.215609

H	-0.444437	2.697398	3.496524
H	2.002418	2.575769	3.958493
C	-0.299918	-3.627546	1.505269
H	-1.325309	-3.654228	1.885849
H	0.345717	-4.064562	2.278287
H	-0.242483	-4.280882	0.630579
C	1.618578	-2.288091	0.621267
H	2.279696	-2.791775	1.338605
H	2.034346	-1.299494	0.412316
H	1.635794	-2.863518	-0.311465
C	0.222053	-1.407799	2.532678
H	-0.782036	-1.262216	2.944659
H	0.690517	-0.432323	2.421696
H	0.804795	-1.975498	3.270319
C	-3.070921	-0.414607	-3.307385
H	-3.647259	-0.896194	-2.509566
H	-2.109122	-0.920304	-3.415914
H	-3.624617	-0.545624	-4.245950
C	-2.018205	1.694101	-4.170029
H	-1.848495	2.767041	-4.028281
H	-2.546987	1.567698	-5.123802
H	-1.049430	1.194287	-4.252572
C	-4.253056	1.770602	-3.022626
H	-4.746117	1.583454	-3.985342
H	-4.183675	2.855621	-2.900373
H	-4.904325	1.368300	-2.240551
H	-3.259036	3.357874	-0.934578
H	-1.807075	3.686419	-1.901756
H	-1.664619	3.408242	-0.158687
H	-1.898580	-2.372596	-2.047438
H	-0.490942	-3.295135	-1.521578
H	-2.019653	-3.387763	-0.597194
Br	0.649027	-0.879030	-3.131786
C	-4.402477	-0.837533	1.919823
C	-4.896627	0.341420	1.360908
C	1.761342	5.953049	-1.106785
H	0.884075	6.056228	-0.454498
H	2.624966	5.726131	-0.468146
H	1.942158	6.927121	-1.580602
C	2.813523	4.736033	-3.024493
H	2.674795	4.032620	-3.850011
H	3.665098	4.387503	-2.426295
H	3.081959	5.709945	-3.455370
C	1.546095	4.849335	-2.160706
C	0.359787	5.249855	-3.055383
H	-0.548489	5.407674	-2.460255
H	0.570829	6.186556	-3.588209
H	0.143722	4.484435	-3.809698
C	1.256162	3.523878	-1.394386
C	1.052310	2.236565	-2.222142
C	4.460640	0.625067	-2.780656
C	4.034443	0.998460	-4.268050
B	2.388547	1.655918	-2.782263
O	2.688730	1.513027	-4.120276
O	3.451303	1.282437	-1.974932
C	4.373599	-0.871889	-2.458856
H	5.112940	-1.450270	-3.023095
H	4.570457	-1.016194	-1.390556
H	3.374162	-1.255801	-2.672155
C	5.826044	1.159597	-2.349527
H	6.001375	0.899572	-1.29981
H	6.628586	0.714419	-2.948092
H	5.88505	2.247113	-2.438743
C	3.967208	-0.192834	-5.224331
H	3.641318	0.153901	-6.211317
H	4.947122	-0.670145	-5.337539
H	3.242162	-0.930255	-4.8736
C	4.873211	2.118523	-4.892638
H	5.903736	1.79588	-5.076219
H	4.426511	2.402255	-5.851634
H	4.892551	3.008217	-4.257647
H	2.085855	3.364767	-0.696125
H	0.384554	3.713967	-0.759121
H	0.400245	2.449389	-3.077855
H	-4.961136	-1.351608	2.697238
H	-5.841062	0.756469	1.701962
C	-3.181314	-1.354352	1.486209
H	-2.809587	-2.268664	1.934516
C	-4.173665	0.993631	0.362019
H	-4.568687	1.91434	-0.052661

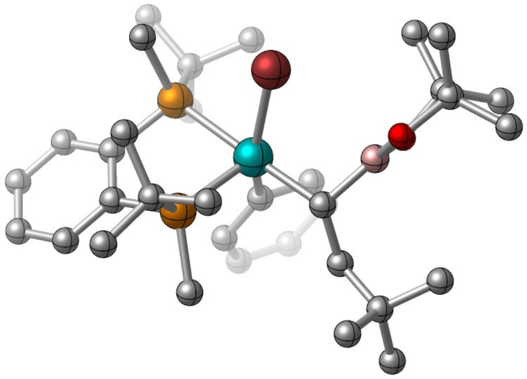

Zero-point correction= 0.855284 (Hartree/Particle)
Thermal correction to Energy= 0.903853
Thermal correction to Enthalpy= 0.904798
Thermal correction to Gibbs Free Energy= 0.777663
Sum of electronic and zero-point Energies= -6022.793820
Sum of electronic and thermal Energies= -6022.745250
Sum of electronic and thermal Enthalpies= -6022.744306
Sum of electronic and thermal Free Energies= -6022.871

^{2}*IV^{S}*
E(scf) = -6023.65049080 a.u.
ν_{min} = 13.6 cm^{-1}

C	-2.673122	-0.686718	0.469207	C	1.639140	1.701367	3.088446
C	-2.954485	0.501658	-0.241569	H	3.503937	0.789485	2.490057
P	-1.148135	-1.596949	-0.024665	H	-0.350813	2.483483	3.379277
P	-1.729159	1.104926	-1.501548	H	2.009126	2.037277	4.054212
Fe	0.231003	0.037908	-1.218478	C	-1.123983	-3.862201	1.777813
C	-1.922906	-2.966367	-0.993558	H	-2.160048	-3.696135	2.088029
C	-0.380520	-2.554380	1.439413	H	-0.606461	-4.347200	2.615870
C	-2.564052	0.969654	-3.211022	H	-1.126084	-4.571429	0.945529
C	-1.719428	2.920542	-1.215355	C	1.037257	-2.916854	0.946342
C	0.664127	0.816470	0.553564	H	1.553289	-3.502506	1.718428
C	1.990428	0.581312	0.970851	H	1.633711	-2.023178	0.744247
C	-0.151546	1.511342	1.465932	H	1.008821	-3.518865	0.030752
C	2.473794	1.002398	2.211677	C	-0.289190	-1.692989	2.709481
H	2.673308	0.045280	0.318490	H	-1.275225	-1.364270	3.054529
C	0.320561	1.952544	2.707259	H	0.333454	-0.812175	2.565214
H	-1.196509	1.694260	1.243058	H	0.161300	-2.292918	3.511432

C	-3.065059	-0.464681	-3.433634	C	1.520181	1.260976	-2.229940
H	-3.810735	-0.753442	-2.684042	C	1.493332	5.051275	-1.942805
H	-2.237201	-1.176460	-3.414256	C	2.333649	4.629879	-0.673840
H	-3.539660	-0.531418	-4.421309	B	1.659234	2.750057	-1.820809
C	-1.500981	1.322106	-4.271106	O	2.585199	3.223492	-0.910674
H	-1.052530	2.307132	-4.100718	O	0.934954	3.787318	-2.390706
H	-1.977056	1.337427	-5.260275	C	0.345514	6.018023	-1.660340
H	-0.702435	0.576718	-4.292187	H	0.726933	6.969109	-1.272076
C	-3.753171	1.939776	-3.342749	H	-0.197449	6.224808	-2.589355
H	-4.194062	1.810568	-4.339686	H	-0.364761	5.607243	-0.939251
H	-3.454315	2.988429	-3.253441	C	2.349823	5.588404	-3.094529
H	-4.540593	1.733823	-2.611426	H	1.724861	5.677628	-3.989536
H	-2.729007	3.315112	-1.083942	H	2.762756	6.575791	-2.862330
H	-1.239598	3.411455	-2.059966	H	3.175345	4.910388	-3.326692
H	-1.133812	3.143922	-0.324991	C	1.55294	4.726694	0.640733
H	-2.378447	-2.555129	-1.892310	H	2.133216	4.24416	1.432691
H	-1.165862	-3.691872	-1.296782	H	1.367573	5.767951	0.925606
H	-2.696996	-3.459356	-0.398024	H	0.596638	4.203265	0.573657
Br	0.400183	-1.686250	-2.987074	C	3.676142	5.342029	-0.527469
C	3.093063	0.014498	-4.719389	H	3.533837	6.421757	-0.405492
H	2.897087	-1.045431	-4.530442	H	4.193838	4.964865	0.361241
H	2.131784	0.481220	-4.960620	H	4.322677	5.169972	-1.391393
H	3.737734	0.098326	-5.605190	H	2.793327	-0.531909	-2.027334
C	4.024332	2.164367	-3.830040	H	1.087433	1.224299	-3.235242
H	4.454082	2.688120	-2.968214	H	3.487788	0.945298	-1.388666
H	3.098414	2.679543	-4.108075	C	-4.123343	1.217356	0.063771
H	4.721616	2.262153	-4.672795	H	-4.350436	2.144095	-0.450559
C	3.761159	0.683678	-3.506277	C	-5.005544	0.764809	1.044465
C	5.106730	-0.015806	-3.235347	H	-5.902171	1.336338	1.268182
H	4.957924	-1.076096	-2.991984	C	-4.730098	-0.414081	1.737987
H	5.765800	0.035128	-4.112234	H	-5.411674	-0.7714	2.505131
H	5.634728	0.450605	-2.393148	C	-3.571922	-1.134412	1.447661
C	2.893077	0.546238	-2.217812	H	-3.370625	-2.0494	1.993196

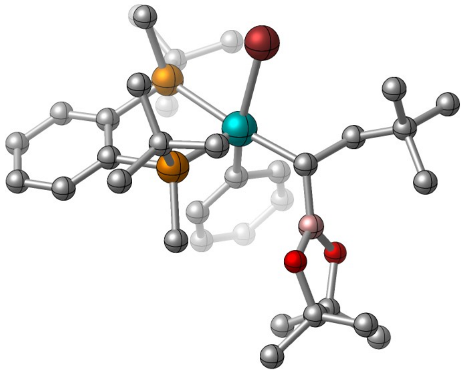

Zero-point correction= 0.855564 (Hartree/Particle)
Thermal correction to Energy= 0.904033
Thermal correction to Enthalpy= 0.904977
Thermal correction to Gibbs Free Energy= 0.777682
Sum of electronic and zero-point Energies= -6022.794927
Sum of electronic and thermal Energies= -6022.746458
Sum of electronic and thermal Enthalpies= -6022.745514
Sum of electronic and thermal Free Energies= -6022.872809

^{2}TS$^{R}_{III\text{-}IV}$

E(scf) = -6023.62140545 a.u.

ν_{min} = -90.5 cm^{-1}

C	-2.519301	-0.753550	0.464133
C	-2.934944	0.457717	-0.143568
P	-0.888762	-1.450818	-0.056989
P	-1.866069	1.221503	-1.456722
Fe	0.145666	0.350475	-1.260463
C	-1.371996	-2.928228	-1.052790
C	0.015777	-2.249439	1.418408
C	-2.786156	0.927920	-3.101271
C	-2.081796	3.033797	-1.205115
C	0.614641	1.297644	0.372635
C	2.017337	1.240656	0.497050
C	-0.094229	1.927514	1.408804
C	2.682057	1.782250	1.600691
H	2.622921	0.802938	-0.297614
C	0.560570	2.483611	2.513456
H	-1.180260	1.962060	1.384858
C	1.952386	2.412071	2.613829
H	3.766952	1.725605	1.662755
H	-0.017509	2.963594	3.300717
H	2.463524	2.842227	3.471814
C	-0.514890	-3.642680	1.807057
H	-1.559700	-3.625429	2.131659
H	0.080636	-4.023831	2.647353
H	-0.423952	-4.365842	0.991230
C	1.470370	-2.399656	0.919607
H	2.076181	-2.881941	1.698064
H	1.918610	-1.427647	0.692832
H	1.531873	-3.018663	0.016634
C	-0.012489	-1.329633	2.649143
H	-1.031913	-1.161122	3.012914
H	0.439717	-0.358984	2.441926
H	0.558209	-1.799724	3.461127

C	-2.926817	-0.584176	-3.328188	H	0.179254	5.146170	-3.662851
H	-3.505660	-1.060027	-2.528493	C	1.419706	3.821758	-1.499685
H	-1.946746	-1.062393	-3.394899	C	1.265952	2.776330	-2.566320
H	-3.453040	-0.763343	-4.274869	C	4.579292	1.063409	-3.123991
C	-1.922654	1.537023	-4.223228	C	4.015231	1.183244	-4.601807
H	-1.836757	2.625421	-4.131411	B	2.498258	2.042895	-3.110858
H	-2.393857	1.327076	-5.192639	O	2.638668	1.598533	-4.405862
H	-0.916901	1.106131	-4.237797	O	3.643077	1.861696	-2.354396
C	-4.185558	1.566995	-3.122125	C	4.526708	-0.357325	-2.550762
H	-4.650927	1.361752	-4.095367	H	5.234428	-1.022501	-3.057251
H	-4.155323	2.654392	-3.001199	H	4.793924	-0.319723	-1.488653
H	-4.840601	1.147158	-2.352168	H	3.521187	-0.774413	-2.635456
H	-3.130498	3.338115	-1.168010	C	5.976663	1.646561	-2.921753
H	-1.596182	3.557369	-2.031320	H	6.26048	1.55226	-1.867847
H	-1.597468	3.336355	-0.276128	H	6.716329	1.105194	-3.522244
H	-1.819512	-2.607356	-1.991893	H	6.018289	2.705909	-3.186618
H	-0.473750	-3.504550	-1.290248	C	4.00202	-0.127152	-5.385399
H	-2.084452	-3.561202	-0.514768	H	3.598025	0.050493	-6.388303
Br	0.837120	-0.995674	-3.107860	H	5.013862	-0.534008	-5.492791
C	-4.538142	-0.751615	1.826948	H	3.363965	-0.864289	-4.893312
C	-4.934944	0.453949	1.248180	C	4.685976	2.289197	-5.424078
C	1.884793	6.185648	-0.814689	H	5.727396	2.046111	-5.660168
H	1.038927	6.149124	-0.115888	H	4.139084	2.412529	-6.364843
H	2.783556	5.876400	-0.265982	H	4.660564	3.246537	-4.893571
H	2.021516	7.230676	-1.122449	H	2.275852	3.564418	-0.867068
C	2.861767	5.332008	-2.963756	H	0.546184	3.845623	-0.837808
H	2.692055	4.752971	-3.879148	H	0.382855	2.843332	-3.196064
H	3.751317	4.922041	-2.470076	H	-5.154503	-1.22514	2.586431
H	3.082106	6.366250	-3.258658	H	-5.860971	0.932228	1.555766
C	1.638726	5.276432	-2.031387	C	-3.339128	-1.347163	1.434984
C	0.390984	5.768079	-2.785113	H	-3.041284	-2.280064	1.899874
H	-0.494307	5.750720	-2.136373	C	-4.139139	1.049096	0.269017
H	0.527432	6.799470	-3.135667	H	-4.464869	1.98654	-0.168644

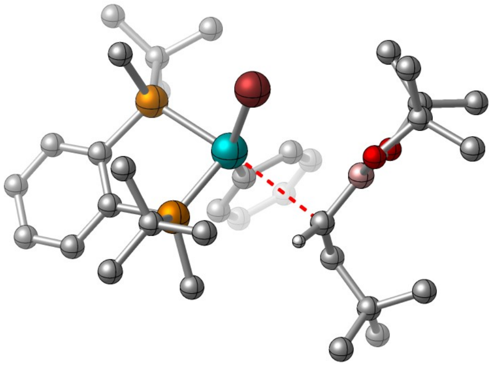

Zero-point correction= 0.851541 (Hartree/Particle)
Thermal correction to Energy= 0.900736
Thermal correction to Enthalpy= 0.901680
Thermal correction to Gibbs Free Energy= 0.771293
Sum of electronic and zero-point Energies= -6022.769864
Sum of electronic and thermal Energies= -6022.720670
Sum of electronic and thermal Enthalpies= -6022.719725
Sum of electronic and thermal Free Energies= -6022.850113

$^2TS^R_{IV\text{-}I}$

E(scf) = -6023.60906523 a.u.

ν_{min} = -230.3 cm^{-1}

C	-2.454424	-0.527351	0.182738
C	-2.796053	0.782028	-0.219417
P	-0.699444	-1.053360	-0.082565
P	-1.655751	1.646006	-1.383776
Fe	0.484783	0.760666	-1.122532
C	-0.821695	-2.589085	-1.100357
C	-0.096279	-1.727128	1.600278
C	-2.485620	1.253683	-3.074179
C	-2.069887	3.435101	-1.144009
C	1.334607	1.816927	0.362076
C	2.498845	1.309153	1.011515
C	0.479819	2.609629	1.182239
C	2.735589	1.505141	2.367084
H	3.217749	0.746405	0.423612
C	0.721993	2.803035	2.541569
H	-0.374187	3.098662	0.736659
C	1.849827	2.250781	3.156098
H	3.628570	1.072673	2.814492
H	0.018660	3.395840	3.123583
H	2.041365	2.406804	4.214177
C	-0.749341	-3.050644	2.039664
H	-1.805999	-2.939469	2.293134
H	-0.241014	-3.399284	2.948935
H	-0.649053	-3.841934	1.289873
C	1.418713	-1.964310	1.459987
H	1.815006	-2.306405	2.425930
H	1.939557	-1.049022	1.182884
H	1.650952	-2.727523	0.710951
C	-0.355710	-0.649741	2.663073
H	-1.427520	-0.480387	2.816305
H	0.105384	0.299150	2.389600
H	0.074037	-0.968081	3.622042

C	-2.167997	-0.205337	-3.433194
H	-2.615731	-0.899822	-2.714876
H	-1.087172	-0.386484	-3.462616
H	-2.576856	-0.441393	-4.424382
C	-1.877277	2.178372	-4.142415
H	-1.996116	3.240282	-3.899912
H	-2.382476	2.001487	-5.101094
H	-0.818022	1.973038	-4.293074
C	-4.011984	1.442029	-3.058901
H	-4.400010	1.257534	-4.069530
H	-4.310886	2.457787	-2.778462
H	-4.507956	0.740796	-2.382420
H	-2.974374	3.732671	-1.682046
H	-1.231119	4.047116	-1.481516
H	-2.225392	3.633730	-0.080278
H	-0.911621	-2.329364	-2.155458
H	0.098352	-3.165400	-0.981522
H	-1.679063	-3.200690	-0.802059
Br	2.167668	-0.874378	-1.915672
C	1.667286	2.663090	-5.213921
H	1.279899	1.650041	-5.381507
H	0.827662	3.365938	-5.280258
H	2.360318	2.892875	-6.033210
C	2.908267	4.198571	-3.673997
H	3.516051	4.289382	-2.767645
H	2.087382	4.921577	-3.607967
H	3.545264	4.480616	-4.521880
C	2.382985	2.763403	-3.853592
C	3.573792	1.790894	-3.860832
H	3.244741	0.758208	-3.999510
H	4.262226	2.055290	-4.674429
H	4.140443	1.826829	-2.924477
C	1.331132	2.422992	-2.742496
C	1.867459	2.504720	-1.293741
C	1.167630	6.130202	-0.351663
C	2.673279	5.949021	0.124630
B	1.891616	3.987583	-0.784601
O	2.911120	4.523246	-0.042577
O	0.906137	4.899297	-1.084623
C	0.153564	6.192696	0.794359
H	0.258967	7.119844	1.367048
H	-0.858138	6.160975	0.376070
H	0.266217	5.345909	1.473758
C	0.931345	7.298228	-1.306514
H	-0.120848	7.310788	-1.610927
H	1.155015	8.252613	-0.817581
H	1.541346	7.215745	-2.209113
C	2.92411	6.292454	1.591512
H	3.978056	6.114781	1.830443
H	2.702293	7.346546	1.79107
H	2.321521	5.67016	2.257149
C	3.69441	6.672524	-0.758588
H	3.616358	7.759425	-0.651271
H	4.701899	6.368233	-0.456465
H	3.568762	6.416274	-1.813724
H	0.49328	3.112918	-2.859769
H	0.944694	1.419165	-3.019931
C	-4.040666	1.315588	0.149707
H	-4.306466	2.326528	-0.142816
C	-4.96321	0.548729	0.859672
H	-5.920984	0.977144	1.142856
C	-4.666716	-0.780739	1.168518
H	-5.39797	-1.400614	1.680429
C	-3.422378	-1.311626	0.830404
H	-3.209992	-2.345414	1.074521
H	2.876046	2.095971	-1.273561

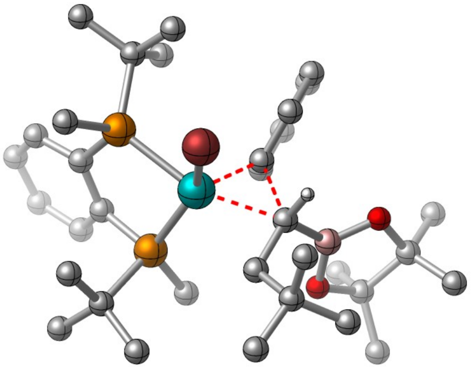

Zero-point correction= 0.854408 (Hartree/Particle)
Thermal correction to Energy= 0.902625
Thermal correction to Enthalpy= 0.903569
Thermal correction to Gibbs Free Energy= 0.776657
Sum of electronic and zero-point Energies= -6022.754657
Sum of electronic and thermal Energies= -6022.706440
Sum of electronic and thermal Enthalpies= -6022.705496
Sum of electronic and thermal Free Energies= -6022.832408

^{2}TS^{S}$_{III\text{-}IV}$

E(scf) = -6023.61672384 a.u.

ν_{min} = -32.1 cm^{-1}

C	-2.842480	-0.635052	0.448709
C	-3.022142	0.686123	-0.030500
P	-1.206856	-1.443120	0.153951
P	-1.690922	1.451090	-1.078064
Fe	0.196898	0.289527	-0.766972
C	-1.618901	-2.820536	-1.003300
C	-0.657196	-2.356403	1.731049
C	-2.384395	1.410171	-2.851122
C	-1.774618	3.248447	-0.665645
C	0.705655	1.165585	0.899741
C	2.095317	1.001490	1.076612
C	0.013495	1.845109	1.918019
C	2.766073	1.507949	2.194888
H	2.683373	0.460143	0.332924
C	0.671438	2.361027	3.039170
H	-1.067154	1.951469	1.858649
C	2.053553	2.199770	3.177901
H	3.839887	1.365987	2.296358
H	0.105184	2.881579	3.808919
H	2.567988	2.601765	4.047356
C	-1.331108	-3.722653	1.949770
H	-2.414212	-3.643583	2.084369
H	-0.923582	-4.177100	2.862954
H	-1.137964	-4.417598	1.126541
C	0.857502	-2.576930	1.523236
H	1.262612	-3.166481	2.356331
H	1.397345	-1.625456	1.485779
H	1.067720	-3.121497	0.594297
C	-0.882634	-1.466454	2.963525
H	-1.948057	-1.306686	3.161805
H	-0.405991	-0.489261	2.851456
H	-0.446707	-1.952564	3.846607

C	-2.584689	-0.056521	-3.264034	C	2.485446	2.424317	-2.103758
H	-3.302575	-0.565271	-2.610475	C	3.788307	1.448980	-5.486924
H	-1.638288	-0.602905	-3.245814	C	2.333801	1.949570	-5.835559
H	-2.976657	-0.097206	-4.288846	B	2.700658	2.075882	-3.578787
C	-1.317132	2.058613	-3.752975	O	1.920089	2.592143	-4.597023
H	-1.221630	3.134105	-3.570544	O	3.737385	1.293466	-4.043063
H	-1.609579	1.932988	-4.803860	C	4.171513	0.108411	-6.105123
H	-0.332288	1.603426	-3.623234	H	4.146314	0.163233	-7.199378
C	-3.717440	2.161533	-2.997750	H	5.189241	-0.160268	-5.801351
H	-4.030381	2.126099	-4.050018	H	3.499999	-0.688151	-5.776444
H	-3.637428	3.217186	-2.718250	C	4.872243	2.494896	-5.774469
H	-4.514435	1.703044	-2.403677	H	5.816882	2.157298	-5.335414
H	-2.797374	3.631182	-0.630433	H	5.023098	2.638261	-6.849620
H	-1.225012	3.809267	-1.423181	H	4.617378	3.460612	-5.325312
H	-1.299835	3.428821	0.299683	C	1.34797	0.807784	-6.105091
H	-1.879168	-2.405119	-1.977602	H	0.33802	1.221386	-6.189212
H	-0.732671	-3.448198	-1.134539	H	1.584642	0.289411	-7.040462
H	-2.451165	-3.431542	-0.640217	H	1.347231	0.087574	-5.282981
Br	1.084619	-0.891095	-2.639356	C	2.2609	2.979763	-6.958751
C	3.257100	4.652349	-0.087393	H	2.634472	2.556269	-7.897936
H	2.712379	4.270203	0.784772	H	1.218927	3.280933	-7.113034
H	4.050234	3.934240	-0.325833	H	2.83935	3.876965	-6.724897
H	3.737992	5.596301	0.200578	H	1.043175	3.209581	-0.74342
C	3.103768	5.381957	-2.493621	H	3.111408	1.951547	-1.350252
H	2.454957	5.494518	-3.371185	H	0.847509	3.74049	-2.418773
H	3.910296	4.689818	-2.764268	C	-4.222399	1.355571	0.258327
H	3.556925	6.358317	-2.278249	H	-4.37799	2.372335	-0.085549
C	2.311117	4.864959	-1.281487	C	-5.239356	0.732907	0.981863
C	1.232688	5.891610	-0.895228	H	-6.158004	1.272597	1.196068
H	0.642105	5.540824	-0.039260	C	-5.076894	-0.582548	1.418182
H	1.685414	6.852786	-0.618837	H	-5.870299	-1.080506	1.96914
H	0.542106	6.075118	-1.728629	C	-3.886146	-1.256862	1.150611
C	1.589087	3.524745	-1.640706	H	-3.771255	-2.278046	1.496462

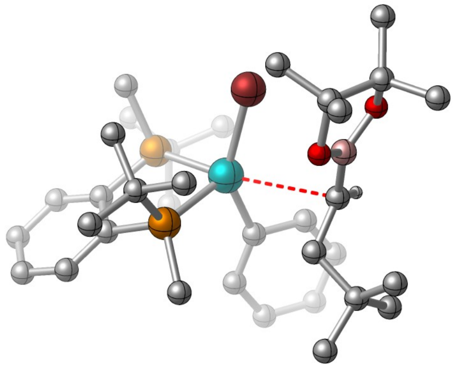

Zero-point correction= 0.850310 (Hartree/Particle)
Thermal correction to Energy= 0.900166
Thermal correction to Enthalpy= 0.901111
Thermal correction to Gibbs Free Energy= 0.767046
Sum of electronic and zero-point Energies= -6022.766414
Sum of electronic and thermal Energies= -6022.716557
Sum of electronic and thermal Enthalpies= -6022.715613
Sum of electronic and thermal Free Energies= -6022.849678

$^{2}TS^{S}_{IV\text{-}I}$

E(scf) = -6023.61873431 a.u.

ν_{min} = -271.9 cm^{-1}

C	-2.710398	-0.740488	0.211488	C	2.196373	1.737670	3.449366
C	-2.986745	0.587881	-0.178030	H	3.778600	0.334081	3.010457
P	-0.958067	-1.311748	0.108195	H	0.547209	3.128999	3.538092
P	-1.647855	1.567001	-1.001561	H	2.474354	1.859386	4.492933
Fe	0.411845	0.508318	-0.595732	C	-1.315918	-3.193393	2.295141
C	-1.096573	-2.818556	-0.941813	H	-2.345695	-2.914509	2.533608
C	-0.482293	-1.992399	1.818185	H	-0.867488	-3.584086	3.218519
C	-2.096973	1.458009	-2.855086	H	-1.331361	-4.013061	1.569191
C	-2.082672	3.289394	-0.521896	C	0.992563	-2.425065	1.700784
C	1.461689	1.396194	0.721258	H	1.356282	-2.733941	2.689658
C	2.582810	0.730836	1.271829	H	1.625249	-1.607406	1.350732
C	0.768554	2.289746	1.570191	H	1.120232	-3.272372	1.018402
C	2.929250	0.886245	2.613281	C	-0.611317	-0.846163	2.833095
H	3.170695	0.065124	0.648519	H	-1.646968	-0.494691	2.914596
C	1.116961	2.443723	2.914062	H	0.019141	0.002200	2.562647
H	-0.047347	2.880258	1.171633	H	-0.296711	-1.195827	3.825011

C	-2.074523	-0.024461	-3.266790
H	-2.844201	-0.604357	-2.745802
H	-1.097643	-0.479103	-3.069376
H	-2.265940	-0.104763	-4.344902
C	-1.006381	2.214290	-3.638761
H	-0.823636	3.222207	-3.251015
H	-1.309990	2.302445	-4.689998
H	-0.059889	1.665868	-3.617922
C	-3.474214	2.058412	-3.174813
H	-3.682877	1.915888	-4.243379
H	-3.515638	3.134693	-2.977636
H	-4.278399	1.569067	-2.616698
H	-3.065346	3.600103	-0.885264
H	-1.321813	3.957511	-0.921066
H	-2.080924	3.360786	0.570099
H	-1.299823	-2.516049	-1.970680
H	-0.151005	-3.363016	-0.932481
H	-1.903332	-3.470673	-0.593753
Br	1.635483	-1.090969	-2.021040
C	5.477973	1.800399	-2.698324
H	5.981124	2.354729	-1.895174
H	5.587444	0.729133	-2.485048
H	6.010326	2.011695	-3.635124
C	3.359959	1.428874	-3.977248
H	2.300749	1.683339	-4.099018
H	3.414414	0.347462	-3.823266
H	3.872356	1.678487	-4.916465
C	3.991672	2.193494	-2.800650
C	3.892113	3.701335	-3.081568
H	4.270144	4.292194	-2.239936
H	4.468044	3.970601	-3.977027
H	2.852941	3.996542	-3.262810
C	3.323758	1.808869	-1.444602
C	1.844095	2.174819	-1.266081
C	0.943932	5.880499	-1.096447
C	2.016332	5.738348	0.056691
B	1.653829	3.684179	-0.927704
O	2.532033	4.396372	-0.140963
O	0.649111	4.492487	-1.432483
C	-0.355059	6.569996	-0.683326
H	-0.160458	7.607616	-0.390593
H	-1.049675	6.581967	-1.530322
H	-0.846362	6.062320	0.149384
C	1.483272	6.541024	-2.368872
H	0.743903	6.426204	-3.168585
H	1.661215	7.610392	-2.215047
H	2.415077	6.079026	-2.701348
C	1.412741	5.788513	1.46398
H	2.177802	5.49244	2.188325
H	1.068134	6.797581	1.713653
H	0.574677	5.095707	1.56552
C	3.185676	6.716201	-0.033241
H	2.835921	7.750743	0.055665
H	3.886015	6.521924	0.786131
H	3.730851	6.610446	-0.974104
H	3.450624	0.72853	-1.341664
H	3.903558	2.277506	-0.640003
H	1.284028	1.922945	-2.16945
C	-4.288528	1.090593	-0.014292
H	-4.516586	2.117016	-0.280037
C	-5.313379	0.281504	0.474271
H	-6.31375	0.689022	0.593256
C	-5.056159	-1.055691	0.782384
H	-5.856945	-1.702756	1.130155
C	-3.763491	-1.559863	0.649766
H	-3.580049	-2.602061	0.884084

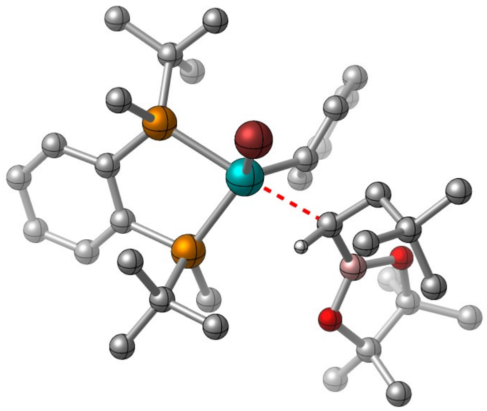

Zero-point correction= 0.853441 (Hartree/Particle)
Thermal correction to Energy= 0.901932
Thermal correction to Enthalpy= 0.902877
Thermal correction to Gibbs Free Energy= 0.774867
Sum of electronic and zero-point Energies= -6022.765293
Sum of electronic and thermal Energies= -6022.716802
Sum of electronic and thermal Enthalpies= -6022.715858
Sum of electronic and thermal Free Energies= -6022.843868

^{3}A

E(scf) = -7716.57681282 a.u.

ν_{min} = 20.7 cm^{-1}

Br	9.684147	6.313932	14.475253	C	5.461745	7.014389	12.466492
Br	8.883517	9.831134	13.237170	C	5.583192	5.630850	13.133788
Fe	7.960040	7.972781	14.427114	H	6.582036	5.467520	13.556031
P	5.845185	8.325438	13.779114	H	5.409406	4.850404	12.382513
P	7.031237	7.376148	16.377120	H	4.848139	5.499046	13.935233
C	4.654766	8.027243	15.144077	C	6.531522	7.155485	11.364439
C	3.276340	8.267666	15.063084	H	6.522230	8.150784	10.907163
H	2.851385	8.736701	14.181093	H	6.336636	6.418830	10.574856
C	2.439366	7.898029	16.116998	H	7.542455	6.972471	11.747236
H	1.371051	8.083772	16.048522	C	4.060696	7.181034	11.860066
C	2.970998	7.278017	17.251086	H	3.273245	7.063947	12.611297
H	2.315704	6.972644	18.062142	H	3.907490	6.404720	11.098958
C	4.346234	7.062753	17.352998	H	3.935248	8.152020	11.369115
H	4.749471	6.593888	18.245055	C	5.365734	9.943338	13.068343
C	5.197879	7.459007	16.312732	H	5.601795	10.717523	13.804404

H	4.300644	9.987820	12.825000	H	7.502079	7.382281	19.420537
H	5.959679	10.133384	12.172095	C	9.101708	8.749128	17.602824
C	7.561470	8.677971	17.646979	H	9.565268	7.786292	17.843599
C	6.967062	10.030542	17.210057	H	9.450773	9.485329	18.337801
H	7.289260	10.311190	16.199966	H	9.471272	9.061436	16.619000
H	7.309484	10.814527	17.896968	C	7.411169	5.738518	17.103751
H	5.871918	10.019731	17.227929	H	7.146975	4.974369	16.366874
C	7.095870	8.337143	19.070109	H	6.854764	5.557577	18.027808
H	6.004733	8.303167	19.147058	H	8.483904	5.669493	17.295756
H	7.449379	9.117489	19.756800				

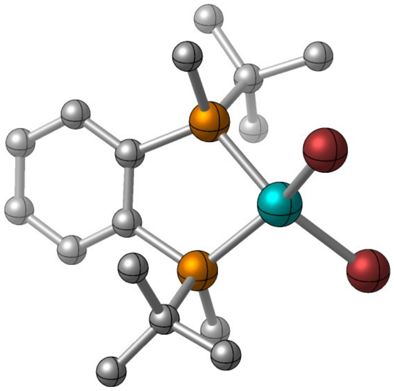

Zero-point correction= 0.410661 (Hartree/Particle)
Thermal correction to Energy= 0.438345
Thermal correction to Enthalpy= 0.439289
Thermal correction to Gibbs Free Energy= 0.351397
Sum of electronic and zero-point Energies= -7716.166152
Sum of electronic and thermal Energies= -7716.138468
Sum of electronic and thermal Enthalpies= -7716.137524
Sum of electronic and thermal Free Energies= -7716.225416

^{3}B

E(scf) = -5376.44652523 a.u.

ν_{min} = 24.7 cm^{-1}

C	-2.030866	1.377428	0.420729	C	-2.872804	2.427037	0.816968
C	-2.597555	0.141601	0.040947	H	-4.436263	-0.924186	-0.355140
C	-3.991950	0.009813	-0.025607	H	-5.899364	0.959364	0.288170
C	-4.819841	1.072324	0.338893	H	-4.901601	3.093544	1.090363
C	-4.259592	2.272202	0.783698	H	-2.454597	3.373987	1.143178

P	-1.414754	-1.237189	-0.253005	H	1.699737	3.349064	-2.460567
P	-0.198110	1.535454	0.246173	H	2.073730	3.172347	-0.736447
C	-2.044262	-2.122103	-1.729535	H	1.974432	1.739337	-1.770528
H	-1.417841	-3.000508	-1.901733	C	-0.574832	4.022285	-1.141904
H	-3.091762	-2.423938	-1.637458	H	-0.399998	4.590285	-2.065159
H	-1.932973	-1.457297	-2.591885	H	-1.655881	3.995155	-0.974814
C	0.313657	2.573994	1.671219	H	-0.107208	4.578601	-0.322419
H	1.387308	2.765194	1.602152	C	-0.662470	1.894047	-2.480830
H	-0.222984	3.525449	1.717949	H	-0.241121	0.895267	-2.645368
H	0.126332	2.017215	2.594519	H	-1.741473	1.788377	-2.324340
C	0.021150	2.615984	-1.303495	H	-0.510180	2.475577	-3.399014
C	-1.650128	-2.407958	1.219957	Fe	0.767884	-0.457312	-0.104477
C	-3.070992	-2.976794	1.336378	C	2.524176	0.058648	0.631449
H	-3.111757	-3.673368	2.184565	C	2.637587	0.158676	2.036366
H	-3.812070	-2.192889	1.520186	C	3.712943	0.274619	-0.096056
H	-3.366682	-3.533051	0.439971	C	3.847561	0.449329	2.677497
C	-0.640369	-3.558804	1.039845	H	1.753071	0.014208	2.659782
H	-0.711602	-4.242285	1.895885	C	4.925336	0.584488	0.527551
H	-0.834970	-4.138183	0.130792	H	3.690252	0.184392	-1.179856
H	0.389701	-3.189239	0.983695	C	5.001169	0.672359	1.921581
C	-1.298903	-1.612872	2.492029	H	3.887943	0.509133	3.764121
H	-0.275910	-1.216566	2.451051	H	5.817979	0.748943	-0.074411
H	-1.982599	-0.771398	2.649542	H	5.944322	0.907986	2.409692
H	-1.363611	-2.270233	3.368377	Br	1.598356	-1.946238	-1.807064
C	1.533172	2.721057	-1.575801				

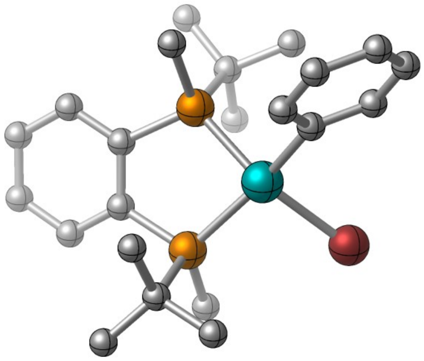

Zero-point correction= 0.499545 (Hartree/Particle)
Thermal correction to Energy= 0.530568
Thermal correction to Enthalpy= 0.531512
Thermal correction to Gibbs Free Energy= 0.437892
Sum of electronic and zero-point Energies= -5375.946980
Sum of electronic and thermal Energies= -5375.915958
Sum of electronic and thermal Enthalpies= -5375.915013
Sum of electronic and thermal Free Energies= -5376.008633

^{3}C

E(scf) = -3036.31162011 a.u.

ν_{min} = 17.4 cm^{-1}

C	-2.072043	-0.628886	0.588882	H	1.356068	2.408497	1.279908
C	-2.696572	-1.233159	1.691222	H	0.573328	3.218894	2.649302
C	-2.767960	-0.531447	-0.634605	C	-1.147253	-1.075135	-4.573776
C	-4.110817	-0.936425	-0.695481	H	-1.931129	-0.524565	-5.106006
C	-4.016981	-1.673693	1.604958	H	-0.256902	-0.442255	-4.530559
H	-4.490918	-2.136422	2.466530	H	-0.903996	-1.963398	-5.171730
C	-4.734327	-1.497901	0.418618	C	-2.900945	-2.346470	-3.317321
H	-5.772382	-1.813432	0.356109	H	-2.715966	-3.176656	-4.012216
H	-2.160009	-1.343611	2.628361	H	-3.222903	-2.778406	-2.365413
H	-4.670048	-0.833249	-1.619940	H	-3.728098	-1.756010	-3.727204
P	-0.404264	0.160693	0.667017	C	-0.523171	-2.380501	-2.508500
P	-1.801915	0.040646	-2.103023	H	-0.792494	-2.642368	-1.478003
Fe	0.290648	0.789119	-1.447538	H	-0.387111	-3.314308	-3.069366
C	-0.682406	1.715032	1.728985	H	0.443214	-1.861428	-2.486752
C	-1.613465	-1.520897	-3.175371	C	0.694812	1.673944	-3.169437
C	-2.984292	1.113666	-3.017407	C	-0.016659	2.851522	-3.501858
H	-2.478098	1.540373	-3.886748	C	1.680968	1.284474	-4.104088
H	-3.877365	0.575106	-3.349054	C	0.227969	3.583330	-4.669562
H	-3.290901	1.934677	-2.361059	H	-0.799450	3.209851	-2.830499
C	0.578733	-0.978256	1.726404	C	1.922626	1.989698	-5.287519
H	1.607382	-0.613173	1.778500	H	2.286668	0.402129	-3.897129
H	0.178073	-1.071221	2.740493	C	1.198045	3.150238	-5.578024
H	0.588976	-1.968421	1.259186	H	-0.343768	4.487530	-4.874964
C	-1.398149	2.738692	0.828101	H	2.687970	1.641236	-5.979809
H	-2.349254	2.344601	0.449900	H	1.388548	3.707589	-6.492527
H	-1.614028	3.652072	1.397500	C	2.179882	1.064922	-0.930881
H	-0.780248	3.021586	-0.033296	C	2.859640	2.303811	-0.887756
C	-1.521895	1.468927	2.991614	C	2.972638	-0.065500	-0.620800
H	-1.562858	2.396676	3.578000	C	4.209059	2.415611	-0.537841
H	-2.550536	1.181905	2.755111	H	2.316828	3.213613	-1.144328
H	-1.086053	0.695622	3.634231	C	4.330383	0.024586	-0.292701
C	0.699931	2.254025	2.140661	H	2.515043	-1.056634	-0.624916
H	1.207632	1.578034	2.837891	C	4.958109	1.272378	-0.240805

H	4.682599	3.396166	-0.510101	H	6.010520	1.352703	0.022280
H	4.897052	-0.878535	-0.069369				

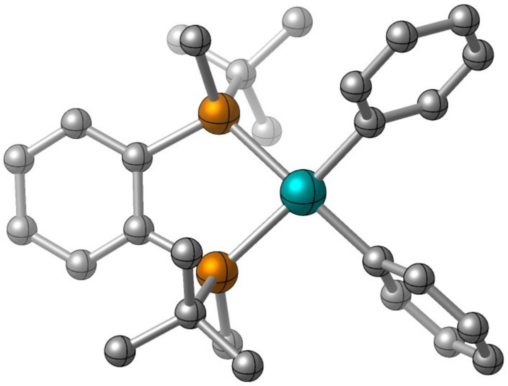

Zero-point correction= 0.587446 (Hartree/Particle)
Thermal correction to Energy= 0.622132
Thermal correction to Enthalpy= 0.623076
Thermal correction to Gibbs Free Energy= 0.520877
Sum of electronic and zero-point Energies= -3035.724174
Sum of electronic and thermal Energies= -3035.689488
Sum of electronic and thermal Enthalpies= -3035.688544
Sum of electronic and thermal Free Energies= -3035.790743

^{4}*I*

E(scf) = -5144.77620597 a.u.

ν_{min} = 27.5 cm^{-1}

C	-3.054468	-2.212337	-0.228763	C	0.928087	-3.381577	0.930233
C	-3.419509	-0.900069	-0.607930	H	1.463322	-3.417288	1.888334
P	-1.301495	-2.732887	-0.553794	H	1.443602	-2.670809	0.274392
P	-2.206742	0.063340	-1.631845	H	0.995442	-4.377214	0.475727
Fe	-0.201537	-1.062765	-1.719782	C	-0.564816	-1.580221	1.854498
C	-1.478784	-4.435977	-1.238215	H	-1.591550	-1.230135	2.012277
C	-0.534540	-2.959148	1.169698	H	-0.040430	-0.827629	1.253111
C	-2.930778	-0.025778	-3.388069	H	-0.071432	-1.639206	2.833491
C	-2.471393	1.807048	-1.091937	C	-2.705716	-1.470883	-3.873366
C	-1.236305	-3.995696	2.057777	H	-3.234081	-2.192857	-3.239938
H	-2.250551	-3.685438	2.326027	H	-1.637376	-1.734126	-3.869786
H	-0.671923	-4.110658	2.993271	H	-3.072470	-1.587632	-4.901528
H	-1.287690	-4.982505	1.583690	C	-2.107766	0.944011	-4.257265

H	-2.274839	1.989153	-3.973557	H	-0.485140	-4.884977	-1.333470
H	-2.400974	0.835304	-5.309896	H	-2.110771	-5.094453	-0.633810
H	-1.032839	0.738534	-4.183324	Br	2.086216	-0.591510	-1.863839
C	-4.420256	0.328309	-3.486894	C	-4.691329	-0.415099	-0.266345
H	-4.733194	0.296936	-4.539606	H	-4.977897	0.596933	-0.535745
H	-4.629681	1.337097	-3.113123	C	-5.611201	-1.227142	0.398189
H	-5.045660	-0.379559	-2.934343	H	-6.593620	-0.837695	0.652353
H	-3.486629	2.181178	-1.261077	C	-5.274030	-2.545026	0.714126
H	-1.762566	2.446840	-1.625847	H	-5.995314	-3.191145	1.207778
H	-2.246268	1.869578	-0.021784	C	-4.003129	-3.030827	0.403784
H	-1.912862	-4.358826	-2.241007	H	-3.752880	-4.054721	0.662786

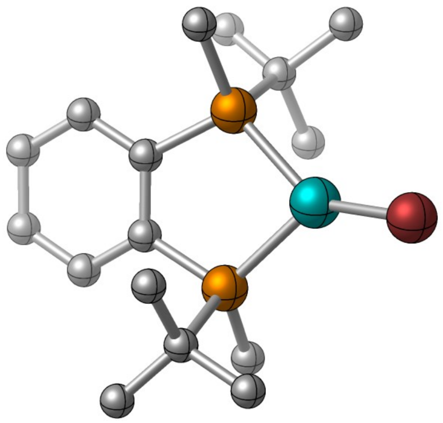

Zero-point correction= 0.408287 (Hartree/Particle)
Thermal correction to Energy= 0.434076
Thermal correction to Enthalpy= 0.435021
Thermal correction to Gibbs Free Energy= 0.352690
Sum of electronic and zero-point Energies= -5144.367919
Sum of electronic and thermal Energies= -5144.342130
Sum of electronic and thermal Enthalpies= -5144.341185
Sum of electronic and thermal Free Energies= -5144.423516

$^{4}IV^{R}$

E(scf) = -6023.66365617a.u.

ν_{min} = 20.3 cm^{-1}

C	-2.577174	0.007468	0.527483	P	-1.001253	-0.828208	1.055352
C	-2.538487	0.981767	-0.499383	P	-0.946276	1.362046	-1.361512

Fe	0.970175	0.512335	-0.001273
C	-1.188204	-2.463178	0.216489
C	-1.138942	-1.290660	2.903046
C	-1.136170	0.760337	-3.159539
C	-1.020350	3.198635	-1.504435
C	1.832245	2.261765	-0.426995
C	2.679338	2.345048	-1.544874
C	1.585574	3.439597	0.291874
C	3.241041	3.564008	-1.938128
H	2.900887	1.450186	-2.121708
C	2.143376	4.663090	-0.093001
H	0.937082	3.404953	1.164552
C	2.973449	4.730787	-1.214257
H	3.888923	3.602494	-2.811839
H	1.932046	5.560607	0.484835
H	3.410753	5.678656	-1.518735
C	-2.030619	-2.512855	3.199136
H	-3.064388	-2.406253	2.862500
H	-2.058253	-2.664318	4.286357
H	-1.624982	-3.426975	2.753941
C	0.297523	-1.658507	3.334280
H	0.271519	-2.088128	4.344565
H	0.938602	-0.779521	3.361026
H	0.748302	-2.406774	2.670196
C	-1.614859	-0.063767	3.700174
H	-2.667341	0.169385	3.511678
H	-1.009666	0.816186	3.466293
H	-1.507202	-0.266700	4.774348
C	-1.452766	-0.744122	-3.148995
H	-2.399409	-0.954989	-2.637934
H	-0.650086	-1.308713	-2.669692
H	-1.544282	-1.101395	-4.183268
C	0.226864	1.006699	-3.837383
H	0.500306	2.067732	-3.838595
H	0.171960	0.675717	-4.883104
H	1.021378	0.440720	-3.344672
C	-2.236924	1.498289	-3.939285
H	-2.251083	1.114625	-4.968156
H	-2.062239	2.577305	-3.998737
H	-3.230897	1.325070	-3.515626
H	-1.925954	3.543367	-2.008575
H	-0.145201	3.554395	-2.052761
H	-0.989436	3.630463	-0.501617
H	-1.161346	-2.313666	-0.862084
H	-0.353845	-3.115436	0.486974
H	-2.136341	-2.935725	0.490026
Br	1.704807	-1.431919	-1.346561
C	-4.986080	0.359958	0.739146
C	-4.939242	1.346863	-0.244860
C	6.254317	-0.635806	0.781550
H	6.207564	-0.738636	-0.310606
H	6.677298	0.352016	1.006486
H	6.954177	-1.393508	1.158957
C	4.974003	-0.623223	2.934718
H	4.009298	-0.787806	3.429828
H	5.307321	0.386837	3.193643
H	5.688407	-1.344214	3.354771
C	4.857725	-0.799105	1.410278
C	4.345118	-2.218256	1.102017
H	4.275526	-2.386968	0.022481
H	5.018309	-2.973578	1.530495
H	3.344563	-2.390810	1.514871
C	3.926094	0.276618	0.776717
C	2.511347	0.367419	1.364780
C	1.625704	2.251885	4.526091
C	2.956753	2.983511	4.098471
B	2.383938	1.378424	2.537160
O	3.420465	2.170735	2.988855
O	1.242151	1.558335	3.306887
C	1.841599	1.169830	5.590948
H	2.101330	1.604830	6.561497
H	0.917465	0.594729	5.706562
H	2.635589	0.476484	5.294981
C	0.486332	3.175187	4.944486
H	-0.392222	2.580126	5.215733
H	0.773852	3.772658	5.816776
H	0.199051	3.853196	4.137029
C	4.05021	2.996527	5.164667
H	4.93729	3.504529	4.771093
H	3.716911	3.537392	6.057531
H	4.343742	1.985504	5.456511
C	2.736432	4.405705	3.575042
H	2.486487	5.093396	4.390091
H	3.657323	4.754185	3.096572

H	1.939425	4.442627	2.831259	C	-3.725043	1.647246	-0.857168
H	4.41744	1.252732	0.871322	H	-5.926562	0.105208	1.220254
H	3.882553	0.060527	-0.296286	H	-5.840557	1.878485	-0.537698
H	2.172381	-0.624817	1.683716	H	-3.706437	2.417749	-1.619487
C	-3.816577	-0.296563	1.116537	H	-3.879544	-1.051186	1.888586

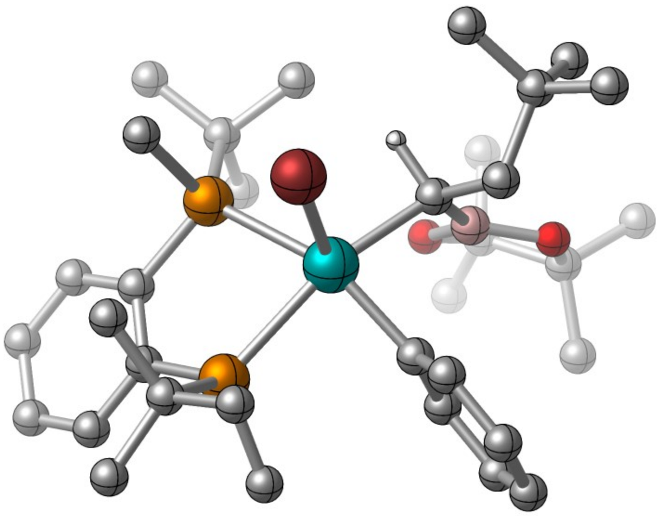

Zero-point correction= 0.853591 (Hartree/Particle)
Thermal correction to Energy= 0.902755
Thermal correction to Enthalpy= 0.903699
Thermal correction to Gibbs Free Energy= 0.773462
Sum of electronic and zero-point Energies= -6022.810065
Sum of electronic and thermal Energies= -6022.760901
Sum of electronic and thermal Enthalpies= -6022.759957
Sum of electronic and thermal Free Energies= -6022.890195

$^{4}\boldsymbol{IV}^{S}$

E(scf) = -6023.66697529 a.u.

ν_{min} = 24.1 cm^{-1}

C	-2.724261	-0.608196	0.495955	C	-2.671257	1.014751	-3.298923
C	-3.069417	0.517418	-0.294376	C	-1.910083	2.957260	-1.237388
P	-1.201248	-1.561644	0.043870	C	1.085001	0.627558	0.545733
P	-1.880451	1.144587	-1.576009	C	2.355179	0.245425	1.009954
Fe	0.323428	-0.018989	-1.200056	C	0.312562	1.442560	1.389943
C	-1.982728	-2.879195	-0.989445	C	2.834311	0.656682	2.256462
C	-0.529795	-2.574161	1.513553	H	2.986309	-0.388248	0.392774

C	0.781421	1.860626	2.640453	H	4.495857	2.801123	-2.942218
H	-0.690522	1.736918	1.094782	H	3.132197	2.719186	-4.070730
C	2.048842	1.471639	3.077949	H	4.770451	2.390558	-4.649408
H	3.821289	0.340063	2.588072	C	3.904655	0.763561	-3.474847
H	0.153651	2.486249	3.271893	C	5.286403	0.131480	-3.223233
H	2.419035	1.793800	4.048308	H	5.193429	-0.934280	-2.976154
C	-1.380352	-3.810832	1.868404	H	5.928886	0.213167	-4.110005
H	-2.399721	-3.563297	2.177661	H	5.802857	0.624860	-2.389373
H	-0.901999	-4.330734	2.708895	C	3.058484	0.574487	-2.180341
H	-1.441843	-4.522994	1.040483	C	1.666817	1.228822	-2.171960
C	0.848800	-3.067586	1.020851	C	1.389862	5.004068	-1.940228
H	1.318206	-3.673237	1.807145	C	2.258627	4.670584	-0.666022
H	1.516380	-2.233782	0.790374	B	1.696494	2.724799	-1.758610
H	0.763604	-3.691707	0.124114	O	2.590928	3.271426	-0.861681
C	-0.344927	-1.699561	2.764836	O	0.889706	3.695960	-2.333171
H	-1.291329	-1.281314	3.123064	C	0.203662	5.931275	-1.685112
H	0.345657	-0.875906	2.586659	H	0.551032	6.913821	-1.346405
H	0.071934	-2.317157	3.571419	H	-0.357971	6.073405	-2.615102
C	-3.084553	-0.441722	-3.559236	H	-0.480467	5.527871	-0.935920
H	-3.850578	-0.777916	-2.850758	C	2.210410	5.537493	-3.119714
H	-2.222902	-1.112841	-3.501986	H	1.577372	5.554466	-4.013294
H	-3.504389	-0.525375	-4.570367	H	2.567516	6.555356	-2.930849
C	-1.559404	1.415819	-4.292959	H	3.072391	4.899402	-3.329185
H	-1.157545	2.415443	-4.093179	C	1.473615	4.769267	0.645002
H	-1.970811	1.418369	-5.311039	H	2.074431	4.340112	1.45211
H	-0.729937	0.702329	-4.268312	H	1.241229	5.809838	0.895615
C	-3.890654	1.932440	-3.481623	H	0.541324	4.201579	0.591493
H	-4.274847	1.812684	-4.503387	C	3.559171	5.461023	-0.549893
H	-3.642240	2.990372	-3.349421	H	3.356335	6.534696	-0.46655
H	-4.704592	1.675543	-2.795744	H	4.098783	5.145818	0.349775
H	-2.924901	3.361189	-1.204106	H	4.212582	5.294235	-1.409685
H	-1.331765	3.466579	-2.006667	H	2.988373	-0.508762	-2.013861
H	-1.425781	3.148411	-0.277240	H	1.186561	1.149079	-3.1537
H	-2.364510	-2.435359	-1.906887	H	3.638652	0.97682	-1.342198
H	-1.240472	-3.632650	-1.260614	C	-4.268315	1.197214	-0.018964
H	-2.811311	-3.346746	-0.449117	H	-4.544026	2.068555	-0.602635
Br	0.519126	-1.729742	-2.944362	C	-5.114598	0.789083	1.010286
C	3.250285	0.060656	-4.677269	H	-6.034782	1.333937	1.203306
H	3.093403	-1.004318	-4.476830	C	-4.767199	-0.311246	1.793915
H	2.271335	0.491303	-4.914769	H	-5.412812	-0.634129	2.606219
H	3.883070	0.157558	-5.570111	C	-3.584624	-1.00168	1.533013
C	4.087059	2.255180	-3.800557	H	-3.336114	-1.857281	2.14816

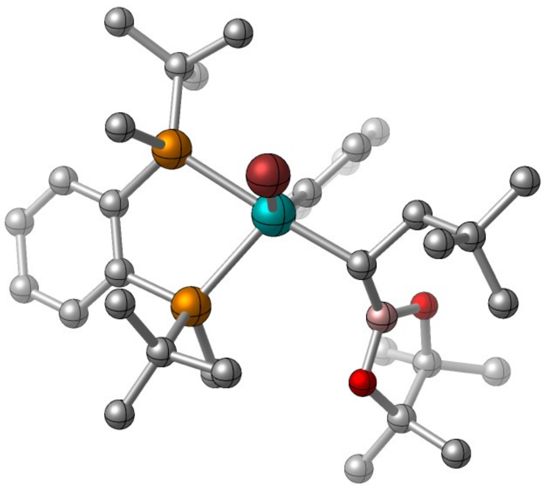

Zero-point correction= 0.853884 (Hartree/Particle)
Thermal correction to Energy= 0.903197
Thermal correction to Enthalpy= 0.904141
Thermal correction to Gibbs Free Energy= 0.773811
Sum of electronic and zero-point Energies= -6022.813091
Sum of electronic and thermal Energies= -6022.763778
Sum of electronic and thermal Enthalpies= -6022.762834
Sum of electronic and thermal Free Energies= -6022.893164

4^R

E(scf) = -878.888177811 a.u.

ν_{min} = 28.5 cm^{-1}

C	1.884805	2.255886	0.888773	H	2.510191	7.228447	0.158928
C	2.948864	2.038115	1.781810	H	4.052598	6.756582	-0.578451
C	0.631731	1.713694	1.209869	H	2.853571	7.654822	-1.527513
C	2.761437	1.310996	2.958328	C	3.061451	5.079237	-2.516857
H	3.931981	2.432860	1.542264	H	2.583094	4.189510	-2.943313
C	0.441578	0.982471	2.385097	H	4.112752	4.838271	-2.322505
H	-0.204021	1.869157	0.531041	H	3.027903	5.862923	-3.284451
C	1.505890	0.779504	3.266444	C	2.349807	5.544798	-1.233852
H	3.599216	1.155851	3.634022	C	0.858460	5.782164	-1.536401
H	-0.540093	0.573366	2.611940	H	0.323982	6.126094	-0.641550
H	1.359752	0.212090	4.181998	H	0.737711	6.547530	-2.313666
C	2.977762	6.872276	-0.768078	H	0.362958	4.872178	-1.894260

C	2.517153	4.515382	-0.085338	H	4.836543	-1.031203	-3.516492
C	2.087189	3.056267	-0.393440	H	3.626927	-1.193855	-2.223250
C	4.030468	0.859786	-2.830708	C	6.458048	1.682841	-2.318535
C	5.116634	1.232596	-1.753245	H	7.137763	1.935728	-1.497757
B	3.139531	2.255737	-1.256927	H	6.917224	0.879513	-2.905287
O	4.496510	2.363005	-1.066249	H	6.353338	2.564942	-2.954785
O	2.794812	1.322115	-2.200105	C	5.321036	0.142239	-0.696533
C	4.177517	1.643762	-4.138007	H	5.827191	-0.732956	-1.116461
H	5.048254	1.306599	-4.709673	H	5.939345	0.542769	0.113328
H	3.282350	1.487406	-4.748773	H	4.366812	-0.177279	-0.265544
H	4.276938	2.717021	-3.950226	H	3.568688	4.511308	0.229246
C	3.896808	-0.630915	-3.119986	H	1.939322	4.878308	0.776187
H	3.115016	-0.792664	-3.869862	H	1.130629	3.063220	-0.927660

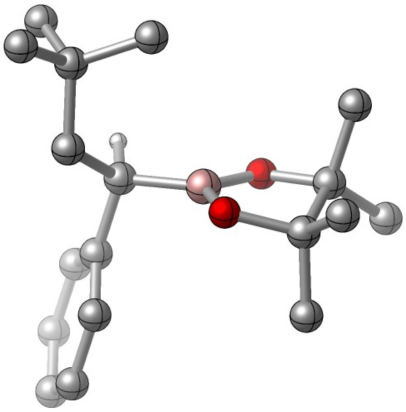

Zero-point correction= 0.444409 (Hartree/Particle)
Thermal correction to Energy= 0.466413
Thermal correction to Enthalpy= 0.467357
Thermal correction to Gibbs Free Energy= 0.394136
Sum of electronic and zero-point Energies= -878.443769
Sum of electronic and thermal Energies= -878.421765
Sum of electronic and thermal Enthalpies= -878.420821
Sum of electronic and thermal Free Energies= -878.494042

4^S^

4^{S}
E(scf) = -878.888155340 a.u.
ν_{min} = 24.6 cm^{-1}

C	2.148834	1.727178	-0.152179	C	2.675803	2.903283	-0.967650
C	2.889887	1.201022	0.916159	C	3.822489	1.545555	-4.341305
C	0.908074	1.137052	-0.451200	C	2.253992	1.449239	-4.440351
C	2.409557	0.124965	1.666763	B	2.856092	2.375693	-2.445117
H	3.853230	1.642211	1.162723	O	1.820813	2.316468	-3.347373
C	0.424030	0.063892	0.298500	O	4.027008	1.847891	-2.925408
H	0.327267	1.517014	-1.286693	C	4.568131	0.258292	-4.673557
C	1.172711	-0.447872	1.362166	H	4.369817	-0.044643	-5.707704
H	3.002672	-0.263997	2.491027	H	5.646376	0.418627	-4.567048
H	-0.538310	-0.376367	0.048318	H	4.280251	-0.558812	-4.007742
H	0.796848	-1.284680	1.945282	C	4.410246	2.718602	-5.131279
C	3.433913	5.932343	-0.277153	H	5.461116	2.844689	-4.850940
H	3.043770	5.984773	0.747433	H	4.360475	2.537796	-6.209927
H	4.281011	5.236581	-0.278430	H	3.885491	3.652499	-4.908739
H	3.824715	6.924166	-0.538014	C	1.711136	0.051455	-4.125622
C	2.920807	5.459395	-2.689864	H	0.622169	0.106674	-4.027055
H	2.182506	5.093166	-3.412231	H	1.949542	-0.659757	-4.922985
H	3.800251	4.806838	-2.744126	H	2.115747	-0.329440	-3.182439
H	3.241338	6.459786	-3.007672	C	1.656812	1.970071	-5.741937
C	2.334972	5.503210	-1.267149	H	2.022090	1.385187	-6.593386
C	1.198790	6.542827	-1.226837	H	0.565810	1.878942	-5.709595
H	0.739893	6.590554	-0.230868	H	1.903539	3.021413	-5.908212
H	1.573189	7.545191	-1.471046	H	1.387695	4.217439	0.182526
H	0.409562	6.294700	-1.948058	H	3.661993	3.162236	-0.566498
C	1.735186	4.132711	-0.856645	H	0.841395	3.946256	-1.465837

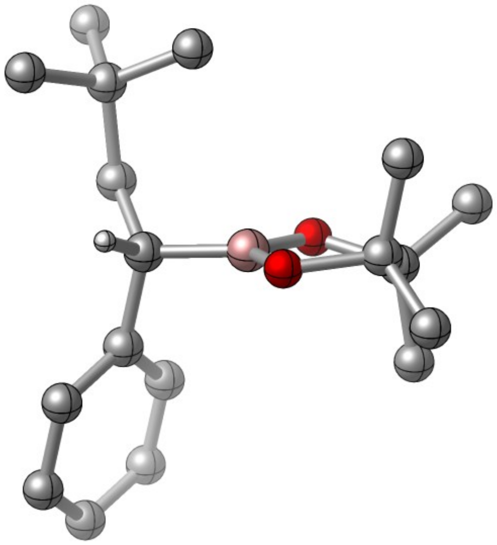

Zero-point correction= 0.444204 (Hartree/Particle)

Thermal correction to Energy= 0.466278
Thermal correction to Enthalpy= 0.467222
Thermal correction to Gibbs Free Energy= 0.393655
Sum of electronic and zero-point Energies= -878.443951
Sum of electronic and thermal Energies= -878.421877
Sum of electronic and thermal Enthalpies= -878.420933
Sum of electronic and thermal Free Energies= -878.494500

$^{4}TS^{R}_{III\text{-}IV}$

E(scf) = -6023.64979557 a.u.

ν_{min} = -77.0 cm^{-1}

C	-2.725887	-0.791529	0.362337	C	1.184054	-2.349328	1.416684
C	-3.119913	0.390660	-0.315095	H	1.666999	-2.817729	2.284378
P	-1.018296	-1.457842	0.084377	H	1.664597	-1.384378	1.237458
P	-1.970322	1.186651	-1.528216	H	1.372177	-2.988608	0.545359
Fe	0.283457	0.321902	-1.098159	C	-0.535470	-1.192909	2.849466
C	-1.342131	-2.956777	-0.941826	H	-1.594997	-1.074738	3.101271
C	-0.326696	-2.189013	1.697614	H	-0.125709	-0.207081	2.610626
C	-2.714997	0.884260	-3.247104	H	-0.017534	-1.559915	3.745442
C	-2.197414	2.989051	-1.222746	C	-2.746549	-0.634439	-3.486399
C	1.335070	1.153393	0.425028	H	-3.380307	-1.146238	-2.753291
C	2.722626	0.989511	0.598234	H	-1.739801	-1.061236	-3.447155
C	0.665386	1.918202	1.400072	H	-3.158352	-0.836964	-4.483710
C	3.405489	1.555631	1.679657	C	-1.765151	1.542855	-4.267424
H	3.286373	0.425396	-0.140753	H	-1.729949	2.632131	-4.151946
C	1.333989	2.496631	2.485720	H	-2.129496	1.333712	-5.281802
H	-0.411538	2.071405	1.314866	H	-0.745534	1.150288	-4.192417
C	2.712347	2.315875	2.628013	C	-4.129758	1.460801	-3.407034
H	4.480329	1.413080	1.779890	H	-4.471762	1.286336	-4.435899
H	0.783206	3.084564	3.218188	H	-4.163351	2.541441	-3.230521
H	3.241411	2.763298	3.466583	H	-4.846474	0.975644	-2.737142
C	-0.894325	-3.567701	2.082469	H	-3.239351	3.315150	-1.280043
H	-1.969726	-3.551867	2.280615	H	-1.614069	3.544840	-1.959438
H	-0.400977	-3.905361	3.003668	H	-1.808489	3.226574	-0.228375
H	-0.700613	-4.322928	1.314307	H	-1.663582	-2.650791	-1.937434

H	-0.413592	-3.523613	-1.049355	C	4.752574	-0.192071	-2.714174
H	-2.113375	-3.591404	-0.494147	H	5.429362	-0.807395	-3.316942
Br	1.069527	-1.135365	-2.851677	H	5.158280	-0.133733	-1.698306
C	-4.903207	-0.865437	1.462528	H	3.775585	-0.677672	-2.664682
C	-5.277092	0.316332	0.821871	C	5.995701	1.910667	-3.250125
C	1.876564	6.165296	-0.697358	H	6.412488	1.845457	-2.239172
H	1.060422	6.124141	0.035663	H	6.692792	1.418134	-3.937361
H	2.804340	5.892908	-0.178538	H	5.929322	2.967928	-3.518119
H	1.972320	7.205453	-1.035425	C	3.863346	-0.010179	-5.459424
C	2.793851	5.282176	-2.859445	H	3.321716	0.134968	-6.40082
H	2.594768	4.698794	-3.765951	H	4.88066	-0.339649	-5.69902
H	3.704980	4.882894	-2.399061	H	3.35057	-0.791953	-4.895235
H	2.990544	6.317279	-3.167580	C	4.359136	2.448613	-5.568875
C	1.607015	5.220708	-1.882001	H	5.376132	2.278476	-5.937898
C	0.324564	5.669810	-2.603129	H	3.688794	2.530528	-6.431043
H	-0.534223	5.669751	-1.919772	H	4.335759	3.403044	-5.033458
H	0.435489	6.688220	-2.997748	H	2.312897	3.559187	-0.681067
H	0.086450	5.012765	-3.448109	H	0.574626	3.798687	-0.625708
C	1.437792	3.776641	-1.302362	H	0.355825	2.712678	-2.901787
C	1.279441	2.693379	-2.330005	H	-5.588356	-1.361097	2.145
C	4.629907	1.227196	-3.279516	H	-6.254422	0.755381	1.003141
C	3.879490	1.299702	-4.675043	C	-3.640761	-1.408808	1.231346
B	2.505568	2.048308	-2.998729	H	-3.3685	-2.324895	1.741163
O	2.511571	1.613788	-4.304662	C	-4.392035	0.931874	-0.061002
O	3.741360	1.958404	-2.393463	H	-4.701961	1.845704	-0.55665

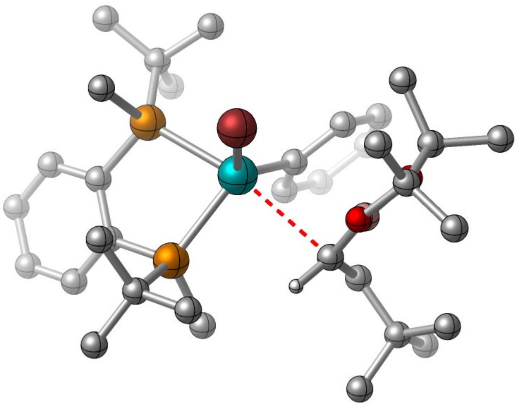

Zero-point correction= 0.851011 (Hartree/Particle)
Thermal correction to Energy= 0.900496
Thermal correction to Enthalpy= 0.901441
Thermal correction to Gibbs Free Energy= 0.769322
Sum of electronic and zero-point Energies= -6022.798785

Sum of electronic and thermal Energies= -6022.749299
Sum of electronic and thermal Enthalpies= -6022.748355
Sum of electronic and thermal Free Energies= -6022.880473

$^{4}TS^{R}_{IV\text{-}I}$
E(scf) = -6023.64589510 a.u.
ν_{min} = -323.3 cm^{-1}

C	-2.607082	0.021979	0.394016	H	-0.882755	0.924166	3.247808
C	-2.563612	0.997861	-0.634357	H	-1.383762	-0.080244	4.614559
P	-1.032133	-0.818306	0.917496	C	-1.433177	-0.790923	-3.248291
P	-0.970861	1.346503	-1.506841	H	-2.367042	-1.025470	-2.723758
Fe	0.866999	0.406780	-0.177073	H	-0.607104	-1.317869	-2.763832
C	-1.234679	-2.482811	0.142931	H	-1.521025	-1.173916	-4.273812
C	-1.107082	-1.200702	2.780731	C	0.181630	1.002615	-3.974581
C	-1.172674	0.724154	-3.289947	H	0.411329	2.073831	-4.010283
C	-0.992403	3.182098	-1.664715	H	0.147341	0.635529	-5.009117
C	1.927191	2.093508	-0.072469	H	0.998704	0.487329	-3.459805
C	2.766911	2.327445	-1.194280	C	-2.303996	1.410061	-4.070947
C	1.384826	3.242132	0.564055	H	-2.317321	1.015332	-5.095874
C	3.014215	3.617514	-1.660574	H	-2.169308	2.494296	-4.143248
H	3.205113	1.484185	-1.719097	H	-3.286194	1.208484	-3.631527
C	1.648584	4.528670	0.100066	H	-1.827194	3.551245	-2.266050
H	0.739730	3.112341	1.428339	H	-0.051966	3.510209	-2.114151
C	2.466564	4.734618	-1.016604	H	-1.055884	3.618994	-0.666157
H	3.647650	3.751880	-2.535588	H	-1.196952	-2.376751	-0.941551
H	1.203417	5.379696	0.612267	H	-0.404847	-3.128804	0.442488
H	2.677069	5.739238	-1.373304	H	-2.186500	-2.942886	0.426341
C	-2.012932	-2.379662	3.180849	Br	1.791485	-1.486362	-1.434803
H	-3.065759	-2.229953	2.929525	C	-5.006051	0.418105	0.634128
H	-1.957562	-2.510867	4.269896	C	-4.952655	1.407963	-0.347453
H	-1.685218	-3.318672	2.723100	C	6.227843	-0.835405	0.820036
C	0.346155	-1.578950	3.147448	H	6.166168	-1.030289	-0.258465
H	0.376074	-1.955933	4.178447	H	6.777049	0.104649	0.960643
H	1.008053	-0.714558	3.091812	H	6.821185	-1.640906	1.272134
H	0.741398	-2.368303	2.496059	C	4.969030	-0.475565	2.953462
C	-1.512675	0.076652	3.535012	H	3.997461	-0.503855	3.460690
H	-2.561075	0.339849	3.360004	H	5.410368	0.508677	3.136776

H	5.605256	-1.236119	3.425264	H	0.955489	3.767361	5.882303
C	4.822947	-0.760135	1.448785	H	0.382733	3.896771	4.205007
C	4.130381	-2.123049	1.265880	C	4.265283	3.076927	5.169088
H	4.041416	-2.385324	0.207876	H	5.138097	3.613298	4.781688
H	4.699501	-2.911718	1.776474	H	3.939272	3.576522	6.088184
H	3.115626	-2.123804	1.680105	H	4.577185	2.059252	5.415176
C	4.049877	0.369554	0.699364	C	2.895073	4.52814	3.660521
C	2.648686	0.694447	1.213463	H	2.606297	5.171194	4.498535
C	1.843212	2.312471	4.540544	H	3.812865	4.927892	3.21712
C	3.153714	3.091889	4.123127	H	2.114497	4.567415	2.898324
B	2.581138	1.572789	2.494958	H	4.657047	1.283172	0.724887
O	3.615932	2.345846	2.961597	H	3.992182	0.055207	-0.347168
O	1.463611	1.643025	3.304338	H	2.099346	-0.237778	1.39691
C	2.086803	1.207334	5.573733	C	-3.844663	-0.261823	0.996428
H	2.341142	1.622286	6.554362	C	-3.740695	1.68711	-0.97508
H	1.174387	0.611201	5.677041	H	-5.946132	0.179247	1.124357
H	2.892784	0.537850	5.258096	H	-5.848402	1.955798	-0.62765
C	0.683280	3.196209	4.987863	H	-3.713608	2.457317	-1.738254
H	-0.181835	2.571324	5.233759	H	-3.91171	-1.020711	1.764996

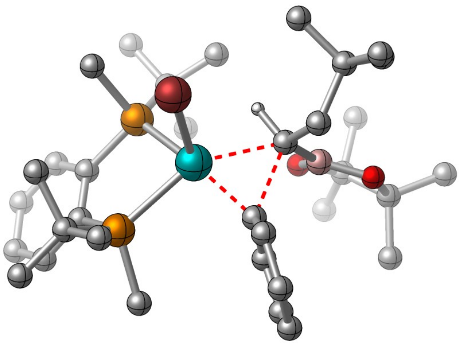

Zero-point correction= 0.851972 (Hartree/Particle)
Thermal correction to Energy= 0.900969
Thermal correction to Enthalpy= 0.901913
Thermal correction to Gibbs Free Energy= 0.771187
Sum of electronic and zero-point Energies= -6022.793923
Sum of electronic and thermal Energies= -6022.744926
Sum of electronic and thermal Enthalpies= -6022.743982
Sum of electronic and thermal Free Energies= -6022.874708

$^{4}TS^{S}_{III-IV}$

E(scf) = -6023.64839295 a.u.

ν_{min} = -25.6 cm^{-1}

C	-2.935770	-0.636864	0.336749	H	-0.440090	1.828880	-3.645458
C	-3.157500	0.679348	-0.142464	C	-3.883498	2.067311	-3.130107
P	-1.268842	-1.405824	0.105655	H	-4.152293	2.023900	-4.194126
P	-1.857504	1.526544	-1.156728	H	-3.919440	3.119524	-2.827526
Fe	0.273554	0.351675	-0.748050	H	-4.651391	1.519774	-2.574231
C	-1.612971	-2.809031	-1.039045	H	-3.035869	3.687167	-0.798735
C	-0.727796	-2.256204	1.714343	H	-1.349657	3.896697	-1.308578
C	-2.489922	1.451373	-2.942552	H	-1.712098	3.424870	0.357766
C	-2.019220	3.302050	-0.684658	H	-1.825507	-2.416465	-2.034755
C	1.317114	1.068353	0.865429	H	-0.722205	-3.438991	-1.110567
C	2.663216	0.731516	1.124613	H	-2.462641	-3.410737	-0.701010
C	0.715825	1.944956	1.792950	Br	1.168887	-0.993286	-2.538685
C	3.367113	1.237567	2.222108	C	3.165949	4.610738	-0.043476
H	3.181434	0.054579	0.444433	H	2.592743	4.288799	0.834224
C	1.402282	2.462684	2.898836	H	3.909810	3.834992	-0.255984
H	-0.327072	2.232587	1.656840	H	3.707375	5.528150	0.222196
C	2.737084	2.110608	3.115071	C	3.077104	5.277250	-2.471042
H	4.406236	0.954982	2.382304	H	2.445203	5.399163	-3.359666
H	0.899262	3.138222	3.588885	H	3.842240	4.527952	-2.707045
H	3.280017	2.510109	3.968764	H	3.588905	6.230240	-2.284301
C	-1.454847	-3.570187	2.046702	C	2.242939	4.854823	-1.249918
H	-2.512646	-3.422075	2.281220	C	1.238164	5.966808	-0.902915
H	-0.989365	-4.015508	2.936379	H	0.615516	5.682045	-0.044794
H	-1.378234	-4.303929	1.237767	H	1.756038	6.899966	-0.645797
C	0.768803	-2.565570	1.487359	H	0.570573	6.177485	-1.748694
H	1.182308	-3.042925	2.385328	C	1.425216	3.560366	-1.571607
H	1.341568	-1.655817	1.288384	C	2.246610	2.383559	-1.988153
H	0.920270	-3.250834	0.644699	C	3.711131	1.459367	-5.321884
C	-0.883868	-1.260295	2.875058	C	2.249518	1.891358	-5.723366
H	-1.937297	-1.028781	3.069859	B	2.528063	2.039367	-3.456348
H	-0.354937	-0.322584	2.677641	O	1.770277	2.527401	-4.505282
H	-0.460809	-1.695349	3.790119	O	3.614362	1.304142	-3.880050
C	-2.514498	-0.026580	-3.367038	C	4.177326	0.136898	-5.921246
H	-3.212022	-0.610156	-2.755504	H	4.187133	0.188426	-7.015946
H	-1.519417	-0.476924	-3.296240	H	5.195318	-0.083407	-5.581667
H	-2.843151	-0.100425	-4.412036	H	3.532462	-0.688966	-5.612371
C	-1.453573	2.210894	-3.789907	C	4.754046	2.554849	-5.573768
H	-1.448265	3.284169	-3.569640	H	5.696251	2.264501	-5.097357
H	-1.703424	2.098454	-4.853220	H	4.938459	2.701071	-6.643311

H	4.43616	3.508718	-5.139752	H	2.865142	1.928039	-1.220673
C	1.322502	0.704373	-6.006985	H	0.705355	3.804737	-2.362195
H	0.296216	1.070986	-6.112433	C	-4.395077	1.295767	0.111655
H	1.601359	0.193595	-6.934904	H	-4.580756	2.30718	-0.233734
H	1.339795	-0.012009	-5.181739	C	-5.409162	0.628184	0.795964
C	2.166766	2.905541	-6.859827	H	-6.35611	1.127923	0.981549
H	2.590599	2.490178	-7.781108	C	-5.207734	-0.684547	1.2245
H	1.117762	3.156662	-7.051722	H	-5.998785	-1.221865	1.740633
H	2.695673	3.83062	-6.61733	C	-3.982077	-1.305845	0.992928
H	0.854767	3.315938	-0.667779	H	-3.842682	-2.327155	1.327034

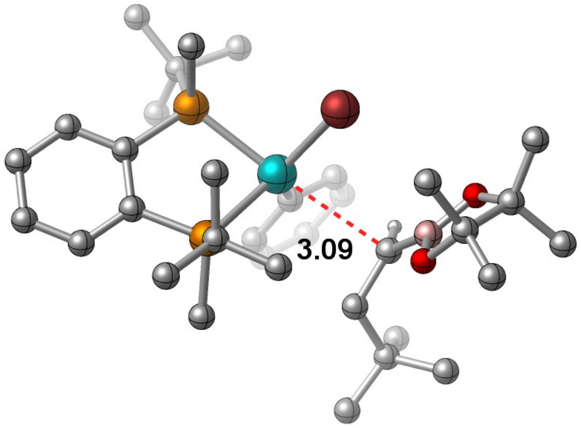

Zero-point correction= 0.850458 (Hartree/Particle)
Thermal correction to Energy= 0.900302
Thermal correction to Enthalpy= 0.901246
Thermal correction to Gibbs Free Energy= 0.767300
Sum of electronic and zero-point Energies= -6022.797935
Sum of electronic and thermal Energies= -6022.748091
Sum of electronic and thermal Enthalpies= -6022.747147
Sum of electronic and thermal Free Energies= -6022.881093

^{4}TS$^{S}_{IV\text{-}I}$
E(scf) = -6023.64719616 a.u.
ν_{min} = -292.8 cm^{-1}

C	-2.725293	-0.664224	0.266578	P	-1.719079	1.457223	-1.329067
C	-3.038264	0.546923	-0.399209	Fe	0.475773	0.602611	-0.666411
P	-0.963632	-1.240946	0.297757	C	-1.080923	-2.771155	-0.727131

C	-0.531079	-1.899409	2.025402
C	-2.122491	1.215195	-3.170223
C	-2.134430	3.221750	-0.993259
C	1.541088	1.484565	0.742200
C	2.657876	0.835578	1.318869
C	0.771996	2.327125	1.585186
C	2.959535	0.986680	2.671717
H	3.275447	0.188456	0.703072
C	1.076585	2.470513	2.938932
H	-0.064155	2.879741	1.165104
C	2.173854	1.803782	3.493974
H	3.813473	0.457469	3.089766
H	0.455243	3.109506	3.563005
H	2.416934	1.922794	4.546364
C	-1.323360	-3.116553	2.531098
H	-2.350508	-2.860466	2.804403
H	-0.838830	-3.498433	3.440153
H	-1.346848	-3.937462	1.806874
C	0.956408	-2.297711	1.903368
H	1.349695	-2.544306	2.898308
H	1.562735	-1.485231	1.494474
H	1.090430	-3.178835	1.265619
C	-0.681028	-0.737533	3.018753
H	-1.724390	-0.407922	3.096246
H	-0.070924	0.120567	2.729507
H	-0.355736	-1.057778	4.017537
C	-2.185147	-0.295464	-3.453336
H	-3.006516	-0.775523	-2.909266
H	-1.245382	-0.787823	-3.186603
H	-2.352816	-0.458545	-4.526331
C	-0.937059	1.834804	-3.938864
H	-0.789484	2.892327	-3.687850
H	-1.126535	1.772756	-5.018750
H	-0.007815	1.294746	-3.731755
C	-3.435999	1.877097	-3.611396
H	-3.588781	1.688463	-4.682507
H	-3.424597	2.963193	-3.471062
H	-4.301182	1.466049	-3.081257
H	-3.140469	3.500201	-1.318189
H	-1.396779	3.857545	-1.483558
H	-2.055838	3.394967	0.084191
H	-1.333919	-2.488495	-1.750359

H	-0.112557	-3.275738	-0.749689
H	-1.849102	-3.452254	-0.346537
Br	1.432919	-0.949990	-2.335559
C	5.452556	1.783889	-2.857830
H	6.061615	2.158882	-2.024872
H	5.448415	0.687605	-2.803518
H	5.951453	2.070837	-3.792715
C	3.226389	1.809387	-4.002846
H	2.209550	2.218252	-4.030664
H	3.135986	0.720076	-3.964730
H	3.721150	2.090835	-4.942290
C	4.019057	2.346057	-2.797459
C	4.083762	3.880481	-2.888488
H	4.586285	4.315516	-2.018492
H	4.625517	4.188759	-3.792554
H	3.080138	4.316690	-2.947286
C	3.400149	1.865042	-1.447841
C	1.970757	2.310211	-1.152255
C	0.994216	5.926084	-0.533668
C	2.311296	5.792414	0.340495
B	1.825953	3.782197	-0.674654
O	2.814763	4.477409	-0.019818
O	0.702939	4.549178	-0.899434
C	-0.217318	6.486146	0.208353
H	-0.028580	7.512307	0.542414
H	-1.082927	6.501284	-0.462728
H	-0.476325	5.878284	1.078317
C	1.194392	6.690662	-1.846145
H	0.287536	6.597447	-2.453106
H	1.381227	7.754558	-1.667510
H	2.028449	6.282373	-2.423639
C	2.052024	5.776911	1.84909
H	2.978812	5.50642	2.365547
H	1.72817	6.75949	2.208328
H	1.297459	5.035625	2.116207
C	3.397779	6.816655	0.018509
H	3.049942	7.833646	0.231102
H	4.276859	6.620631	0.641757
H	3.708799	6.764005	-1.027406
H	3.449106	0.771399	-1.455334
H	4.052518	2.20999	-0.636021
H	1.316421	2.143187	-2.015408

C	-4.364323	1.013459	-0.376576	C	-5.078326	-0.930934	0.857728
H	-4.618193	1.951157	-0.85878	H	-5.862908	-1.516714	1.32936
C	-5.376169	0.290687	0.252492	C	-3.765745	-1.399534	0.859195
H	-6.393309	0.673385	0.256643	H	-3.555365	-2.356281	1.320802

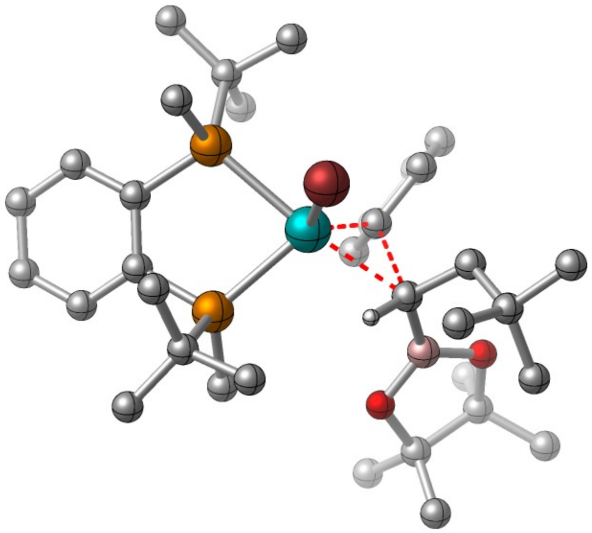

Zero-point correction= 0.852160 (Hartree/Particle)
Thermal correction to Energy= 0.901251
Thermal correction to Enthalpy= 0.902195
Thermal correction to Gibbs Free Energy= 0.770966
Sum of electronic and zero-point Energies= -6022.795036
Sum of electronic and thermal Energies= -6022.745945
Sum of electronic and thermal Enthalpies= -6022.745001
Sum of electronic and thermal Free Energies= -6022.876230

^{4}TS$^{R}_{IV\text{-}I}$C1
E(scf) = -6023.64119679 a.u.
ν_{min} = -266.8 cm^{-1}

C	-2.770283	-0.340224	0.435151	C	-0.645512	-2.197519	1.579806
C	-2.922357	0.994741	-0.010746	C	-2.462215	1.920015	-2.817111
P	-1.205507	-1.245868	0.029996	C	-1.480915	3.452798	-0.471041
P	-1.640121	1.733021	-1.121165	C	1.936686	0.779546	0.026446
Fe	0.377050	0.380117	-1.085816	C	3.135663	0.038228	-0.072850
C	-1.822753	-2.564335	-1.103363	C	1.547933	1.235017	1.309022

C	3.882249	-0.268541	1.063767
H	3.475097	-0.284385	-1.050962
C	2.293323	0.915489	2.445999
H	0.657944	1.853866	1.413668
C	3.465209	0.161034	2.331544
H	4.798081	-0.847768	0.962823
H	1.960187	1.260790	3.422774
H	4.053270	-0.077954	3.213860
C	-1.396280	-3.514766	1.840119
H	-2.464617	-3.371047	2.025319
H	-0.974311	-3.991165	2.735453
H	-1.284963	-4.221772	1.011608
C	0.837726	-2.524701	1.306359
H	1.233812	-3.145710	2.120829
H	1.443910	-1.619828	1.243853
H	0.965590	-3.080298	0.368960
C	-0.756561	-1.276761	2.806346
H	-1.799407	-1.096417	3.087362
H	-0.275871	-0.309267	2.630563
H	-0.252910	-1.745216	3.662358
C	-2.810559	0.505673	-3.315109
H	-3.539485	0.015125	-2.659226
H	-1.917443	-0.123628	-3.382263
H	-3.252819	0.569888	-4.318149
C	-1.391110	2.541315	-3.735416
H	-1.143165	3.567581	-3.439410
H	-1.776228	2.582772	-4.763212
H	-0.464651	1.959824	-3.749217
C	-3.723115	2.795634	-2.813626
H	-4.088648	2.903182	-3.843849
H	-3.529315	3.803069	-2.428665
H	-4.530429	2.347984	-2.225558
H	-2.442429	3.958684	-0.346838
H	-0.863829	4.046141	-1.148968
H	-0.977545	3.411755	0.500681
H	-2.146942	-2.096504	-2.035358
H	-1.006260	-3.251536	-1.339545
H	-2.660770	-3.116232	-0.665943
Br	0.715563	-1.065095	-3.016385
C	3.702258	4.758975	-2.485224
H	4.619589	4.159368	-2.463093
H	3.057284	4.361257	-3.273726
H	3.971939	5.785458	-2.766326
C	1.656359	5.498079	-1.241476
H	1.098214	5.472865	-0.296675
H	1.017229	5.073051	-2.023935
H	1.832181	6.550684	-1.498701
C	2.988205	4.735784	-1.124115
C	3.876755	5.443015	-0.082503
H	4.844425	4.935515	0.020903
H	4.073210	6.483130	-0.373206
H	3.399261	5.456086	0.905885
C	2.754213	3.281883	-0.612532
C	1.884495	2.365897	-1.481625
C	4.114497	1.020421	-4.263259
C	2.899209	1.654160	-5.082476
B	2.539544	1.879379	-2.810012
O	1.963152	2.059895	-4.046993
O	3.804525	1.347105	-2.881759
C	4.204769	-0.506105	-4.347010
H	4.439981	-0.838396	-5.363914
H	5.004989	-0.850208	-3.682152
H	3.271469	-0.968581	-4.021200
C	5.481937	1.628505	-4.579241
H	6.235410	1.177944	-3.924311
H	5.770395	1.429071	-5.617137
H	5.497396	2.708266	-4.412583
C	2.172197	0.676969	-6.007056
H	1.332158	1.193995	-6.484556
H	2.839181	0.30978	-6.79492
H	1.767846	-0.16556	-5.44564
C	3.261689	2.916433	-5.87256
H	3.932215	2.68299	-6.706494
H	2.344131	3.3516	-6.282832
H	3.738734	3.672814	-5.245403
H	3.733455	2.811535	-0.45916
H	0.92172	2.841441	-1.658993
H	2.304005	3.378473	0.383209
C	-4.070053	1.713715	0.366814
H	-4.192527	2.74521	0.054196
C	-5.069639	1.125721	1.139105
H	-5.947284	1.702036	1.41946
C	-4.942581	-0.206218	1.537423
H	-5.724053	-0.681049	2.124699

C	-3.802835	-0.926741	1.186163	H	-3.718316	-1.958894	1.505758

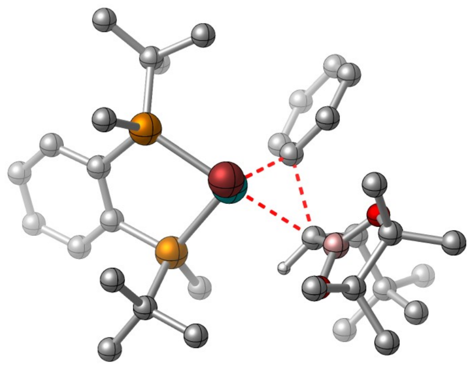

Zero-point correction= 0.851372 (Hartree/Particle)
Thermal correction to Energy= 0.900730
Thermal correction to Enthalpy= 0.901674
Thermal correction to Gibbs Free Energy= 0.770005
Sum of electronic and zero-point Energies= -6022.789825
Sum of electronic and thermal Energies= -6022.740467
Sum of electronic and thermal Enthalpies= -6022.739523
Sum of electronic and thermal Free Energies= -6022.871192

^{4}TS$^{R}_{IV\text{-}I}$C2

E(scf) = -6023.63715906 a.u.

ν_{min} = -225.3 cm^{-1}

C	-2.509847	-1.382997	0.226037	Fe	0.127165	0.873569	-0.765419
C	-3.178575	-0.463489	-0.618247	C	-0.007723	-2.803296	-0.031565
P	-0.691236	-1.181946	0.518759	C	-0.427970	-1.186043	2.399087
P	-2.179310	0.631668	-1.720947	C	-2.283784	-0.247452	-3.409601

C	-3.237726	2.133200	-1.914531	Br	1.714244	-0.432324	-2.114072
C	0.334032	2.405917	0.485642	C	-0.459819	4.685773	-4.543324
C	1.309251	2.291854	1.507784	H	-1.147220	3.866214	-4.789872
C	-0.815573	3.178440	0.776610	H	-1.024767	5.440920	-3.981176
C	1.103470	2.843100	2.771018	H	-0.138059	5.143687	-5.487632
H	2.238467	1.770018	1.297687	C	1.675221	5.367990	-3.434007
C	-1.013327	3.741644	2.038064	H	2.602485	5.041719	-2.950370
H	-1.557329	3.340182	0.001022	H	1.192358	6.095475	-2.774466
C	-0.062386	3.568428	3.049844	H	1.955658	5.877280	-4.365410
H	1.856121	2.706112	3.544711	C	0.748728	4.178531	-3.733408
H	-1.916045	4.317190	2.233629	C	1.519994	3.145396	-4.573813
H	-0.218736	4.001968	4.034020	H	0.889956	2.287933	-4.834121
C	-0.872372	-2.441182	3.168605	H	1.874954	3.597449	-5.509281
H	-1.960309	-2.522927	3.236542	H	2.394250	2.762043	-4.036338
H	-0.491863	-2.374758	4.197164	C	0.187014	3.520097	-2.430597
H	-0.475977	-3.365953	2.735364	C	1.210356	2.945584	-1.458980
C	1.098394	-1.029671	2.569736	C	2.723203	5.929567	0.363587
H	1.329521	-0.791393	3.616070	C	3.942160	4.893830	0.323530
H	1.503323	-0.229466	1.946488	B	2.070300	3.968810	-0.668806
H	1.624371	-1.957339	2.316461	O	3.396765	3.744632	-0.375174
C	-1.172903	0.038932	2.955802	O	1.630781	5.202970	-0.253342
H	-2.258460	-0.087005	2.864329	C	2.269518	6.324441	1.770841
H	-0.893494	0.958368	2.439154	H	3.053157	6.877263	2.300251
H	-0.938680	0.166825	4.020725	H	1.388353	6.970550	1.693236
C	-1.690265	-1.658486	-3.253515	H	1.987617	5.451339	2.361648
H	-2.275688	-2.272575	-2.560588	C	2.945910	7.199711	-0.464044
H	-0.654521	-1.616193	-2.905791	H	2.023691	7.790605	-0.462722
H	-1.693612	-2.160769	-4.230038	H	3.745858	7.815152	-0.038777
C	-1.406691	0.574069	-4.371114	H	3.194442	6.971764	-1.502626
H	-1.797677	1.587831	-4.514559	C	4.409153	4.409343	1.69862
H	-1.382717	0.086926	-5.355011	H	5.170125	3.633199	1.563288
H	-0.378519	0.641859	-4.003711	H	4.852891	5.22536	2.27898
C	-3.710457	-0.354119	-3.967360	H	3.586825	3.976671	2.271002
H	-3.673335	-0.862414	-4.940243	C	5.154181	5.369507	-0.481461
H	-4.171954	0.625344	-4.131707	H	5.616944	6.249857	-0.02271
H	-4.363963	-0.942946	-3.315972	H	5.900488	4.568511	-0.512852
H	-4.205884	1.925825	-2.377725	H	4.883273	5.612779	-1.512211
H	-2.710861	2.871495	-2.524077	H	-0.42596	4.267582	-1.909819
H	-3.413636	2.565991	-0.925454	H	-0.49471	2.73675	-2.77084
H	-0.150130	-2.898107	-1.109655	C	-4.580586	-0.508214	-0.703629
H	1.066492	-2.828162	0.168095	H	-5.110635	0.208226	-1.322374
H	-0.495270	-3.646660	0.468359	C	-5.314001	-1.476847	-0.020389

H	-6.397769	-1.491582	-0.099238	C	-3.259519	-2.393149	0.852191
C	-4.647515	-2.444028	0.733746	H	-2.756395	-3.154658	1.435796
H	-5.205171	-3.231149	1.234249	H	1.85487	2.19519	-1.914081

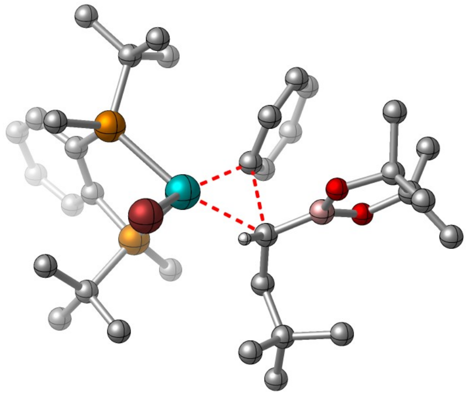

Zero-point correction= 0.851612 (Hartree/Particle)
Thermal correction to Energy= 0.900912
Thermal correction to Enthalpy= 0.901857
Thermal correction to Gibbs Free Energy= 0.770223
Sum of electronic and zero-point Energies= -6022.785547
Sum of electronic and thermal Energies= -6022.736247
Sum of electronic and thermal Enthalpies= -6022.735303
Sum of electronic and thermal Free Energies= -6022.866936

^{4}TS^{R}$_{IV\text{-}I}$C3

E(scf) = -6023.64511421 a.u.

ν_{min} = -286.9 cm^{-1}

C	-2.323375	-1.066472	-0.154072	P	-0.767938	-0.430868	0.626531
C	-3.058440	-0.236979	-1.034526	P	-2.217408	1.193221	-1.855699

Fe	0.131834	1.165998	-1.141519	H	-3.006478	2.820048	-0.237774
C	0.232578	-1.949198	0.905210	H	-0.216863	-2.645694	1.619082
C	-1.356954	0.023031	2.386684	H	0.380568	-2.444318	-0.056650
C	-2.590035	1.025767	-3.722781	H	1.204825	-1.623531	1.272679
C	-3.211378	2.644830	-1.298595	Br	0.953208	-0.787125	-2.420690
C	0.754143	2.905795	-1.888544	C	0.686338	4.404932	3.103650
C	-0.052718	4.073462	-1.880522	H	-0.341585	4.682369	2.835986
C	1.620658	2.734129	-3.003140	H	0.638359	3.499731	3.722481
C	-0.045884	4.969402	-2.946514	H	1.099311	5.212215	3.721965
H	-0.700486	4.272506	-1.032074	C	2.965091	3.769424	2.276741
C	1.612611	3.629210	-4.068882	H	3.623592	3.621559	1.413040
H	2.306362	1.893570	-3.009810	H	2.947613	2.835793	2.850785
C	0.777540	4.754195	-4.059429	H	3.417866	4.545094	2.907559
H	-0.694171	5.843277	-2.910913	C	1.545502	4.174654	1.846352
H	2.276210	3.453882	-4.913765	C	1.598761	5.479531	1.032878
H	0.781465	5.455203	-4.889747	H	0.592103	5.805194	0.743052
C	-2.286475	-1.013631	3.041133	H	2.055900	6.284472	1.622357
H	-3.253231	-1.080332	2.534220	H	2.187851	5.368764	0.115089
H	-2.476249	-0.712097	4.080496	C	0.878924	3.036955	1.024835
H	-1.843065	-2.014819	3.068749	C	1.640638	2.564119	-0.220041
C	-0.094757	0.150634	3.267415	C	3.998081	-0.214699	0.902901
H	-0.345843	0.677841	4.197050	C	4.698141	0.333775	-0.407084
H	0.722474	0.687932	2.780062	B	2.701794	1.421926	-0.050769
H	0.289369	-0.838294	3.542080	O	3.891017	1.496499	-0.739059
C	-2.118103	1.356300	2.283089	O	2.658954	0.367720	0.835445
H	-2.995599	1.254294	1.633242	C	3.879985	-1.734207	0.956838
H	-1.495717	2.161711	1.888949	H	4.874573	-2.192531	0.922565
H	-2.473416	1.663002	3.275878	H	3.402558	-2.040131	1.893847
C	-2.428797	-0.446864	-4.141412	H	3.286237	-2.120278	0.126224
H	-3.221938	-1.083243	-3.736911	C	4.623267	0.304269	2.201397
H	-1.463146	-0.844546	-3.814022	H	3.976966	0.025895	3.040647
H	-2.470687	-0.513335	-5.237015	H	5.613833	-0.130569	2.370187
C	-1.505755	1.864170	-4.428773	H	4.71615	1.393306	2.194258
H	-1.537466	2.915821	-4.127153	C	4.622514	-0.619792	-1.601633
H	-1.664444	1.822924	-5.514793	H	4.995321	-0.097717	-2.489855
H	-0.503343	1.485323	-4.215986	H	5.240587	-1.510049	-1.443543
C	-3.969161	1.555974	-4.150915	H	3.592384	-0.921596	-1.80233
H	-4.058601	1.464917	-5.241993	C	6.138603	0.80074	-0.207459
H	-4.099917	2.614341	-3.900850	H	6.776506	-0.032882	0.106656
H	-4.800195	0.995184	-3.714274	H	6.529386	1.188897	-1.154109
H	-4.286002	2.484298	-1.429373	H	6.210269	1.596999	0.53758
H	-2.910639	3.534631	-1.859042	H	0.72089	2.184286	1.684539

H	-0.12377	3.389612	0.748721	H	-4.541184	-3.656204	0.117176
H	2.261376	3.383979	-0.575496	C	-4.912501	-1.822138	-0.961797
C	-2.868011	-2.30593	0.221874	H	-5.923978	-2.0924	-1.253274
H	-2.301935	-2.971509	0.864578	C	-4.366606	-0.611979	-1.384859
C	-4.142699	-2.692218	-0.187936	H	-4.964668	0.039778	-2.011091

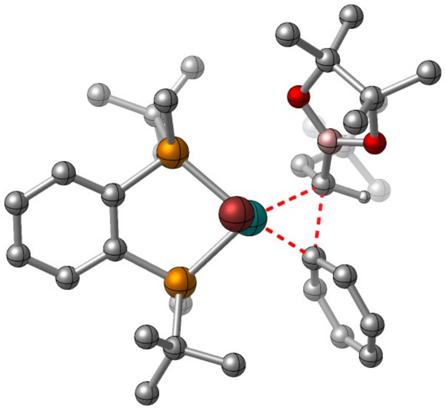

Zero-point correction= 0.852097 (Hartree/Particle)
Thermal correction to Energy= 0.901200
Thermal correction to Enthalpy= 0.902144
Thermal correction to Gibbs Free Energy= 0.771845
Sum of electronic and zero-point Energies= -6022.793017
Sum of electronic and thermal Energies= -6022.743914
Sum of electronic and thermal Enthalpies= -6022.742970
Sum of electronic and thermal Free Energies= -6022.873270

$^{4}TS^{S}_{IV\text{-}I}$C1

E(scf) = -6023.63785866 a.u.

ν_{min} = -303.1 cm^{-1}

C	-2.595987	-1.051555	0.082144	C	-3.075089	2.074211	-2.672458
C	-3.168840	0.181482	-0.313626	C	-2.133161	2.872229	0.015794
P	-0.755511	-1.212743	0.202984	C	1.249749	1.893153	-1.965185
P	-2.106273	1.443694	-1.153935	C	1.104550	3.025167	-1.117408
Fe	0.102285	0.374113	-1.488656	C	1.461367	2.149944	-3.348626
C	-0.459436	-2.989975	-0.196600	C	1.089636	4.320288	-1.632197
C	-0.441121	-1.081413	2.079169	H	0.978478	2.873767	-0.049043

C	1.455236	3.445502	-3.850861
H	1.619791	1.307539	-4.015874
C	1.263855	4.546706	-3.002310
H	0.951282	5.160240	-0.954040
H	1.605742	3.603671	-4.917027
H	1.270940	5.558127	-3.399290
C	-1.400746	-1.915883	2.941566
H	-2.435645	-1.571347	2.861672
H	-1.102096	-1.826130	3.994964
H	-1.371729	-2.980872	2.685005
C	1.001561	-1.551553	2.342599
H	1.228450	-1.427808	3.410524
H	1.727369	-0.958899	1.784316
H	1.136866	-2.612106	2.100533
C	-0.584495	0.405503	2.444217
H	-1.601509	0.764530	2.249159
H	0.125658	1.021114	1.883838
H	-0.381958	0.547979	3.514399
C	-3.720262	0.884558	-3.404479
H	-4.544985	0.445275	-2.834467
H	-2.982409	0.102713	-3.608105
H	-4.124025	1.231262	-4.365543
C	-1.992451	2.695123	-3.578546
H	-1.448615	3.502543	-3.075401
H	-2.463042	3.119058	-4.476141
H	-1.262737	1.945817	-3.895536
C	-4.132616	3.149104	-2.367460
H	-4.591689	3.470762	-3.312278
H	-3.695769	4.037634	-1.899557
H	-4.940921	2.787801	-1.725265
H	-3.149563	3.123186	0.334712
H	-1.679762	3.743577	-0.465496
H	-1.537251	2.623848	0.897520
H	-0.926296	-3.198425	-1.162924
H	0.615812	-3.154466	-0.302596
H	-0.851282	-3.680263	0.556452
Br	-0.493136	-1.076194	-3.432566
C	3.987678	-1.451592	-3.209831
H	3.112959	-1.721643	-3.814359
H	4.210283	-0.395252	-3.399786
H	4.844660	-2.034357	-3.570820
C	4.949502	-1.292440	-0.885444
H	4.782247	-1.488746	0.181694
H	5.161329	-0.224635	-1.000969
H	5.847838	-1.845503	-1.189080
C	3.733482	-1.729731	-1.717262
C	3.510918	-3.242809	-1.526293
H	2.642131	-3.589394	-2.100389
H	4.385168	-3.816186	-1.860360
H	3.335653	-3.487021	-0.470253
C	2.427930	-1.023245	-1.243117
C	2.454169	0.531918	-1.305531
C	4.370948	2.638036	1.209490
C	3.495855	1.726951	2.156117
B	3.055276	1.230918	-0.042591
O	2.531641	1.144298	1.229150
O	4.187308	2.009384	-0.092278
C	3.853400	4.074571	1.089916
H	4.023617	4.636497	2.014228
H	4.385714	4.576610	0.275623
H	2.787069	4.098676	0.854187
C	5.862626	2.648000	1.529166
H	6.386120	3.289465	0.812222
H	6.040417	3.045595	2.534488
H	6.296791	1.647339	1.465669
C	2.72802	2.472761	3.241765
H	2.139074	1.76182	3.830615
H	3.419583	2.985663	3.919203
H	2.042395	3.210628	2.818261
C	4.275658	0.560135	2.769496
H	5.001469	0.910853	3.510076
H	3.57492	-0.116815	3.267719
H	4.80789	-0.007976	2.00073
H	1.640624	-1.450534	-1.871234
H	3.09485	0.792073	-2.143618
H	2.215797	-1.343203	-0.219274
C	-4.555746	0.358307	-0.174114
H	-5.005254	1.311539	-0.426649
C	-5.378872	-0.675497	0.268144
H	-6.449695	-0.514189	0.360166
C	-4.822964	-1.9198	0.569527
H	-5.45699	-2.743714	0.886249
C	-3.442921	-2.096295	0.488776
H	-3.022006	-3.056674	0.767275

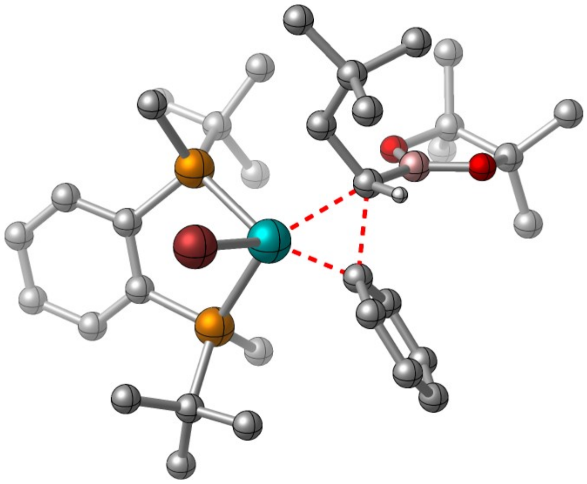

Zero-point correction= 0.851868 (Hartree/Particle)
Thermal correction to Energy= 0.901092
Thermal correction to Enthalpy= 0.902036
Thermal correction to Gibbs Free Energy= 0.770382
Sum of electronic and zero-point Energies= -6022.785991
Sum of electronic and thermal Energies= -6022.736766
Sum of electronic and thermal Enthalpies= -6022.735822
Sum of electronic and thermal Free Energies= -6022.867476

$^{4}TS^{S}_{IV\text{-}I}$C2

E(scf) = -6023.64339924 a.u.

ν_{min} = -306.0 cm^{-1}

C	-2.597882	-1.098327	-0.242263	H	4.526277	-1.354786	0.575092
C	-3.136038	0.147243	-0.643552	H	2.902580	1.609798	3.244050
P	-0.821375	-1.181274	0.233754	H	4.526802	-0.237159	2.808163
P	-2.007474	1.475758	-1.287199	C	-1.954424	-2.004532	2.775285
Fe	0.346645	0.566273	-1.080141	H	-2.976185	-1.707895	2.520901
C	-0.310303	-2.915968	-0.116034	H	-1.853090	-1.945179	3.867717
C	-0.905576	-1.087157	2.128213	H	-1.821314	-3.054564	2.492494
C	-2.729672	1.929246	-3.003306	C	0.491593	-1.482776	2.641935
C	-2.416731	2.925803	-0.216177	H	0.571468	-1.246242	3.711272
C	1.979918	0.876776	0.022856	H	1.290707	-0.951234	2.123358
C	2.935384	-0.150923	-0.214228	H	0.665981	-2.558725	2.527715
C	2.007206	1.501045	1.296438	C	-1.228360	0.374442	2.481711
C	3.819228	-0.553987	0.784006	H	-2.203447	0.673518	2.080261
H	2.990559	-0.601566	-1.200905	H	-0.470312	1.059424	2.092665
C	2.913006	1.107655	2.278494	H	-1.263331	0.496782	3.572406
H	1.329282	2.324075	1.502987	C	-2.930112	0.628562	-3.801865
C	3.823526	0.070586	2.039165	H	-3.713235	-0.001829	-3.366971

H	-2.005588	0.045976	-3.847015	C	3.464625	1.086344	-4.661710
H	-3.230853	0.878385	-4.828361	C	2.218638	1.936939	-5.196148
C	-1.652384	2.783334	-3.695538	B	2.188746	1.830840	-2.890037
H	-1.519212	3.751504	-3.197551	O	1.544818	2.358608	-3.979113
H	-1.965261	2.993095	-4.727799	O	3.296415	1.090004	-3.219119
H	-0.684385	2.282188	-3.733390	C	3.480510	-0.374055	-5.116563
C	-4.039074	2.736844	-2.978914	H	3.592568	-0.451230	-6.203520
H	-4.270671	3.055805	-4.004329	H	4.330664	-0.883151	-4.648926
H	-3.961803	3.642752	-2.367907	H	2.570205	-0.887833	-4.804072
H	-4.893654	2.152606	-2.630113	C	4.828575	1.724760	-4.939632
H	-3.495162	3.046049	-0.073755	H	5.608225	1.120470	-4.463841
H	-2.010789	3.838449	-0.662086	H	5.038805	1.770079	-6.013459
H	-1.947956	2.785203	0.762615	H	4.892074	2.735164	-4.526165
H	-0.571348	-3.150059	-1.150304	C	1.206024	1.138106	-6.020187
H	0.778832	-2.971228	-0.025274	H	0.362478	1.788499	-6.276515
H	-0.765460	-3.649741	0.556068	H	1.651558	0.775119	-6.952599
Br	0.367704	-0.961413	-3.030849	H	0.817877	0.29456	-5.447898
C	2.648727	4.873488	0.179050	C	2.596602	3.212811	-5.95238
H	2.059211	4.781130	1.099886	H	3.111393	2.979501	-6.890586
H	3.399367	4.074480	0.185501	H	1.683358	3.767354	-6.193534
H	3.184812	5.830394	0.219191	H	3.236206	3.866474	-5.354183
C	2.593047	5.035548	-2.330648	H	0.459622	3.331023	-0.185099
H	1.986163	4.926666	-3.235583	H	2.876341	2.405588	-1.019992
H	3.420097	4.318812	-2.397220	H	0.237323	3.507436	-1.909152
H	3.031211	6.041969	-2.325382	C	-4.533296	0.295247	-0.671752
C	1.746858	4.806419	-1.065955	H	-4.969548	1.261534	-0.894483
C	0.680763	5.913270	-0.968388	C	-5.381681	-0.783104	-0.425794
H	0.034579	5.764936	-0.093049	H	-6.458712	-0.64293	-0.463275
H	1.144789	6.903630	-0.875207	C	-4.842404	-2.041861	-0.15266
H	0.040972	5.927158	-1.860391	H	-5.494188	-2.896843	0.00602
C	1.002743	3.442235	-1.132937	C	-3.461039	-2.187854	-0.04091
C	1.875599	2.210264	-1.403214	H	-3.051658	-3.154217	0.236536

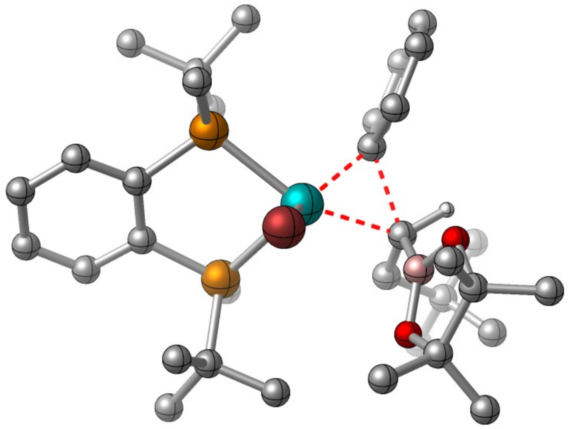

Zero-point correction= 0.851991 (Hartree/Particle)
Thermal correction to Energy= 0.901053
Thermal correction to Enthalpy= 0.901997
Thermal correction to Gibbs Free Energy= 0.771943
Sum of electronic and zero-point Energies= -6022.791408
Sum of electronic and thermal Energies= -6022.742346
Sum of electronic and thermal Enthalpies= -6022.741402
Sum of electronic and thermal Free Energies= -6022.871456

^{4}TS$^S_{IV\text{-}I}$C3

E(scf) = -6023.64897344 a.u.

ν_{min} = -283.8 cm^{-1}

C	-2.113851	-0.904534	0.113333	H	2.719063	2.087024	-2.706824
C	-2.775861	-0.138547	-0.876481	C	1.087589	4.992846	-3.430943
P	-0.449927	-0.386504	0.741603	H	-0.449352	5.876602	-2.191990
P	-1.868637	1.227546	-1.735038	H	2.666558	3.883155	-4.398390
Fe	0.477314	1.111521	-0.982286	H	1.076389	5.791262	-4.168197
C	0.405440	-1.983547	1.051099	C	-1.756520	-0.509483	3.324200
C	-0.784367	0.319355	2.471194	H	-2.764031	-0.528052	2.897702
C	-2.197369	1.076435	-3.607821	H	-1.826706	-0.064172	4.326086
C	-2.842593	2.710333	-1.223432	H	-1.416911	-1.543658	3.448866
C	1.100533	2.892305	-1.518021	C	0.578166	0.387810	3.191511
C	0.252611	4.019257	-1.371615	H	0.476734	0.982422	4.108643
C	2.002227	2.895888	-2.609617	H	1.361335	0.837823	2.576819
C	0.233603	5.038630	-2.319726	H	0.924170	-0.610586	3.481422
H	-0.401079	4.083209	-0.504829	C	-1.368139	1.728972	2.269903
C	1.976426	3.919337	-3.557516	H	-2.330495	1.680982	1.747223

H	-0.699675	2.375031	1.690246	C	2.696344	3.715418	0.419602
H	-1.538955	2.208781	3.242412	C	2.023765	2.337950	0.228024
C	-2.113044	-0.401409	-4.026890	C	4.286444	-0.730752	0.490953
H	-2.947296	-0.990213	-3.631904	C	5.022034	0.161213	-0.604251
H	-1.175645	-0.852772	-3.689164	B	3.079972	1.196216	0.091696
H	-2.145541	-0.466080	-5.122959	O	4.241198	1.385472	-0.619657
C	-1.041393	1.859608	-4.266908	O	3.023769	-0.034526	0.707955
H	-1.016422	2.906125	-3.942963	C	3.976482	-2.155046	0.032497
H	-1.169260	1.849542	-5.357740	H	4.898517	-2.682477	-0.235798
H	-0.073319	1.409854	-4.031131	H	3.498417	-2.711218	0.845806
C	-3.531761	1.682813	-4.074615	H	3.297616	-2.151740	-0.822334
H	-3.602632	1.580289	-5.165946	C	4.992959	-0.766428	1.849599
H	-3.605617	2.750372	-3.842566	H	4.344481	-1.274913	2.571143
H	-4.403119	1.177850	-3.647145	H	5.939432	-1.314284	1.793552
H	-3.914865	2.561460	-1.383568	H	5.192669	0.238583	2.229491
H	-2.511141	3.584700	-1.789541	C	4.972977	-0.410154	-2.024076
H	-2.668834	2.903137	-0.161205	H	5.38193	0.335193	-2.715308
H	0.394412	-2.562763	0.124257	H	5.57361	-1.3219	-2.110738
H	1.441560	-1.746786	1.291431	H	3.946157	-0.624821	-2.325578
H	-0.041887	-2.571748	1.858225	C	6.462628	0.53442	-0.254218
Br	1.248836	-0.777304	-2.377198	H	7.095421	-0.35845	-0.202688
C	3.931992	5.385591	1.795378	H	6.864556	1.191269	-1.032999
H	4.811981	5.321489	1.142723	H	6.528119	1.064932	0.6987
H	3.281835	6.178199	1.402890	H	2.005625	4.521938	0.149853
H	4.274379	5.695840	2.791131	H	3.541743	3.794281	-0.273677
C	1.992668	4.177613	2.819259	H	1.320804	2.184007	1.047147
H	1.451177	3.233131	2.940439	C	-2.765597	-2.028374	0.650704
H	1.280272	4.928980	2.453779	H	-2.263526	-2.639473	1.393192
H	2.327574	4.491293	3.816456	C	-4.059221	-2.370471	0.262804
C	3.190330	4.036444	1.860535	H	-4.540924	-3.244237	0.693562
C	4.152440	2.966649	2.402940	C	-4.73579	-1.57633	-0.664815
H	4.990258	2.805824	1.715170	H	-5.753725	-1.819057	-0.957981
H	4.562595	3.269590	3.375234	C	-4.094595	-0.474698	-1.227052
H	3.645391	2.006617	2.545746	H	-4.629374	0.125416	-1.954528

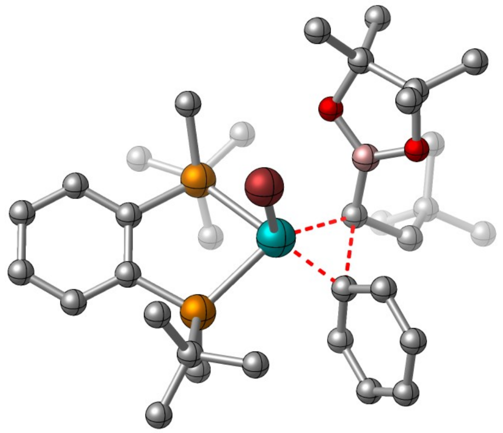

Zero-point correction= 0.852293 (Hartree/Particle)
Thermal correction to Energy= 0.901123
Thermal correction to Enthalpy= 0.902068
Thermal correction to Gibbs Free Energy= 0.772822
Sum of electronic and zero-point Energies= -6022.796680
Sum of electronic and thermal Energies= -6022.747850
Sum of electronic and thermal Enthalpies= -6022.746906
Sum of electronic and thermal Free Energies= -6022.876152

^{5}A

E(scf) = -7716.60352855 a.u.

ν_{min} = 22.4 cm^{-1}

Br	9.587840	6.642992	13.742510	C	2.982068	7.701194	17.147447
Br	8.709389	10.656356	14.301065	H	2.320906	7.493495	17.984325
Fe	8.087286	8.370643	14.331879	C	4.354471	7.494503	17.279755
P	5.815378	8.338238	13.513807	H	4.740687	7.119756	18.221147
P	7.065632	7.626554	16.405613	C	5.232764	7.760850	16.217048
C	4.699078	8.189855	14.975948	C	5.429947	6.845636	12.420898
C	3.317056	8.413865	14.865291	C	5.791209	5.585795	13.229787
H	2.897887	8.763615	13.927193	H	6.846315	5.587561	13.524163
C	2.464228	8.182588	15.943526	H	5.619164	4.694605	12.612714
H	1.397913	8.363168	15.838551	H	5.175420	5.493408	14.131594

C	6.359427	6.963586	11.196675
H	6.129160	7.848631	10.592622
H	6.229884	6.081426	10.556629
H	7.413541	7.009672	11.493842
C	3.965289	6.774699	11.967130
H	3.281645	6.647979	12.812475
H	3.836542	5.907703	11.305687
H	3.662072	7.664216	11.404020
C	5.230641	9.826508	12.609139
H	5.335114	10.692549	13.269642
H	4.192166	9.754054	12.273313
H	5.880307	9.980862	11.742703
C	7.611641	8.614304	17.922000
C	7.021436	10.030558	17.802424
H	7.299548	10.501711	16.853147
H	7.413071	10.657281	18.614102
H	5.928461	10.022156	17.880732
C	7.232915	7.990575	19.273843
H	6.151875	7.980830	19.439460
H	7.677562	8.590239	20.079115
H	7.611799	6.968312	19.379289
C	9.150680	8.679317	17.802011
H	9.605832	7.681939	17.825795
H	9.557548	9.253652	18.643950
H	9.459210	9.171492	16.873609
C	7.341882	5.856521	16.822639
H	7.111972	5.248925	15.943039
H	6.714582	5.529792	17.657822
H	8.395864	5.704733	17.073285

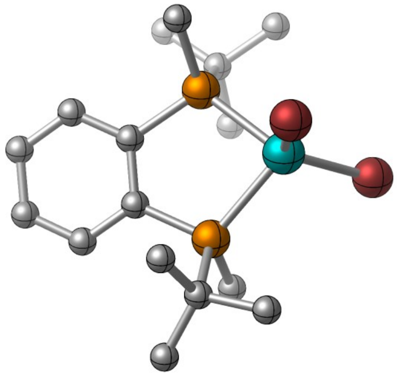

Zero-point correction= 0.409751 (Hartree/Particle)
Thermal correction to Energy= 0.437885
Thermal correction to Enthalpy= 0.438830
Thermal correction to Gibbs Free Energy= 0.349666
Sum of electronic and zero-point Energies= -7716.193778
Sum of electronic and thermal Energies= -7716.165643
Sum of electronic and thermal Enthalpies= -7716.164699
Sum of electronic and thermal Free Energies= -7716.253862

^{5}B

E(scf) = -5376.45924151 a.u.

ν_{min} = 14.2 cm^{-1}

C	-2.117724	-0.578163	-1.035358
C	-2.417533	0.585753	-0.286230
C	-3.711989	1.126807	-0.350213
C	-4.677767	0.577609	-1.192424
C	-4.357535	-0.522277	-1.991234
C	-3.091400	-1.099819	-1.902915
H	-3.968983	1.986247	0.260314
H	-5.672443	1.013200	-1.233035
H	-5.096964	-0.940892	-2.668638
H	-2.866994	-1.970945	-2.510147
P	-1.068611	1.400054	0.676203
P	-0.506580	-1.435734	-0.740170
C	-1.782517	1.633633	2.353245
H	-1.061711	2.171289	2.976127
H	-2.732911	2.176353	2.339399
H	-1.940735	0.644960	2.795071
C	0.048746	-1.960379	-2.414436
H	0.961531	-2.554818	-2.316530
H	-0.701185	-2.542131	-2.958461
H	0.291197	-1.062589	-2.991942
C	-0.952893	-3.022185	0.190919
C	-0.831412	3.124290	-0.060017
C	-2.044051	4.060042	0.036319
H	-1.758544	5.057750	-0.323466
H	-2.876626	3.716730	-0.584753
H	-2.399312	4.171831	1.066958
C	0.352480	3.729875	0.722587
H	0.655428	4.677511	0.259566
H	0.084854	3.938392	1.765029
H	1.218979	3.058162	0.726495
C	-0.438348	2.920586	-1.534929
H	0.419088	2.245099	-1.639265
H	-1.268682	2.505481	-2.117545
H	-0.161957	3.884307	-1.981644
C	0.386922	-3.728396	0.481800
H	0.201258	-4.622559	1.091155
H	0.884656	-4.054455	-0.439061
H	1.071828	-3.079542	1.038711
C	-1.883341	-3.967019	-0.582704
H	-2.037858	-4.881001	0.006426
H	-2.867115	-3.520138	-0.756432
H	-1.461416	-4.267243	-1.548328
C	-1.619421	-2.598933	1.513864
H	-0.962134	-1.953689	2.106435
H	-2.564857	-2.072381	1.339508
H	-1.839534	-3.492371	2.112792
Fe	0.989631	0.097977	0.453722
C	2.635136	0.362469	-0.738537
C	3.177957	1.563516	-1.242817
C	3.334843	-0.818080	-1.082158
C	4.332686	1.593145	-2.034544
H	2.691549	2.511767	-1.014997
C	4.489674	-0.808546	-1.870777
H	2.969781	-1.778350	-0.714426
C	4.992548	0.403613	-2.353530
H	4.718144	2.542649	-2.402546
H	4.997639	-1.741325	-2.110389
H	5.888956	0.420359	-2.969634
Br	1.489491	-0.561860	2.708700

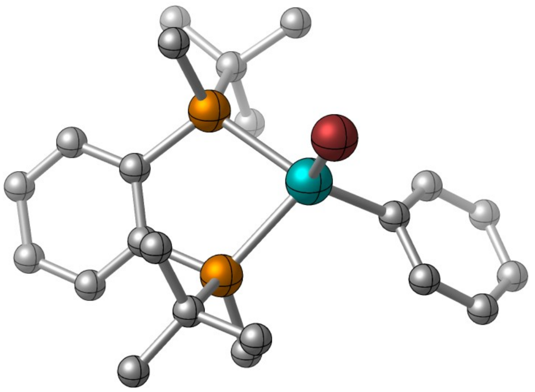

Zero-point correction= 0.498343 (Hartree/Particle)

Thermal correction to Energy= 0.530176
Thermal correction to Enthalpy= 0.531120
Thermal correction to Gibbs Free Energy= 0.433265
Sum of electronic and zero-point Energies= -5375.960899
Sum of electronic and thermal Energies= -5375.929066
Sum of electronic and thermal Enthalpies= -5375.928121
Sum of electronic and thermal Free Energies= -5376.025976

^{5}C

E(scf) = -3036.31336029 a.u.

ν_{min} = 18.9 cm^{-1}

C	-1.945400	-1.030077	0.281139	C	0.259625	3.901718	-1.510884
C	-2.496533	-1.943926	1.195059	C	2.466701	2.996784	-1.418570
C	-2.763804	-0.506967	-0.749363	C	0.778642	5.201479	-1.459105
C	-4.127417	-0.844283	-0.771608	H	-0.822975	3.786493	-1.564668
C	-3.841628	-2.304293	1.129538	C	3.007240	4.286365	-1.359529
H	-4.248493	-3.012397	1.846516	H	3.155438	2.150878	-1.410345
C	-4.665563	-1.733766	0.157140	C	2.159921	5.398363	-1.379855
H	-5.722112	-1.984936	0.117169	H	0.108707	6.059954	-1.478252
H	-1.875156	-2.369909	1.976514	H	4.085556	4.426702	-1.299889
H	-4.780671	-0.406894	-1.518687	H	2.571100	6.404701	-1.336391
P	-0.213742	-0.405840	0.455356	C	0.735822	-1.858018	1.081186
P	-1.995724	0.557182	-2.057092	H	1.796046	-1.589740	1.111617
Fe	0.447267	0.778430	-1.607651	H	0.423872	-2.191962	2.075324
C	-0.298241	0.796311	1.923358	H	0.616328	-2.688834	0.379633
C	-2.369449	-0.250128	-3.735201	C	-3.042584	2.075472	-2.015054
C	1.693793	-0.474168	-2.683931	H	-2.768102	2.729240	-2.848398
C	1.749375	-1.880422	-2.560533	H	-4.111809	1.849097	-2.073435
C	2.568444	0.081808	-3.645088	H	-2.849220	2.607750	-1.079068
C	2.602458	-2.679022	-3.331383	C	1.141973	1.296645	2.148260
H	1.096462	-2.378036	-1.843177	H	1.557237	1.748510	1.240307
C	3.429158	-0.696998	-4.428205	H	1.145044	2.061551	2.935818
H	2.579470	1.162925	-3.787776	H	1.809373	0.488345	2.469357
C	3.447940	-2.086107	-4.273543	C	-1.193948	1.971296	1.491947
H	2.608115	-3.760277	-3.200911	H	-2.222830	1.645656	1.300575
H	4.083842	-0.224094	-5.158812	H	-1.223222	2.725069	2.289565
H	4.113255	-2.698738	-4.878340	H	-0.807169	2.454795	0.589121
C	1.075540	2.750282	-1.492318	C	-0.845126	0.177684	3.217166

H	-0.828401	0.934896	4.012667
H	-1.881088	-0.157245	3.104057
H	-0.242262	-0.669376	3.562429
C	-1.896343	-1.712172	-3.668456
H	-1.995941	-2.175557	-4.658809
H	-0.843601	-1.782396	-3.374457
H	-2.493985	-2.299200	-2.961774
C	-1.502576	0.527165	-4.748103
H	-1.643145	0.105601	-5.751985
H	-1.777615	1.588142	-4.791108
H	-0.438199	0.460009	-4.497601
C	-3.839330	-0.194799	-4.178678
H	-3.921846	-0.606160	-5.193768
H	-4.489439	-0.794983	-3.535742
H	-4.226970	0.829234	-4.209546

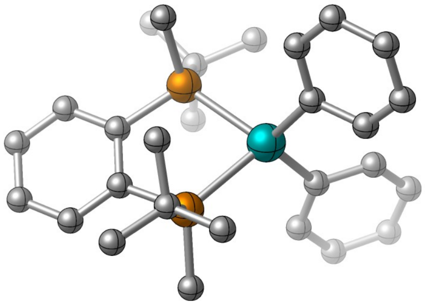

Zero-point correction= 0.587512 (Hartree/Particle)
Thermal correction to Energy= 0.622464
Thermal correction to Enthalpy= 0.623408
Thermal correction to Gibbs Free Energy= 0.521075
Sum of electronic and zero-point Energies= -3035.725848
Sum of electronic and thermal Energies= -3035.690896
Sum of electronic and thermal Enthalpies= -3035.689952
Sum of electronic and thermal Free Energies= -3035.792285

^{6}*I*

E(scf) = -5144.73516290 a.u.

ν_{min} = 19.2 cm^{-1}

C	-2.971777	-2.101675	-0.142459
C	-3.398945	-0.832672	-0.608184
P	-1.198177	-2.575662	-0.351086
P	-2.255602	0.164963	-1.668851
Fe	0.030955	-0.653157	-1.263454
C	-1.269188	-4.157862	-1.292125
C	-0.499752	-3.031252	1.351785
C	-2.953206	0.012852	-3.428272
C	-2.576572	1.908367	-1.166055
C	-1.144225	-4.245862	2.035052
H	-2.174891	-4.045595	2.341697
H	-0.577821	-4.489665	2.943974
H	-1.135602	-5.136208	1.396595
C	0.994352	-3.315435	1.092178
H	1.517712	-3.445067	2.047920
H	1.466561	-2.482897	0.552753
H	1.141550	-4.228208	0.503337
C	-0.639891	-1.787307	2.246056
H	-1.691682	-1.545401	2.439332
H	-0.167745	-0.910230	1.780707
H	-0.149125	-1.965476	3.211740
C	-2.909086	-1.479169	-3.805855
H	-3.555341	-2.080609	-3.156405
H	-1.888684	-1.871958	-3.751183

H	-3.263025	-1.604089	-4.837826	H	-1.673977	-3.945717	-2.285815
C	-1.991338	0.806011	-4.335259	H	-0.255701	-4.550131	-1.415175
H	-1.980654	1.874012	-4.087937	H	-1.895822	-4.912199	-0.805744
H	-2.317267	0.712632	-5.379819	Br	0.923014	-1.606214	-3.299229
H	-0.969388	0.418928	-4.266178	C	-4.715605	-0.418812	-0.351375
C	-4.382996	0.549559	-3.585699	H	-5.048583	0.562567	-0.673749
H	-4.685984	0.460930	-4.638083	C	-5.621263	-1.260574	0.296032
H	-4.463597	1.607126	-3.311113	H	-6.639375	-0.925218	0.474726
H	-5.101665	-0.020572	-2.988696	C	-5.221607	-2.538931	0.687495
H	-3.625604	2.208592	-1.247311	H	-5.928835	-3.212982	1.163372
H	-1.966057	2.567717	-1.791043	C	-3.905534	-2.950250	0.474913
H	-2.250289	2.028599	-0.127661	H	-3.608428	-3.945348	0.787637

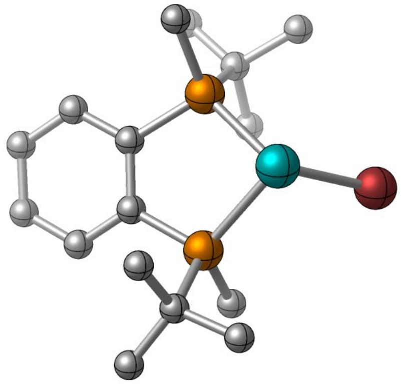

Zero-point correction= 0.408112 (Hartree/Particle)
Thermal correction to Energy= 0.434158
Thermal correction to Enthalpy= 0.435102
Thermal correction to Gibbs Free Energy= 0.351456
Sum of electronic and zero-point Energies= -5144.327051
Sum of electronic and thermal Energies= -5144.301005
Sum of electronic and thermal Enthalpies= -5144.300061
Sum of electronic and thermal Free Energies= -5144.383707

^{6}*IV*^{R}
E(scf) = -6023.64614322 a.u.
ν_{min} = 17.6 cm^{-1}

C	-2.907422	-0.842472	0.250433	P	-1.161168	-1.468757	0.328955
C	-3.182025	0.372160	-0.426587	P	-1.921342	1.144572	-1.536876

Fe	0.571484	0.676203	-1.089037
C	-1.306694	-3.110257	-0.515431
C	-0.821813	-1.945398	2.150919
C	-2.454293	0.617035	-3.291253
C	-2.281124	2.948010	-1.444745
C	1.772472	0.775022	0.563886
C	3.002628	0.089812	0.577056
C	1.470112	1.567840	1.686521
C	3.880911	0.178387	1.662177
H	3.275561	-0.529753	-0.273383
C	2.335386	1.657936	2.782379
H	0.541594	2.138408	1.712804
C	3.545833	0.958871	2.772410
H	4.824127	-0.364630	1.644297
H	2.069243	2.273549	3.639582
H	4.223587	1.025741	3.620418
C	-1.575864	-3.170380	2.696974
H	-2.645555	-2.987265	2.825158
H	-1.176338	-3.417080	3.690380
H	-1.446381	-4.056589	2.066865
C	0.690456	-2.254522	2.193680
H	0.981371	-2.510674	3.221301
H	1.290928	-1.400495	1.876079
H	0.950470	-3.108907	1.556328
C	-1.124838	-0.719274	3.028111
H	-2.195342	-0.484233	3.037723
H	-0.578726	0.166595	2.687876
H	-0.815898	-0.918893	4.063069
C	-2.412767	-0.918904	-3.362590
H	-3.118815	-1.378524	-2.662659
H	-1.408927	-1.299057	-3.155650
H	-2.689671	-1.238533	-4.375569
C	-1.443782	1.206916	-4.294842
H	-1.458301	2.302729	-4.293011
H	-1.719420	0.878117	-5.305509
H	-0.417826	0.876336	-4.107051
C	-3.868603	1.106574	-3.642451
H	-4.108101	0.784471	-4.664421
H	-3.951872	2.198318	-3.617829
H	-4.628384	0.682258	-2.979237
H	-3.319533	3.196281	-1.676857
H	-1.633143	3.467559	-2.150649

H	-2.049411	3.301872	-0.435686
H	-1.439457	-2.946498	-1.586913
H	-0.376453	-3.669534	-0.376552
H	-2.145850	-3.705047	-0.139423
Br	1.269176	-1.200872	-2.408178
C	-5.250023	-0.958417	0.940714
C	-5.496297	0.277999	0.343510
C	1.777339	5.893562	-0.346435
H	0.969172	5.805441	0.391966
H	2.710688	5.588624	0.144021
H	1.870089	6.954299	-0.614491
C	2.672811	5.154086	-2.567060
H	2.462909	4.638186	-3.510904
H	3.587516	4.720974	-2.149034
H	2.864885	6.209106	-2.803548
C	1.494315	5.025179	-1.586956
C	0.216297	5.538973	-2.271049
H	-0.639804	5.517361	-1.585119
H	0.344830	6.575967	-2.608333
H	-0.037702	4.936938	-3.151793
C	1.322103	3.552025	-1.099151
C	1.147375	2.463043	-2.163417
C	4.541919	1.399352	-3.469605
C	3.658910	1.415346	-4.788703
B	2.385108	2.041705	-2.976164
O	2.314326	1.618709	-4.291143
O	3.681744	2.038599	-2.493301
C	4.846378	-0.007611	-2.943494
H	5.522741	-0.550826	-3.612572
H	5.328078	0.079236	-1.963619
H	3.928010	-0.584342	-2.818040
C	5.838684	2.202569	-3.558087
H	6.353958	2.167466	-2.591948
H	6.50916	1.781377	-4.315803
H	5.65254	3.251668	-3.800496
C	3.67288	0.109402	-5.580665
H	3.021534	0.207218	-6.456448
H	4.68274	-0.130685	-5.932124
H	3.297615	-0.718758	-4.975025
C	3.963646	2.596814	-5.716926
H	4.953333	2.508711	-6.177744
H	3.213484	2.623341	-6.514601

H	3.91247	3.547484	-5.176903	H	-6.48399	0.728172	0.393618
H	2.195493	3.304604	-0.485939	C	-3.969855	-1.506177	0.888321
H	0.466912	3.551115	-0.403348	H	-3.798357	-2.47053	1.350068
H	0.318312	2.685938	-2.839995	C	-4.473594	0.924345	-0.347378
H	-6.047826	-1.492271	1.450169	H	-4.697357	1.863831	-0.840091

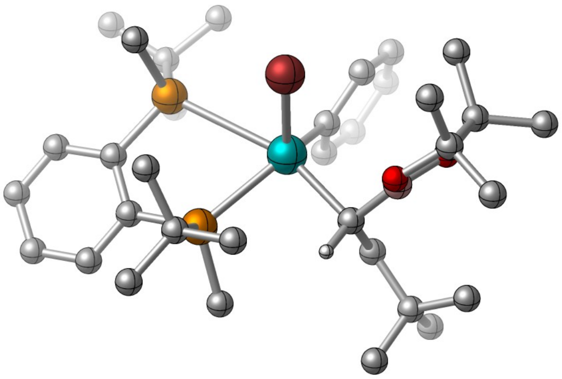

Zero-point correction= 0.851753 (Hartree/Particle)
Thermal correction to Energy= 0.901782
Thermal correction to Enthalpy= 0.902726
Thermal correction to Gibbs Free Energy= 0.767820
Sum of electronic and zero-point Energies= -6022.794390
Sum of electronic and thermal Energies= -6022.744361
Sum of electronic and thermal Enthalpies= -6022.743417
Sum of electronic and thermal Free Energies= -6022.878323

$^{6}\boldsymbol{IV}^{S}$

E(scf) = -6023.64578570 a.u.

ν_{min} = 18.2 cm^{-1}

C	-3.057404	-0.866951	0.360160	C	1.504501	0.323856	0.532788
C	-3.106200	0.456236	-0.143261	C	2.618438	-0.464887	0.872891
P	-1.536846	-1.886494	0.063061	C	1.092360	1.293863	1.465269
P	-1.815638	1.134468	-1.287053	C	3.275477	-0.312192	2.098310
Fe	0.576038	0.279979	-1.278545	H	2.978843	-1.216310	0.172141
C	-2.245577	-3.319017	-0.879709	C	1.743009	1.459406	2.693073
C	-1.052008	-2.682872	1.732413	H	0.241025	1.935313	1.237071
C	-2.559604	0.993869	-3.029749	C	2.836795	0.650243	3.012926
C	-1.794242	2.931851	-0.893936	H	4.129982	-0.941127	2.340253

H	1.398448	2.214404	3.397086
H	3.347198	0.771999	3.965466
C	-1.976482	-3.787009	2.271382
H	-2.951418	-3.403866	2.585227
H	-1.512099	-4.240535	3.158389
H	-2.137135	-4.589532	1.543699
C	0.335778	-3.300527	1.453330
H	0.742780	-3.729305	2.379283
H	1.043443	-2.552104	1.087357
H	0.281414	-4.107474	0.712130
C	-0.916043	-1.565474	2.779810
H	-1.886058	-1.110015	3.011421
H	-0.237531	-0.775261	2.444733
H	-0.509610	-1.979162	3.712841
C	-2.843049	-0.493041	-3.304776
H	-3.613780	-0.888617	-2.634407
H	-1.938022	-1.099959	-3.198711
H	-3.204011	-0.606413	-4.335281
C	-1.494864	1.507698	-4.019800
H	-1.147654	2.519352	-3.782946
H	-1.924436	1.525439	-5.029780
H	-0.622125	0.847404	-4.046669
C	-3.853398	1.807538	-3.185031
H	-4.241762	1.662730	-4.201588
H	-3.687373	2.881523	-3.049841
H	-4.631240	1.482089	-2.487171
H	-2.741862	3.436949	-1.096217
H	-1.007801	3.395624	-1.490222
H	-1.553750	3.049490	0.167081
H	-2.495717	-2.989444	-1.891485
H	-1.486280	-4.103082	-0.963248
H	-3.143340	-3.737842	-0.412453
Br	0.856904	-1.713182	-2.620321
C	3.625455	0.224732	-4.743460
H	3.517564	-0.831323	-4.469296
H	2.636030	0.582562	-5.050645
H	4.288044	0.287636	-5.617152
C	4.335900	2.516180	-3.993243
H	4.680402	3.135019	-3.156522
H	3.380510	2.925931	-4.340538
H	5.057651	2.617403	-4.814730
C	4.189904	1.045069	-3.569236
C	5.575397	0.487228	-3.194827
H	5.501873	-0.556481	-2.861802
H	6.262881	0.517507	-4.050610
H	6.028631	1.066893	-2.380101
C	3.270421	0.908987	-2.319796
C	1.867738	1.522540	-2.452723
C	1.237744	5.243128	-2.194257
C	2.156093	4.978620	-0.945100
B	1.774364	3.007570	-2.068401
O	2.651939	3.640821	-1.203407
O	0.818864	3.896817	-2.560263
C	0.002694	6.095390	-1.919222
H	0.297372	7.105290	-1.612306
H	-0.597479	6.179858	-2.831923
H	-0.629177	5.668606	-1.137249
C	2.002197	5.805655	-3.397954
H	1.352363	5.770823	-4.278735
H	2.305506	6.844813	-3.233577
H	2.894859	5.210267	-3.612321
C	1.382769	4.921907	0.37713
H	2.041935	4.52527	1.155646
H	1.035877	5.913901	0.685622
H	0.517903	4.257316	0.300573
C	3.346566	5.923393	-0.812329
H	3.008037	6.959304	-0.696954
H	3.931545	5.655546	0.074209
H	4.00648	5.865535	-1.681312
H	3.224605	-0.164159	-2.093517
H	1.430363	1.339551	-3.440516
H	3.775236	1.374168	-1.464237
C	-4.217771	1.264803	0.160377
H	-4.267911	2.284727	-0.202872
C	-5.279881	0.779979	0.920514
H	-6.123466	1.427235	1.144539
C	-5.260142	-0.54008	1.371109
H	-6.093715	-0.93908	1.943109
C	-4.160977	-1.347441	1.086384
H	-4.164323	-2.373792	1.433247

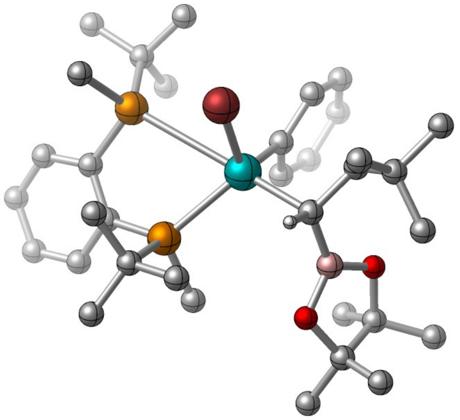

Zero-point correction= 0.851018 (Hartree/Particle)
Thermal correction to Energy= 0.901411
Thermal correction to Enthalpy= 0.902356
Thermal correction to Gibbs Free Energy= 0.766398
Sum of electronic and zero-point Energies= -6022.794768
Sum of electronic and thermal Energies= -6022.744374
Sum of electronic and thermal Enthalpies= -6022.743430
Sum of electronic and thermal Free Energies= -6022.879388

$^{6}TS^{R}_{III-IV}$

E(scf) = -6023.63812318 a.u.

ν_{min} = -150.8 cm^{-1}

C	-2.895455	-0.841933	0.123305	H	0.409938	2.237199	1.800851
C	-3.160294	0.396825	-0.512185	C	3.685767	1.764744	2.558192
P	-1.160062	-1.471971	0.138605	H	5.056563	0.548249	1.418806
P	-1.851174	1.206219	-1.548921	H	2.090726	2.898816	3.469779
Fe	0.438729	0.420233	-0.832178	H	4.431365	2.067771	3.290027
C	-1.279768	-3.080740	-0.757139	C	-1.463719	-3.201606	2.473191
C	-0.718337	-1.976908	1.917165	H	-2.523033	-3.000944	2.654670
C	-2.350037	0.754136	-3.334233	H	-1.020333	-3.474857	3.440360
C	-2.224665	3.003778	-1.380521	H	-1.380051	-4.075540	1.818833
C	1.735676	0.976980	0.638253	C	0.791375	-2.298980	1.866487
C	3.070402	0.527906	0.568463	H	1.151391	-2.507759	2.882394
C	1.421210	1.839069	1.708386	H	1.372180	-1.464581	1.465301
C	4.033964	0.911246	1.507645	H	0.998989	-3.184256	1.253998
H	3.365404	-0.130342	-0.245583	C	-0.963694	-0.763708	2.828998
C	2.370755	2.229835	2.657893	H	-2.026850	-0.500516	2.873665

H	-0.400938	0.112989	2.495013
H	-0.632476	-0.997410	3.849414
C	-2.303148	-0.777553	-3.464719
H	-3.013955	-1.267691	-2.790892
H	-1.298449	-1.160995	-3.263763
H	-2.568751	-1.059436	-4.491864
C	-1.319247	1.372700	-4.296950
H	-1.333853	2.468010	-4.264137
H	-1.575120	1.075928	-5.322935
H	-0.299090	1.032897	-4.096787
C	-3.756280	1.259338	-3.693962
H	-3.975515	0.983534	-4.733974
H	-3.838689	2.349255	-3.622198
H	-4.530385	0.808227	-3.065757
H	-3.270390	3.251830	-1.577550
H	-1.601073	3.564790	-2.077113
H	-1.974216	3.318174	-0.362608
H	-1.431044	-2.890589	-1.820914
H	-0.338679	-3.626023	-0.644131
H	-2.104441	-3.695489	-0.382365
Br	1.407039	-1.156924	-2.360973
C	-5.231167	-0.978940	0.813735
C	-5.476262	0.272292	0.246708
C	1.860741	6.016393	-0.266444
H	1.064034	5.911265	0.481815
H	2.798704	5.686005	0.197682
H	1.959656	7.084159	-0.502447
C	2.705837	5.348325	-2.531394
H	2.477654	4.852439	-3.482129
H	3.628934	4.905121	-2.142422
H	2.893562	6.408760	-2.745570
C	1.548806	5.193293	-1.529347
C	0.251774	5.720675	-2.165285
H	-0.590167	5.656437	-1.464385
H	0.361186	6.773207	-2.458066
H	-0.012318	5.154166	-3.066284
C	1.389946	3.699105	-1.088913
C	1.177936	2.701230	-2.196573
C	4.499439	1.496956	-3.544724
C	3.551611	1.399506	-4.818722
B	2.354476	2.134840	-2.996342
O	2.228884	1.637709	-4.279241
O	3.665662	2.158373	-2.561350
C	4.897087	0.140079	-2.953484
H	5.554540	-0.417131	-3.629895
H	5.434870	0.309783	-2.014384
H	4.014631	-0.462830	-2.733459
C	5.752938	2.348578	-3.746884
H	6.312463	2.39742	-2.80636
H	6.406879	1.909138	-4.508598
H	5.507956	3.371233	-4.043928
C	3.538317	0.031796	-5.499272
H	2.846861	0.055218	-6.349112
H	4.532395	-0.234452	-5.875672
H	3.19286	-0.740443	-4.808772
C	3.797409	2.501346	-5.856033
H	4.769963	2.390371	-6.347243
H	3.01653	2.446738	-6.62213
H	3.747071	3.494325	-5.397867
H	2.286048	3.426567	-0.522514
H	0.555862	3.66576	-0.372228
H	0.255268	2.822313	-2.751352
H	-6.029872	-1.524243	1.309248
H	-6.465382	0.718327	0.305699
C	-3.951731	-1.526879	0.746935
H	-3.777348	-2.503247	1.183111
C	-4.45276	0.942553	-0.420879
H	-4.677057	1.896335	-0.885396

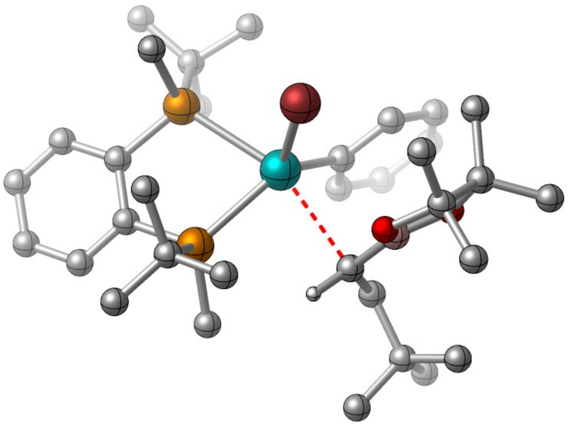

Zero-point correction= 0.850718 (Hartree/Particle)
Thermal correction to Energy= 0.900478
Thermal correction to Enthalpy= 0.901423
Thermal correction to Gibbs Free Energy= 0.767104
Sum of electronic and zero-point Energies= -6022.787406
Sum of electronic and thermal Energies= -6022.737645
Sum of electronic and thermal Enthalpies= -6022.736701
Sum of electronic and thermal Free Energies= -6022.871019

^{6}TS^{R}$_{IV\text{-}I}$

E(scf) = -6023.62490931 a.u.

ν_{min} = -359.3 cm^{-1}

C	-2.737532	-1.520985	0.219663	C	-0.274120	3.532315	2.089160
C	-3.341137	-0.527898	-0.590790	H	-1.170054	3.263653	0.169514
P	-0.902327	-1.492510	0.455446	C	0.781678	3.198495	2.954842
P	-2.266572	0.500074	-1.684333	H	2.613151	2.062158	3.154192
Fe	0.025634	0.459703	-0.771699	H	-1.068239	4.191159	2.438099
C	-0.363902	-3.162309	-0.101988	H	0.811249	3.589784	3.968549
C	-0.558581	-1.445579	2.316350	C	-1.157633	-2.590308	3.146993
C	-2.473359	-0.295011	-3.396453	H	-2.245644	-2.514787	3.226141
C	-3.115628	2.131848	-1.787656	H	-0.754474	-2.535697	4.167218
C	0.688179	2.130323	0.274940	H	-0.901377	-3.577715	2.746807
C	1.751746	1.831217	1.200791	C	0.979406	-1.487424	2.435906
C	-0.327162	3.001953	0.806881	H	1.269376	-1.263812	3.470491
C	1.794772	2.337611	2.490931	H	1.459331	-0.746691	1.789540
H	2.560015	1.182323	0.864733	H	1.378180	-2.476779	2.184995

C	-1.090907	-0.095862	2.828288	H	2.632333	2.704644	-4.005632
H	-2.181207	-0.031753	2.727671	C	0.501970	3.471072	-2.341438
H	-0.646023	0.744485	2.289074	C	1.624227	3.168265	-1.375024
H	-0.844282	0.021545	3.891322	C	2.633410	6.398411	0.310754
C	-1.933893	-1.734442	-3.314383	C	3.921151	5.496983	0.537125
H	-2.517128	-2.345929	-2.617045	B	2.312130	4.315783	-0.616936
H	-0.883702	-1.753265	-3.008194	O	3.651176	4.323250	-0.273633
H	-2.000926	-2.202093	-4.305370	O	1.657523	5.463355	-0.211397
C	-1.604763	0.534921	-4.359364	C	2.055339	7.019266	1.581876
H	-1.998730	1.548984	-4.483197	H	2.775254	7.698689	2.051799
H	-1.599199	0.059809	-5.349100	H	1.158582	7.595896	1.329339
H	-0.568932	0.598984	-4.011967	H	1.766949	6.253130	2.305380
C	-3.926045	-0.320462	-3.893512	C	2.823340	7.487205	-0.752119
H	-3.948529	-0.750290	-4.903889	H	1.855043	7.956390	-0.956445
H	-4.364944	0.681168	-3.956323	H	3.516406	8.264266	-0.412493
H	-4.565310	-0.941465	-3.258284	H	3.197961	7.070192	-1.690556
H	-4.119419	2.076722	-2.218417	C	4.094092	5.019333	1.982149
H	-2.505733	2.808151	-2.393151	H	4.897297	4.275316	2.016117
H	-3.189292	2.548424	-0.778497	H	4.363124	5.846864	2.647678
H	-0.561228	-3.252578	-1.173245	H	3.184521	4.546146	2.355813
H	0.713978	-3.263845	0.051221	C	5.23	6.115634	0.046945
H	-0.886347	-3.965910	0.427074	H	5.457744	7.036959	0.594693
Br	1.599112	-0.575646	-2.263197	H	6.05094	5.409724	0.214096
C	-0.371593	4.385284	-4.506742	H	5.196143	6.342751	-1.02146
H	-1.016173	3.506258	-4.624824	H	-0.186721	4.190577	-1.878239
H	-0.949006	5.157898	-3.982068	H	-0.077133	2.556233	-2.527857
H	-0.141906	4.764638	-5.511107	C	-4.74216	-0.446142	-0.645801
C	1.764808	5.310283	-3.596574	H	-5.217514	0.325729	-1.242728
H	2.734674	5.094206	-3.134502	C	-5.541683	-1.360752	0.038311
H	1.258426	6.056989	-2.975684	H	-6.624004	-1.280813	-0.016006
H	1.959129	5.756601	-4.580867	C	-4.946758	-2.395029	0.763433
C	0.915331	4.036089	-3.737127	H	-5.561743	-3.136965	1.265585
C	1.708463	2.980330	-4.526151	C	-3.557352	-2.470948	0.851192
H	1.122756	2.064131	-4.664986	H	-3.109995	-3.2815	1.414864
H	1.982932	3.360769	-5.519068	H	2.264442	2.341001	-1.67748

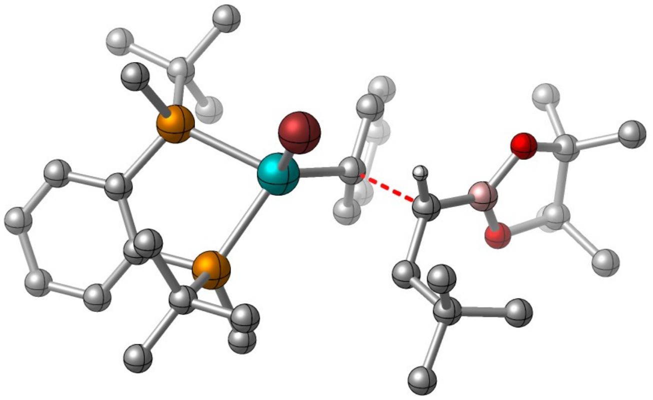

Zero-point correction= 0.849743 (Hartree/Particle)
Thermal correction to Energy= 0.899427
Thermal correction to Enthalpy= 0.900371
Thermal correction to Gibbs Free Energy= 0.767400
Sum of electronic and zero-point Energies= -6022.775167
Sum of electronic and thermal Energies= -6022.725483
Sum of electronic and thermal Enthalpies= -6022.724539
Sum of electronic and thermal Free Energies= -6022.857509

^{6}TS^{S}$_{III\text{-}IV}$

E(scf) = -6023.63882144 a.u.

ν_{min} = -210.9 cm^{-1}

C	-3.022386	-0.836323	0.262569
C	-3.078158	0.502766	-0.193366
P	-1.481409	-1.801617	-0.039389
P	-1.743827	1.187722	-1.285717
Fe	0.391685	-0.143730	-1.016881
C	-2.069531	-3.216744	-1.068165
C	-0.954439	-2.622793	1.592785
C	-2.492662	1.103691	-3.035557
C	-1.727734	2.970680	-0.842680
C	1.590766	0.287611	0.572279
C	2.803943	-0.359131	0.878855
C	1.203045	1.337621	1.428679
C	3.581461	0.008080	1.981337
H	3.155681	-1.166546	0.237364
C	1.969960	1.719908	2.535390
H	0.276689	1.876233	1.229996
C	3.164816	1.051294	2.814570
H	4.513120	-0.514325	2.190685
H	1.639147	2.535404	3.175966
H	3.768265	1.342020	3.671529
C	-1.852129	-3.773500	2.077812
H	-2.844816	-3.432431	2.384006
H	-1.386931	-4.236015	2.958971
H	-1.970049	-4.558046	1.323251
C	0.453862	-3.184871	1.302240
H	0.861064	-3.638111	2.215601
H	1.141529	-2.399740	0.979913
H	0.433986	-3.959570	0.526561
C	-0.871289	-1.537250	2.678745
H	-1.859928	-1.125694	2.912667
H	-0.214907	-0.713557	2.382311
H	-0.462610	-1.972039	3.600496
C	-2.823279	-0.366708	-3.343070
H	-3.591171	-0.763739	-2.670477
H	-1.931281	-0.998048	-3.273968
H	-3.204460	-0.443417	-4.369674
C	-1.420934	1.597312	-4.026124
H	-1.019498	2.582073	-3.765284

H	-1.865058	1.666266	-5.027882
H	-0.586531	0.892715	-4.084770
C	-3.760504	1.960221	-3.175007
H	-4.153333	1.845283	-4.193817
H	-3.560120	3.025953	-3.022425
H	-4.549412	1.648517	-2.483544
H	-2.692131	3.461717	-0.995365
H	-0.974083	3.465920	-1.454098
H	-1.450204	3.063974	0.211612
H	-2.300941	-2.858862	-2.073040
H	-1.270885	-3.958871	-1.150730
H	-2.961624	-3.686750	-0.641536
Br	0.866588	-1.809740	-2.695949
C	3.521193	0.218743	-4.673825
H	3.256666	-0.810366	-4.406040
H	2.608107	0.705489	-5.035481
H	4.235799	0.189416	-5.507335
C	4.426659	2.420186	-3.864515
H	4.799536	2.993552	-3.007677
H	3.528264	2.926218	-4.236950
H	5.183789	2.457777	-4.659111
C	4.119649	0.965877	-3.469089
C	5.423765	0.268141	-3.043058
H	5.233659	-0.771792	-2.746742
H	6.154123	0.255016	-3.862983
H	5.886671	0.780154	-2.189432
C	3.141815	0.914668	-2.249454
C	1.811479	1.600661	-2.460004
C	1.208502	5.315646	-2.251889
C	2.147364	5.063895	-1.014637
B	1.707079	3.075489	-2.082447
O	2.614372	3.712311	-1.249390
O	0.759482	3.969868	-2.579902
C	-0.008406	6.191818	-1.972187
H	0.305563	7.202394	-1.687569
H	-0.622633	6.268963	-2.876159
H	-0.632593	5.786080	-1.172767
C	1.958226	5.836185	-3.483427
H	1.290052	5.793485	-4.349979
H	2.284934	6.872613	-3.349582
H	2.835023	5.218377	-3.701443
C	1.4021	5.053698	0.325133
H	2.074116	4.669883	1.099005
H	1.075599	6.058662	0.613477
H	0.527727	4.398365	0.287078
C	3.357008	5.990047	-0.930337
H	3.039603	7.034698	-0.835532
H	3.954999	5.734347	-0.048919
H	3.99786	5.897031	-1.810459
H	3.013758	-0.147515	-2.011417
H	1.254839	1.267235	-3.330348
H	3.646999	1.363785	-1.386906
C	-4.202706	1.282626	0.130583
H	-4.259297	2.317726	-0.186502
C	-5.269934	0.748965	0.850689
H	-6.126594	1.373667	1.088816
C	-5.241461	-0.589686	1.244052
H	-6.080243	-1.023211	1.781952
C	-4.125518	-1.370065	0.949485
H	-4.116577	-2.409181	1.25656

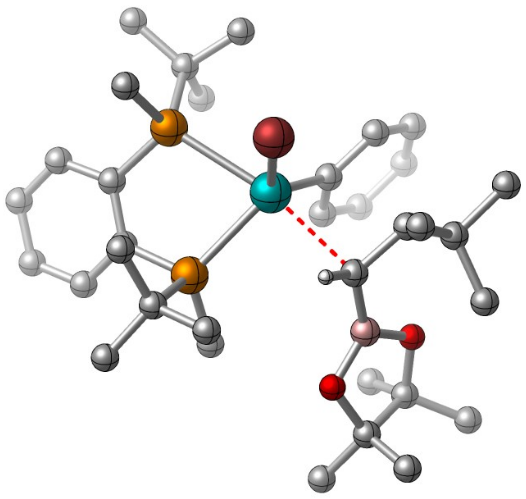

Zero-point correction= 0.850750 (Hartree/Particle)
Thermal correction to Energy= 0.900528
Thermal correction to Enthalpy= 0.901472
Thermal correction to Gibbs Free Energy= 0.767510
Sum of electronic and zero-point Energies= -6022.788071
Sum of electronic and thermal Energies= -6022.738293
Sum of electronic and thermal Enthalpies= -6022.737349
Sum of electronic and thermal Free Energies= -6022.871312

^{6}TS$^{S}_{IV\text{-}I}$

E(scf) = -6023.62601101 a.u.

ν_{min} = -354.6 cm^{-1}

C	-2.788807	-1.248905	-0.091979	H	0.414732	2.613511	1.224732
C	-3.127819	0.013093	-0.638541	C	3.045324	1.388915	3.002378
P	-1.044481	-1.560317	0.421025	H	4.454802	-0.075546	2.266548
P	-1.780167	1.169819	-1.169368	H	1.441214	2.776527	3.439334
Fe	0.379606	0.081158	-0.676238	H	3.506233	1.472109	3.983049
C	-0.735747	-3.334300	0.052848	C	-1.986687	-2.429483	2.998731
C	-1.070656	-1.409388	2.308312	H	-3.035746	-2.294296	2.717047
C	-2.102609	1.592343	-2.995375	H	-1.918001	-2.295369	4.086595
C	-2.137691	2.708222	-0.224598	H	-1.697367	-3.462692	2.777157
C	1.814466	1.163385	0.390522	C	0.384456	-1.605139	2.775769
C	2.980444	0.403884	0.781627	H	0.439937	-1.467963	3.863429
C	1.301127	2.018977	1.437320	H	1.060772	-0.879999	2.312830
C	3.579080	0.527109	2.028486	H	0.753422	-2.612480	2.549430
H	3.401290	-0.308429	0.074898	C	-1.533131	0.019431	2.644189
C	1.886468	2.126013	2.688070	H	-2.560981	0.204651	2.313599

H	-0.876689	0.768062	2.189545
H	-1.497003	0.169562	3.731011
C	-2.385897	0.279720	-3.748207
H	-3.346246	-0.158906	-3.456680
H	-1.597709	-0.461887	-3.574063
H	-2.420422	0.481579	-4.826874
C	-0.785862	2.206543	-3.515841
H	-0.468693	3.071916	-2.922318
H	-0.926444	2.540417	-4.552472
H	0.020585	1.468190	-3.509529
C	-3.242245	2.597398	-3.226001
H	-3.316552	2.805976	-4.301657
H	-3.057685	3.551578	-2.720684
H	-4.215830	2.216593	-2.905565
H	-3.158055	3.067474	-0.390623
H	-1.414727	3.475175	-0.513469
H	-2.011143	2.496687	0.841598
H	-0.788118	-3.472218	-1.031198
H	0.281132	-3.578694	0.374953
H	-1.439760	-4.016473	0.538029
Br	1.162278	-1.124601	-2.606180
C	5.497237	1.802289	-3.412111
H	6.213529	2.141368	-2.652224
H	5.476856	0.704912	-3.387343
H	5.881743	2.107484	-4.394166
C	3.134610	1.878571	-4.246522
H	2.146494	2.343861	-4.158520
H	2.991337	0.794396	-4.174897
H	3.522946	2.112993	-5.246733
C	4.095422	2.387893	-3.156437
C	4.174197	3.922201	-3.226964
H	4.793606	4.330340	-2.420126
H	4.601825	4.244216	-4.185885
H	3.180583	4.375082	-3.140658
C	3.637148	1.895855	-1.748557
C	2.253746	2.316623	-1.301392
C	0.868131	5.749110	-0.603032
C	2.225226	5.795384	0.201737
B	1.980776	3.747340	-0.813847
O	2.912242	4.599755	-0.258433
O	0.721690	4.333000	-0.893562
C	-0.359203	6.216398	0.174181
H	-0.250676	7.264043	0.476361
H	-1.249265	6.137879	-0.459838
H	-0.527084	5.610941	1.068030
C	0.934392	6.469463	-1.954515
H	0.031236	6.233063	-2.527121
H	0.989937	7.556011	-1.831029
H	1.799286	6.140222	-2.5384
C	2.036411	5.655744	1.715226
H	3.012262	5.483878	2.180819
H	1.59878	6.561352	2.149046
H	1.398385	4.803406	1.956945
C	3.108228	7.0023	-0.102672
H	2.595158	7.934862	0.158077
H	4.027361	6.942849	0.490272
H	3.39036	7.040252	-1.157618
H	3.689636	0.800522	-1.777094
H	4.378575	2.228027	-1.009207
H	1.478595	1.965358	-1.978069
C	-4.483726	0.325669	-0.829699
H	-4.76652	1.30726	-1.193199
C	-5.484202	-0.607744	-0.562403
H	-6.526062	-0.346056	-0.726424
C	-5.141808	-1.879239	-0.098231
H	-5.913348	-2.620915	0.089832
C	-3.804995	-2.188704	0.147188
H	-3.554902	-3.169539	0.538974

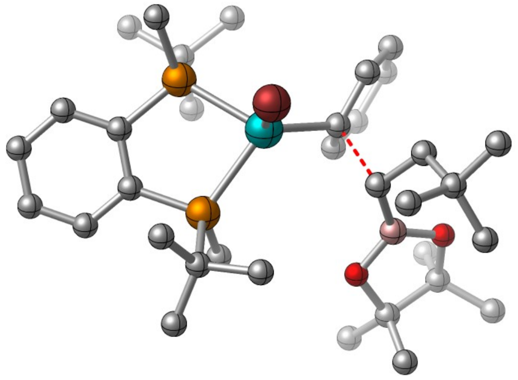

Zero-point correction= 0.849491 (Hartree/Particle)
Thermal correction to Energy= 0.899390
Thermal correction to Enthalpy= 0.900334
Thermal correction to Gibbs Free Energy= 0.765845
Sum of electronic and zero-point Energies= -6022.776521
Sum of electronic and thermal Energies= -6022.726621
Sum of electronic and thermal Enthalpies= -6022.725677
Sum of electronic and thermal Free Energies= -6022.860166

II

E(scf) = -157.807071705 a.u.

ν_{min} = 135.5 cm^{-1}

C	1.602924	6.181018	-0.081549
H	0.673653	5.899665	0.431413
H	2.448419	5.895754	0.558419
H	1.608940	7.287357	-0.132400
C	3.037162	5.516827	-2.111940
H	3.054676	4.798716	-2.942483
H	3.837851	5.249365	-1.409671
H	3.314309	6.498977	-2.542233
C	1.699015	5.563388	-1.442019
C	0.470866	5.526252	-2.297558
H	-0.424764	5.267004	-1.717532
H	0.266964	6.509577	-2.764559
H	0.567066	4.806266	-3.121082

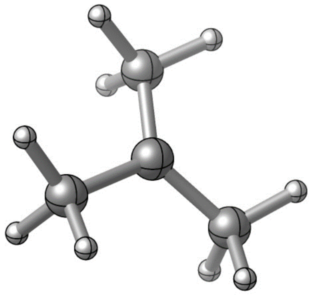

Zero-point correction= 0.117111 (Hartree/Particle)
Thermal correction to Energy= 0.123410
Thermal correction to Enthalpy= 0.124354

Thermal correction to Gibbs Free Energy= 0.087770
Sum of electronic and zero-point Energies= -157.689961
Sum of electronic and thermal Energies= -157.683662
Sum of electronic and thermal Enthalpies= -157.682718
Sum of electronic and thermal Free Energies= -157.719301

III

E(scf) = -647.163183679 a.u.

ν_{min} = 27.2 cm^{-1}

C	1.603502	6.157851	-0.113383	C	5.106523	1.777156	-4.573719
H	0.676484	5.987906	0.449169	H	6.162702	1.618064	-4.814469
H	2.446567	5.994105	0.569903	H	4.513316	1.573214	-5.471076
H	1.620608	7.211602	-0.420003	H	4.964134	2.829084	-4.304853
C	3.007879	5.496054	-2.086730	C	4.849529	-0.608561	-3.841558
H	3.079631	4.884107	-2.993571	H	4.409290	-0.791476	-4.827792
H	3.875966	5.261797	-1.459163	H	5.918711	-0.841895	-3.897799
H	3.077437	6.549646	-2.386093	H	4.379348	-1.291381	-3.129720
C	1.691194	5.230185	-1.336812	C	6.605023	1.885008	-2.068971
C	0.499005	5.501312	-2.269704	H	6.919774	2.132892	-1.049464
H	-0.454323	5.300877	-1.763673	H	7.340279	1.191978	-2.493019
H	0.491403	6.549596	-2.594546	H	6.611404	2.805822	-2.657246
H	0.541730	4.875128	-3.168587	C	5.188579	0.095469	-1.026785
C	1.655560	3.747741	-0.825807	H	5.932420	-0.671604	-1.265635
C	1.764765	2.710368	-1.896364	H	5.409078	0.491933	-0.030292
C	4.640440	0.848959	-3.446553	H	4.200063	-0.374321	-0.993817
C	5.221004	1.246058	-2.038718	H	2.479305	3.627830	-0.110293
B	3.076749	2.005936	-2.251285	H	0.714605	3.619686	-0.268671
O	4.259582	2.237562	-1.575508	H	0.864748	2.501489	-2.474978
O	3.212460	1.087625	-3.273404				

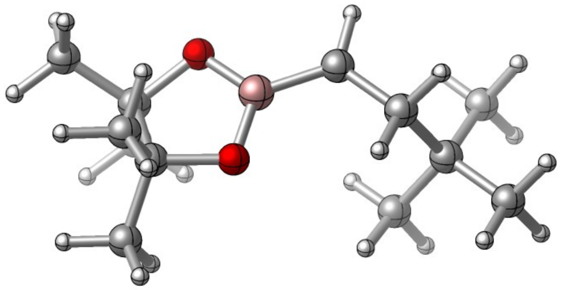

Zero-point correction= 0.349341 (Hartree/Particle)
Thermal correction to Energy= 0.366891
Thermal correction to Enthalpy= 0.367835
Thermal correction to Gibbs Free Energy= 0.304101

Sum of electronic and zero-point Energies= -646.813843
Sum of electronic and thermal Energies= -646.796293
Sum of electronic and thermal Enthalpies= -646.795349
Sum of electronic and thermal Free Energies= -646.859083

TS*II-III*
E(scf) = -647.124259477 a.u.
ν_{min} = -275.2 cm^{-1}

C	2.006961	6.201449	-0.286709	C	4.986749	1.799211	-4.387422
H	1.116742	6.115485	0.349016	H	6.052650	1.726522	-4.627120
H	2.876046	5.854197	0.284956	H	4.413710	1.601246	-5.299168
H	2.158513	7.278560	-0.481226	H	4.769588	2.823263	-4.065969
C	3.090433	5.202502	-2.378825	C	4.894132	-0.632948	-3.778922
H	2.899647	4.507156	-3.204760	H	4.473647	-0.796223	-4.777174
H	3.897278	4.785567	-1.766565	H	5.977383	-0.787867	-3.836691
H	3.458366	6.145012	-2.821435	H	4.469120	-1.382501	-3.106881
C	1.847618	5.447434	-1.574744	C	6.457725	1.882167	-1.865879
C	0.586224	5.689854	-2.351978	H	6.746172	2.101992	-0.832213
H	-0.297047	5.719427	-1.702445	H	7.242641	1.262204	-2.313509
H	0.632958	6.659438	-2.879392	H	6.406054	2.828342	-2.410153
H	0.430438	4.916283	-3.113856	C	5.160657	-0.049547	-0.928243
C	1.423131	3.286975	-0.709161	H	5.957787	-0.750939	-1.195705
C	1.561859	2.372012	-1.714697	H	5.344992	0.311191	0.089000
C	4.579819	0.784237	-3.313150	H	4.206786	-0.587279	-0.929280
C	5.120493	1.149728	-1.881821	H	2.240880	3.457471	-0.012885
B	2.929213	1.770678	-2.080734	H	0.448037	3.645361	-0.384952
O	4.090338	2.048983	-1.383249	H	0.688043	2.134483	-2.323730
O	3.138105	0.913809	-3.142358				

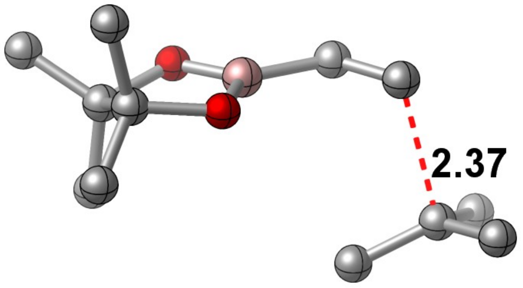

Zero-point correction= 0.345100 (Hartree/Particle)
Thermal correction to Energy= 0.363425
Thermal correction to Enthalpy= 0.364369
Thermal correction to Gibbs Free Energy= 0.298967

Sum of electronic and zero-point Energies= -646.779159
Sum of electronic and thermal Energies= -646.760835
Sum of electronic and thermal Enthalpies= -646.759890
Sum of electronic and thermal Free Energies= -646.825292

^1B_Q
E(scf) = -5562.14238363 a.u.
ν_{min} = 17.49 cm^{-1}

Br	3.188392	6.103958	12.880795
Fe	4.366464	8.003215	13.625668
P	5.378936	7.788525	15.581599
P	6.367482	7.824525	12.598098
C	7.002193	6.972560	15.203101
C	7.413403	6.923221	13.831099
C	4.497089	6.633446	16.801520
C	4.570449	5.188475	16.277433
H	4.200428	5.112906	15.251032
H	3.942055	4.546683	16.908641
H	5.591861	4.795903	16.314117
C	3.030396	7.110168	16.816241
H	2.946806	8.160832	17.121172
H	2.452598	6.506994	17.528371
H	2.568081	7.002802	15.828151
C	5.081457	6.696996	18.222985
H	6.141948	6.430884	18.235126
H	4.542338	5.981230	18.857965
H	4.963912	7.687106	18.675499
C	5.945755	9.194257	16.629994
H	6.383165	9.967419	15.995324
H	6.698441	8.851702	17.345977
H	5.102055	9.621327	17.178754
C	7.400356	9.335263	12.122161
C	7.506161	10.232549	13.366556
H	6.518183	10.551776	13.711093
H	8.087157	11.130468	13.119989
H	8.016710	9.722140	14.191764
C	8.806538	8.961603	11.624246
H	9.388805	8.432362	12.383898
H	9.345551	9.883265	11.367425
H	8.773795	8.338939	10.724129
C	6.639103	10.075220	11.006525
H	6.536523	9.461859	10.103925
H	7.198148	10.978438	10.729367
H	5.642286	10.385842	11.331288
C	6.428644	6.694013	11.150923
H	5.925256	5.765622	11.433432
H	7.453935	6.473301	10.843560
H	5.876677	7.140668	10.318620
C	3.889760	9.864850	13.575522
C	3.288069	10.131004	12.319620
H	3.341208	9.387480	11.520219
C	2.632131	11.332851	12.040930
H	2.189059	11.496274	11.060526
C	2.547359	12.324634	13.023176
H	2.039297	13.263112	12.814594
C	3.122694	12.093682	14.274276
H	3.058777	12.855077	15.049623
C	3.780589	10.886464	14.539204
H	4.214704	10.749935	15.524010
N	8.502676	6.283153	13.452377
N	7.726729	6.437795	16.165945
C	9.681760	5.186830	16.797655
C	10.822519	4.508615	16.428301
C	11.195123	4.403569	15.062092
C	10.426191	4.980890	14.075672
C	9.249218	5.688980	14.426216
C	8.868992	5.787573	15.803832
H	9.376135	5.273703	17.836127
H	11.446956	4.044893	17.186536
H	12.098642	3.861570	14.797934
H	10.695177	4.916002	13.025559

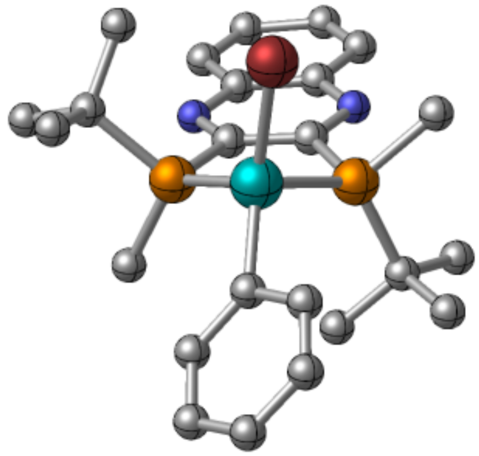

Zero-point correction= 0.521730 (Hartree/Particle)
Thermal correction to Energy= 0.555552
Thermal correction to Enthalpy= 0.556496
Thermal correction to Gibbs Free Energy= 0.456658
Sum of electronic and zero-point Energies= -5561.620654
Sum of electronic and thermal Energies= -5561.586831
Sum of electronic and thermal Enthalpies= -5561.585887
Sum of electronic and thermal Free Energies= -5561.685726

$^{3}B_{Q}$
E(scf) = -5562.17020917 a.u.
ν_{min} = 14.01 cm^{-1}

C	-2.041058	-0.818166	0.562935
C	-2.678531	-0.619452	-0.706158
P	-0.236173	-0.435284	0.643365
P	-1.579085	-0.263687	-2.156585
Fe	0.520489	0.136311	-1.478153
C	-0.157267	1.112499	1.727648
C	0.457432	-1.812401	1.631325
H	1.479797	-1.562930	1.925215
H	-0.151713	-2.020193	2.515406
H	0.493384	-2.702408	0.995847
C	-2.544554	0.980195	-3.096750
H	-2.090239	1.124897	-4.080251
H	-3.587139	0.670789	-3.204705
H	-2.510416	1.932592	-2.559337
C	-1.666844	-1.877353	-3.152602
C	1.006549	1.154238	-3.094424
C	1.878587	0.737514	-4.120863
C	0.497038	2.466360	-3.212884
C	2.206927	1.562149	-5.200810
H	2.319436	-0.255075	-4.066313
C	0.831655	3.311385	-4.277617
H	-0.190526	2.849562	-2.456484
C	1.686439	2.857866	-5.285026
H	2.879897	1.197673	-5.975526
H	0.420209	4.318450	-4.323979
H	1.944828	3.503093	-6.121714
C	1.305490	1.596914	1.698176
H	1.626887	1.855364	0.682241
H	1.405032	2.492649	2.324763
H	1.996641	0.838567	2.081731
C	-1.062951	2.173758	1.075456
H	-0.783960	2.353628	0.029144
H	-2.119914	1.886265	1.102558
H	-0.960405	3.124471	1.613516

C	-0.598288	0.850343	3.174517	H	-3.351330	-1.363953	-4.458675
H	0.069751	0.143817	3.679188	N	-2.713539	-1.207240	1.629318
H	-0.568932	1.793455	3.736685	N	-3.983483	-0.736926	-0.857047
H	-1.617169	0.454818	3.226448	C	-6.843104	-1.641686	1.235966
C	-1.262318	-3.030100	-2.213575	C	-6.114427	-1.240297	0.137833
H	-1.224545	-3.964723	-2.787203	C	-4.707701	-1.100754	0.240361
H	-0.270113	-2.869099	-1.775013	C	-4.060996	-1.369071	1.490491
H	-1.980933	-3.168973	-1.398010	C	-4.836033	-1.785347	2.601838
C	-0.644253	-1.765938	-4.297592	C	-6.201203	-1.917248	2.472039
H	0.376508	-1.671434	-3.915408	H	-7.920926	-1.752939	1.160262
H	-0.694057	-2.671591	-4.915844	H	-6.585037	-1.030865	-0.818267
H	-0.842076	-0.904364	-4.944793	H	-4.326236	-1.985585	3.539608
C	-3.070373	-2.127579	-3.725479	H	-6.798547	-2.233551	3.322316
H	-3.075874	-3.097398	-4.240390	Br	2.667903	-0.902981	-1.132439
H	-3.836814	-2.153632	-2.945225				

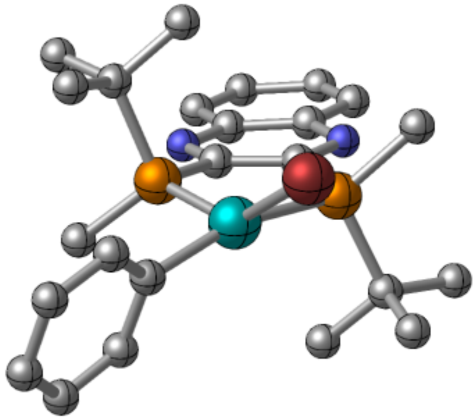

Zero-point correction= 0.521128 (Hartree/Particle)
Thermal correction to Energy= 0.555018
Thermal correction to Enthalpy= 0.555962
Thermal correction to Gibbs Free Energy= 0.454992
Sum of electronic and zero-point Energies= -5561.649081
Sum of electronic and thermal Energies= -5561.615192
Sum of electronic and thermal Enthalpies= -5561.614247
Sum of electronic and thermal Free Energies= -5561.715217

$^{5}B_{Q}$

E(scf) = -5562.18442703 a.u.

ν_{min} = 18.78 cm^{-1}

C	-1.950870	-0.912978	0.486566	P	-1.913818	-0.621894	-2.355584
C	-2.735111	-0.950026	-0.720117	Fe	0.485084	-0.355031	-1.934132
P	-0.177236	-0.369001	0.410672	C	-0.188552	1.351769	1.191982

C	0.634946	-1.488756	1.617366
H	1.663618	-1.151147	1.776127
H	0.099565	-1.524510	2.569757
H	0.668087	-2.492864	1.183300
C	-2.795874	0.899436	-2.906169
H	-2.512424	1.128824	-3.937939
H	-3.881105	0.777674	-2.841112
H	-2.494452	1.736169	-2.269839
C	-2.479556	-1.997964	-3.519798
C	1.488887	1.198357	-2.775530
C	2.866817	1.049955	-3.051644
C	0.927883	2.453945	-3.095259
C	3.638111	2.083610	-3.593505
H	3.347940	0.095566	-2.839136
C	1.682703	3.497862	-3.642086
H	-0.133931	2.628589	-2.917629
C	3.046173	3.315359	-3.888892
H	4.698329	1.930708	-3.788089
H	1.211388	4.451331	-3.874791
H	3.640470	4.122996	-4.310663
C	1.258755	1.874722	1.106315
H	1.605602	1.931589	0.068212
H	1.304985	2.885086	1.533006
H	1.956175	1.244387	1.670027
C	-1.102543	2.233887	0.323240
H	-0.758194	2.255604	-0.716528
H	-2.143096	1.890325	0.341386
H	-1.083958	3.264424	0.699931
C	-0.667617	1.352803	2.650297
H	-0.007279	0.763430	3.295748
H	-0.666194	2.383835	3.028589
H	-1.682878	0.957163	2.749775
C	-2.232507	-3.347941	-2.821852
H	-2.434523	-4.161409	-3.530814
H	-1.192380	-3.442614	-2.491746
H	-2.892708	-3.486329	-1.958689
C	-1.560979	-1.876197	-4.753393
H	-0.510281	-2.028502	-4.483148
H	-1.839471	-2.639816	-5.491286
H	-1.655240	-0.896164	-5.237305
C	-3.950971	-1.877318	-3.946220
H	-4.202577	-2.722374	-4.601150
H	-4.624548	-1.898058	-3.085406
H	-4.138094	-0.957909	-4.511942
N	-2.472061	-1.233508	1.657412
N	-4.021940	-1.246233	-0.695378
C	-6.518949	-2.216722	1.791180
C	-5.960937	-1.881924	0.577517
C	-4.582882	-1.555350	0.505689
C	-3.790242	-1.570569	1.697360
C	-4.390567	-1.924196	2.932523
C	-5.730441	-2.239838	2.972777
H	-7.573683	-2.469051	1.851904
H	-6.544992	-1.863333	-0.337790
H	-3.770799	-1.931661	3.824224
H	-6.196210	-2.508947	3.916417
Br	1.518946	-2.499871	-2.243680

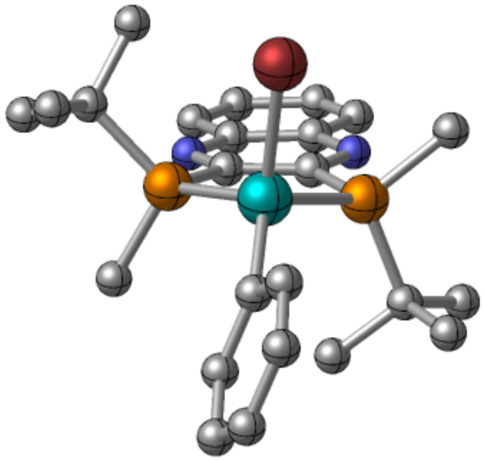

Zero-point correction= 0.520646 (Hartree/Particle)
Thermal correction to Energy= 0.554994
Thermal correction to Enthalpy= 0.555938
Thermal correction to Gibbs Free Energy= 0.452756
Sum of electronic and zero-point Energies= -5561.663781
Sum of electronic and thermal Energies= -5561.629433
Sum of electronic and thermal Enthalpies= -5561.628489
Sum of electronic and thermal Free Energies= -5561.731671

$^{2}TS_{Q}{}^{R}{}_{III\text{-}IV}$
E(scf) = -6209.34795377 a.u.
ν_{min} = -99.07 cm^{-1}

C	-2.383744	-0.615487	0.254634	H	0.070371	0.033210	2.784225
C	-2.723559	0.556021	-0.516692	H	-0.125118	-1.313947	3.909776
P	-0.653437	-1.269895	0.131301	C	-2.244174	-0.817877	-3.440113
P	-1.474775	1.262268	-1.711406	H	-2.884452	-1.226282	-2.650089
Fe	0.479662	0.571799	-0.985240	H	-1.245288	-1.253813	-3.352202
C	-0.876966	-2.823981	-0.840890	H	-2.662286	-1.136629	-4.403613
C	-0.164092	-1.933824	1.844554	C	-1.211898	1.192133	-4.503211
C	-2.171748	0.714760	-3.400720	H	-1.074888	2.277919	-4.498036
C	-1.833353	3.060733	-1.668926	H	-1.628634	0.915713	-5.480840
C	0.476578	1.597776	0.677564	H	-0.231919	0.717801	-4.410576
C	1.777757	1.530248	1.216635	C	-3.570372	1.309487	-3.647553
C	-0.499340	2.242037	1.456156	H	-3.943984	0.936500	-4.610468
C	2.088262	2.053672	2.472682	H	-3.548648	2.402432	-3.706132
H	2.583448	1.056904	0.650223	H	-4.288756	1.022986	-2.874324
C	-0.199953	2.779678	2.713849	H	-2.907381	3.251785	-1.606727
H	-1.525559	2.313211	1.105939	H	-1.417792	3.532269	-2.562076
C	1.094648	2.682557	3.229492	H	-1.332264	3.494399	-0.806509
H	3.102995	1.979151	2.857434	H	-1.031417	-2.587910	-1.891162
H	-0.982377	3.264719	3.293994	H	0.039213	-3.416457	-0.766461
H	1.328126	3.093209	4.208738	H	-1.723831	-3.408794	-0.470494
C	-0.862633	-3.261236	2.193210	Br	1.627502	-0.815319	-2.558961
H	-1.950991	-3.154661	2.191044	N	-3.251086	-1.173845	1.076555
H	-0.551271	-3.569193	3.200577	N	-3.898201	1.141255	-0.396087
H	-0.588591	-4.070205	1.509090	C	-5.437508	-1.151767	2.080267
C	1.360122	-2.154635	1.744894	C	-4.477284	-0.590470	1.202005
H	1.725491	-2.602001	2.678566	C	-4.799505	0.593172	0.468117
H	1.890142	-1.208514	1.593426	C	-6.071293	1.195473	0.634543
H	1.628802	-2.831196	0.925089	C	-6.986423	0.629727	1.495640
C	-0.459727	-0.904553	2.947209	C	-6.668731	-0.548921	2.220215
H	-1.530226	-0.692003	3.030747	H	-5.172711	-2.050824	2.629004

H	-6.294565	2.095255	0.068781	O	2.466864	4.361382	-0.069195
H	-7.963696	1.086005	1.624762	O	0.871721	4.990364	-1.596490
H	-7.407692	-0.974819	2.892868	C	-0.364646	5.857322	0.277785
C	2.459304	3.120728	-5.854270	H	-0.451709	6.655009	1.023143
H	2.104175	2.118911	-6.128788	H	-1.255031	5.884743	-0.358915
H	1.630999	3.824077	-6.010726	H	-0.343518	4.896120	0.797411
H	3.265973	3.393385	-6.547216	C	0.810234	7.387816	-1.314431
C	3.410040	4.573434	-4.038362	H	-0.148229	7.472540	-1.838215
H	3.826488	4.610328	-3.025141	H	0.881151	8.215552	-0.599867
H	2.576400	5.284645	-4.083339	H	1.607943	7.494620	-2.053416
H	4.189617	4.916826	-4.730955	C	2.164451	5.996895	1.687312
C	2.946634	3.151663	-4.395048	H	3.136180	5.761161	2.134852
C	4.118418	2.167839	-4.233413	H	1.930031	7.043269	1.913571
H	3.800547	1.139606	-4.445125	H	1.416459	5.355633	2.157508
H	4.937364	2.421922	-4.919077	C	3.429185	6.526842	-0.408706
H	4.521436	2.185500	-3.214248	H	3.364957	7.602591	-0.214243
C	1.746804	2.712344	-3.492056	H	4.346927	6.146954	0.052299
C	2.014221	2.730441	-2.016165	H	3.505464	6.371040	-1.489096
C	0.880801	6.043894	-0.593877	H	0.896497	3.367285	-3.719028
C	2.230299	5.773247	0.179606	H	1.480651	1.700982	-3.808865
B	1.755498	4.004443	-1.195939	H	2.800556	2.058618	-1.673792

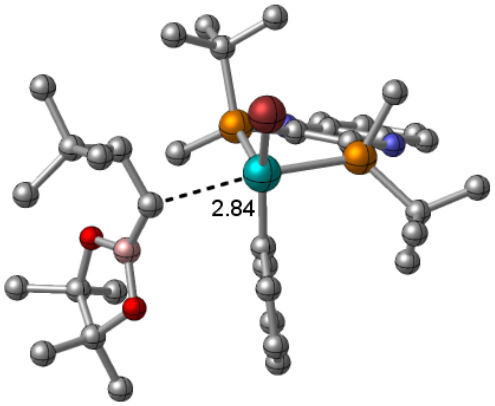

Zero-point correction= 0.874424 (Hartree/Particle)
Thermal correction to Energy= 0.926096
Thermal correction to Enthalpy= 0.927040
Thermal correction to Gibbs Free Energy= 0.790843
Sum of electronic and zero-point Energies= -6208.473530
Sum of electronic and thermal Energies= -6208.421858
Sum of electronic and thermal Enthalpies= -6208.420914
Sum of electronic and thermal Free Energies= -6208.557110

$^{4}TS_{Q}{}^{R}{}_{III\text{-}IV}$

E(scf) = -6209.37209908 a.u.

ν_{min} = -32.64 cm^{-1}

C	-2.639896	-0.810284	0.217630
C	-2.905073	0.392261	-0.533644
P	-0.912006	-1.489377	0.252095
P	-1.591545	1.124911	-1.631646
Fe	0.541335	0.252828	-0.762183
C	-1.063599	-3.052515	-0.713224
C	-0.583895	-2.076849	2.021110
C	-2.183867	0.657201	-3.373402
C	-1.899593	2.926093	-1.440503
C	1.345142	1.246849	0.826252
C	2.671564	1.060414	1.270173
C	0.531379	2.063320	1.639613
C	3.153490	1.637382	2.449638
H	3.348991	0.441588	0.681325
C	0.996227	2.652743	2.821883
H	-0.507028	2.236416	1.354849
C	2.314637	2.438000	3.232047
H	4.182774	1.466454	2.760132
H	0.332677	3.272306	3.422848
H	2.685593	2.890438	4.148890
C	-1.332704	-3.369312	2.387476
H	-2.415098	-3.248218	2.292890
H	-1.105772	-3.627595	3.430752
H	-1.020060	-4.215873	1.767049
C	0.939263	-2.321688	2.069915
H	1.209302	-2.731482	3.051969
H	1.499400	-1.392755	1.922875
H	1.265123	-3.040899	1.308511
C	-0.957382	-0.958242	3.008246
H	-2.038498	-0.786698	3.036902
H	-0.458768	-0.015814	2.763042
H	-0.638554	-1.248850	4.017902
C	-2.147641	-0.876655	-3.479017
H	-2.833573	-1.353876	-2.769851
H	-1.136312	-1.260374	-3.308720
H	-2.453418	-1.177695	-4.489325
C	-1.177273	1.258804	-4.368722
H	-1.144903	2.351738	-4.308892
H	-1.477965	0.991430	-5.390187
H	-0.169120	0.870385	-4.204163
C	-3.595246	1.177361	-3.685206
H	-3.869737	0.864591	-4.701507
H	-3.646245	2.270863	-3.653220
H	-4.346846	0.780316	-2.997159
H	-2.922185	3.205520	-1.705609
H	-1.178244	3.489231	-2.037735
H	-1.725443	3.180424	-0.391849
H	-1.143809	-2.822979	-1.776073
H	-0.151880	-3.638758	-0.566111
H	-1.933489	-3.636979	-0.399308
Br	1.734641	-1.113923	-2.342850
N	-3.583679	-1.392786	0.935314
N	-4.081085	0.991075	-0.478519
C	-5.850703	-1.397450	1.739068
C	-4.810766	-0.804967	0.978678
C	-5.055656	0.419692	0.281221
C	-6.331094	1.032943	0.369997
C	-7.322614	0.437058	1.117483
C	-7.082052	-0.783776	1.802882
H	-5.644424	-2.327419	2.260659
H	-6.495026	1.963474	-0.165389
H	-8.302606	0.900335	1.186553
H	-7.882078	-1.232685	2.384484
C	2.031607	2.664107	-5.675737
H	1.863167	1.580662	-5.715665
H	1.056313	3.159110	-5.766478
H	2.631417	2.943131	-6.551878
C	2.947483	4.591089	-4.349434
H	3.503997	4.904041	-3.457848
H	1.986644	5.120181	-4.344323
H	3.513884	4.921487	-5.229788
C	2.742378	3.066876	-4.372197
C	4.104162	2.356328	-4.294531
H	3.979331	1.266255	-4.280570
H	4.728272	2.614585	-5.159880
H	4.653828	2.642236	-3.390144
C	1.836618	2.621385	-3.174158
C	2.369415	2.962181	-1.821626

C	0.757307	6.096343	-0.498104	H	0.995492	7.596955	-2.062251
C	2.199616	6.165172	0.136701	C	2.231932	6.475243	1.629778
B	1.981093	4.250354	-1.092632	H	3.270220	6.498859	1.977961
O	2.702574	4.815634	-0.063021	H	1.781612	7.453190	1.833791
O	0.891834	5.018829	-1.467289	H	1.702504	5.713232	2.206580
C	-0.323677	5.672316	0.501313	C	3.149065	7.099409	-0.622058
H	-0.502442	6.451244	1.249869	H	2.879465	8.151270	-0.481036
H	-1.259248	5.494405	-0.037800	H	4.167385	6.950660	-0.248330
H	-0.043320	4.748574	1.015226	H	3.147226	6.878306	-1.694592
C	0.318759	7.355826	-1.238939	H	0.855045	3.089425	-3.311163
H	-0.682256	7.205394	-1.657868	H	1.704402	1.537241	-3.260474
H	0.276501	8.212030	-0.556403	H	3.174942	2.340179	-1.436855

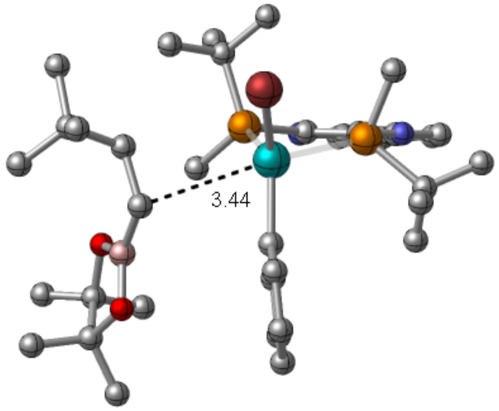

Zero-point correction= 0.872299 (Hartree/Particle)
Thermal correction to Energy= 0.924918
Thermal correction to Enthalpy= 0.925862
Thermal correction to Gibbs Free Energy= 0.784924
Sum of electronic and zero-point Energies= -6208.499800
Sum of electronic and thermal Energies= -6208.447182
Sum of electronic and thermal Enthalpies= -6208.446237
Sum of electronic and thermal Free Energies= -6208.587176

$^{2}TS_{Q}{}^{S}{}_{III\text{-}IV}$

E(scf) = -6209.34759961 a.u.

ν_{min} = -105.65 cm^{-1}

C	-2.731149	-0.722019	0.413031	C	-0.507656	-2.429851	1.571776
C	-3.029852	0.557584	-0.176956	C	-2.566384	1.234600	-3.039336
P	-1.091286	-1.491426	0.027915	C	-2.035867	3.144925	-0.909264
P	-1.785209	1.364324	-1.311483	C	0.535899	1.092717	0.766146
Fe	0.153374	0.324872	-0.990399	C	1.877490	0.820832	1.107405
C	-1.596098	-2.853626	-1.111409	C	-0.227670	1.791341	1.716833

C	2.433913	1.236216	2.319872
H	2.516221	0.262721	0.419984
C	0.318661	2.221992	2.931203
H	-1.281547	1.984904	1.537867
C	1.654245	1.949419	3.235905
H	3.472860	1.007979	2.547603
H	-0.303187	2.760827	3.643172
H	2.081728	2.281338	4.178826
C	-1.316507	-3.712227	1.840069
H	-2.377743	-3.494841	1.988593
H	-0.932782	-4.186666	2.753462
H	-1.218359	-4.442358	1.030551
C	0.959231	-2.796342	1.262832
H	1.368410	-3.391325	2.089799
H	1.580205	-1.902085	1.149601
H	1.051092	-3.392068	0.346735
C	-0.564336	-1.525585	2.814088
H	-1.590081	-1.221971	3.048448
H	0.045373	-0.628886	2.697225
H	-0.176947	-2.083951	3.676684
C	-2.770543	-0.247259	-3.386783
H	-3.461818	-0.737521	-2.691187
H	-1.818801	-0.784264	-3.385471
H	-3.199067	-0.328356	-4.394130
C	-1.548802	1.855929	-4.011613
H	-1.398817	2.925202	-3.829436
H	-1.919103	1.746051	-5.039739
H	-0.575375	1.367448	-3.946365
C	-3.912121	1.973578	-3.133252
H	-4.316129	1.838069	-4.145475
H	-3.807829	3.049993	-2.964976
H	-4.647829	1.585376	-2.422252
H	-3.095206	3.342113	-0.728587
H	-1.685853	3.759016	-1.740924
H	-1.468952	3.418211	-0.019255
H	-1.876729	-2.437155	-2.077577
H	-0.741720	-3.517511	-1.268746
H	-2.436843	-3.422195	-0.703676
Br	0.918371	-0.997080	-2.820990
N	-3.583252	-1.326770	1.219073
N	-4.162169	1.184045	0.077843
C	-5.702067	-1.302805	2.358319
C	-4.759564	-0.694858	1.491435
C	-5.052212	0.580511	0.914490
C	-6.280038	1.220839	1.217331
C	-7.179044	0.606908	2.061644
C	-6.888724	-0.659619	2.634422
H	-5.459930	-2.270646	2.787400
H	-6.481557	2.189037	0.768407
H	-8.121925	1.092746	2.295847
H	-7.614224	-1.121482	3.297907
C	3.088568	4.461060	0.097207
H	2.441938	4.245968	0.957123
H	3.723581	3.583512	-0.068082
H	3.744100	5.299165	0.367690
C	3.189810	5.079418	-2.340826
H	2.617505	5.253081	-3.260405
H	3.863767	4.234122	-2.521767
H	3.809933	5.965553	-2.153089
C	2.255787	4.802085	-1.151040
C	1.405890	6.052127	-0.864204
H	0.710995	5.874830	-0.032991
H	2.039278	6.907298	-0.594670
H	0.812569	6.338276	-1.742363
C	1.274340	3.628102	-1.477595
C	1.931385	2.346418	-1.894628
C	3.628793	1.542080	-5.148493
C	2.219655	2.046378	-5.655032
B	2.291652	2.054585	-3.361816
O	1.624637	2.592975	-4.444614
O	3.409751	1.334860	-3.728306
C	4.096762	0.224222	-5.759125
H	4.208701	0.314746	-6.845528
H	5.070760	-0.049168	-5.338606
H	3.396823	-0.585518	-5.541133
C	4.732215	2.599711	-5.270868
H	5.621138	2.248229	-4.736561
H	5.006335	2.779608	-6.315757
H	4.422978	3.548746	-4.821991
C	1.307694	0.915279	-6.142043
H	0.309855	1.320136	-6.338487
H	1.685547	0.472098	-7.069655
H	1.212877	0.135152	-5.382843
C	2.272491	3.156836	-6.700903

H	2.773992	2.809821	-7.611356	H	0.676790	3.467787	-0.573848
H	1.252989	3.456038	-6.967823	H	2.578158	1.893315	-1.147027
H	2.795740	4.040947	-6.328393	H	0.592205	3.964631	-2.268300

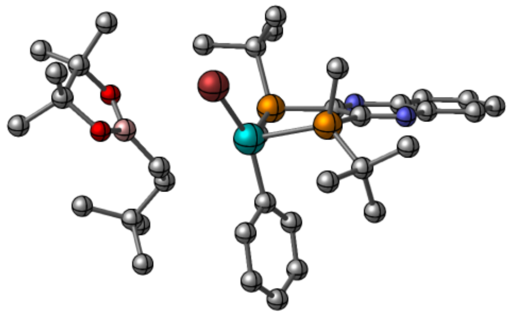

Zero-point correction= 0.874041 (Hartree/Particle)
Thermal correction to Energy= 0.925879
Thermal correction to Enthalpy= 0.926824
Thermal correction to Gibbs Free Energy= 0.789517
Sum of electronic and zero-point Energies= -6208.473559
Sum of electronic and thermal Energies= -6208.421720
Sum of electronic and thermal Enthalpies= -6208.420776
Sum of electronic and thermal Free Energies= -6208.558082

$^{4}TS_{Q}{}^{S}{}_{III\text{-}IV}$

E(scf) = -6209.37470681 a.u.

ν_{min} = -18.43 cm^{-1}

C	-2.934004	-0.671184	0.376438	C	2.945435	2.091159	3.030166
C	-3.145725	0.659791	-0.133599	H	4.589617	0.965183	2.202501
P	-1.266241	-1.458284	0.167833	H	1.121727	3.089441	3.610565
P	-1.810910	1.510559	-1.115065	H	3.530586	2.501471	3.850148
Fe	0.278248	0.287778	-0.665342	C	-1.665476	-3.532675	2.120057
C	-1.637663	-2.849494	-0.979651	H	-2.733315	-3.303212	2.167209
C	-0.829301	-2.281085	1.809701	H	-1.356191	-3.936916	3.093505
C	-2.443508	1.452927	-2.899069	H	-1.511803	-4.320971	1.375119
C	-2.019733	3.259512	-0.582326	C	0.658865	-2.663916	1.664155
C	1.416696	1.020967	0.869152	H	0.997963	-3.162324	2.581517
C	2.780524	0.705886	1.048772	H	1.285859	-1.780911	1.501020
C	0.854523	1.889878	1.829024	H	0.822584	-3.355543	0.828591
C	3.537926	1.228103	2.102725	C	-0.989562	-1.241580	2.932432
H	3.270028	0.037306	0.340191	H	-2.039765	-0.970004	3.086260
C	1.594157	2.420226	2.893407	H	-0.418178	-0.329870	2.726741
H	-0.198021	2.165646	1.748492	H	-0.612533	-1.663127	3.873276

C	-2.536456	-0.025976	-3.313276
H	-3.262363	-0.576190	-2.702970
H	-1.562548	-0.519783	-3.238615
H	-2.865019	-0.089612	-4.358732
C	-1.373782	2.160094	-3.751059
H	-1.317379	3.231351	-3.529224
H	-1.636817	2.060952	-4.812355
H	-0.378396	1.730233	-3.612443
C	-3.804145	2.141288	-3.077549
H	-4.098010	2.078793	-4.134021
H	-3.765978	3.202435	-2.808683
H	-4.588872	1.666076	-2.481317
H	-3.076219	3.536367	-0.542252
H	-1.488576	3.915687	-1.275253
H	-1.581187	3.381093	0.412378
H	-1.815092	-2.452932	-1.981167
H	-0.766365	-3.508754	-1.027112
H	-2.514188	-3.415875	-0.650973
Br	1.175926	-0.921366	-2.538127
N	-3.888644	-1.321858	1.017591
N	-4.285753	1.297215	0.063189
C	-6.127933	-1.348072	1.896050
C	-5.076884	-0.686808	1.211191
C	-5.272402	0.650353	0.741180
C	-6.510837	1.300781	0.974192
C	-7.513962	0.635438	1.643127
C	-7.322087	-0.694483	2.104630
H	-5.960184	-2.363019	2.243999
H	-6.636996	2.316041	0.609924
H	-8.465588	1.127596	1.822183
H	-8.130403	-1.196134	2.628785
C	3.095295	4.672460	-0.053677
H	2.576566	4.233353	0.806889
H	3.913234	3.996531	-0.327309
H	3.541152	5.623083	0.267027
C	2.894863	5.466433	-2.435486
H	2.231340	5.599607	-3.299312
H	3.708300	4.797328	-2.740958
H	3.335620	6.442080	-2.192877
C	2.129543	4.895083	-1.230286
C	1.029522	5.882129	-0.804062
H	0.464176	5.497071	0.054252
H	1.459599	6.849783	-0.514588
H	0.319514	6.063859	-1.621564
C	1.438644	3.547896	-1.623716
C	2.369949	2.472676	-2.076740
C	3.761005	1.475665	-5.420144
C	2.296526	1.920122	-5.799659
B	2.628135	2.120896	-3.543790
O	1.850833	2.591103	-4.587256
O	3.696439	1.361919	-3.973352
C	4.192144	0.129017	-5.992103
H	4.177305	0.148920	-7.087763
H	5.214196	-0.098126	-5.669592
H	3.542058	-0.677294	-5.644756
C	4.815803	2.545162	-5.727722
H	5.764452	2.251225	-5.266503
H	4.976295	2.658903	-6.805011
H	4.525372	3.516137	-5.312868
C	1.347410	0.740282	-6.036322
H	0.325277	1.119649	-6.135997
H	1.603486	0.200191	-6.954116
H	1.366382	0.045765	-5.192533
C	2.204006	2.907785	-6.958622
H	2.600899	2.463391	-7.878390
H	1.155030	3.170874	-7.133807
H	2.752363	3.829923	-6.750683
H	0.883642	3.207139	-0.740472
H	2.980830	2.005695	-1.308455
H	0.707171	3.763147	-2.411364

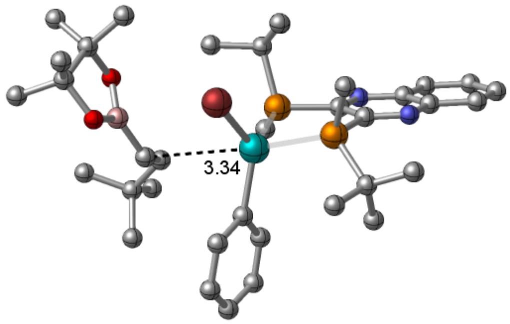

Zero-point correction= 0.871798 (Hartree/Particle)
Thermal correction to Energy= 0.924516
Thermal correction to Enthalpy= 0.925460
Thermal correction to Gibbs Free Energy= 0.783894
Sum of electronic and zero-point Energies= -6208.502909
Sum of electronic and thermal Energies= -6208.450191
Sum of electronic and thermal Enthalpies= -6208.449246
Sum of electronic and thermal Free Energies= -6208.590813

$^{6}TS_{Q}{}^{S}{}_{III\text{-}IV}$

E(scf) = -6209.36207453 a.u.

ν_{min} = -222.86 cm^{-1}

C	-2.860898	-0.641895	0.283492	H	-1.256349	-3.922859	2.929609
C	-3.063523	0.708652	-0.171214	H	-1.439273	-4.302129	1.213420
P	-1.210128	-1.430941	0.006314	C	0.738261	-2.653642	1.482346
P	-1.779844	1.587631	-1.202192	H	1.090103	-3.135863	2.403869
Fe	0.526339	0.473246	-0.833395	H	1.367216	-1.778413	1.293709
C	-1.628929	-2.842656	-1.106585	H	0.887689	-3.361973	0.658664
C	-0.745726	-2.263311	1.644490	C	-0.892134	-1.234627	2.778822
C	-2.471652	1.440285	-2.963282	H	-1.941413	-0.973043	2.954331
C	-2.084455	3.336060	-0.708137	H	-0.332633	-0.316697	2.572971
C	1.483273	1.012288	0.884771	H	-0.495637	-1.662563	3.708868
C	2.788473	0.599265	1.217839	C	-2.434165	-0.045672	-3.356885
C	0.866273	1.921729	1.767692	H	-3.097628	-0.650315	-2.728343
C	3.441689	1.058852	2.366229	H	-1.418902	-0.453317	-3.292351
H	3.312319	-0.096464	0.562143	H	-2.771575	-0.154092	-4.395725
C	1.504818	2.390751	2.921892	C	-1.511184	2.226562	-3.868607
H	-0.143933	2.273543	1.556190	H	-1.517158	3.299699	-3.648358
C	2.799501	1.958596	3.222720	H	-1.822561	2.106167	-4.914771
H	4.450256	0.718991	2.594098	H	-0.484477	1.869037	-3.782606
H	0.997787	3.091703	3.582520	C	-3.899407	1.989028	-3.095937
H	3.304363	2.320678	4.115399	H	-4.220047	1.886505	-4.141338
C	-1.579908	-3.515139	1.962027	H	-3.957716	3.051424	-2.836481
H	-2.646431	-3.284356	2.026816	H	-4.614450	1.444875	-2.471854

H	-3.156319	3.538157	-0.650178	H	0.687177	5.935440	-0.097537
H	-1.615475	4.009782	-1.427203	H	1.938999	7.007864	-0.750545
H	-1.637976	3.513610	0.274582	H	0.669244	6.377010	-1.815709
H	-1.774781	-2.476655	-2.124173	C	1.248841	3.689419	-1.550847
H	-0.786086	-3.539582	-1.119097	C	1.921353	2.408530	-1.963977
H	-2.530765	-3.363972	-0.771431	C	3.609938	1.511537	-5.202403
Br	1.401325	-1.087136	-2.423082	C	2.160917	1.883942	-5.691578
N	-3.810553	-1.299547	0.926519	B	2.303706	2.110131	-3.417360
N	-4.185140	1.357126	0.086249	O	1.609725	2.562582	-4.530354
C	-6.023084	-1.321783	1.871077	O	3.438752	1.400754	-3.767027
C	-4.979678	-0.652539	1.182021	C	4.144670	0.184007	-5.730852
C	-5.161833	0.705962	0.773038	H	4.202092	0.194248	-6.825408
C	-6.378691	1.370711	1.071084	H	5.152988	0.010616	-5.338852
C	-7.374283	0.697665	1.743515	H	3.514059	-0.650623	-5.416330
C	-7.196280	-0.654078	2.143593	C	4.632044	2.629054	-5.443447
H	-5.865727	-2.353377	2.171715	H	5.553434	2.389519	-4.902205
H	-6.495356	2.402194	0.752034	H	4.873908	2.735842	-6.506389
H	-8.309674	1.200258	1.972247	H	4.261524	3.589287	-5.070489
H	-7.998714	-1.161151	2.671582	C	1.281701	0.654979	-5.954075
C	3.119218	4.610071	-0.092532	H	0.248744	0.981744	-6.112113
H	2.534694	4.379170	0.806588	H	1.609760	0.111564	-6.846799
H	3.776185	3.754841	-0.285716	H	1.296141	-0.025360	-5.097780
H	3.756661	5.475769	0.130446	C	2.103716	2.836264	-6.881336
C	3.055405	5.199414	-2.537235	H	2.582913	2.388835	-7.759584
H	2.426767	5.340674	-3.425246	H	1.058617	3.046250	-7.134733
H	3.749214	4.377478	-2.746966	H	2.594547	3.787764	-6.661763
H	3.651597	6.110136	-2.394076	H	0.686419	3.541646	-0.619845
C	2.201778	4.902560	-1.293168	H	2.636912	2.025348	-1.235723
C	1.324466	6.125319	-0.971319	H	0.524081	3.987653	-2.319441

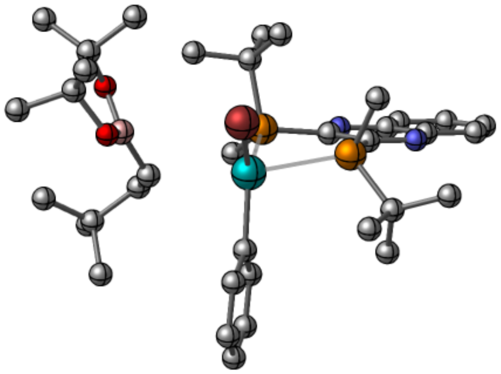

Zero-point correction= 0.872348 (Hartree/Particle)

Thermal correction to Energy= 0.924884
Thermal correction to Enthalpy= 0.925829
Thermal correction to Gibbs Free Energy= 0.784527
Sum of electronic and zero-point Energies= -6208.489726
Sum of electronic and thermal Energies= -6208.437190
Sum of electronic and thermal Enthalpies= -6208.436246
Sum of electronic and thermal Free Energies= -6208.577547

$^{2}IV_{Q}^{R}$
E(scf) = -6209.37523018 a.u.
ν_{min} = 18.68 cm^{-1}

C	-2.470527	-0.638275	0.135768	H	-0.309698	-1.729846	3.791560
C	-2.828089	0.523178	-0.631472	C	-2.006635	-0.417318	-3.697908
P	-0.714302	-1.204849	0.023536	H	-2.775660	-0.905505	-3.088402
P	-1.501338	1.396862	-1.603833	H	-1.040797	-0.892025	-3.511547
Fe	0.483101	0.744893	-0.848613	H	-2.262431	-0.584437	-4.751919
C	-0.933968	-2.657370	-1.096790	C	-0.820093	1.713265	-4.293529
C	-0.236625	-2.047711	1.662809	H	-0.717678	2.790672	-4.124166
C	-1.936239	1.092831	-3.432365	H	-1.070321	1.568080	-5.352517
C	-1.966750	3.158047	-1.370708	H	0.145875	1.235999	-4.105395
C	0.308720	1.469679	0.966351	C	-3.288352	1.737655	-3.792396
C	1.541566	1.433027	1.649856	H	-3.527987	1.479453	-4.832042
C	-0.785444	2.005404	1.666217	H	-3.258754	2.829442	-3.725705
C	1.674227	1.902740	2.958571	H	-4.102593	1.374985	-3.157899
H	2.429468	1.065467	1.141845	H	-3.050735	3.269279	-1.443736
C	-0.661666	2.490924	2.973581	H	-1.480213	3.761397	-2.139154
H	-1.772406	2.037264	1.215398	H	-1.640542	3.509650	-0.392333
C	0.570048	2.441102	3.627494	H	-1.143321	-2.316006	-2.107234
H	2.642991	1.858658	3.452320	H	-0.009961	-3.239353	-1.121171
H	-1.533490	2.898604	3.481259	H	-1.759804	-3.284900	-0.750587
H	0.669648	2.814489	4.643832	Br	1.525637	-0.774590	-2.434715
C	-0.899799	-3.429424	1.827829	N	-3.354883	-1.279389	0.874299
H	-1.991175	-3.356241	1.818047	N	-4.053435	1.008519	-0.625212
H	-0.595518	-3.845956	2.797211	C	-5.613111	-1.438727	1.686253
H	-0.587496	-4.142014	1.059175	C	-4.628434	-0.793041	0.897616
C	1.294452	-2.216382	1.571964	C	-4.983465	0.365558	0.135995
H	1.659965	-2.713994	2.479660	C	-6.314017	0.851863	0.179981
H	1.802248	-1.250916	1.486122	C	-7.251436	0.203421	0.953626
H	1.588956	-2.831220	0.713403	C	-6.899424	-0.945790	1.709665
C	-0.601883	-1.186509	2.883027	H	-5.323236	-2.315256	2.258095
H	-1.679161	-1.000830	2.939254	H	-6.561158	1.732972	-0.404801
H	-0.081208	-0.231506	2.888252	H	-8.272758	0.571184	0.990195

H	-7.657553	-1.437719	2.312221	O	3.800943	1.517478	-0.564765
C	1.746593	6.130070	-0.386512	C	4.888736	-0.609888	-0.694726
H	0.708089	6.206599	-0.038221	H	5.778481	-1.171279	-0.998945
H	2.380083	5.956489	0.493094	H	4.763779	-0.728470	0.387520
H	2.027929	7.101085	-0.815292	H	4.009150	-1.033276	-1.183719
C	3.380561	4.925775	-1.852298	C	6.183082	1.474923	-0.216548
H	3.527299	4.197141	-2.654305	H	6.047124	1.240314	0.844893
H	4.023499	4.634836	-1.011799	H	7.139826	1.048998	-0.538633
H	3.727516	5.901247	-2.218572	H	6.233421	2.562018	-0.315173
C	1.907038	4.995976	-1.417556	C	5.313573	0.007024	-3.472559
C	1.034066	5.318885	-2.643318	H	5.286371	0.323895	-4.521028
H	-0.019883	5.432480	-2.360237	H	6.299084	-0.428850	-3.273704
H	1.352461	6.259540	-3.111901	H	4.544753	-0.756180	-3.334525
H	1.098684	4.534405	-3.406052	C	5.990019	2.361607	-2.950301
C	1.450916	3.674432	-0.730210	H	7.040047	2.081836	-2.812799
C	1.552286	2.366263	-1.541797	H	5.837116	2.612991	-4.005333
C	5.023845	0.883183	-1.017157	H	5.786767	3.259360	-2.360455
C	5.047017	1.214233	-2.573263	H	2.036548	3.564821	0.189917
B	3.014042	1.829398	-1.661186	H	0.421765	3.836417	-0.394054
O	3.699172	1.670153	-2.846200	H	1.177458	2.534660	-2.558500

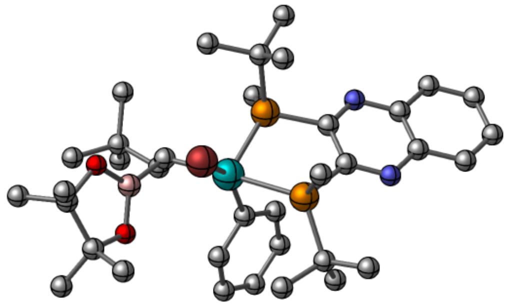

Zero-point correction= 0.877362 (Hartree/Particle)
Thermal correction to Energy= 0.928542
Thermal correction to Enthalpy= 0.929487
Thermal correction to Gibbs Free Energy= 0.795426
Sum of electronic and zero-point Energies= -6208.497869
Sum of electronic and thermal Energies= -6208.446688
Sum of electronic and thermal Enthalpies= -6208.445744
Sum of electronic and thermal Free Energies= -6208.579804

$^4IV_Q^R$

E(scf) = -6209.39452470 a.u.

ν_{min} = 16.7 cm^{-1}

C	-2.285645	-1.107510	-0.217519	P	-0.813483	-0.306024	0.586207
C	-3.009219	-0.426205	-1.250922	P	-2.299727	1.119207	-1.994271

Fe	0.006546	1.429504	-1.074488	H	-3.397864	2.592043	-0.391568
C	0.208141	-1.789972	0.928206	H	0.546522	-2.189454	-0.031040
C	-1.470446	0.232534	2.288014	H	1.077512	-1.482917	1.503100
C	-2.607194	0.935391	-3.868478	H	-0.370738	-2.549015	1.459971
C	-3.547304	2.364161	-1.449349	Br	0.993472	-0.383429	-2.416925
C	-0.056485	3.266038	-1.877315	N	-2.668462	-2.293676	0.220177
C	-0.886339	4.291718	-1.402723	N	-4.141142	-0.908239	-1.731754
C	0.773383	3.562538	-2.973841	C	-4.240169	-4.111620	0.129604
C	-0.918751	5.553646	-2.012134	C	-3.801500	-2.833803	-0.302296
H	-1.524988	4.117789	-0.539011	C	-4.573549	-2.110705	-1.265419
C	0.749183	4.815629	-3.589241	C	-5.779221	-2.670384	-1.760034
H	1.453753	2.801511	-3.351850	C	-6.185048	-3.909829	-1.317973
C	-0.102924	5.818908	-3.112913	C	-5.410943	-4.634688	-0.372163
H	-1.578988	6.325944	-1.621909	H	-3.637166	-4.645280	0.858229
H	1.401307	5.014069	-4.437596	H	-6.351105	-2.105129	-2.489987
H	-0.122014	6.796086	-3.589415	H	-7.104912	-4.346148	-1.696563
C	-2.513618	-0.725778	2.885178	H	-5.753408	-5.611706	-0.043426
H	-3.447849	-0.724354	2.314997	C	1.577103	4.967902	2.652799
H	-2.746441	-0.400839	3.908326	H	0.564921	5.334883	2.436960
H	-2.149352	-1.756509	2.934588	H	1.507608	4.275748	3.502435
C	-0.255438	0.302396	3.236001	H	2.182189	5.827113	2.970497
H	-0.528545	0.872085	4.133423	C	3.603780	3.786604	1.776016
H	0.615458	0.779453	2.779232	H	4.082209	3.290538	0.924739
H	0.050039	-0.699140	3.557817	H	3.568359	3.073118	2.608078
C	-2.105685	1.621417	2.107562	H	4.245827	4.623959	2.079756
H	-2.961006	1.575524	1.423575	C	2.192085	4.277667	1.420517
H	-1.391095	2.353641	1.718396	C	2.273447	5.298980	0.271984
H	-2.474280	1.991942	3.072855	H	1.278339	5.668170	-0.001657
C	-2.191596	-0.474459	-4.328053	H	2.885656	6.161682	0.566277
H	-2.860136	-1.246763	-3.936396	H	2.722462	4.866898	-0.628504
H	-1.168392	-0.707861	-4.023382	C	1.266741	3.083381	1.039356
H	-2.240346	-0.514825	-5.424305	C	1.695413	2.218579	-0.159195
C	-1.713003	1.979394	-4.560904	C	3.953667	-0.484703	1.29632
H	-1.913010	2.996406	-4.207132	C	4.639906	-0.170859	-0.09131
H	-1.907779	1.955172	-5.641375	B	2.731201	1.096093	0.148369
H	-0.653236	1.769047	-4.400064	O	3.925182	1.008795	-0.541209
C	-4.076714	1.192472	-4.249130	O	2.655367	0.164696	1.16849
H	-4.188162	1.031315	-5.329915	C	3.74632	-1.970802	1.575801
H	-4.383057	2.223146	-4.041487	H	4.711922	-2.486479	1.624551
H	-4.756198	0.511499	-3.731308	H	3.245832	-2.102184	2.541608
H	-4.560997	1.980008	-1.592227	H	3.136047	-2.448242	0.806953
H	-3.420159	3.286889	-2.020259	C	4.653862	0.168573	2.492282

H	4.012535	0.070168	3.374812	C	6.125379	0.171738	-0.002747
H	5.613274	-0.313318	2.70778	H	6.69703	-0.675034	0.393741
H	4.829787	1.232737	2.319505	H	6.507639	0.400222	-1.003492
C	4.421325	-1.260557	-1.145386	H	6.305298	1.042726	0.632211
H	4.788587	-0.894433	-2.109812	H	1.157206	2.451405	1.927277
H	4.965356	-2.177463	-0.894192	H	0.275849	3.524148	0.864488
H	3.359905	-1.486648	-1.265892	H	2.167333	2.848488	-0.916124

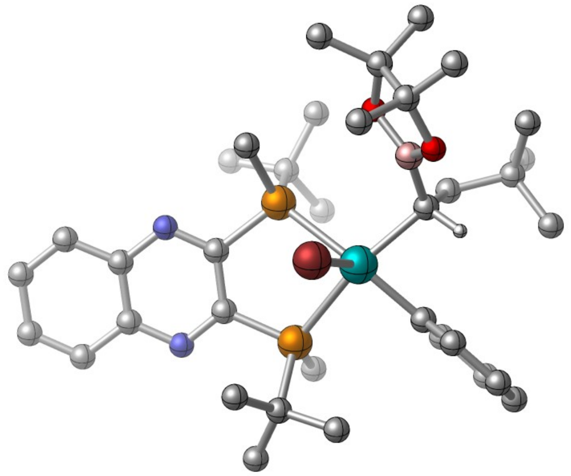

Zero-point correction= 0.876033 (Hartree/Particle)
Thermal correction to Energy= 0.927893
Thermal correction to Enthalpy= 0.928837
Thermal correction to Gibbs Free Energy= 0.791278
Sum of electronic and zero-point Energies= -6208.518491
Sum of electronic and thermal Energies= -6208.466632
Sum of electronic and thermal Enthalpies= -6208.465687
Sum of electronic and thermal Free Energies= -6208.603247

$^{6}IV_{Q}^{R}$

E(scf) = -6209.37404406 a.u.

ν_{min} = 4.92 cm^{-1}

C	-2.796926	-0.641537	0.444626	C	1.374670	1.362017	1.206588
C	-2.912230	0.452936	-0.490512	C	2.455134	0.724881	1.845630
P	-1.130436	-1.409360	0.792903	C	0.734148	2.400632	1.908554
P	-1.496119	1.053898	-1.545563	C	2.868824	1.095265	3.129503
Fe	0.877064	0.898530	-0.708860	H	2.980149	-0.078305	1.332976
C	-1.436007	-3.133148	0.169048	C	1.135331	2.776896	3.195090
C	-1.107052	-1.668970	2.684205	H	-0.096182	2.935000	1.447261
C	-1.762526	0.190258	-3.219596	C	2.206078	2.121333	3.809352
C	-1.957757	2.808761	-1.835624	H	3.705650	0.583195	3.600525

H	0.617118	3.579781	3.715781	H	-6.452555	2.139617	-0.873468
H	2.524284	2.411145	4.808090	H	-8.471240	1.331231	0.351615
C	-2.077125	-2.734710	3.219083	H	-8.307861	-0.607134	1.893437
H	-3.115854	-2.487721	2.988684	C	1.650609	6.241110	-0.941899
H	-1.971342	-2.800454	4.311356	H	0.685472	6.240076	-0.417987
H	-1.859675	-3.728306	2.812243	H	2.441149	6.170552	-0.183854
C	0.342105	-2.104556	2.988354	H	1.755246	7.210371	-1.446919
H	0.468923	-2.231515	4.072169	C	3.125099	5.092877	-2.613081
H	1.069608	-1.361959	2.646895	H	3.193264	4.339807	-3.406208
H	0.587017	-3.063532	2.514782	H	3.917379	4.884833	-1.885673
C	-1.390485	-0.318281	3.363112	H	3.324031	6.071670	-3.069007
H	-2.425317	0.005026	3.202951	C	1.739767	5.076035	-1.945099
H	-0.720801	0.468249	3.001324	C	0.657084	5.263993	-3.021852
H	-1.235713	-0.411133	4.446624	H	-0.342627	5.324269	-2.573706
C	-1.703950	-1.328816	-2.993355	H	0.823939	6.191454	-3.585177
H	-2.526134	-1.677215	-2.358697	H	0.657467	4.437847	-3.742974
H	-0.753398	-1.628665	-2.541810	C	1.512789	3.751385	-1.152549
H	-1.792388	-1.839256	-3.960839	C	1.698172	2.436379	-1.919132
C	-0.613411	0.616720	-4.153413	C	5.041983	1.184337	-3.188723
H	-0.607742	1.697357	-4.332773	C	5.342391	1.468526	-1.659682
H	-0.747410	0.122581	-5.124336	B	3.156737	1.987959	-2.149930
H	0.366988	0.324392	-3.763590	O	4.170976	2.212068	-1.235187
C	-3.112404	0.577488	-3.844521	O	3.600525	1.324160	-3.275798
H	-3.237111	0.021664	-4.783038	C	5.640827	2.231829	-4.134150
H	-3.163486	1.644511	-4.084827	H	6.733367	2.166436	-4.173247
H	-3.956556	0.330548	-3.193520	H	5.249364	2.063445	-5.143099
H	-3.007498	2.899667	-2.119788	H	5.361599	3.245428	-3.830321
H	-1.317515	3.212792	-2.621876	C	5.410597	-0.220242	-3.659522
H	-1.788635	3.378998	-0.917844	H	5.157671	-0.329773	-4.720015
H	-1.357442	-3.152557	-0.919813	H	6.484837	-0.404520	-3.545111
H	-0.656049	-3.789359	0.569103	H	4.857164	-0.979382	-3.101961
H	-2.417205	-3.515630	0.467499	C	6.577150	2.328042	-1.397886
Br	1.872416	-1.162905	-1.410507	H	6.679925	2.499400	-0.320780
N	-3.865544	-1.083882	1.086708	H	7.484485	1.823889	-1.749484
N	-4.061226	1.072080	-0.691805	H	6.506905	3.302290	-1.887730
C	-6.217127	-0.913617	1.569075	C	5.401186	0.203439	-0.796310
C	-5.059195	-0.463994	0.884703	H	6.284620	-0.401809	-1.027032
C	-5.156072	0.647331	-0.007006	H	5.450514	0.498615	0.257270
C	-6.403458	1.296085	-0.191206	H	4.504851	-0.405133	-0.934303
C	-7.511542	0.841247	0.488536	H	2.196477	3.751217	-0.295853
C	-7.417921	-0.268632	1.370564	H	0.502677	3.815426	-0.720770
H	-6.124945	-1.760818	2.242211	H	1.117981	2.405608	-2.848310

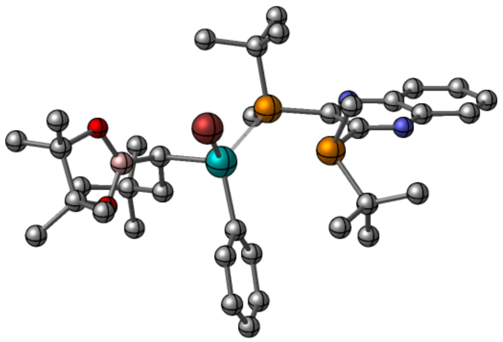

Zero-point correction= 0.873101 (Hartree/Particle)
Thermal correction to Energy= 0.925943
Thermal correction to Enthalpy= 0.926887
Thermal correction to Gibbs Free Energy= 0.783512
Sum of electronic and zero-point Energies= -6208.500943
Sum of electronic and thermal Energies= -6208.448101
Sum of electronic and thermal Enthalpies= -6208.447157
Sum of electronic and thermal Free Energies= -6208.590532

$^{2}\mathbf{IV}_{\mathbf{Q}}^{S}$
E(scf) = -6209.37538655 a.u.
ν_{min} = 24.55 cm^{-1}

C	-2.387585	-0.477011	0.293665	H	-1.915175	-3.138655	2.049397
C	-2.765401	0.637664	-0.529558	H	-0.484247	-3.645153	2.967060
P	-0.672587	-1.132423	0.081003	H	-0.596245	-4.023765	1.248081
P	-1.489766	1.422163	-1.639659	C	1.394159	-2.173663	1.542222
Fe	0.549823	0.720886	-0.999944	H	1.783845	-2.695554	2.425969
C	-1.074971	-2.609257	-0.953909	H	1.946557	-1.235084	1.435702
C	-0.117431	-1.923271	1.722295	H	1.606068	-2.799160	0.666590
C	-2.083716	1.100309	-3.421178	C	-0.355092	-0.986790	2.918595
C	-1.888185	3.194987	-1.412802	H	-1.417698	-0.759080	3.048923
C	0.596693	1.547391	0.794763	H	0.194973	-0.052114	2.828559
C	1.865027	1.464006	1.404691	H	-0.007527	-1.491164	3.830147
C	-0.415877	2.171939	1.542305	C	-2.302080	-0.403397	-3.643149
C	2.112323	1.971203	2.680831	H	-3.076750	-0.803828	-2.978689
H	2.689497	0.994265	0.872201	H	-1.374678	-0.960127	-3.497326
C	-0.178856	2.701191	2.817012	H	-2.632360	-0.569075	-4.676685
H	-1.428027	2.236548	1.157115	C	-0.985173	1.622280	-4.368239
C	1.088949	2.604582	3.392913	H	-0.767893	2.683355	-4.200210
H	3.105996	1.882833	3.114947	H	-1.329949	1.513988	-5.404955
H	-0.991857	3.176797	3.361770	H	-0.058712	1.053585	-4.256567
H	1.276329	3.010280	4.384070	C	-3.403270	1.845160	-3.703883
C	-0.829630	-3.261868	1.997700	H	-3.743282	1.570874	-4.710967

H	-3.279932	2.931987	-3.686602	C	4.194257	3.544385	-3.112938
H	-4.191765	1.574062	-2.995339	H	4.590987	4.010444	-2.205197
H	-2.971057	3.335551	-1.417606	H	4.873284	3.783464	-3.942344
H	-1.416928	3.781227	-2.200867	H	3.226150	4.008693	-3.334672
H	-1.484291	3.534097	-0.464251	C	3.146449	1.689633	-1.716444
H	-1.410426	-2.279281	-1.934973	C	1.693216	2.223555	-1.779639
H	-0.180185	-3.220562	-1.089632	C	0.892352	5.818659	-0.750992
H	-1.866230	-3.200247	-0.484915	C	2.263256	5.540857	-0.012678
Br	1.199593	-0.918611	-2.737585	B	1.596702	3.656610	-1.172444
N	-3.233297	-1.032691	1.139458	O	2.430031	4.111794	-0.168473
N	-3.976132	1.156934	-0.477043	O	0.754422	4.661010	-1.620626
C	-5.431233	-1.060524	2.116055	C	-0.307886	5.842609	0.201368
C	-4.488704	-0.506936	1.213821	H	-0.271965	6.719829	0.855893
C	-4.867943	0.599125	0.388789	H	-1.233622	5.889924	-0.380589
C	-6.181199	1.124578	0.481870	H	-0.338736	4.946049	0.826369
C	-7.077974	0.565083	1.365177	C	0.869149	7.068515	-1.628317
C	-6.701077	-0.530856	2.185870	H	-0.110018	7.156434	-2.112022
H	-5.123335	-1.898117	2.734937	H	1.033654	7.970688	-1.028721
H	-6.447272	1.964369	-0.153226	H	1.628184	7.026416	-2.413145
H	-8.085875	0.962942	1.440051	C	2.269464	5.855885	1.482388
H	-7.426980	-0.952364	2.875256	H	3.250313	5.604250	1.900456
C	5.455312	1.434580	-2.645389	H	2.086764	6.921549	1.660713
H	5.882227	1.857827	-1.726584	H	1.520562	5.271091	2.020710
H	5.404938	0.344927	-2.521190	C	3.463888	6.218641	-0.682671
H	6.154427	1.644322	-3.465883	H	3.440466	7.304147	-0.538436
C	3.530098	1.404089	-4.239842	H	4.385689	5.832157	-0.235457
H	2.538088	1.790018	-4.500517	H	3.496594	6.010525	-1.754629
H	3.439320	0.317486	-4.153923	H	3.180090	0.598223	-1.601367
H	4.206916	1.634042	-5.074134	H	3.631547	2.099836	-0.823784
C	4.061510	2.023356	-2.935633	H	1.335139	2.249070	-2.816008

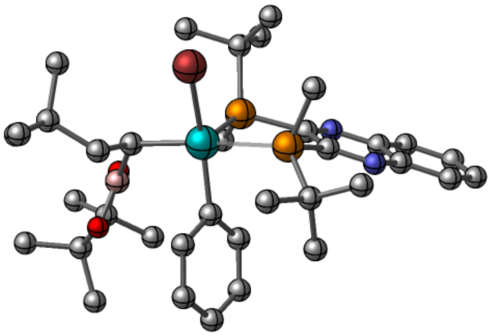

Zero-point correction= 0.878115 (Hartree/Particle)
Thermal correction to Energy= 0.928913

Thermal correction to Enthalpy= 0.929857
Thermal correction to Gibbs Free Energy= 0.797971
Sum of electronic and zero-point Energies= -6208.497271
Sum of electronic and thermal Energies= -6208.446474
Sum of electronic and thermal Enthalpies= -6208.445530
Sum of electronic and thermal Free Energies= -6208.577415

^{4}IV$_Q$^{S}
E(scf) = -6209.39799650 a.u.
ν_{min} = 5.96 cm^{-1}

C	-1.994643	-0.924225	0.083156	C	-2.208073	-0.281519	-3.836122
C	-2.732551	-0.019104	-0.753567	H	-2.952088	-0.915349	-3.344169
P	-0.352080	-0.408935	0.763845	H	-1.212114	-0.673882	-3.616659
P	-1.839482	1.384428	-1.581014	H	-2.374146	-0.354353	-4.918968
Fe	0.551254	1.270996	-0.837001	C	-1.349238	2.040270	-4.246321
C	0.493455	-2.012470	1.040983	H	-1.377175	3.099030	-3.969934
C	-0.836563	0.190195	2.506542	H	-1.625743	1.964811	-5.306428
C	-2.335083	1.195493	-3.418094	H	-0.318698	1.693470	-4.134242
C	-2.765970	2.871061	-0.999268	C	-3.770887	1.684648	-3.681632
C	1.161521	2.706109	-2.061948	H	-4.006501	1.517217	-4.741316
C	0.523752	3.957478	-2.102125	H	-3.884943	2.756602	-3.488878
C	2.224946	2.495598	-2.952921	H	-4.503267	1.140566	-3.080194
C	0.907857	4.950798	-3.008046	H	-3.841721	2.676700	-0.963805
H	-0.286775	4.175285	-1.409669	H	-2.572858	3.703416	-1.681606
C	2.613857	3.480106	-3.867544	H	-2.415601	3.153246	-0.002726
H	2.754993	1.550167	-2.936356	H	0.529129	-2.549199	0.091155
C	1.954232	4.711801	-3.902915	H	1.514943	-1.796164	1.355437
H	0.388114	5.907094	-3.017618	H	-0.027718	-2.618619	1.786100
H	3.439598	3.285941	-4.549749	Br	1.090654	-0.749762	-2.230382
H	2.256145	5.477825	-4.613396	N	-2.507129	-2.077639	0.470121
C	-1.930551	-0.664968	3.170117	N	-4.002797	-0.234349	-1.041869
H	-2.898501	-0.554729	2.672263	C	-4.380783	-3.586538	0.494361
H	-2.053403	-0.334428	4.210306	C	-3.791326	-2.354093	0.114337
H	-1.677118	-1.729577	3.185466	C	-4.571535	-1.389027	-0.598381
C	0.426962	0.130683	3.391386	C	-5.933118	-1.662561	-0.885091
H	0.231334	0.659116	4.333267	C	-6.483046	-2.863065	-0.493776
H	1.306371	0.584630	2.927921	C	-5.702424	-3.830844	0.193563
H	0.683829	-0.904939	3.639041	H	-3.769967	-4.307547	1.029437
C	-1.357125	1.630592	2.358245	H	-6.508793	-0.915305	-1.423304
H	-2.253628	1.652433	1.727127	H	-7.523991	-3.080509	-0.715179
H	-0.613940	2.302989	1.919137	H	-6.160455	-4.771210	0.486538
H	-1.633425	2.031432	3.342055	C	3.239076	5.549019	1.672004

H	4.011039	5.633778	0.896533	C	4.240813	-2.181758	0.038803
H	2.376806	6.147884	1.350344	H	5.182444	-2.640799	-0.282347
H	3.635296	6.000531	2.591108	H	3.834080	-2.783510	0.859106
C	1.798639	4.017491	3.030827	H	3.525554	-2.209438	-0.786671
H	1.495881	2.988028	3.252110	C	5.248036	-0.743058	1.833276
H	0.895263	4.583768	2.767559	H	4.691887	-1.321153	2.579070
H	2.202127	4.446563	3.957377	H	6.239184	-1.193965	1.717338
C	2.840948	4.078277	1.898394	H	5.368426	0.273994	2.219057
C	4.089738	3.281612	2.314645	C	4.881034	-0.272340	-2.021312
H	4.826297	3.249890	1.504416	H	5.179467	0.523377	-2.712369
H	4.564839	3.735501	3.194351	H	5.509597	-1.148142	-2.215870
H	3.836748	2.247458	2.575851	H	3.839562	-0.528443	-2.226892
C	2.232944	3.545608	0.566661	C	6.504283	0.649728	-0.358312
C	1.906141	2.049686	0.528085	H	7.172217	-0.217335	-0.410535
C	4.463983	-0.748534	0.517282	H	6.804252	1.356889	-1.139140
C	5.052341	0.232442	-0.585373	H	6.641885	1.139255	0.608888
B	3.102598	1.097811	0.284209	H	1.330642	4.143309	0.379566
O	4.220631	1.416700	-0.452939	H	2.920237	3.785257	-0.252745
O	3.164970	-0.174926	0.816726	H	1.410528	1.771427	1.458910

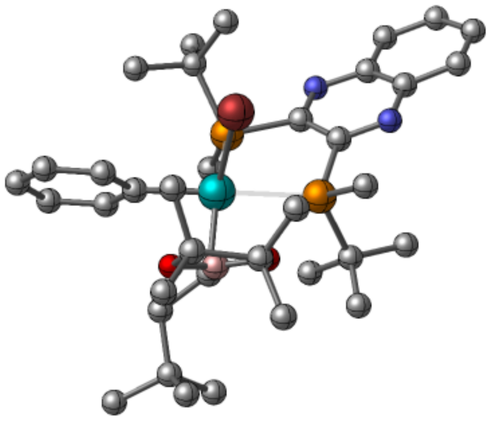

Zero-point correction= 0.875400 (Hartree/Particle)
Thermal correction to Energy= 0.927320
Thermal correction to Enthalpy= 0.928264
Thermal correction to Gibbs Free Energy= 0.790184
Sum of electronic and zero-point Energies= -6208.522597
Sum of electronic and thermal Energies= -6208.470676
Sum of electronic and thermal Enthalpies= -6208.469732
Sum of electronic and thermal Free Energies= -6208.607813

$^{6}IV_{Q}^{S}$

E(scf) = -6209.37530941 a.u.

ν_{min} = 11.59 cm^{-1}

C	-2.841162	-0.846644	0.214410	C	-3.241772	1.616378	-3.646855
C	-3.011400	0.399209	-0.489498	H	-3.421306	1.393735	-4.707170
P	-1.139975	-1.573329	0.353529	H	-3.169730	2.704791	-3.546849
P	-1.566517	1.183926	-1.365814	H	-4.112921	1.277997	-3.077944
Fe	0.456715	0.120640	-0.471576	H	-2.880673	3.261634	-1.281657
C	-1.291842	-3.124404	-0.625022	H	-1.134461	3.572586	-1.575486
C	-0.916775	-2.127577	2.143185	H	-1.694099	3.135625	0.038035
C	-1.950524	0.914724	-3.201442	H	-1.357601	-2.874568	-1.686329
C	-1.859405	2.964798	-1.029565	H	-0.390721	-3.726142	-0.475051
C	1.443662	1.167790	0.982110	H	-2.175606	-3.699161	-0.332337
C	2.827665	1.024792	1.222454	Br	1.645339	-1.161824	-2.120014
C	0.760929	2.060861	1.836005	N	-3.860268	-1.456153	0.793571
C	3.490707	1.729450	2.232380	N	-4.184792	1.004172	-0.539420
H	3.409584	0.351064	0.594807	C	-6.201095	-1.473424	1.345832
C	1.404833	2.769606	2.857897	C	-5.080602	-0.856098	0.733969
H	-0.311240	2.211987	1.705359	C	-5.241521	0.402121	0.071859
C	2.778824	2.607762	3.055542	C	-6.517616	1.020047	0.044175
H	4.561134	1.596587	2.379100	C	-7.588331	0.399162	0.648093
H	0.839439	3.447365	3.495203	C	-7.429712	-0.852967	1.300302
H	3.288955	3.158475	3.842637	H	-6.057541	-2.428283	1.842858
C	-1.755580	-3.358329	2.520928	H	-6.617661	1.975406	-0.462375
H	-2.824769	-3.174143	2.386146	H	-8.568483	0.866777	0.628147
H	-1.577733	-3.603155	3.576829	H	-8.291062	-1.320797	1.768284
H	-1.477325	-4.237590	1.929635	C	5.531090	1.566265	-3.287585
C	0.586940	-2.453964	2.264856	H	6.184394	2.014194	-2.527668
H	0.801649	-2.806764	3.281860	H	5.394307	0.507972	-3.031352
H	1.205664	-1.570581	2.071331	H	6.055800	1.613471	-4.250821
H	0.891807	-3.243181	1.566766	C	3.294582	1.624297	-4.423968
C	-1.261058	-0.942104	3.061271	H	2.347334	2.160849	-4.555060
H	-2.326812	-0.691557	3.020308	H	3.055405	0.593922	-4.136770
H	-0.680267	-0.048910	2.805001	H	3.803199	1.605532	-5.396849
H	-1.019504	-1.205353	4.099181	C	4.179656	2.296697	-3.359331
C	-2.056921	-0.602392	-3.438063	C	4.411986	3.772140	-3.726491
H	-2.895235	-1.046103	-2.888617	H	5.022163	4.278258	-2.967622
H	-1.133180	-1.115143	-3.150418	H	4.930021	3.859771	-4.690491
H	-2.222932	-0.791580	-4.506474	H	3.461856	4.314525	-3.805803
C	-0.747783	1.473210	-3.986198	C	3.501889	2.194452	-1.951975
H	-0.599266	2.543556	-3.802105	C	2.162373	2.846916	-1.864228
H	-0.927148	1.343881	-5.061661	C	0.851055	6.088886	-0.466644
H	0.175250	0.941497	-3.734087	C	2.394910	6.177109	-0.153883

B	1.923062	4.201691	-1.204700	C	2.738139	6.561819	1.281486
O	2.845520	4.814603	-0.383197	H	3.826287	6.594852	1.403005
O	0.784088	4.965582	-1.392101	H	2.337624	7.552084	1.525711
C	0.004929	5.712122	0.753480	H	2.343159	5.833897	1.994255
H	-0.022813	6.524356	1.487352	C	3.162679	7.062141	-1.142138
H	-1.020236	5.508436	0.428044	H	2.928939	8.122657	-1.001741
H	0.393714	4.812602	1.238321	H	4.236796	6.920519	-0.983961
C	0.262781	7.316790	-1.153869	H	2.935626	6.787154	-2.177370
H	-0.803635	7.155298	-1.345914	H	3.404246	1.126407	-1.719106
H	0.362686	8.201617	-0.515297	H	4.182236	2.636114	-1.213212
H	0.750835	7.518797	-2.110404	H	1.341348	2.327021	-2.350672

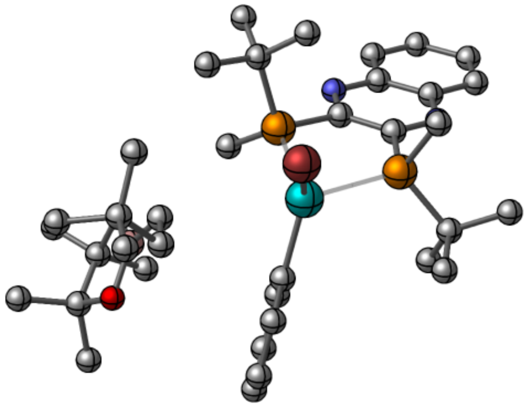

Zero-point correction= 0.871869 (Hartree/Particle)
Thermal correction to Energy= 0.925439
Thermal correction to Enthalpy= 0.926383
Thermal correction to Gibbs Free Energy= 0.781887
Sum of electronic and zero-point Energies= -6208.503441
Sum of electronic and thermal Energies= -6208.449871
Sum of electronic and thermal Enthalpies= -6208.448926
Sum of electronic and thermal Free Energies= -6208.593423

$^{2}TS_{Q}{}^{R}{}_{IV\text{-}I}$
E(scf) = -6209.33905497 a.u.
ν_{min} = -270.06 cm^{-1}

C	-2.857056	-0.542997	0.402162	C	-1.987609	3.143527	-1.318961
C	-3.034983	0.635465	-0.399851	C	1.199825	1.698092	1.074277
P	-1.116983	-1.112827	0.709229	C	2.372060	1.310439	1.765674
P	-1.536635	1.365116	-1.210889	C	0.299067	2.556400	1.746466
Fe	0.332356	0.537064	-0.131040	C	2.591429	1.711537	3.081077
C	-1.113844	-2.812087	0.002020	H	3.097762	0.687679	1.259991
C	-1.023516	-1.410884	2.581859	C	0.519124	2.942089	3.073142
C	-1.676169	0.732022	-3.008004	H	-0.577805	2.939003	1.234551

C	1.664440	2.522892	3.750699
H	3.494619	1.384266	3.592428
H	-0.206502	3.583655	3.569079
H	1.845027	2.835857	4.775764
C	-1.821697	-2.647009	3.031786
H	-2.878163	-2.570146	2.762414
H	-1.751206	-2.729479	4.124916
H	-1.419678	-3.573377	2.608750
C	0.468003	-1.613876	2.907636
H	0.579582	-1.792600	3.985288
H	1.064167	-0.737089	2.642675
H	0.887732	-2.476898	2.378453
C	-1.554568	-0.161865	3.303180
H	-2.631203	-0.031089	3.147965
H	-1.040594	0.745174	2.975349
H	-1.383844	-0.267334	4.382416
C	-1.613307	-0.803832	-2.980543
H	-2.445784	-1.244275	-2.420386
H	-0.669905	-1.151354	-2.545537
H	-1.665634	-1.186496	-4.007861
C	-0.464876	1.259252	-3.799812
H	-0.411529	2.352837	-3.800880
H	-0.557389	0.934100	-4.844411
H	0.481645	0.865363	-3.415194
C	-2.977673	1.198760	-3.678301
H	-3.035483	0.761965	-4.684115
H	-3.012686	2.287414	-3.791677
H	-3.867230	0.883752	-3.124452
H	-1.913373	3.597487	-0.327181
H	-3.009946	3.269517	-1.683108
H	-1.293419	3.655954	-1.988556
H	-1.012420	-2.764622	-1.082373
H	-0.252533	-3.354875	0.397803
H	-2.036487	-3.338932	0.261929
Br	1.903596	-1.224431	-0.789639
N	-3.884505	-1.184004	0.926134
N	-4.225598	1.171492	-0.593146
C	-6.260947	-1.304133	1.277930
C	-5.125981	-0.658896	0.725320
C	-5.295835	0.549919	-0.022779
C	-6.595133	1.092078	-0.189140
C	-7.679698	0.446706	0.362831
C	-7.512203	-0.757013	1.097575
H	-6.110541	-2.220646	1.840762
H	-6.701586	2.010623	-0.758606
H	-8.677303	0.857439	0.236514
H	-8.384278	-1.246371	1.521651
C	1.705332	6.177508	-1.224783
H	0.610582	6.254305	-1.194379
H	2.084064	6.406641	-0.220150
H	2.076452	6.953077	-1.907307
C	3.690149	4.767476	-1.779761
H	4.062128	3.808497	-2.143227
H	4.153084	4.956164	-0.803318
H	4.038010	5.542027	-2.475917
C	2.154309	4.775371	-1.680816
C	1.562184	4.486443	-3.070680
H	0.465498	4.483761	-3.044877
H	1.871157	5.257865	-3.788389
H	1.898231	3.517792	-3.454504
C	1.651966	3.762009	-0.603569
C	1.649105	2.263526	-0.964337
C	4.565974	0.831554	-2.934714
C	5.223314	0.979855	-1.483893
B	3.016388	1.658860	-1.427061
O	4.169992	1.584117	-0.686628
O	3.200903	1.282265	-2.740266
C	5.185955	1.730066	-4.009691
H	6.219478	1.438143	-4.224786
H	4.604386	1.631026	-4.932597
H	5.174373	2.784389	-3.722757
C	4.497872	-0.605213	-3.458591
H	3.982221	-0.607022	-4.425502
H	5.498930	-1.025552	-3.604762
H	3.928408	-1.239082	-2.777705
C	6.432646	1.915670	-1.423387
H	6.757037	2.013549	-0.381836
H	7.270200	1.514655	-2.004488
H	6.197444	2.916194	-1.793479
C	5.577477	-0.349446	-0.809911
H	6.362066	-0.882132	-1.358004
H	5.945799	-0.146217	0.201967
H	4.694627	-0.984947	-0.723881
H	2.244631	3.937364	0.301763

H	0.624740	4.051016	-0.351054	H	0.972113	2.164192	-1.812765

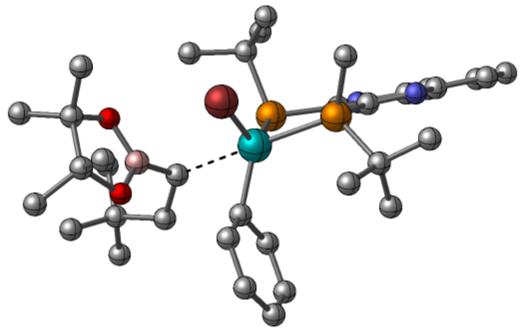

Zero-point correction= 0.875162 (Hartree/Particle)
Thermal correction to Energy= 0.926481
Thermal correction to Enthalpy= 0.927425
Thermal correction to Gibbs Free Energy= 0.792578
Sum of electronic and zero-point Energies= -6208.463893
Sum of electronic and thermal Energies= -6208.412574
Sum of electronic and thermal Enthalpies= -6208.411630
Sum of electronic and thermal Free Energies= -6208.546477

$^{4}TS_{Q}{}^{R}{}_{IV\text{-}I}$

E(scf) = -6209.37429416 a.u.

ν_{min} = -325.6 cm^{-1}

C	-2.563112	-0.069769	0.409867	C	-2.097134	-2.268886	3.181765
C	-2.571704	0.884874	-0.669547	H	-3.101537	-2.063218	2.805649
P	-0.949959	-0.822270	0.958751	H	-2.148525	-2.354061	4.275949
P	-0.979551	1.358629	-1.508753	H	-1.770052	-3.240546	2.795551
Fe	0.884412	0.441017	-0.175555	C	0.308723	-1.559790	3.273170
C	-1.119007	-2.489474	0.183106	H	0.275407	-1.865324	4.327517
C	-1.108103	-1.148595	2.818031	H	1.013148	-0.730237	3.189869
C	-1.181738	0.815687	-3.312758	H	0.693954	-2.410581	2.697271
C	-1.176212	3.187966	-1.545774	C	-1.513643	0.168564	3.502882
C	1.951901	2.112552	-0.075194	H	-2.544969	0.445722	3.261662
C	2.799293	2.339455	-1.190447	H	-0.844714	0.988276	3.223273
C	1.391898	3.257997	0.547618	H	-1.447255	0.045900	4.592402
C	3.028462	3.626334	-1.674363	C	-1.472792	-0.695183	-3.329735
H	3.251324	1.494306	-1.699499	H	-2.432262	-0.930396	-2.853445
C	1.633900	4.541962	0.062629	H	-0.676756	-1.254597	-2.829867
H	0.749901	3.130113	1.414424	H	-1.524267	-1.043289	-4.369876
C	2.453285	4.742489	-1.053399	C	0.182014	1.093712	-3.977567
H	3.666834	3.758970	-2.545840	H	0.456628	2.153660	-3.919269
H	1.173169	5.393513	0.559683	H	0.129021	0.821241	-5.040224
H	2.647099	5.744691	-1.425939	H	0.976372	0.502215	-3.512185

C	-2.293380	1.572354	-4.055997
H	-2.371796	1.175826	-5.077433
H	-2.077268	2.643127	-4.136385
H	-3.266029	1.454701	-3.569305
H	-2.150485	3.469932	-1.952212
H	-0.374581	3.630538	-2.143107
H	-1.088759	3.571430	-0.526808
H	-1.104437	-2.376323	-0.902299
H	-0.263596	-3.109208	0.465376
H	-2.052865	-2.972912	0.484685
Br	1.790443	-1.405178	-1.511895
N	-3.680077	-0.416717	1.023993
N	-3.691446	1.464108	-1.065628
C	-6.061881	-0.178529	1.262678
C	-4.843616	0.163722	0.622402
C	-4.849357	1.122618	-0.437690
C	-6.072019	1.721464	-0.834769
C	-7.240986	1.370246	-0.196795
C	-7.235660	0.416250	0.855807
H	-6.036654	-0.908449	2.066527
H	-6.052964	2.448183	-1.641690
H	-8.180814	1.824625	-0.497053
H	-8.171637	0.157699	1.342740
C	6.186135	-0.933427	0.866493
H	6.151433	-1.096062	-0.218528
H	6.758704	-0.015098	1.050484
H	6.740856	-1.769279	1.312452
C	4.873484	-0.584027	2.969723
H	3.886861	-0.600388	3.448187
H	5.332442	0.385226	3.187052
H	5.477607	-1.369094	3.443336

C	4.765888	-0.832301	1.455172
C	4.041364	-2.170941	1.219692
H	3.993645	-2.414637	0.154553
H	4.563903	-2.983738	1.741978
H	3.008496	-2.149748	1.586438
C	4.045377	0.335464	0.711601
C	2.649990	0.691739	1.219345
C	1.874704	2.352056	4.532578
C	3.199952	3.101004	4.10789
B	2.598961	1.577108	2.496255
O	3.651704	2.327279	2.959949
O	1.481632	1.677879	3.302007
C	2.098487	1.252239	5.57556
H	2.358961	1.671721	6.552635
H	1.176251	0.672387	5.683466
H	2.893793	0.567174	5.266191
C	0.732682	3.262888	4.970645
H	-0.144991	2.658043	5.222131
H	1.016128	3.836994	5.859669
H	0.447159	3.962031	4.180869
C	4.307401	3.083887	5.157902
H	5.19094	3.598063	4.764811
H	3.987115	3.604883	6.06705
H	4.600252	2.06524	5.422625
C	2.969016	4.533361	3.6192
H	2.685118	5.195632	4.443733
H	3.896718	4.909877	3.176063
H	2.195071	4.572456	2.85006
H	4.68113	1.228228	0.762206
H	3.992998	0.040679	-0.34077
H	2.085098	-0.229514	1.411877

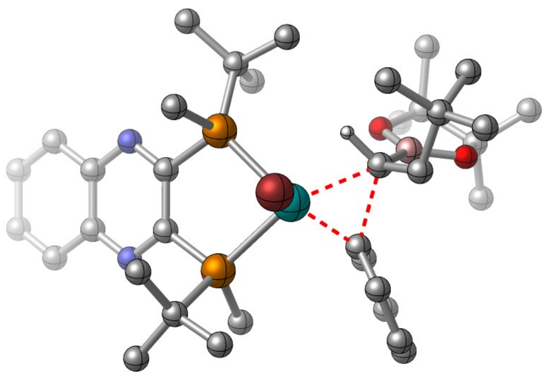

Zero-point correction= 0.873953 (Hartree/Particle)
Thermal correction to Energy= 0.925514
Thermal correction to Enthalpy= 0.926458
Thermal correction to Gibbs Free Energy= 0.789832
Sum of electronic and zero-point Energies= -6208.500341
Sum of electronic and thermal Energies= -6208.448780
Sum of electronic and thermal Enthalpies= -6208.447836
Sum of electronic and thermal Free Energies= -6208.584462

$^{4}TS_{Q}{}^{R}{}_{IV\text{-}I}C1$
E(scf) = -6209.36598206 a.u.
ν_{min} = -275.8 cm^{-1}

C	-2.494303	-0.626652	0.127696	H	-0.222611	0.238151	2.616329
C	-2.879662	0.494498	-0.698967	H	-0.329819	-0.998944	3.874116
P	-0.720201	-1.193848	0.118203	C	-2.225000	-0.954353	-3.563495
P	-1.662554	1.228335	-1.904489	H	-2.856278	-1.386739	-2.779121
Fe	0.544915	0.639841	-1.038444	H	-1.196614	-1.301105	-3.422934
C	-0.813096	-2.771368	-0.838973	H	-2.578346	-1.344392	-4.526800
C	-0.338743	-1.815529	1.868989	C	-1.306957	1.091106	-4.658345
C	-2.283425	0.579813	-3.583325	H	-1.239477	2.183578	-4.684500
C	-2.152361	3.010971	-1.926889	H	-1.654224	0.759135	-5.645593
C	1.248927	1.788214	0.399244	H	-0.302028	0.686642	-4.505118
C	2.378291	1.360171	1.147853	C	-3.711234	1.052913	-3.897302
C	0.234705	2.493114	1.105944	H	-4.015533	0.647627	-4.871714
C	2.471840	1.597778	2.514812	H	-3.779714	2.144055	-3.958991
H	3.179781	0.833001	0.636471	H	-4.432303	0.709218	-3.149842
C	0.338912	2.723979	2.477721	H	-3.207876	3.131429	-1.669047
H	-0.623399	2.882711	0.570054	H	-1.977894	3.421357	-2.925213
C	1.457837	2.286845	3.195052	H	-1.529073	3.575086	-1.228364
H	3.345549	1.244111	3.058134	H	-0.853851	-2.563142	-1.905751
H	-0.462137	3.253320	2.989414	H	0.101207	-3.340270	-0.650690
H	1.540111	2.479577	4.261024	H	-1.683048	-3.366539	-0.545240
C	-1.056247	-3.121390	2.250743	Br	1.891293	-0.907325	-2.411663
H	-2.142375	-3.011901	2.205411	N	-3.363876	-1.207444	0.934489
H	-0.779717	-3.388946	3.280124	N	-4.093693	1.008211	-0.635084
H	-0.761773	-3.957866	1.608435	C	-5.586356	-1.299039	1.851544
C	1.186597	-2.047757	1.866481	C	-4.627272	-0.702990	0.994106
H	1.502299	-2.392440	2.860236	C	-4.992691	0.435624	0.211748
H	1.733749	-1.129176	1.635996	C	-6.305287	0.962392	0.310251
H	1.485957	-2.812448	1.140529	C	-7.217309	0.365013	1.152275
C	-0.691390	-0.712387	2.877520	C	-6.856734	-0.771088	1.924529
H	-1.773456	-0.559304	2.946123	H	-5.289798	-2.164053	2.437482

H	-6.560415	1.829551	-0.291871
H	-8.225455	0.761914	1.229911
H	-7.595098	-1.223543	2.580316
C	1.764633	3.129801	-5.196106
H	1.481522	2.109833	-5.482794
H	0.857600	3.748045	-5.206292
H	2.442294	3.518043	-5.967353
C	2.875501	4.590182	-3.493186
H	3.428066	4.644966	-2.549369
H	2.010946	5.259459	-3.420052
H	3.538294	4.973731	-4.279762
C	2.441121	3.150148	-3.811381
C	3.679804	2.237007	-3.856752
H	3.391126	1.190258	-4.002101
H	4.345859	2.535825	-4.676849
H	4.259780	2.291056	-2.928356
C	1.389156	2.609275	-2.786703
C	1.885902	2.535618	-1.320870
C	1.08704	6.053418	-0.10464
C	2.55972	5.835579	0.480217
B	1.87611	3.956376	-0.667634
O	2.88929	4.472819	0.096257
O	0.833084	4.834389	-0.853576
C	-0.007927	6.163586	0.959701
H	0.108173	7.073923	1.556987
H	-0.983636	6.203637	0.463291
H	-0.005596	5.301016	1.62775
C	0.954588	7.221953	-1.083118
H	-0.062406	7.233975	-1.489704
H	1.13179	8.179024	-0.580944
H	1.649256	7.132646	-1.921224
C	2.656335	5.910812	2.004823
H	3.684671	5.68968	2.30992
H	2.399478	6.911261	2.369455
H	2.003667	5.177575	2.48373
C	3.625937	6.739357	-0.144984
H	3.457506	7.790824	0.11019
H	4.609176	6.446916	0.238284
H	3.649777	6.642836	-1.233426
H	0.501281	3.247237	-2.834483
H	1.099838	1.633855	-3.182661
H	2.875988	2.082357	-1.279311

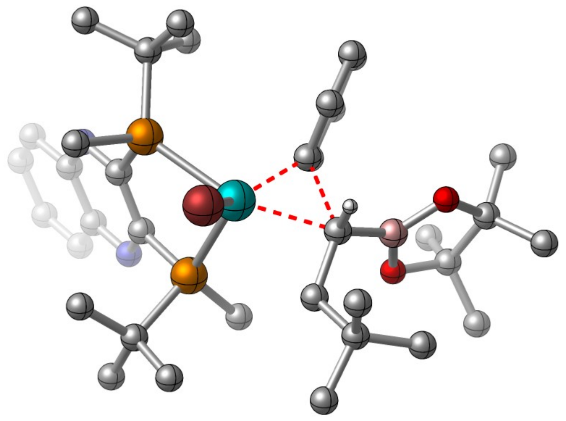

Zero-point correction= 0.874621 (Hartree/Particle)
Thermal correction to Energy= 0.926399
Thermal correction to Enthalpy= 0.927343
Thermal correction to Gibbs Free Energy= 0.790001
Sum of electronic and zero-point Energies= -6208.491361
Sum of electronic and thermal Energies= -6208.439583
Sum of electronic and thermal Enthalpies= -6208.438639
Sum of electronic and thermal Free Energies= -6208.575981

$^{4}TS_{Q}{}^{R}{}_{IV-I}C2$
E(scf) = -6209.36857739 a.u.
ν_{min} = -273.0 cm^{-1}

C	-2.807999	-0.631428	0.455311
C	-2.935620	0.292204	-0.640599
P	-1.112085	-1.060157	1.080415
P	-1.410237	0.952712	-1.476246
Fe	0.540572	0.454589	-0.094938
C	-1.005279	-2.820839	0.539974
C	-1.254129	-1.148013	2.967481
C	-1.457287	0.210379	-3.215168
C	-1.883265	2.721446	-1.692499
C	1.394847	1.656738	1.182633
C	2.615846	1.284883	1.789673
C	0.468560	2.390572	1.959664
C	2.872515	1.595103	3.124507
H	3.358989	0.760913	1.199252
C	0.727409	2.691565	3.298032
H	-0.458852	2.737454	1.506586
C	1.930789	2.295752	3.890451
H	3.816574	1.290907	3.572175
H	-0.011942	3.239854	3.878297
H	2.138623	2.540193	4.928840
C	-1.971380	-2.408223	3.478641
H	-2.987023	-2.485042	3.081367
H	-2.034130	-2.361670	4.574586
H	-1.425114	-3.322284	3.222239
C	0.206263	-1.143628	3.463445
H	0.222617	-1.255406	4.555690
H	0.716494	-0.211607	3.209626
H	0.783547	-1.972424	3.034906
C	-1.985037	0.107816	3.473950
H	-3.051843	0.077516	3.230705
H	-1.558036	1.024999	3.057269
H	-1.890316	0.166953	4.566253
C	-1.394215	-1.320450	-3.066516
H	-2.275473	-1.716721	-2.547596
H	-0.494687	-1.629598	-2.524504
H	-1.360150	-1.781510	-4.062320
C	-0.178120	0.714217	-3.910343
H	-0.189459	1.801783	-4.050289
H	-0.103985	0.257124	-4.906399
H	0.725945	0.458403	-3.351451
C	-2.696967	0.617172	-4.024165
H	-2.642108	0.154512	-5.019029
H	-2.753394	1.701749	-4.168645
H	-3.625427	0.288289	-3.548309
H	-2.928747	2.814798	-1.998019
H	-1.240895	3.192256	-2.440454
H	-1.740893	3.243006	-0.740442
H	-0.919596	-2.849638	-0.548078
H	-0.101997	-3.271041	0.959739
H	-1.888368	-3.386443	0.851347
Br	1.855619	-1.413728	-0.936468
N	-3.870618	-1.160758	1.036064
N	-4.120132	0.682804	-1.077085
C	-6.265830	-1.324299	1.184852
C	-5.098993	-0.783584	0.587074
C	-5.225897	0.164376	-0.476685
C	-6.516484	0.557256	-0.914306
C	-7.632750	0.016604	-0.315697
C	-7.506811	-0.928591	0.737405
H	-6.147850	-2.043014	1.990489
H	-6.589920	1.278806	-1.722716
H	-8.623723	0.311629	-0.648692
H	-8.403577	-1.339877	1.191872
C	2.461793	6.344620	-0.315534
H	1.622832	6.546985	0.362852
H	3.366692	6.234818	0.295823
H	2.594822	7.226212	-0.955772
C	3.401794	4.865645	-2.103496
H	3.213954	4.038104	-2.792899
H	4.315239	4.639595	-1.541319
H	3.587320	5.762661	-2.708704
C	2.206813	5.081550	-1.160889
C	0.934433	5.292719	-2.000975
H	0.048522	5.403659	-1.362419

H	1.020234	6.198478	-2.615045	H	3.396031	-0.374704	-4.693896
H	0.757816	4.452101	-2.681495	H	5.014289	-0.819186	-4.119245
C	2.024762	3.896754	-0.165120	H	3.579381	-1.091453	-3.090141
C	1.709800	2.519137	-0.768671	C	6.298465	1.961478	-1.990213
C	4.189711	0.99069	-3.230824	H	6.779985	2.015821	-1.008015
C	5.055299	1.07551	-1.89007	H	7.021297	1.546976	-2.701346
B	2.909958	1.837289	-1.500046	H	6.049892	2.98014	-2.297279
O	4.155562	1.704672	-0.93777	C	5.444284	-0.282912	-1.300314
O	2.868325	1.433565	-2.814529	H	6.125293	-0.827314	-1.963255
C	4.648649	1.941986	-4.340939	H	5.955908	-0.12076	-0.344964
H	5.624384	1.643013	-4.73861	H	4.558651	-0.891624	-1.110315
H	3.921174	1.907515	-5.158952	H	2.926842	3.824541	0.454973
H	4.716894	2.976288	-3.997208	H	1.213663	4.192122	0.511277
C	4.043028	-0.417239	-3.810497	H	0.844092	2.615098	-1.422476

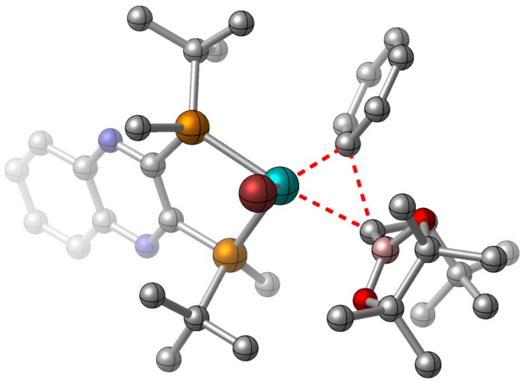

Zero-point correction= 0.873593 (Hartree/Particle)
Thermal correction to Energy= 0.925537
Thermal correction to Enthalpy= 0.926481
Thermal correction to Gibbs Free Energy= 0.788688
Sum of electronic and zero-point Energies= -6208.494984
Sum of electronic and thermal Energies= -6208.443040
Sum of electronic and thermal Enthalpies= -6208.442096
Sum of electronic and thermal Free Energies= -6208.579890

$^{4}TS_{Q}{}^{R}{}_{IV\text{-}I}$C3
E(scf) = -6209.37429465 a.u.
ν_{min} = -288.3 cm^{-1}

C	-2.227385	-1.066282	-0.128207	P	-0.696191	-0.386538	0.687750
C	-2.974534	-0.265886	-1.065944	P	-2.180402	1.214296	-1.863284

Fe	0.159431	1.172604	-1.162878
C	0.264680	-1.923558	0.983775
C	-1.372392	0.056932	2.421094
C	-2.669820	1.090361	-3.695442
C	-3.179043	2.616220	-1.198237
C	0.786353	2.896808	-1.931077
C	-0.006812	4.072220	-1.937708
C	1.639046	2.687226	-3.047893
C	0.000687	4.944371	-3.023043
H	-0.644430	4.295154	-1.087411
C	1.628388	3.558158	-4.134966
H	2.315431	1.839119	-3.042525
C	0.807777	4.692748	-4.140209
H	-0.635963	5.827021	-3.000504
H	2.279189	3.355293	-4.983441
H	0.810080	5.374681	-4.986181
C	-2.308024	-1.004289	3.026894
H	-3.256912	-1.075157	2.488077
H	-2.535790	-0.720054	4.063619
H	-1.854629	-1.999931	3.047765
C	-0.157421	0.216755	3.360190
H	-0.473005	0.725207	4.280519
H	0.659263	0.793515	2.918245
H	0.249050	-0.759718	3.645631
C	-2.152121	1.375870	2.285633
H	-2.999290	1.260882	1.598707
H	-1.526195	2.194527	1.923491
H	-2.557102	1.670925	3.262675
C	-2.403791	-0.342799	-4.192418
H	-3.114190	-1.060707	-3.771843
H	-1.389896	-0.669490	-3.942793
H	-2.512486	-0.366077	-5.284998
C	-1.728712	2.059988	-4.435837
H	-1.845662	3.091980	-4.090484
H	-1.960625	2.040158	-5.509068
H	-0.679223	1.779753	-4.309300
C	-4.130948	1.484233	-3.968282
H	-4.333887	1.372344	-5.042379
H	-4.329872	2.529598	-3.707004
H	-4.830453	0.848121	-3.420416
H	-4.251672	2.405300	-1.238078
H	-2.964897	3.516171	-1.782191
H	-2.887715	2.803937	-0.161278
H	0.639184	-2.266959	0.017289
H	1.116467	-1.675612	1.615949
H	-0.343828	-2.704668	1.445418
Br	0.903808	-0.823684	-2.424376
N	-2.655250	-2.259172	0.240110
N	-4.174913	-0.635267	-1.474421
C	-4.350555	-3.963568	0.143011
C	-3.860807	-2.685117	-0.225823
C	-4.660122	-1.834164	-1.049925
C	-5.945932	-2.265793	-1.463613
C	-6.402519	-3.507992	-1.081265
C	-5.599789	-4.362216	-0.279100
H	-3.724803	-4.596137	0.765670
H	-6.539042	-1.603299	-2.087250
H	-7.384943	-3.845980	-1.398531
H	-5.982400	-5.338494	0.004654
C	0.684573	4.420217	3.056499
H	-0.342316	4.695197	2.782403
H	0.634530	3.520087	3.682468
H	1.094751	5.232684	3.669781
C	2.967118	3.779101	2.243530
H	3.629051	3.624381	1.383636
H	2.948141	2.850240	2.825239
H	3.417167	4.560150	2.869535
C	1.548964	4.180520	1.804605
C	1.604783	5.478723	0.980770
H	0.598914	5.803228	0.687013
H	2.061805	6.287753	1.564628
H	2.195079	5.360038	0.064759
C	0.887184	3.035691	0.989641
C	1.657190	2.553353	-0.247575
C	3.966278	-0.244611	0.916564
C	4.675487	0.267431	-0.402174
B	2.70288	1.398844	-0.062696
O	3.89445	1.444985	-0.748351
O	2.635384	0.355171	0.83315
C	3.827988	-1.76059	1.001193
H	4.815906	-2.232692	0.96262
H	3.358846	-2.043319	1.949322
H	3.217472	-2.153298	0.186381
C	4.59638	0.293935	2.204689

H	3.943071	0.045797	3.047954	C	6.127245	0.701798	-0.213622
H	5.578964	-0.153556	2.38657	H	6.744485	-0.142037	0.114171
H	4.707821	1.380908	2.172769	H	6.525826	1.063168	-1.167603
C	4.571591	-0.701759	-1.581768	H	6.22043	1.50895	0.517144
H	4.948762	-0.201068	-2.480346	H	0.728463	2.186636	1.655075
H	5.172146	-1.602158	-1.414226	H	-0.115954	3.382551	0.706535
H	3.53436	-0.986121	-1.771052	H	2.287099	3.365945	-0.604215

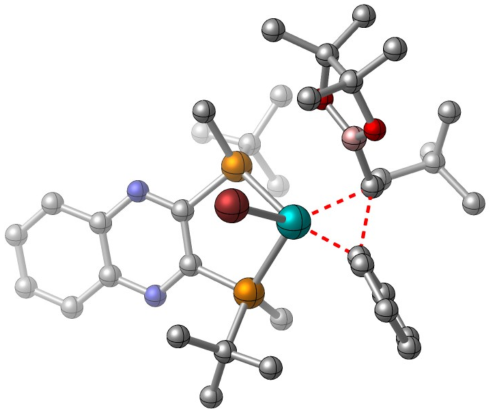

Zero-point correction= 0.874821 (Hartree/Particle)
Thermal correction to Energy= 0.926301
Thermal correction to Enthalpy= 0.927245
Thermal correction to Gibbs Free Energy= 0.791720
Sum of electronic and zero-point Energies= -6208.499473
Sum of electronic and thermal Energies= -6208.447994
Sum of electronic and thermal Enthalpies= -6208.447050
Sum of electronic and thermal Free Energies= -6208.582575

$^{6}TS_{Q}{}^{R}{}_{IV\text{-}I}$

E(scf) = -6209.35341503 a.u.

ν_{min} = -347.84 cm^{-1}

C	-2.827697	-0.724297	0.364984	C	-1.492857	2.376212	-2.032107
C	-2.819483	0.163119	-0.768958	C	1.570173	1.498296	1.049138
P	-1.215553	-1.239470	1.124247	C	2.836170	1.161811	1.655452
P	-1.210293	0.614040	-1.586800	C	0.694938	2.265931	1.901393
Fe	0.600810	0.021594	-0.036002	C	3.195663	1.586650	2.925953
C	-1.209503	-3.050914	0.791714	H	3.538663	0.560482	1.083349
C	-1.421560	-1.078410	2.997201	C	1.048807	2.684656	3.175237
C	-1.259796	-0.340406	-3.223043	H	-0.283438	2.553444	1.513692

C	2.308543	2.352182	3.706891	C	-7.542783	-0.673897	0.358432
H	4.166766	1.309572	3.333982	H	-6.348906	-1.849190	1.719656
H	0.342395	3.256467	3.775581	H	-6.317128	1.397238	-2.085882
H	2.588459	2.673301	4.706828	H	-8.478126	0.607372	-1.121307
C	-2.260773	-2.206536	3.618269	H	-8.493675	-1.008576	0.762820
H	-3.263753	-2.248740	3.183927	C	2.833777	6.621492	0.081146
H	-2.361576	-2.023883	4.696674	H	2.352549	6.727819	1.062121
H	-1.785423	-3.185619	3.495514	H	3.907906	6.472329	0.250477
C	0.018127	-1.121434	3.551893	H	2.707877	7.569696	-0.457969
H	-0.013089	-1.104932	4.649174	C	2.946721	5.329586	-2.065573
H	0.605619	-0.261149	3.215672	H	2.489314	4.554799	-2.692035
H	0.545915	-2.033905	3.247141	H	4.002929	5.068270	-1.931021
C	-2.062155	0.284019	3.312963	H	2.896185	6.276612	-2.619008
H	-3.113421	0.316796	3.008916	C	2.229145	5.446841	-0.709329
H	-1.529982	1.106726	2.825313	C	0.732685	5.722329	-0.945523
H	-2.019353	0.461598	4.395400	H	0.188731	5.791111	0.005922
C	-1.386069	-1.837584	-2.888399	H	0.589124	6.669030	-1.482510
H	-2.330048	-2.068199	-2.380746	H	0.263791	4.931615	-1.542574
H	-0.554099	-2.176287	-2.263000	C	2.415095	4.152237	0.140889
H	-1.359244	-2.418714	-3.819256	C	1.961732	2.866916	-0.520422
C	0.095289	-0.078340	-3.904672	C	3.867560	1.065990	-3.239437
H	0.233069	0.979997	-4.150084	C	4.970055	1.272882	-2.133831
H	0.141732	-0.650004	-4.841502	B	2.971841	2.144573	-1.427869
H	0.930228	-0.380823	-3.269649	O	4.338137	2.224386	-1.235507
C	-2.411215	0.097031	-4.139620	O	2.637669	1.362023	-2.517069
H	-2.383458	-0.506990	-5.056527	C	3.956717	2.086558	-4.379668
H	-2.321971	1.147781	-4.435855	H	4.824499	1.899160	-5.020958
H	-3.389754	-0.044556	-3.671263	H	3.052816	2.011479	-4.993346
H	-2.470221	2.516238	-2.501083	H	4.020615	3.109166	-3.994701
H	-0.701946	2.706063	-2.711566	C	3.790785	-0.349297	-3.802852
H	-1.446588	2.983516	-1.122937	H	3.042075	-0.394467	-4.600931
H	-1.070831	-3.216307	-0.278785	H	4.756848	-0.643249	-4.229367
H	-0.364100	-3.505461	1.315714	H	3.504581	-1.064744	-3.029919
H	-2.144715	-3.517965	1.113830	C	6.277864	1.877338	-2.635804
Br	1.892715	-1.873377	-0.747922	H	6.975095	1.991255	-1.798430
N	-3.959077	-1.152931	0.896668	H	6.746119	1.224105	-3.380782
N	-3.942699	0.632528	-1.282650	H	6.123935	2.863158	-3.081915
C	-6.364245	-1.145507	0.892601	C	5.252845	0.007629	-1.317035
C	-5.126534	-0.704890	0.358993	H	5.811833	-0.730712	-1.902436
C	-5.117661	0.220517	-0.732766	H	5.854370	0.279271	-0.442960
C	-6.346462	0.694901	-1.258058	H	4.326166	-0.456730	-0.970067
C	-7.533895	0.251154	-0.719748	H	3.476843	4.063203	0.404322

H	1.872727	4.310687	1.083062	H	0.937970	2.895622	-0.887278

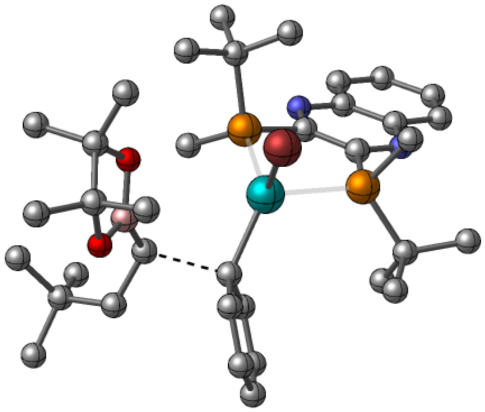

Zero-point correction= 0.871802 (Hartree/Particle)
Thermal correction to Energy= 0.924110
Thermal correction to Enthalpy= 0.925055
Thermal correction to Gibbs Free Energy= 0.785318
Sum of electronic and zero-point Energies= -6208.481613
Sum of electronic and thermal Energies= -6208.429305
Sum of electronic and thermal Enthalpies= -6208.428360
Sum of electronic and thermal Free Energies= -6208.568097

$^{2}TS_{Q}{}^{S}{}_{IV\text{-}I}$

E(scf) = -6209.34573665 a.u.

ν_{min} = -283.36 cm^{-1}

C	-2.662712	-0.663597	0.303844	H	0.536910	3.107214	3.543585
C	-2.953319	0.641553	-0.216508	H	2.465075	1.850499	4.511665
P	-0.923632	-1.285017	0.142885	C	-1.313351	-3.240308	2.221739
P	-1.603373	1.604985	-1.064221	H	-2.377897	-3.002111	2.276159
Fe	0.438532	0.515472	-0.586475	H	-0.985578	-3.589806	3.210338
C	-1.185843	-2.763534	-0.923518	H	-1.180068	-4.071590	1.521170
C	-0.467551	-2.017090	1.829946	C	1.011620	-2.434309	1.725863
C	-2.064254	1.460083	-2.908171	H	1.342989	-2.824533	2.697299
C	-2.121440	3.309350	-0.608043	H	1.650628	-1.588303	1.465189
C	1.491571	1.388702	0.732835	H	1.165361	-3.220872	0.978938
C	2.611516	0.728661	1.290095	C	-0.625256	-0.907140	2.881500
C	0.782180	2.274779	1.575196	H	-1.672293	-0.601499	2.993475
C	2.944455	0.884502	2.634715	H	-0.031622	-0.025187	2.629162
H	3.207298	0.066625	0.670581	H	-0.279390	-1.274811	3.856154
C	1.116528	2.426893	2.923393	C	-2.118540	-0.032061	-3.276968
H	-0.029756	2.865357	1.168786	H	-2.907939	-0.563115	-2.732760
C	2.196757	1.728349	3.465687	H	-1.160225	-0.524397	-3.078877
H	3.794313	0.338421	3.038819	H	-2.326959	-0.133342	-4.349958

C	-0.939119	2.135692	-3.715901	H	3.450798	0.298704	-3.795518
H	-0.705175	3.143990	-3.357445	H	3.896585	1.617479	-4.907785
H	-1.243926	2.209487	-4.767901	C	4.011065	2.165245	-2.800132
H	-0.021541	1.540131	-3.681496	C	3.895409	3.667935	-3.102068
C	-3.409490	2.132642	-3.223116	H	4.267859	4.274590	-2.269359
H	-3.662969	1.943547	-4.274664	H	4.467442	3.930164	-4.002052
H	-3.364204	3.218188	-3.086724	H	2.852928	3.949784	-3.286257
H	-4.223563	1.742806	-2.604416	C	3.347808	1.792985	-1.438504
H	-3.193562	3.440391	-0.767140	C	1.867899	2.154894	-1.262639
H	-1.549315	4.024669	-1.194215	C	0.906314	5.840556	-1.119989
H	-1.909233	3.475093	0.452016	C	1.999216	5.733832	0.016779
H	-1.349620	-2.446101	-1.954130	B	1.657281	3.663847	-0.936272
H	-0.291970	-3.389824	-0.900412	O	2.538844	4.400194	-0.175782
H	-2.052425	-3.333103	-0.574914	O	0.624066	4.442848	-1.424472
Br	1.636443	-1.117275	-1.990391	C	-0.391653	6.525123	-0.697101
N	-3.599277	-1.414357	0.853961	H	-0.198185	7.564320	-0.409141
N	-4.161449	1.166994	-0.122319	H	-1.095181	6.533605	-1.536746
C	-5.890699	-1.659483	1.544180	H	-0.872097	6.016473	0.141116
C	-4.853749	-0.894570	0.952073	C	1.418223	6.482017	-2.413515
C	-5.135519	0.422245	0.468958	H	0.668880	6.340875	-3.199624
C	-6.446260	0.948394	0.595778	H	1.584304	7.556606	-2.284722
C	-7.433804	0.184877	1.177684	H	2.351141	6.024983	-2.750178
C	-7.155154	-1.124547	1.652893	C	1.416664	5.785939	1.432813
H	-5.656280	-2.657316	1.903063	H	2.197953	5.509415	2.147619
H	-6.638300	1.950184	0.222892	H	1.058488	6.790925	1.679634
H	-8.439856	0.582448	1.276121	H	0.592203	5.079990	1.552953
H	-7.952663	-1.705423	2.107222	C	3.146250	6.734406	-0.102744
C	5.501270	1.788650	-2.693623	H	2.775913	7.762492	-0.022565
H	5.999770	2.359974	-1.899585	H	3.862310	6.566245	0.708801
H	5.621560	0.721810	-2.464535	H	3.679941	6.627340	-1.050033
H	6.030284	1.991042	-3.634231	H	3.478892	0.714025	-1.323588
C	3.386265	1.377337	-3.965030	H	3.926822	2.272870	-0.639995
H	2.324991	1.620365	-4.090991	H	1.309285	1.891660	-2.164705

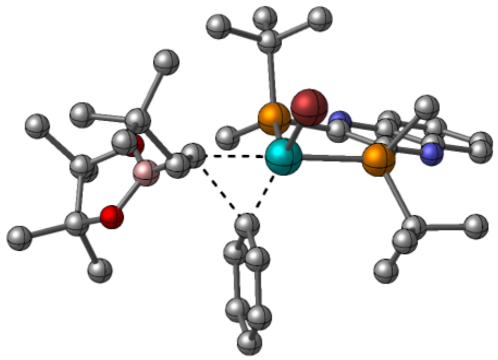

Zero-point correction= 0.875426 (Hartree/Particle)
Thermal correction to Energy= 0.926563
Thermal correction to Enthalpy= 0.927508
Thermal correction to Gibbs Free Energy= 0.793529
Sum of electronic and zero-point Energies= -6208.470311
Sum of electronic and thermal Energies= -6208.419173
Sum of electronic and thermal Enthalpies= -6208.418229
Sum of electronic and thermal Free Energies= -6208.552207

$^{4}TS_{Q}{}^{S}{}_{IV\text{-}I}$
E(scf) = -6209.37606846 a.u.
ν_{min} = -290.7 cm^{-1}

C	-2.028045	-0.919046	0.109543
C	-2.703643	-0.144371	-0.900918
P	-0.368253	-0.387844	0.768816
P	-1.826217	1.267295	-1.731528
Fe	0.517134	1.110037	-1.006953
C	0.429968	-2.013905	1.059917
C	-0.770415	0.282149	2.497275
C	-2.286362	1.171084	-3.569641
C	-2.790098	2.708014	-1.095040
C	1.167508	2.869319	-1.590779
C	0.337634	4.015374	-1.507684
C	2.082265	2.803398	-2.667982
C	0.352547	4.990658	-2.500510
H	-0.326701	4.130373	-0.654870
C	2.085883	3.781112	-3.664513
H	2.784699	1.978157	-2.719799
C	1.218181	4.875983	-3.597603
H	-0.314991	5.846677	-2.421028
H	2.784061	3.691656	-4.494525
H	1.230991	5.639057	-4.371279
C	-1.706386	-0.614358	3.322615
H	-2.704514	-0.685218	2.880764
H	-1.814881	-0.187042	4.329042
H	-1.312657	-1.629809	3.434194
C	0.576240	0.424240	3.236464
H	0.424677	0.998924	4.159368
H	1.335256	0.933763	2.637680
H	0.981400	-0.553462	3.518982
C	-1.426021	1.660659	2.303358
H	-2.380078	1.571685	1.770731
H	-0.784009	2.348400	1.741814
H	-1.631819	2.117289	3.280120
C	-2.091096	-0.272116	-4.066917
H	-2.832671	-0.953431	-3.637516
H	-1.091804	-0.644087	-3.824722
H	-2.209760	-0.292566	-5.158584
C	-1.276155	2.099409	-4.276009
H	-1.349259	3.133241	-3.921592
H	-1.479955	2.102012	-5.355066
H	-0.244465	1.766726	-4.124999
C	-3.719354	1.639855	-3.872092
H	-3.904698	1.540253	-4.950441

H	-3.873235	2.692764	-3.612094
H	-4.461151	1.037177	-3.341509
H	-3.866084	2.518597	-1.149255
H	-2.547870	3.595235	-1.686302
H	-2.512390	2.902738	-0.056452
H	0.628324	-2.461426	0.082791
H	1.384810	-1.840158	1.555238
H	-0.201167	-2.681725	1.651209
Br	1.209375	-0.821818	-2.395278
N	-2.576077	-2.011049	0.611533
N	-3.924328	-0.452699	-1.300741
C	-4.435760	-3.534157	0.694282
C	-3.817824	-2.364866	0.182260
C	-4.515957	-1.556234	-0.767758
C	-5.823871	-1.924030	-1.174046
C	-6.402815	-3.061583	-0.656467
C	-5.705087	-3.871251	0.279352
H	-3.886445	-4.135898	1.412310
H	-6.336932	-1.295897	-1.896383
H	-7.403257	-3.350076	-0.966140
H	-6.184224	-4.764601	0.669602
C	3.923210	5.405961	1.764265
H	4.818510	5.319008	1.135586
H	3.294222	6.200524	1.342394
H	4.244382	5.728027	2.763291
C	1.944034	4.240470	2.760696
H	1.383899	3.306573	2.880182
H	1.253332	4.999282	2.369849
H	2.260395	4.560254	3.761944
C	3.161660	4.068159	1.832664
C	4.096291	2.994461	2.414596
H	4.946400	2.808810	1.748786
H	4.488659	3.309193	3.390416
H	3.572422	2.044242	2.564126
C	2.696676	3.731584	0.385391
C	2.019210	2.356228	0.199836
C	4.226144	-0.745917	0.528573
C	5.001525	0.131974	-0.546481
B	3.069202	1.20321	0.109722
O	4.262044	1.382663	-0.549926
O	2.961129	-0.038205	0.695892
C	3.925339	-2.171515	0.07089
H	4.854209	-2.701172	-0.168139
H	3.422569	-2.722369	0.872237
H	3.272199	-2.171547	-0.803756
C	4.887257	-0.770227	1.910008
H	4.211279	-1.26382	2.61643
H	5.830279	-1.326467	1.891833
H	5.083186	0.238398	2.282947
C	4.938224	-0.41803	-1.974276
H	5.369823	0.32441	-2.654674
H	5.511862	-1.345758	-2.072931
H	3.906152	-0.601203	-2.279989
C	6.451564	0.447026	-0.181964
H	7.048466	-0.470389	-0.133416
H	6.88553	1.094286	-0.951553
H	6.529501	0.965981	0.776434
H	2.018099	4.538895	0.088935
H	3.557345	3.79408	-0.290479
H	1.289629	2.223738	1.000975

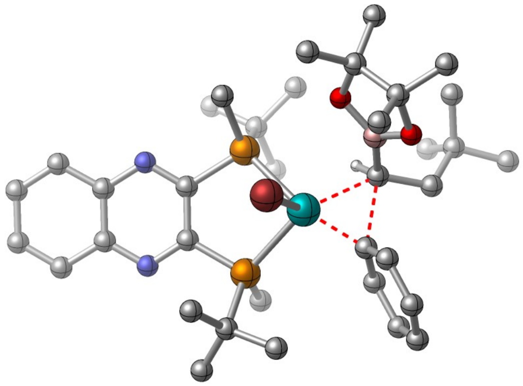

Zero-point correction= 0.874137 (Hartree/Particle)

Thermal correction to Energy= 0.925721
Thermal correction to Enthalpy= 0.926665
Thermal correction to Gibbs Free Energy= 0.790827
Sum of electronic and zero-point Energies= -6208.501932
Sum of electronic and thermal Energies= -6208.450348
Sum of electronic and thermal Enthalpies= -6208.449403
Sum of electronic and thermal Free Energies= -6208.585241

$^{4}TS_{Q}{}^{S}{}_{IV\text{-}I}$C1
E(scf) = -6209.37534118 a.u.
ν_{min} = -305.5 cm^{-1}

C	-2.695719	-0.629849	0.275903	H	-0.727594	-0.948756	4.125273
C	-3.006384	0.504464	-0.555036	C	-2.182823	-0.427246	-3.558223
P	-0.935108	-1.227237	0.370576	H	-3.030752	-0.862765	-3.016585
P	-1.656527	1.374802	-1.494021	H	-1.265232	-0.944052	-3.261003
Fe	0.512765	0.530336	-0.724967	H	-2.338864	-0.617029	-4.628361
C	-1.092502	-2.781924	-0.611013	C	-0.871751	1.650013	-4.111195
C	-0.657530	-1.825215	2.144647	H	-0.689475	2.707849	-3.884350
C	-2.071657	1.089150	-3.321710	H	-1.072982	1.571430	-5.187846
C	-2.125117	3.125702	-1.169844	H	0.041043	1.085322	-3.898045
C	1.590894	1.454744	0.637001	C	-3.366605	1.788133	-3.761874
C	2.745851	0.883442	1.220336	H	-3.571184	1.533724	-4.810544
C	0.776892	2.265117	1.470129	H	-3.284198	2.878655	-3.699699
C	3.055633	1.099995	2.562729	H	-4.225876	1.474171	-3.161519
H	3.390241	0.248340	0.620400	H	-3.171465	3.312453	-1.422936
C	1.085918	2.469905	2.814377	H	-1.462309	3.789203	-1.727219
H	-0.100246	2.746686	1.047870	H	-1.980955	3.327325	-0.105013
C	2.233363	1.894521	3.371233	H	-1.278986	-2.523158	-1.654750
H	3.945293	0.636996	2.984771	H	-0.150120	-3.333490	-0.564947
H	0.431209	3.086662	3.426916	H	-1.912861	-3.403317	-0.239605
H	2.485882	2.065332	4.414168	Br	1.428479	-1.068226	-2.358192
C	-1.435242	-3.098252	2.517445	N	-3.637650	-1.257237	0.957996
H	-2.513348	-2.961998	2.406362	N	-4.239513	0.970185	-0.651885
H	-1.224381	-3.348290	3.566427	C	-5.953442	-1.439783	1.583280
H	-1.127323	-3.956581	1.910445	C	-4.914490	-0.795055	0.864657
C	0.856089	-2.109292	2.224040	C	-5.220360	0.337784	0.047649
H	1.101928	-2.500638	3.220236	C	-6.557590	0.803478	-0.032682
H	1.447004	-1.203699	2.061482	C	-7.547382	0.157549	0.674188
H	1.169310	-2.858447	1.486417	C	-7.243913	-0.968602	1.485318
C	-1.025262	-0.680186	3.102997	H	-5.700955	-2.298691	2.198092
H	-2.104189	-0.490341	3.108356	H	-6.767328	1.665810	-0.658737
H	-0.508119	0.249004	2.842377	H	-8.573691	0.508365	0.615005

H	-8.043584	-1.459761	2.032299	O	0.744146	4.523407	-1.078233
C	5.399728	1.717923	-3.062439	C	-0.142837	5.769517	0.781098
H	6.032972	2.088960	-2.245942	H	-0.131344	6.71539	1.332485
H	5.397007	0.621423	-3.013301	H	-1.144969	5.628766	0.363809
H	5.870775	2.009476	-4.010214	H	0.050963	4.956638	1.483588
C	3.137040	1.736978	-4.133644	C	0.529685	6.909239	-1.334559
H	2.128003	2.164311	-4.143684	H	-0.519789	6.818761	-1.635363
H	3.029832	0.649740	-4.074755	H	0.66418	7.888928	-0.863401
H	3.612579	1.993849	-5.089754	H	1.142625	6.86783	-2.238056
C	3.968713	2.279329	-2.957608	C	2.596617	6.140696	1.591486
C	4.028036	3.812905	-3.049888	H	3.661721	6.069967	1.836741
H	4.579304	4.242138	-2.206945	H	2.270403	7.168792	1.783132
H	4.522392	4.126200	-3.978995	H	2.055944	5.466141	2.259277
H	3.023400	4.252308	-3.052654	C	3.334325	6.592009	-0.753293
C	3.394852	1.808601	-1.586032	H	3.136161	7.663892	-0.648627
C	1.970097	2.248526	-1.259395	H	4.366791	6.401811	-0.442394
C	0.877616	5.784193	-0.361624	H	3.2473	6.321762	-1.808214
C	2.39101	5.763633	0.125096	H	3.451897	0.715124	-1.579884
B	1.824855	3.72269	-0.779792	H	4.06682	2.170419	-0.798273
O	2.774679	4.371768	-0.029658	H	1.301133	2.068583	-2.108281

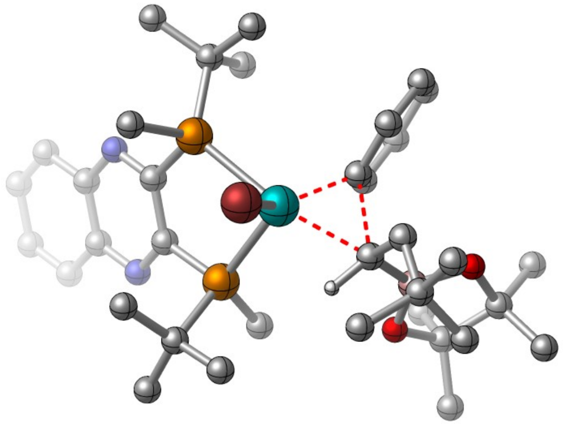

Zero-point correction= 0.874172 (Hartree/Particle)
Thermal correction to Energy= 0.925810
Thermal correction to Enthalpy= 0.926754
Thermal correction to Gibbs Free Energy= 0.790308
Sum of electronic and zero-point Energies= -6208.501169
Sum of electronic and thermal Energies= -6208.449531
Sum of electronic and thermal Enthalpies= -6208.448587
Sum of electronic and thermal Free Energies= -6208.585033

$^{4}TS_{Q}{}^{S}{}_{IV\text{-}I}C2$

E(scf) = -6209.36850005 a.u.

ν_{min} = -313.6 cm^{-1}

C	-2.751373	-0.496787	0.450576	H	-1.728748	3.335694	-3.780832
C	-3.133161	0.727411	-0.207106	H	-2.136737	2.167384	-5.043717
P	-1.092648	-1.235190	0.063216	H	-0.759932	1.864204	-3.965704
P	-1.948457	1.621550	-1.345429	C	-4.139595	2.146190	-3.139703
Fe	0.297204	0.610748	-1.087441	H	-4.524034	2.038387	-4.163076
C	-1.636592	-2.585568	-1.076105	H	-4.124282	3.216282	-2.906991
C	-0.512966	-2.183743	1.595168	H	-4.845334	1.659581	-2.460216
C	-2.739744	1.518692	-3.067394	H	-3.378905	3.507306	-0.700713
C	-2.302454	3.350836	-0.799634	H	-1.887961	4.063533	-1.515091
C	1.602596	1.222443	0.238693	H	-1.834889	3.521485	0.174644
C	2.748167	0.416445	0.469418	H	-1.939253	-2.157342	-2.031202
C	1.060028	1.923862	1.341329	H	-0.794744	-3.256189	-1.263161
C	3.302550	0.310362	1.740067	H	-2.472411	-3.145796	-0.646052
H	3.200441	-0.104579	-0.370672	Br	0.858782	-1.015563	-2.843839
C	1.629678	1.816823	2.613019	N	-3.564067	-1.101155	1.299508
H	0.191866	2.563152	1.204096	N	-4.312516	1.281166	0.012603
C	2.752341	1.012646	2.824160	C	-5.677187	-1.154146	2.449166
H	4.175955	-0.320788	1.890812	C	-4.779512	-0.538518	1.540063
H	1.187119	2.362973	3.443669	C	-5.163847	0.668450	0.878537
H	3.198164	0.936135	3.812073	C	-6.439269	1.233502	1.134650
C	-1.364444	-3.413339	1.953572	C	-7.293397	0.612946	2.019002
H	-2.401625	-3.141885	2.161688	C	-6.910129	-0.585012	2.679543
H	-0.942912	-3.884625	2.852379	H	-5.366039	-2.069649	2.943688
H	-1.353505	-4.165586	1.157734	H	-6.710503	2.150721	0.620082
C	0.916302	-2.642799	1.238551	H	-8.272046	1.039937	2.218659
H	1.359178	-3.156020	2.102539	H	-7.602416	-1.053252	3.373392
H	1.561763	-1.800531	0.975810	C	2.551278	5.082416	-0.005019
H	0.919719	-3.345951	0.397318	H	1.859286	5.054181	0.846049
C	-0.473858	-1.207931	2.781721	H	3.275742	4.271047	0.131451
H	-1.481858	-0.894844	3.075864	H	3.104817	6.029026	0.042206
H	0.115373	-0.315793	2.553628	C	2.788963	5.040517	-2.509900
H	-0.010349	-1.700256	3.647003	H	2.273689	4.923891	-3.468997
C	-2.808090	0.037059	-3.471882	H	3.554470	4.258569	-2.443767
H	-3.474677	-0.531296	-2.812289	H	3.307210	6.008217	-2.513292
H	-1.816231	-0.425535	-3.460640	C	1.794004	4.959579	-1.340056
H	-3.204509	-0.043491	-4.492660	C	0.792827	6.124443	-1.452516
C	-1.776463	2.264564	-4.010489	H	0.053944	6.091960	-0.641039

H	1.304821	7.093923	-1.398536	H	5.811694	1.568379	-4.160509
H	0.247712	6.087120	-2.404703	H	5.258906	2.053284	-5.775154
C	0.978007	3.635689	-1.377550	H	4.880174	3.057576	-4.35634
C	1.797950	2.343486	-1.400578	C	1.54737	0.868768	-5.946972
C	3.711587	1.215152	-4.478868	H	0.644054	1.383053	-6.293584
C	2.39629	1.850229	-5.134135	H	2.090917	0.506793	-6.826236
B	2.239491	1.870661	-2.830241	H	1.237105	0.022293	-5.333972
O	1.60478	2.257236	-3.985586	C	2.647018	3.106944	-5.971732
O	3.452513	1.268022	-3.050906	H	3.224662	2.871018	-6.87211
C	3.963247	-0.247487	-4.848769	H	1.683655	3.523419	-6.285238
H	4.154943	-0.359143	-5.921569	H	3.177395	3.877825	-5.408485
H	4.844953	-0.605989	-4.30598	H	0.327496	3.659918	-0.500091
H	3.116513	-0.871758	-4.56049	H	2.75481	2.52661	-0.915423
C	4.987294	2.029842	-4.714474	H	0.316172	3.652825	-2.250195

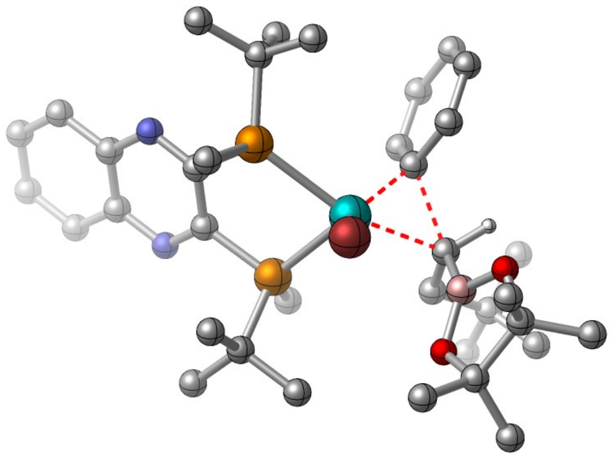

Zero-point correction= 0.874582 (Hartree/Particle)
Thermal correction to Energy= 0.926210
Thermal correction to Enthalpy= 0.927155
Thermal correction to Gibbs Free Energy= 0.790424
Sum of electronic and zero-point Energies= -6208.493918
Sum of electronic and thermal Energies= -6208.442290
Sum of electronic and thermal Enthalpies= -6208.441345
Sum of electronic and thermal Free Energies= -6208.578076

^{4}TS$_{Q}^{S}_{IV\text{-}I}$C3

E(scf) = -6209.36626937 a.u.

ν_{min} = -302.6 cm^{-1}

C	-2.549255	-1.053510	0.079746	P	-0.702421	-1.205667	0.244768
C	-3.138320	0.181396	-0.375052	P	-2.064614	1.466272	-1.182530

Fe	0.121016	0.361870	-1.501256
C	-0.452664	-2.986462	-0.152373
C	-0.457069	-1.084157	2.130646
C	-3.094326	2.150630	-2.622164
C	-2.071580	2.829304	0.063393
C	1.269528	1.868704	-2.015794
C	1.143173	3.026691	-1.203132
C	1.461181	2.074818	-3.409241
C	1.132437	4.304349	-1.759756
H	1.027274	2.909765	-0.129532
C	1.455421	3.354010	-3.954012
H	1.607220	1.209884	-4.049770
C	1.287663	4.483228	-3.138944
H	1.011158	5.167475	-1.108157
H	1.590606	3.476067	-5.026829
H	1.296283	5.480819	-3.569314
C	-1.432716	-1.945225	2.948415
H	-2.468049	-1.607904	2.849624
H	-1.159813	-1.874548	4.010395
H	-1.392127	-3.002351	2.666174
C	0.985542	-1.531508	2.431853
H	1.188782	-1.381970	3.501106
H	1.714231	-0.942030	1.873016
H	1.137559	-2.595041	2.215738
C	-0.632876	0.396669	2.504610
H	-1.646439	0.747649	2.276844
H	0.090435	1.025769	1.976630
H	-0.472077	0.528977	3.582982
C	-3.658382	0.978584	-3.445362
H	-4.446497	0.444530	-2.905647
H	-2.872651	0.265351	-3.711529
H	-4.095119	1.371390	-4.373534
C	-2.084717	2.935457	-3.483174
H	-1.591372	3.734731	-2.918746
H	-2.609875	3.401174	-4.327909
H	-1.305868	2.281396	-3.885080
C	-4.234165	3.084082	-2.181902
H	-4.786164	3.416081	-3.072187
H	-3.859817	3.982212	-1.678406
H	-4.935661	2.576505	-1.515008
H	-3.075644	3.012662	0.457042
H	-1.694208	3.741509	-0.407844
H	-1.402593	2.573665	0.887630
H	-0.712571	-3.125563	-1.205459
H	0.602538	-3.241405	-0.024597
H	-1.072181	-3.640182	0.466235
Br	-0.532661	-1.126642	-3.402369
N	-3.298365	-2.075139	0.449483
N	-4.444672	0.371335	-0.329450
C	-5.490163	-2.988105	0.837020
C	-4.650228	-1.913433	0.450157
C	-5.230056	-0.655336	0.098702
C	-6.636746	-0.490188	0.165147
C	-7.429368	-1.547766	0.554177
C	-6.854371	-2.802894	0.887316
H	-5.028533	-3.936553	1.095554
H	-7.057674	0.474250	-0.103758
H	-8.507781	-1.427313	0.604369
H	-7.502332	-3.621548	1.187260
C	3.936454	-1.515665	-3.208071
H	3.054795	-1.809027	-3.791203
H	4.145345	-0.462166	-3.427301
H	4.793158	-2.098849	-3.568856
C	4.939691	-1.303918	-0.904579
H	4.789496	-1.472595	0.169899
H	5.151635	-0.239764	-1.049996
H	5.831794	-1.866624	-1.208570
C	3.709666	-1.759925	-1.705297
C	3.488695	-3.267799	-1.475469
H	2.604069	-3.624134	-2.018498
H	4.352683	-3.850609	-1.819473
H	3.340492	-3.488231	-0.410118
C	2.414372	-1.041298	-1.224211
C	2.447729	0.514446	-1.309920
C	4.358286	2.634636	1.197416
C	3.468071	1.74239	2.148406
B	3.039775	1.22407	-0.047181
O	2.504958	1.155881	1.22086
O	4.177065	1.993084	-0.098491
C	3.857165	4.075218	1.057846
H	4.024501	4.644651	1.978026
H	4.403013	4.563115	0.243943
H	2.793412	4.109407	0.811713
C	5.848089	2.633061	1.526024

H	6.382225	3.25985	0.804016	C	4.234465	0.577317	2.78167
H	6.024226	3.041558	2.527221	H	4.962884	0.932847	3.517293
H	6.271983	1.627204	1.477525	H	3.527136	-0.084466	3.29053
C	2.697889	2.507611	3.218549	H	4.762133	-0.008387	2.022776
H	2.096003	1.809097	3.809158	H	1.616307	-1.477716	-1.833091
H	3.38835	3.020503	3.897079	H	3.104485	0.758577	-2.140496
H	2.024321	3.248114	2.78048	H	2.215948	-1.342604	-0.191478

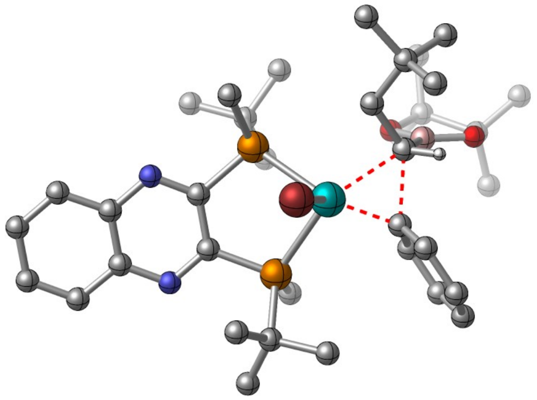

Zero-point correction= 0.874232 (Hartree/Particle)
Thermal correction to Energy= 0.925095
Thermal correction to Enthalpy= 0.926039
Thermal correction to Gibbs Free Energy= 0.791755
Sum of electronic and zero-point Energies= -6208.492037
Sum of electronic and thermal Energies= -6208.441174
Sum of electronic and thermal Enthalpies= -6208.440230
Sum of electronic and thermal Free Energies= -6208.574514

^{6}**TS**$_{Q}{}^{S}{}_{IV\text{-}I}$
E(scf) = -6209.35264839 a.u.
ν_{min} = -403.79 cm^{-1}

C	-2.800223	-1.082065	0.182862	Fe	0.360191	0.153851	-0.552006
C	-3.120354	0.177003	-0.432818	C	-0.898521	-3.252856	0.289645
P	-1.044009	-1.456586	0.635424	C	-1.018281	-1.272167	2.517304
P	-1.754931	1.304538	-0.998395	C	-2.197665	1.759794	-2.783747

C	-2.063919	2.812399	0.010363
C	1.884437	1.172246	0.425604
C	3.035022	0.363441	0.743258
C	1.482061	2.063339	1.486830
C	3.718942	0.468159	1.948023
H	3.375273	-0.372554	0.017801
C	2.155205	2.157571	2.694714
H	0.612030	2.695916	1.322828
C	3.293430	1.366524	2.940691
H	4.579112	-0.172828	2.135913
H	1.795403	2.840255	3.462630
H	3.823887	1.439160	3.886379
C	-1.934187	-2.276568	3.231086
H	-2.977599	-2.176799	2.916967
H	-1.886760	-2.100110	4.314148
H	-1.621413	-3.310937	3.050979
C	0.444706	-1.482304	2.952778
H	0.521741	-1.356445	4.040577
H	1.117111	-0.757875	2.482833
H	0.802241	-2.489809	2.709887
C	-1.448308	0.169479	2.841322
H	-2.481563	0.370407	2.536264
H	-0.789724	0.899638	2.359223
H	-1.381845	0.335226	3.924273
C	-2.553240	0.464897	-3.538388
H	-3.505826	0.043676	-3.201192
H	-1.772272	-0.296093	-3.425139
H	-2.646434	0.688656	-4.609084
C	-0.917799	2.368025	-3.392404
H	-0.531124	3.204369	-2.798863
H	-1.139824	2.741446	-4.400954
H	-0.130167	1.613555	-3.481215
C	-3.351462	2.770397	-2.880444
H	-3.579092	2.950118	-3.939834
H	-3.084182	3.735170	-2.434879
H	-4.257344	2.399713	-2.393506
H	-3.101831	3.145905	-0.077902
H	-1.376621	3.595944	-0.316363
H	-1.854348	2.582748	1.059290
H	-0.821329	-3.385898	-0.793651
H	0.022191	-3.628506	0.746413
H	-1.758302	-3.812089	0.667537

Br	0.956668	-1.134601	-2.496713
N	-3.736917	-1.969910	0.472126
N	-4.373096	0.529470	-0.667607
C	-6.066322	-2.568651	0.471704
C	-5.029510	-1.643172	0.193215
C	-5.355109	-0.363093	-0.358474
C	-6.711494	-0.033083	-0.604053
C	-7.700264	-0.949935	-0.318439
C	-7.376404	-2.223072	0.219269
H	-5.796837	-3.535306	0.886985
H	-6.937718	0.943113	-1.022875
H	-8.740763	-0.701944	-0.507615
H	-8.174246	-2.928389	0.433386
C	5.369305	1.645822	-3.585519
H	6.133145	1.967427	-2.865458
H	5.312446	0.550161	-3.547226
H	5.715065	1.927905	-4.588574
C	2.970656	1.790962	-4.300550
H	2.007945	2.299316	-4.176725
H	2.787752	0.715035	-4.198670
H	3.319337	1.988626	-5.322937
C	4.002766	2.282942	-3.269360
C	4.129470	3.812376	-3.369022
H	4.808512	4.210030	-2.606168
H	4.511919	4.104561	-4.356083
H	3.159135	4.302002	-3.233275
C	3.601617	1.826629	-1.831717
C	2.250935	2.287063	-1.329955
C	0.961905	5.754083	-0.616250
C	2.370850	5.796715	0.093653
B	2.027930	3.731012	-0.853172
O	3.006902	4.578749	-0.381975
O	0.775441	4.332994	-0.862079
C	-0.201524	6.260712	0.231164
H	-0.055219	7.313468	0.497426
H	-1.134723	6.182789	-0.337388
H	-0.316062	5.681330	1.150347
C	0.946046	6.439980	-1.986847
H	-0.000213	6.210225	-2.487985
H	1.031956	7.527620	-1.895268
H	1.760110	6.077819	-2.622338
C	2.284263	5.695720	1.619526

H	3.287966	5.523644	2.021448	H	4.205744	6.919690	0.230460
H	1.887019	6.616917	2.059132	H	3.459589	6.988094	-1.372556
H	1.654979	4.857640	1.926477	H	3.627755	0.730192	-1.843158
C	3.249521	6.980621	-0.300305	H	4.383996	2.154032	-1.134003
H	2.769627	7.927737	-0.029239	H	1.435528	1.935007	-1.958391

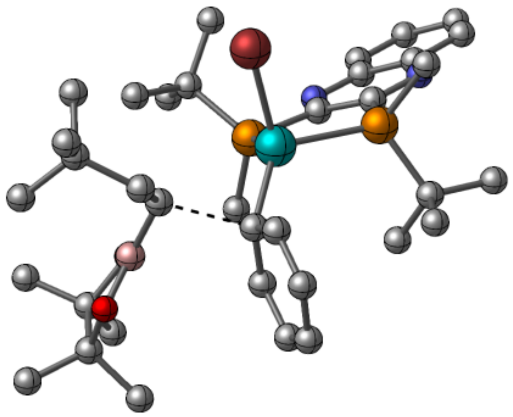

Zero-point correction= 0.872130 (Hartree/Particle)
Thermal correction to Energy= 0.924358
Thermal correction to Enthalpy= 0.925302
Thermal correction to Gibbs Free Energy= 0.786326
Sum of electronic and zero-point Energies= -6208.480518
Sum of electronic and thermal Energies= -6208.428290
Sum of electronic and thermal Enthalpies= -6208.427346
Sum of electronic and thermal Free Energies= -6208.566322

$^{2}I_{Q}$

E(scf) = -5330.47280038 a.u.

ν_{min} = 19.96 cm^{-1}

Fe	4.382461	7.757253	13.980594	H	2.754554	6.532246	16.109534
P	5.481533	7.602186	15.953553	C	5.355134	6.154587	18.406998
P	6.333487	7.366052	13.001422	H	6.438246	6.013290	18.358042
C	7.210908	7.165980	15.620389	H	4.915405	5.308873	18.952763
C	7.571798	6.961644	14.272345	H	5.152288	7.062298	18.986269
C	4.733136	6.225720	17.003940	C	5.540442	9.101669	17.011948
C	4.968782	4.909903	16.242419	H	5.855435	9.949175	16.395219
H	4.550430	4.950274	15.227286	H	6.261461	8.963197	17.823044
H	4.475888	4.085377	16.772474	H	4.551149	9.314382	17.427985
H	6.034837	4.672506	16.161360	C	6.939499	8.888630	12.064939
C	3.222097	6.506602	17.102297	C	6.910256	10.054527	13.069606
H	3.013802	7.460571	17.600676	H	5.892999	10.230439	13.445680
H	2.734287	5.712376	17.681382	H	7.246684	10.974968	12.576665

H	7.565586	9.868620	13.927927	N	8.060453	6.994570	16.644943
C	8.360509	8.697600	11.509306	N	8.783824	6.528455	13.893490
H	9.088524	8.510612	12.302407	C	9.681882	6.318524	14.923801
H	8.655236	9.609998	10.973682	C	10.982317	5.865453	14.628008
H	8.410741	7.864899	10.799182	C	11.917821	5.667473	15.640940
C	5.958850	9.165987	10.911416	C	11.572654	5.917559	16.977997
H	5.984809	8.373254	10.155508	C	10.291921	6.361606	17.297804
H	6.239457	10.105142	10.417971	C	9.327170	6.567996	16.292836
H	4.928350	9.262849	11.268307	H	11.233584	5.681796	13.586318
C	6.365379	5.953961	11.835403	H	12.917787	5.320501	15.393511
H	6.023003	5.063446	12.371932	H	12.304717	5.764227	17.766772
H	7.384271	5.781855	11.476417	H	10.004284	6.556641	18.328044
H	5.695645	6.139428	10.991310	Br	2.575278	8.054427	12.562870

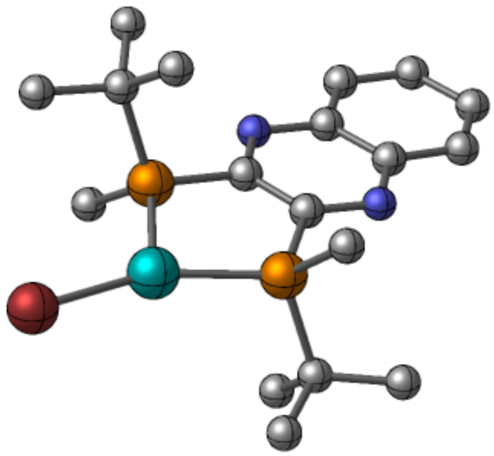

Zero-point correction= 0.428604 (Hartree/Particle)
Thermal correction to Energy= 0.457283
Thermal correction to Enthalpy= 0.458227
Thermal correction to Gibbs Free Energy= 0.369369
Sum of electronic and zero-point Energies= -5330.044196
Sum of electronic and thermal Energies= -5330.015517
Sum of electronic and thermal Enthalpies= -5330.014573
Sum of electronic and thermal Free Energies= -5330.103431

^{4}I$_Q$

E(scf) = -5330.50279157 a.u.

ν_{min} = 26.67 cm^{-1}

Fe	4.307320	8.192308	14.092793	C	7.504499	7.120673	14.376897
P	5.350523	7.760688	16.101120	C	4.694743	6.141263	16.842327
P	6.220258	7.405974	13.060829	C	4.779193	5.074084	15.735180
C	7.131964	7.373375	15.743369	H	4.209599	5.373137	14.844853

H	4.358033	4.127091	16.096582	H	8.020744	7.285506	10.507370
H	5.813763	4.885343	15.426971	C	5.937022	9.046994	10.871712
C	3.216577	6.393584	17.196530	H	5.629701	8.186007	10.265112
H	3.106651	7.152265	17.979469	H	6.325364	9.813549	10.188107
H	2.761166	5.465507	17.566644	H	5.046904	9.446743	11.369660
H	2.644146	6.726133	16.321263	C	6.259532	5.808873	12.140568
C	5.459245	5.673223	18.088216	H	5.732638	5.049261	12.727089
H	6.518301	5.496637	17.877399	H	7.282059	5.466670	11.955118
H	5.024300	4.731284	18.449742	H	5.736486	5.929775	11.186680
H	5.392793	6.401588	18.903753	N	8.005729	7.286318	16.729474
C	5.495397	8.921739	17.522350	N	8.719173	6.715710	14.054869
H	5.846557	9.885436	17.137968	C	9.633025	6.594656	15.062173
H	6.196376	8.566328	18.282809	C	10.952274	6.170665	14.767105
H	4.506651	9.071483	17.966659	C	11.884337	6.076663	15.778855
C	7.026189	8.664197	11.891835	C	11.536186	6.398257	17.115898
C	7.394726	9.896088	12.738204	C	10.257833	6.807802	17.431216
H	6.527492	10.277493	13.291615	C	9.279432	6.910389	16.411588
H	7.755583	10.699842	12.083392	H	11.199350	5.934559	13.736067
H	8.188245	9.670713	13.460054	H	12.897713	5.755789	15.554790
C	8.266047	8.132875	11.157514	H	12.287914	6.317674	17.896006
H	9.047143	7.814780	11.852874	H	9.968980	7.053214	18.449094
H	8.675411	8.928313	10.519561	Br	2.829766	9.843089	13.319604

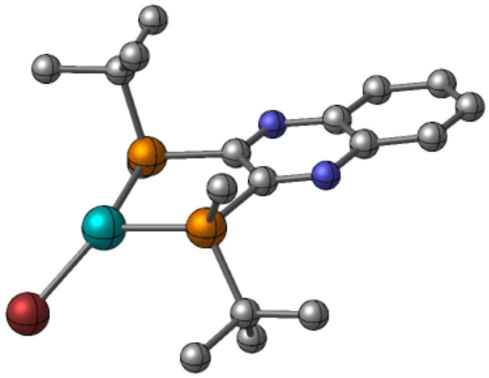

Zero-point correction= 0.429963 (Hartree/Particle)
Thermal correction to Energy= 0.458562
Thermal correction to Enthalpy= 0.459506
Thermal correction to Gibbs Free Energy= 0.370383
Sum of electronic and zero-point Energies= -5330.072828
Sum of electronic and thermal Energies= -5330.044230
Sum of electronic and thermal Enthalpies= -5330.043286
Sum of electronic and thermal Free Energies= -5330.132408

$^{6}I_{Q}$

E(scf) = -5330.48645893 a.u.
ν_{min} = 23.11 cm^{-1}

Fe	4.197580	7.607420	13.886977
P	5.381936	7.533709	15.978276
P	6.352287	7.269527	12.838631
C	7.101665	7.093118	15.563723
C	7.496515	6.909873	14.223482
C	4.674079	6.180990	17.085183
C	4.917746	4.839434	16.373469
H	4.505229	4.836786	15.354087
H	4.425974	4.032146	16.930566
H	5.985296	4.605749	16.306576
C	3.160488	6.452231	17.185746
H	2.948378	7.420434	17.654168
H	2.683226	5.674232	17.795026
H	2.679528	6.448564	16.198010
C	5.306306	6.166744	18.485406
H	6.390371	6.036760	18.429683
H	4.881338	5.337930	19.067670
H	5.098903	7.094099	19.031023
C	5.487518	9.055059	17.000765
H	5.739182	9.895692	16.346400
H	6.261295	8.948658	17.766245
H	4.519663	9.257728	17.469126
C	6.998061	8.811214	11.957944
C	7.130945	9.917817	13.019794
H	6.184495	10.095177	13.547178
H	7.417128	10.858026	12.531677
H	7.897365	9.675505	13.763620
C	8.350278	8.565207	11.270696
H	9.106391	8.219971	11.980890
H	8.698426	9.502482	10.816042
H	8.267528	7.821078	10.470471
C	5.936449	9.211922	10.916286
H	5.774682	8.422511	10.172454
H	6.273320	10.107944	10.379111
H	4.974854	9.444172	11.388067
C	6.584622	5.866706	11.675000
H	6.100403	4.977289	12.091217
H	7.651249	5.662775	11.547464
H	6.127415	6.097928	10.708416
N	7.950785	6.945036	16.607072
N	8.747631	6.538751	13.872832
C	9.637469	6.369666	14.915545
C	10.964922	5.988260	14.642643
C	11.890757	5.819602	15.673324
C	11.505850	6.031506	17.003802
C	10.195978	6.409542	17.301191
C	9.241559	6.582705	16.280196
H	11.246783	5.832146	13.603921
H	12.911059	5.524623	15.441014
H	12.226649	5.901421	17.807304
H	9.879078	6.576526	18.328033
Br	2.774415	9.447217	13.443492

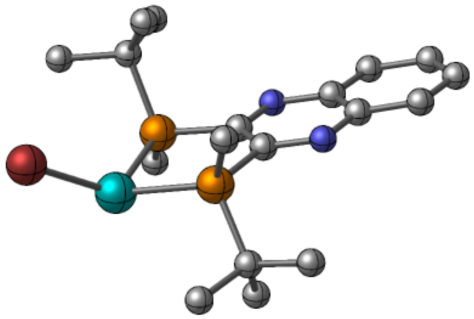

Zero-point correction= 0.427947 (Hartree/Particle)
Thermal correction to Energy= 0.456739
Thermal correction to Enthalpy= 0.457684
Thermal correction to Gibbs Free Energy= 0.367879
Sum of electronic and zero-point Energies= -5330.058512
Sum of electronic and thermal Energies= -5330.029720
Sum of electronic and thermal Enthalpies= -5330.028775
Sum of electronic and thermal Free Energies= -5330.118580

10. References

[1] Liu, L.; Aguilera, M. C.; Lee, W.; Youshaw, C. R.; Neidig, M. L.; Gutierrez, O. General method for iron-catalyzed multicomponent radical cascades–cross-couplings. *Science* **2021,** *374*, 432–439.

[2] Kutner, A.; Perlman, K. L.; Lago, A.; Schnoes, H. K.; DeLuca, H. F.; Sicinski, R. R. Novel convergent synthesis of side-chain-modified analogs of 1.alpha.,25-dihydroxycholecalciferol and 1.alpha.,25-dihydroxyergocalciferol. *J. Org. Chem.* **1988,** *53*, 3450–3457.

[3] Dudnik, A. S.; Fu, G. C. Nickel-Catalyzed Coupling Reactions of Alkyl Electrophiles, Including Unactivated Tertiary Halides, To Generate Carbon–Boron Bonds. *J. Am. Chem. Soc.* **2012,** *134*, 10693–10697.

[4] Wang, X.-X.; Lu, X.; He, S.-J.; Fu, Y. Nickel-catalyzed three-component olefin reductive dicarbofunctionalization to access alkylborates. *Chem. Sci.* **2020,** *11*, 7950–7956.

[5] Huang, M.; Hu, J.; Shi, S.; Friedrich, A.; Krebs, J.; Westcott, S. A.; Radius, U.; Marder, T. B. Selective, Transition Metal-free 1,2-Diboration of Alkyl Halides, Tosylates, and Alcohols. *Chem. Eur. J.* **2022,** *28*, e202200480.

[6] Barber, T.; Argent, S. P.; Ball, L. T. Expanding Ligand Space: Preparation, Characterization, and Synthetic Applications of Air-Stable, Odorless Di-tert-alkylphosphine Surrogates. *ACS Catal.* **2020,** *10*, 5454–5461.

[7] Ajvazi, N.; Stavber, S. Direct halogenation of alcohols with halosilanes under catalyst- and organic solvent-free reaction conditions. *Tetrahedron Lett.* **2016,** *57*, 2430–2433.

[8] Shim, E.; Zakarian, A. Stereoselective α-Tertiary Alkylation of N-(Arylacetyl)oxazolidinones. *Synlett* **2020,** *31*, 683–686.

[9] Andersen, T. L.; Frederiksen, M. W.; Domino, K.; Skrydstrup, T. Direct Access to α,α-Difluoroacylated Arenes by Palladium-Catalyzed Carbonylation of (Hetero)Aryl Boronic Acid Derivatives. *Angew. Chem. Int. Ed.* **2016,** *55*, 10396–10400.

[10] Chierchia, M.; Xu, P.; Lovinger, G. J.; Morken, J. P. Enantioselective Radical Addition/Cross-Coupling of Organozinc Reagents, Alkyl Iodides, and Alkenyl Boron Reagents. *Angew. Chem. Int. Ed.* **2019,** *58*, 14245–14249.

[11] Campbell, M. W.; Compton, J. S.; Kelly, C. B.; Molander, G. A. Three-Component Olefin Dicarbofunctionalization Enabled by Nickel/Photoredox Dual Catalysis. *J. Am. Chem. Soc.* **2019,** *141*, 20069–20078.
[12] Scholz, R.; Hellmann, G.; Rohs, S.; Özdemir, D.; Raabe, G.; Vermeeren, C.; Gais, H.-J. Enantioselective Synthesis, Configurational Stability, and Reactivity of Lithium α-tert-Butylsulfonyl Carbanion Salts. *Eur. J. Org. Chem.* **2010,** *2010*, 4588–4616.
[13] Horoiwa, S.; Yokoi, T.; Masumoto, S.; Minami, S.; Ishizuka, C.; Kishikawa, H.; Ozaki, S.; Kitsuda, S.; Nakagawa, Y.; Miyagawa, H. Structure-based virtual screening for insect ecdysone receptor ligands using MM/PBSA. *Bioorg. Med. Chem.* **2019,** *27*, 1065–1075.
[14] Koike, T.; Okumura, R.; Kato, T.; Abe, M.; Akita, M. One-Electron Injection-triggered Radical Reaction of Alkyl Benzoates Promoted by 1,4-Bis(diphenylamino)benzene Photocatalysis. *ChemCatChem* **2023,** *15*, e202201311.
[15] Wei, X.; Shu, W.; García-Domínguez, A.; Merino, E.; Nevado, C. Asymmetric Ni-Catalyzed Radical Relayed Reductive Coupling. *J. Am. Chem. Soc.* **2020,** *142*, 13515–13522.
[16] (a) Lee, C.; Yang, W.; Parr, R. G. Development of the Colle-Salvetti Correlation-Energy Formula into a Functional of the Electron Density. *Phys. Rev. B* **1988**, *37*, 785–789. (b) Becke, A. D. Density-Functional Thermochemistry. III. The Role of Exact Exchange. *J. Chem. Phys.* **1993**, *98*, 5648–5652.
[17] (a) Grimme, S. Accurate description of van der Waals complexes by density functional theory including empirical corrections. *J. Comput. Chem.* **2004**, *25*, 1463–1473. (b) Grimme, S.; Antony, J.; Ehrlich, S.; Krieg, H. A consistent and accurate ab initio parametrization of density functional dispersion correction (DFT-D) for the 94 elements H-Pu. *J. Chem. Phys.* **2010**, *132*, 154104. (c) Grimme, S. Density functional theory with London dispersion corrections. *WIRES Comput. Mol. Sci.* **2011**, *1*, 211-228. (d) Ehrlich, S.; Moellmann, J.; Grimme, S. Dispersion-Corrected Density Functional Theory for Aromatic Interactions in Complex Systems. *Acc. Chem. Res.* **2012**, *46*, 916–926.
[18] (a) Petersson, G. A.; Tensfeldt, T. G.; Montgomery, J. A., Jr. A Complete Basis Set Model Chemistry. III. The Complete Basis Set-quadratic Configuration Interaction Family of Methods. *J. Chem. Phys.* **1991**, *94*, 6091–6101. (b) Petersson, G. A.; Bennett, A.; Tensfeldt, T. G.; Al-Laham, M. A.; Shirley, W. A.; Mantzaris, J. A Complete Basis Set Model Chemistry. I. The Total Energies of Closed-shell Atoms and Hydrides of the First-row Elements. *J. Chem. Phys.* **1988**, *89*, 2193–2218.
[19] Marenich, A. V.; Cramer, C. J.; Truhlar, D. G. Universal Solvation Model Based on Solute Electron Density and on a Continuum Model of the Solvent Defined by the Bulk Dielectric Constant and Atomic Surface Tensions. *J. Phys. Chem. B* **2009**, *113*, 6378–6396.
[20] Gaussian 16, Revision C.01, Frisch, M. J.; Trucks, G. W.; Schlegel, H. B.; Scuseria, G. E.; Robb, M. A.; Cheeseman, J. R.; Scalmani, G.; Barone, V.; Petersson, G. A.; Nakatsuji, H.; Li, X.; Caricato, M.; Marenich, A. V.; Bloino, J.; Janesko, B. G.; Gomperts, R.; Mennucci, B.; Hratchian, H. P.; Ortiz, J. V.; Izmaylov, A. F.; Sonnenberg, J. L.; Williams-Young, D.; Ding, F.; Lipparini, F.; Egidi, F.; Goings, J.; Peng, B.; Petrone, A.; Henderson, T.; Ranasinghe, D.; Zakrzewski, V. G.; Gao, J.; Rega, N.; Zheng, G.; Liang, W.; Hada, M.; Ehara, M.; Toyota, K.; Fukuda, R.; Hasegawa, J.; Ishida, M.; Nakajima, T.; Honda, Y.; Kitao, O.; Nakai, H.; Vreven, T.; Throssell, K.; Montgomery, J. A., Jr.; Peralta, J. E.; Ogliaro, F.; Bearpark, M. J.; Heyd, J. J.; Brothers, E. N.; Kudin, K. N.; Staroverov, V. N.; Keith, T. A.; Kobayashi, R.; Normand, J.; Raghavachari, K.; Rendell, A. P.; Burant, J. C.; Iyengar, S. S.; Tomasi, J.; Cossi, M.; Millam, J. M.; Klene, M.; Adamo, C.; Cammi, R.; Ochterski, J. W.; Martin, R. L.; Morokuma, K.; Farkas, O.; Foresman, J. B.; Fox, D. J. Gaussian, Inc., Wallingford CT, 2016.
[21] Legault, C. Y. (2009) CYLview, 1.0b, Université de Sherbrooke: Sherbrooke, Canada, http://www.cylview.org.
[22] Lu, T.; Chen, F., Multiwfn: A multifunctional wavefunction analyzer. J. Comp. Chem. 2012, 33, 580-592.

[23] Humphrey, W.; Dalke, A.; Schulten, K., VMD – Visual Molecular Dynamics. J. Mol. Graphics 1996, 14, 33-38.
[24] Unnikrishnan*,* A.; Sunoj, R. B. Insights into the role of noncovalent interactions in distal functionalization of the aryl C(sp^2)–H bond. *Chem. Sci.*, **2019**, *10*, 3826-3835.
